# Accessing Highly Substituted Indoles via B(C_6_F_5_)_3_-Catalyzed Secondary Alkyl Group Transfer

**DOI:** 10.1021/acs.joc.4c00025

**Published:** 2024-02-23

**Authors:** Salma A. Elsherbeni, Rebecca L. Melen, Alexander P. Pulis, Louis C. Morrill

**Affiliations:** † Cardiff Catalysis Institute, School of Chemistry, Cardiff University, Main Building, Park Place, Cardiff, CF10 3AT, U.K.; ‡ Department of Pharmaceutical Chemistry, Faculty of Pharmacy, Tanta University, Tanta, Egypt; § Cardiff Catalysis Institute, School of Chemistry, Cardiff University, Translational Research Hub, Maindy Road, Cathays, Cardiff, CF24 4HQ, U.K.; ∥ School of Chemistry, University of Leicester, Leicester, LE1 7RH, U.K.

## Abstract

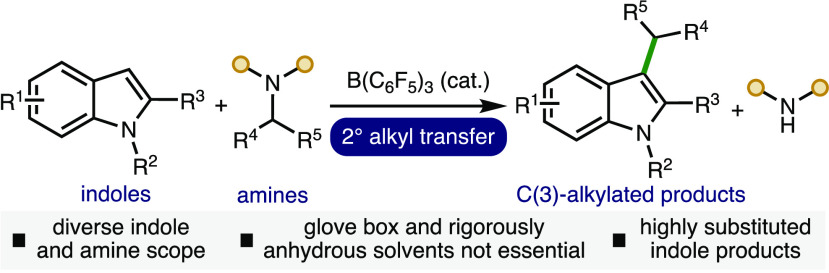

Herein, we report a synthetic method to access a range of highly substituted indoles via the B(C_6_F_5_)_3_-catalyzed transfer of 2° alkyl groups from amines. The transition-metal-free catalytic approach has been demonstrated across a broad range of indoles and amine 2° alkyl donors, including various substituents on both reacting components, to access useful C(3)-alkylated indole products. The alkyl transfer process can be performed using Schlenk line techniques in combination with commercially available B(C_6_F_5_)_3_·*n*H_2_O and solvents, which obviates the requirement for specialized equipment (e.g., glovebox).

Indole-containing molecules have diverse applications, spanning functional materials, pigments, and pharmaceuticals.^1^ As such, the development of methods to access indoles with various substitution patterns has received considerable attention from the synthetic community.^2^ Highly substituted indole frameworks, for example those bearing substitution at the 1-, 2-, and 3-positions, occur within biologically active molecules such as beclabuvir (antiviral drug for the treatment of hepatitis C virus (HCV) infection), deleobuvir (nonnucleoside inhibitor of HCV NS5B RNA polymerase), and bazedoxifene (selective estrogen receptor modulator) (Scheme 1). Despite their importance, relatively few methods exist for their synthesis, especially for those that contain 2° alkyl groups at the C(3)-position, which are typically accessed via C(3)-alkylation of 1,2-disubstituted indoles.^3−7^ Using 1,2-dimethylindole as a representative example, existing synthetic approaches include the Pt-catalyzed hydroarylation with styrene, reported in 2006 by Widenhoefer and co-workers,^3^ which produced the corresponding C(3)-alkylated indole in 55% yield as a (1:1.1) mixture of linear and branched isomers (Scheme 2A). In 2011, Tsuchimoto and co-workers disclosed an In-catalyzed reductive alkylation protocol employing phenylacetylene and Ph_2_MeSiH as the reductant, which produced the indole product in 98% yield (Scheme 2B).^4^ In 2016, the same group reported that the alkyne could be replaced with acetophenone using similar reaction conditions to give the C(3)-alkylated indole product.^5^ In 2020, Melen and co-workers disclosed the B(C_6_F_5_)_3_-catalyzed C(3)-alkylation of 1,2-dimethylindole with a donor–acceptor diazo compound to give the indole product in 81% yield (Scheme 2C).^6^ Recently, the same group described the borane-catalyzed C(3)-allylation of indoles (including 1,2-dimethylindole) with allyl esters.^7^ Despite these advances, it remains necessary to develop new synthetic approaches that avoid the use of catalysts based on precious metals and diversify the range of accessible indole-containing molecules. Building upon our ongoing interest in the applications of boranes in catalysis,^8,9^ we recently discovered that B(C_6_F_5_)_3_ could be employed as a catalyst for the direct C(3)-alkylation of indoles and oxindoles using amines as alkyl donors,^10^ whereby the mechanism of alkyl transfer is initiated by B(C_6_F_5_)_3_-mediated α-*N* C(sp^3^)–H hydride abstraction to form electrophilic iminium ions.^11−13^ However, the method was restricted to the transfer of 1° alkyl groups, and almost exclusively to C(3)-methylation, in order to mitigate against anticipated unproductive pathways resulting from enamine formation when amine alkyl donors that contain β-*N* C(sp^3^)–H bonds were employed. Despite the aforementioned challenge, herein, we describe a significant advance of this approach to include the B(C_6_F_5_)_3_-catalyzed transfer of 2° alkyl groups for the first time, enabling access to a more diverse range of valuable highly substituted indoles (*vide infra*).

**Scheme 1:**
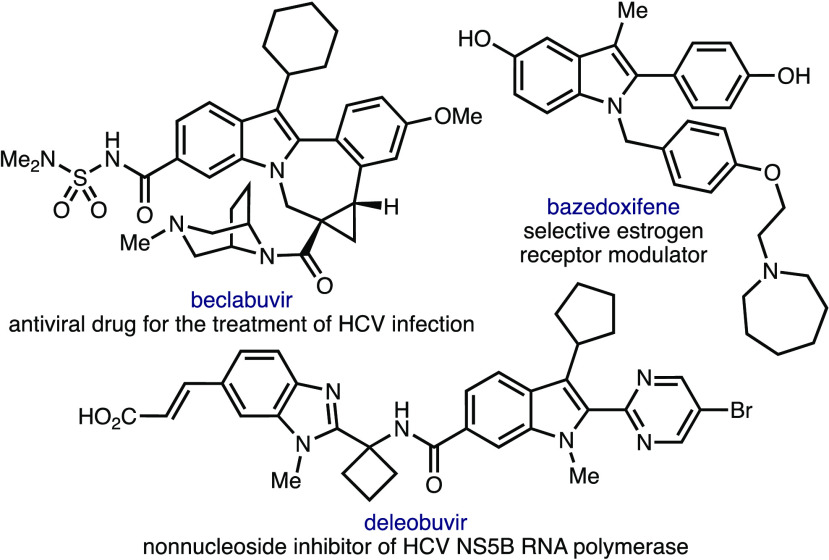
Biologically Active Molecules Containing Highly Substituted Indoles

**Scheme 2:**
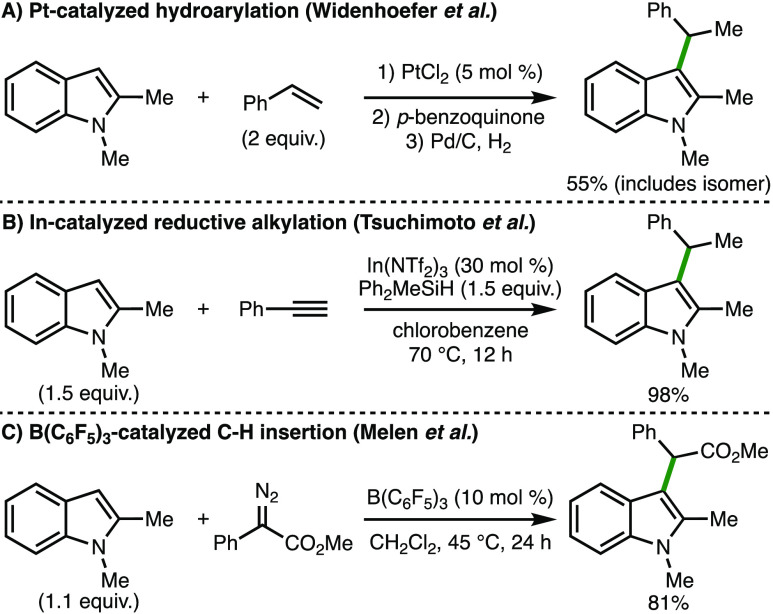
Existing Synthetic Approaches

For reaction optimization, the C(3)-alkylation of 1,2-dimethylindole **1** to form **2** was investigated using a selection of mono- and diarylamines **3**–**7** as secondary alkyl group transfer reagents (Table 1).^14,15^ Employing B(C_6_F_5_)_3_ (10 mol %)^16^ as the catalyst with diarylamine **7** (1.2 equiv) in dichloroethane (DCE) at 50 °C for 18 h under argon, 62% conversion to **2** was observed (entry 1). Monoarylamines **3** and **4** were found to be unreactive under these reaction conditions (entry 2), whereas less electron-rich diarylamines **5** and **6** gave 54% and 50% conversion to **2**, respectively (entry 3). Increasing the concentration ([**1**] = 2 M) resulted in 84% conversion to **2** (entry 4), which could be isolated in 58% yield. The discrepancy in conversion vs isolated yield in this case was attributed to the challenging separation of **2** from residual **1** via silica gel chromatography. No product formation was observed in the absence of B(C_6_F_5_)_3_ (entry 5), whereas only 55% conversion to **2** occurred upon lowering the catalyst loading to 5 mol % (entry 6). Various other modifications to the reaction parameters, including switching solvent to dichloromethane (DCM), cyclohexane, or toluene (entry 7), reducing the reaction time to 6 h (entry 8), or lowering the reaction temperature to 40 °C, all diminished the observed conversion to **2**. As such, the optimized reaction conditions, which are mild, are those represented by Table 1, entry 4.

**Table 1:**
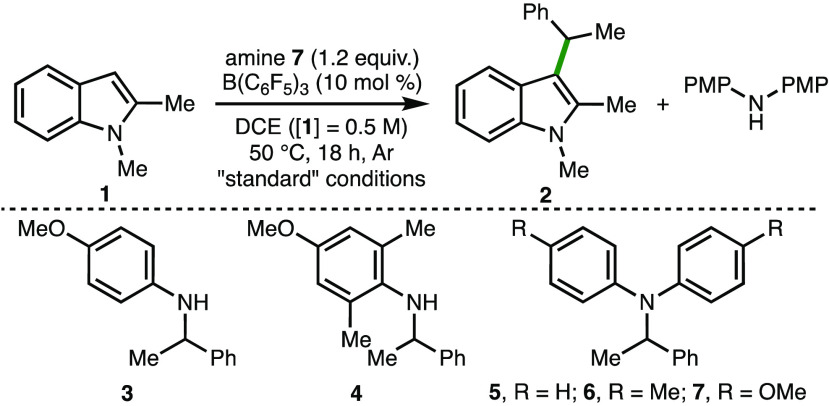
Reaction Optimization^a^

entry	variation from “standard” conditions	yield^b^ (%)
1	none	62
2	amine **3** or **4**	<2
3	amine **5** or **6**	54, 50
4	[**1**] = 2 M	84 (58)
5^c^	no B(C_6_F_5_)_3_	<2
6^c^	B(C_6_F_5_)_3_ (5 mol %)	55
7^c^	DCM, cyclohexane, toluene	77, 80, 70
8^c^	6 h	74
9^c^	40 °C	66

a Reactions performed with 0.1 mmol of **1**.

b As determined by ^1^H NMR analysis of the crude reaction mixture with 1,3,5-trimethylbenzene as the internal standard. Isolated yield given in parentheses.

c [**1**] = 2 M. PMP = 4-OMeC_6_H_4_.

The commercially available borane catalyst, B(C_6_F_5_)_3_, which readily forms the B(C_6_F_5_)_3_·*n*H_2_O (*n* = 0, 1) adduct when exposed to moisture in air, is typically transferred to an argon or nitrogen filled glovebox and purified via sublimation prior to use. Alternatively, the active B(C_6_F_5_)_3_ can be generated from the water adduct via treatment with Et_3_SiH in commercially supplied solvents using Schlenk line techniques, which obviates the requirement for specialized equipment and rigorously anhydrous solvents. Using this alternative protocol, the C(3)-alkylated indole **2** was formed in 75% yield on a 0.1 mmol scale, and in 66% yield upon scale-up to 1 mmol of indole **1** (Scheme 3).

**Scheme 3:**
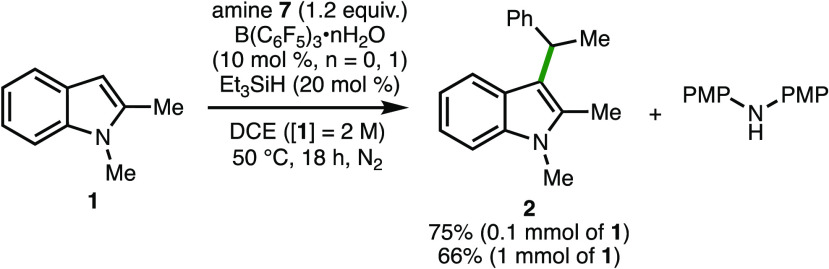
Alternative Protocol and Reaction Scale-up Yields as determined by ^1^H NMR analysis of the crude reaction mixture with 1,3,5-trimethylbenzene as the internal standard.

With the optimized reaction conditions in hand, the substrate scope of the secondary alkyl transfer process was investigated (Scheme 4). Initially, the impact of various substitutions on the aromatic ring within the benzylamine fragment upon conversion to products was studied. It was found that electron-releasing substituents (e.g., methyl and methoxy) were well tolerated at the 2-, 3-, and 4-positions on the aromatic ring, giving products **8**–**12** in high yields. Conversely, the strongly electron-withdrawing 4-CF_3_ group resulted in no observed product **13** formation, with starting materials recovered. Incorporation of an ethyl group at the benzylic position within the amine (R^4^ = Et) gave 51% conversion to product **14**. However, no conversion to C(3)-alkylated indole **15** was observed when a homobenzylic amine was employed, which highlighted the necessity of the benzylamine motif within the amine secondary alkyl group transfer reagent. The dihydroindenyl and tetrahydronaphthyl groups could be transferred to the C(3)-position of 1,2-dimethylindole to access products **16** and **17**, which were both formed in 59% and 61% yield, respectively. Within the indole fragment, a selection of substituents could be incorporated at the 5- and 6-positions to give products **18**–**22** in synthetically useful yields, including halides that enable facile subsequent product elaboration via established cross-coupling methodologies. Incorporation of a 5-NO_2_ group within the indole resulted in no observable conversion to **23**, which could be attributed to the reduced nucleophilicity of the indole. Both 1-methyl-2-phenylindole and 1-methylindole underwent efficient C(3)-alkylation to afford products **24** and **25** in 75% and 66% yields, respectively. Furthermore, it was found that 2-methylindole is a competent nucleophile in the secondary alkyl transfer process when used in combination with 2,2,6,6-tetramethylpiperidine (10 mol %) as a Brønsted base, which enabled good conversion to product **26**. Finally, the protocol was utilized to access an analogue of indomethacin, which is a nonsteroidal anti-inflammatory drug. The attenuated nucleophilicity of the *N*-benzoylated indole resulted in 26% conversion to indomethacin derivative **27**. It was found that 1,2,5-trimethylpyrrole was unreactive under the optimized reaction conditions.

**Scheme 4:**
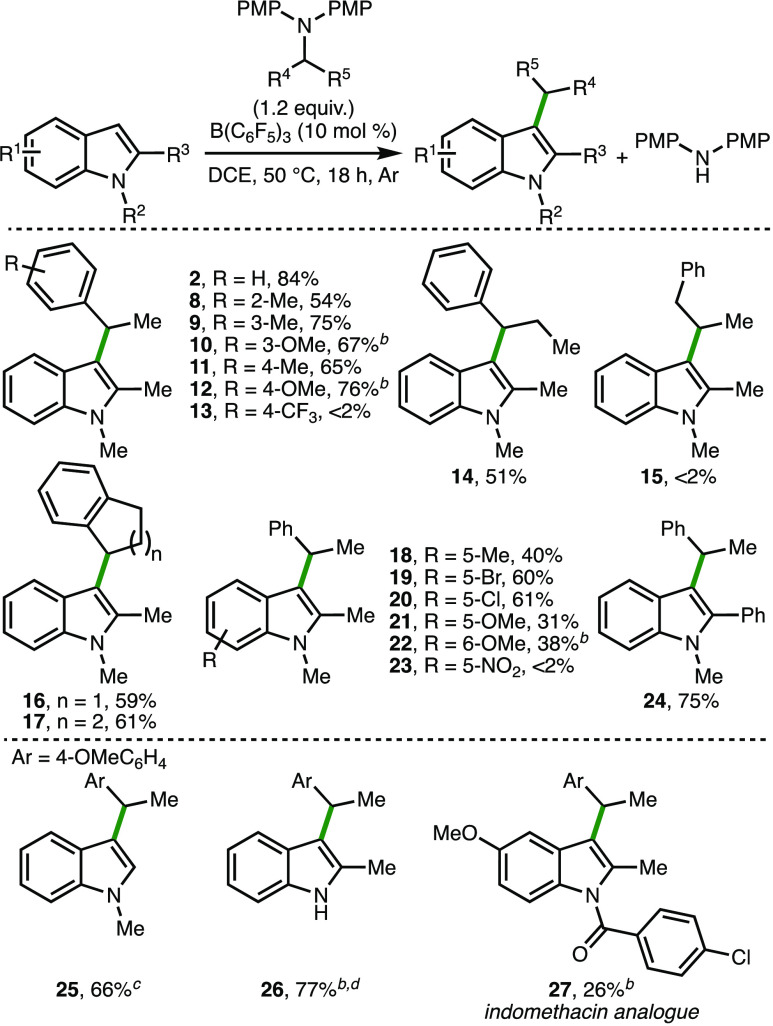
Substrate Scope Reactions performed with 0.1 mmol of substrate. [Substrate] = 2 M. Yields as determined by ^1^H NMR analysis of the crude reaction mixture with 1,3,5-trimethylbenzene as the internal standard. Twenty-four h. B(C_6_F_5_)_3_ (10 mol %) was prepared *in situ* from B(C_6_F_5_)_3_·*n*H_2_O (10 mol %, *n* = 0, 1) and Et_3_SiH (20 mol %) under N_2_. 2,2,6,6-Tetramethylpiperidine (10 mol %) added.

To gain insight into the reaction mechanism, experiments using deuterated substrates and reagents were performed (Scheme 5). Initially, employing C(3)-deuterated 1,2-dimethylindole **28** with the previously optimized reaction conditions (c.f., Table 1, entry 4), C(3)-alkylated indole **2** was formed in 61% yield without any deuterium incorporation within the product (Scheme 5A). In contrast, the B(C_6_F_5_)_3_-catalyzed C(3)-alkylation of 1,2-dimethylindole **1** with deuterated amine **29** gave product **30** with >98% D incorporation at the benzylic position (Scheme 5B). Based upon these results, and related processes described in the literature,^11^ a plausible reaction mechanism initiates with B(C_6_F_5_)_3_-mediated α-*N* C(sp^3^)–H hydride abstraction within the amine to give the corresponding iminium–borohydride ion pair (Scheme 5C). The iminium ion, which will be in equilibrium with the corresponding enamine (unproductive pathway), is intercepted by the indole, with subsequent amine elimination providing access to an α,β-unsaturated iminium ion. Hydride transfer from [HB(C_6_F_5_)_3_]^−^ to this iminium ion forms the observed C(3)-alkylated product, with regeneration of the borane catalyst.

**Scheme 5:**
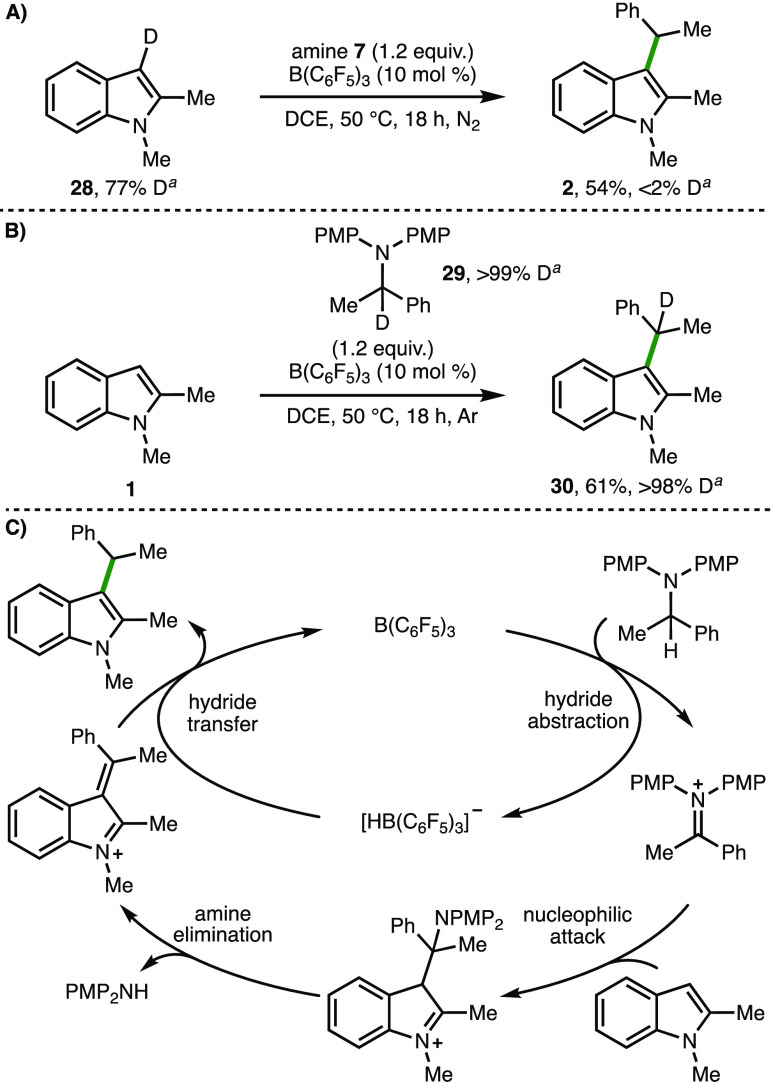
Reaction Mechanism Reactions performed with 0.1 mmol of substrate. [Substrate] = 2 M. Yields as determined by ^1^H NMR analysis of the crude reaction mixture with 1,3,5-trimethylbenzene as the internal standard.

In summary, we have developed a synthetic method to access a range of highly substituted indoles via the B(C_6_F_5_)_3_-catalyzed transfer of 2° alkyl groups from amine donors. Future work will focus on exploring alternative synthetic applications that are enabled by borane-mediated α-*N* C(sp^3^)–H hydride abstraction within amines, which will be reported in due course.

## Data Availability

The data underlying this study are openly available in the Cardiff University data catalogue at: 10.17035/d.2023.0296158061.

## Supporting Information Available

Optimization data, experimental procedures, characterization of new compounds and spectral data (PDF)
