# Supplementary material for: Accessing Highly Substituted Indoles via B(C6F5)3-Catalyzed Secondary Alkyl Group Transfer
Source: J Org Chem. 2024 Feb 23;89(6):4244–8. doi: 10.1021/acs.joc.4c00025 (PMC10949240; doi:10.1021/acs.joc.4c00025)
Supplement: Supplementary file 1 — jo4c00025_si_001.pdf [file jo4c00025_si_001.pdf]

SUPPORTING INFORMATION

Accessing Highly Substituted Indoles via $B(C_6F_5)_3$-Catalyzed Secondary Alkyl Group Transfer

Salma A. Elsherbeni ,[†,‡] Rebecca L. Melen ,[*,§] Alexander P. Pulis ,[*,∥] and Louis C. Morrill [*,†]

[†] Cardiff Catalysis Institute, School of Chemistry, Cardiff University , Main Building, Park Place, Cardiff, CF10 3AT, U.K.

[‡] Department of Pharmaceutical Chemistry, Faculty of Pharmacy, Tanta University , Tanta, Egypt

[§] Cardiff Catalysis Institute, School of Chemistry, Cardiff University, Translational Research Hub , Maindy Road, Cathays, Cardiff, CF24 4HQ, U.K.

[∥] School of Chemistry, University of Leicester , Leicester, LE1 7RH, U.K.

[*]E−mail: melenr@cardiff.ac.uk

[*]E−mail: a.pulis@leicester.ac.uk

[*]E−mail: MorrillLC@cardiff.ac.uk

Table of Contents

1. General information.. 2
2. Experimental and characterization data.. 3
    2.1. Synthesis of alcohols.. 3
        2.1.1. General procedure 1.. 3
    2.2. Synthesis of alkylating agents.. 14
        2.2.1. General procedure 2.. 17
        2.2.2. General procedure 3.. 20
    2.3. Synthesis of indole substrates.. 40
        2.3.1. General procedure 4.. 40
    2.4. Synthesis of deuterated substrates.. 52
    2.5. Optimization studies.. 57
    2.6. Substrate scope in the $B(C_6F_5)_3$-catalyzed alkylation.. 58
        2.6.1. General procedure 5.. 58
    2.7. The use of $H_2O{\cdot}B(C_6F_5)_3$ in the borane-catalyzed alkylation of indoles 83
        2.7.1. General procedure 6.. 83
    2.8. Scale up experiment.. 87
    2.9. Mechanistic studies.. 88
3. References.. 92

1. General information

Unless stated otherwise, all reactions were performed using oven-dried 10 mL microwave vials sealed with an aluminium crimped cap and were stirred with Teflon-coated magnetic stirrer bars. Dry solvents were obtained after passing these previously degassed solvents through activated alumina columns (Mbraun, SPS-800). Dry 1,2-dichloroethane (DCE) was obtained commercially from Alfa aesar, degassed and stored in an ampoule fitted with a Teflon valve under nitrogen atmosphere to use in the glove box. All other solvents and commercial reagents were used as supplied without further purification unless stated otherwise.

Unless stated otherwise, an argon-filled glove box (MBraun) was used to manipulate reagents including the storage of starting materials, catalysts and preparation of reactions.

Room temperature (rt) refers to 20−25 °C. Temperatures of 0 °C was obtained using ice/water. All reactions involving heating were carried out using DrySyn blocks and a contact thermometer. In vacuo refers to the use of a rotary evaporator under reduced pressure.

Analytical thin layer chromatography was carried out using aluminium plates coated with silica (Kieselgel 60 F254 silica) and visualization was achieved using ultraviolet light (254 nm), followed by staining with 1% aqueous $KMnO_4$ solution. Preparative TLC Flash chromatography used Kieselgel 60 silica in the solvent system stated.

Melting points were recorded on a Gallenkamp melting point apparatus, and corrected by linear interpolation of melting point standards benzophenone (47–49 ˚C), and benzoic acid (121–123 ˚C).

Infrared spectra were recorded on a Shimadzu IRAffinity-1 Fourier Transform ATIR spectrometer as thin films using a Pike MIRacle ATR accessory. Characteristic peaks are quoted (νmax / cm-1).

^{1}H, $^{13}C\{^{1}H\}$, $^{19}F\{^{1}H\}$ NMR spectra were obtained on either a Bruker Avance 300 (300 MHz ^{1}H, 75 MHz $^{13}C\{^{1}H\}$) or a Bruker Avance 400 (400 MHz ^{1}H, 101 MHz $^{13}C\{^{1}H\}$, 376 MHz ^{19}F) or a Bruker Avance 500 (500 MHz ^{1}H, 126 MHz $^{13}C\{^{1}H\}$, 471 MHz ^{19}F) spectrometer at rt in the solvent stated. Chemical shifts are reported in parts per million (ppm) relative to the residual solvent signal. All coupling constants, *J*, are quoted in Hz. Multiplicities are reported with the following symbols: s = singlet, d = doublet, t = triplet, q = quartet, hept = heptet, m = multiplet and multiples thereof. The abbreviation br to denote broad.

High resolution mass spectrometry (HRMS, m/z) data was acquired at Cardiff University on a Micromass LCT spectrometer. The NMR yields were determined by integration of suitable baseline separated ^{1}H NMR signals.

Tris(pentafluorophenyl)borane ($B(C_6F_5)_3$) was bought from Acros Organics and sublimed at 120 °C under reduced pressure (high vacuum) before use in the glove box. Tris(pentafluorophenyl)borane ($B(C_6F_5)_3$), used in the in situ drying procedure was purchased from Alfa aesar.

2. Experimental and characterization data

2.1. Synthesis of the alcohols

2.1.1. General procedure 1: 1-phenylpropan-1-ol

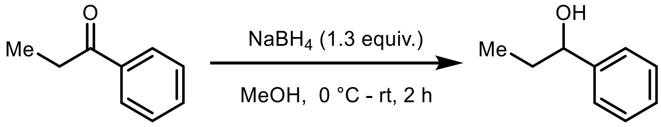

The title compound was prepared according to literature procedure.[1] 1-phenyl-1-propanone (1 g, 7.5 mmol) was dissolved in methanol (7.5 mL) and the solution was cooled to 0 °C. Sodium borohydride was then added portion wise over a period of 20 min. The mixture was warmed to room temperature and stirred for 2 h. After completion, methanol was removed under vacuum and the residue was dissolved in water (2.5 mL), extracted with DCM (2 x 2.5 mL) and washed with brine (2.5 mL), dried over $MgSO_4$, filtered, and concentrated in vacuo to give the title compound as a colourless oil (1 g, 100%); R_f = 0.34 (eluent = 15% EtOAc in petroleum ether); **^{1}H NMR (500 MHz, Chloroform-*d*)** δ 7.35 (d, *J* = 5.1 Hz, 4H), 7.31 – 7.25 (m, 1H), 4.60 (t, *J* = 6.6 Hz, 1H), 1.86 (br s, 1H), 1.85 – 1.70 (m, 2H), 0.92 (t, *J* = 7.4 Hz, 3H); **^{13}C{^{1}H} NMR (126 MHz, Chloroform-*d*)** δ 144.7, 128.5, 127.6, 126.1, 76.2, 32.0, 10.3.

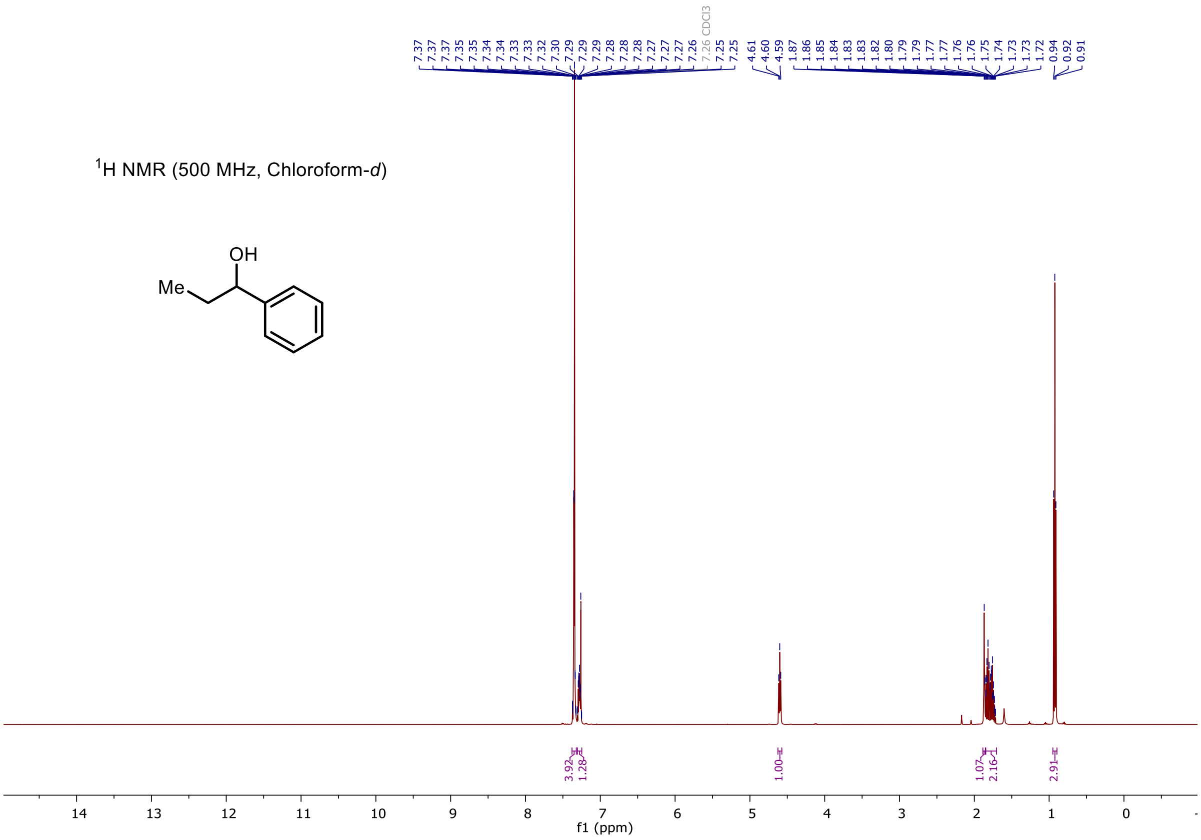

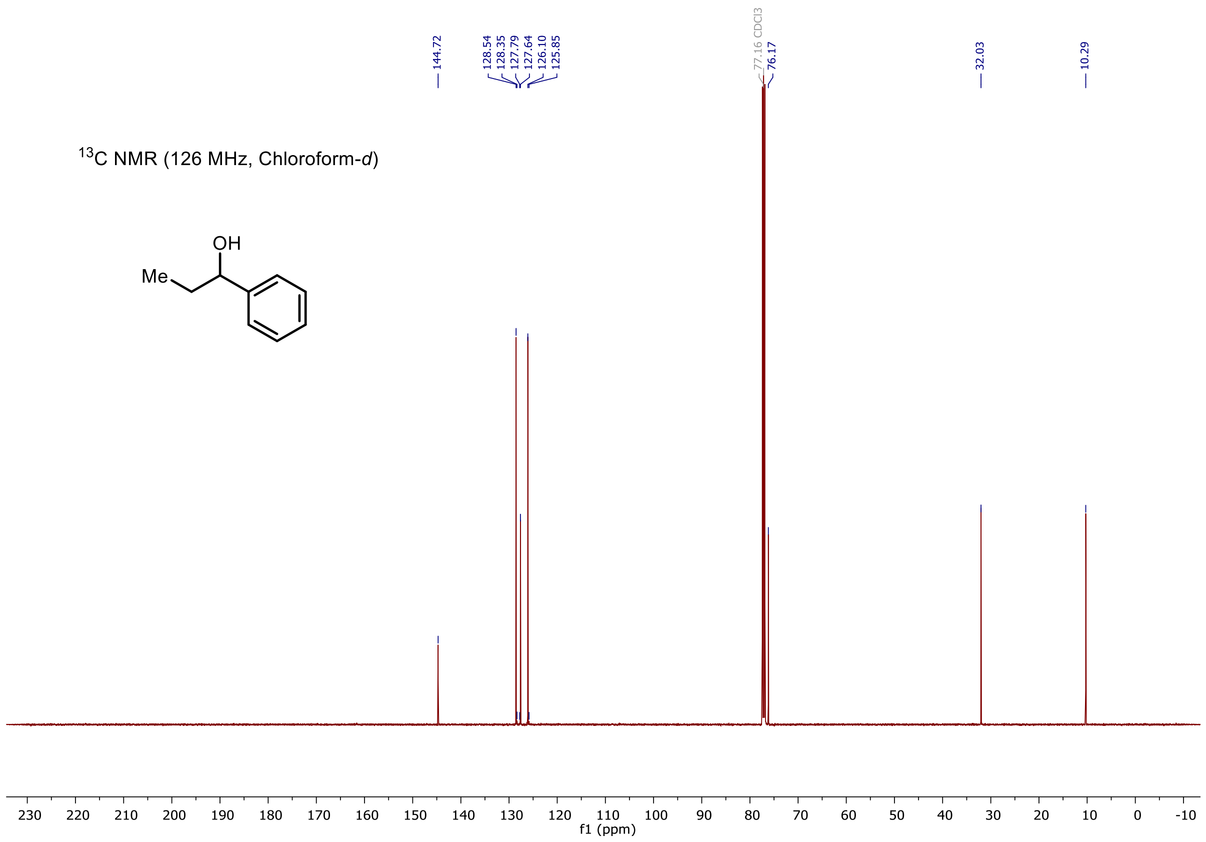

1-(o-tolyl)ethan-1-ol

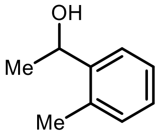

The title compound was prepared according to general procedure 1 using 2'-methylacetophenone (1 mL, 7.5 mmol) and obtained as a yellow oil (660 mg, 64%); R_f = 0.32 (eluent = 15% EtOAc in petroleum ether); **^{1}H NMR (500 MHz, Chloroform-*d*)** δ 7.52 (dd, *J* = 7.7, 1.4 Hz, 1H), 7.26 – 7.22 (m, 1H), 7.20 – 7.11 (m, 2H), 5.13 (q, *J* = 6.4 Hz, 1H), 2.35 (s, 3H), 1.83 (br s, 1H), 1.47 (d, *J* = 6.5 Hz, 3H); **^{13}C{^{1}H} NMR (126 MHz, Chloroform-*d*)** δ 144.0, 134.3, 130.5, 127.3, 126.5, 124.6, 66.9, 24.1, 19.0.

^{1}H NMR (500 MHz, Chloroform-*d*)

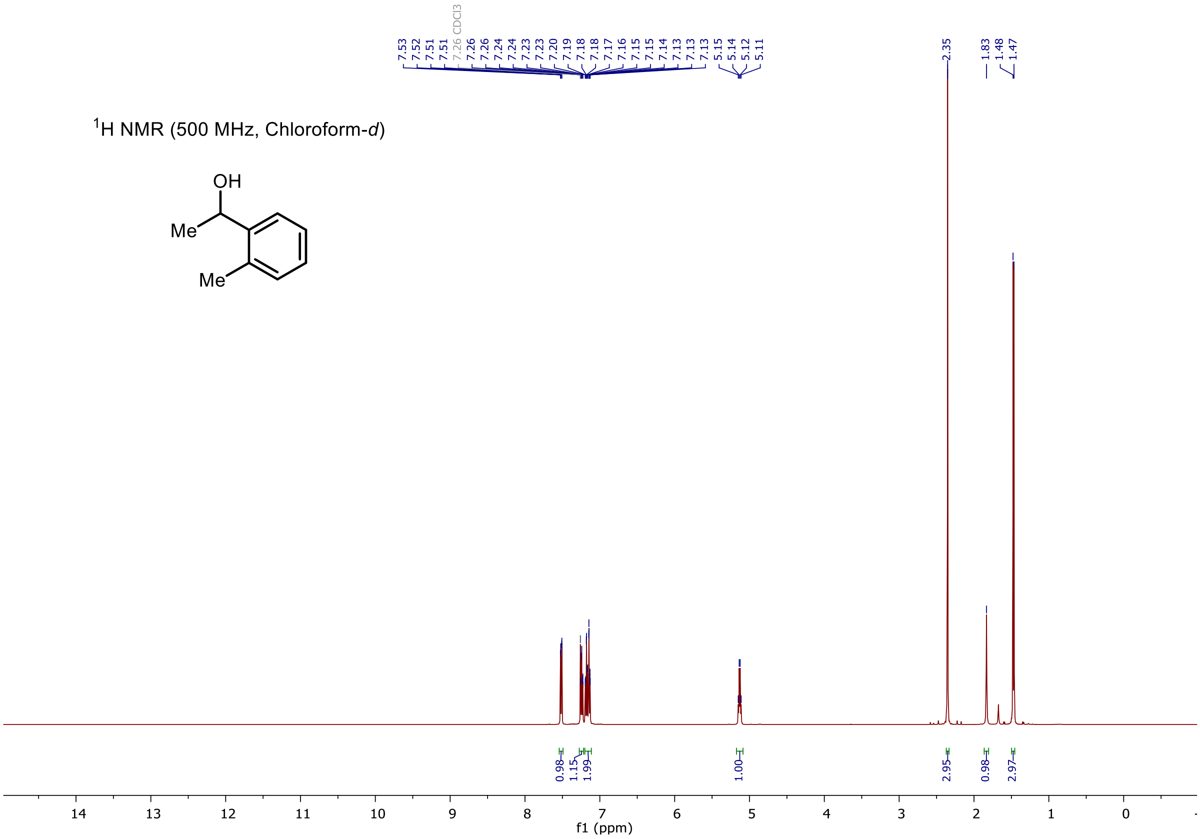

^{13}C NMR (126 MHz, Chloroform-*d*)

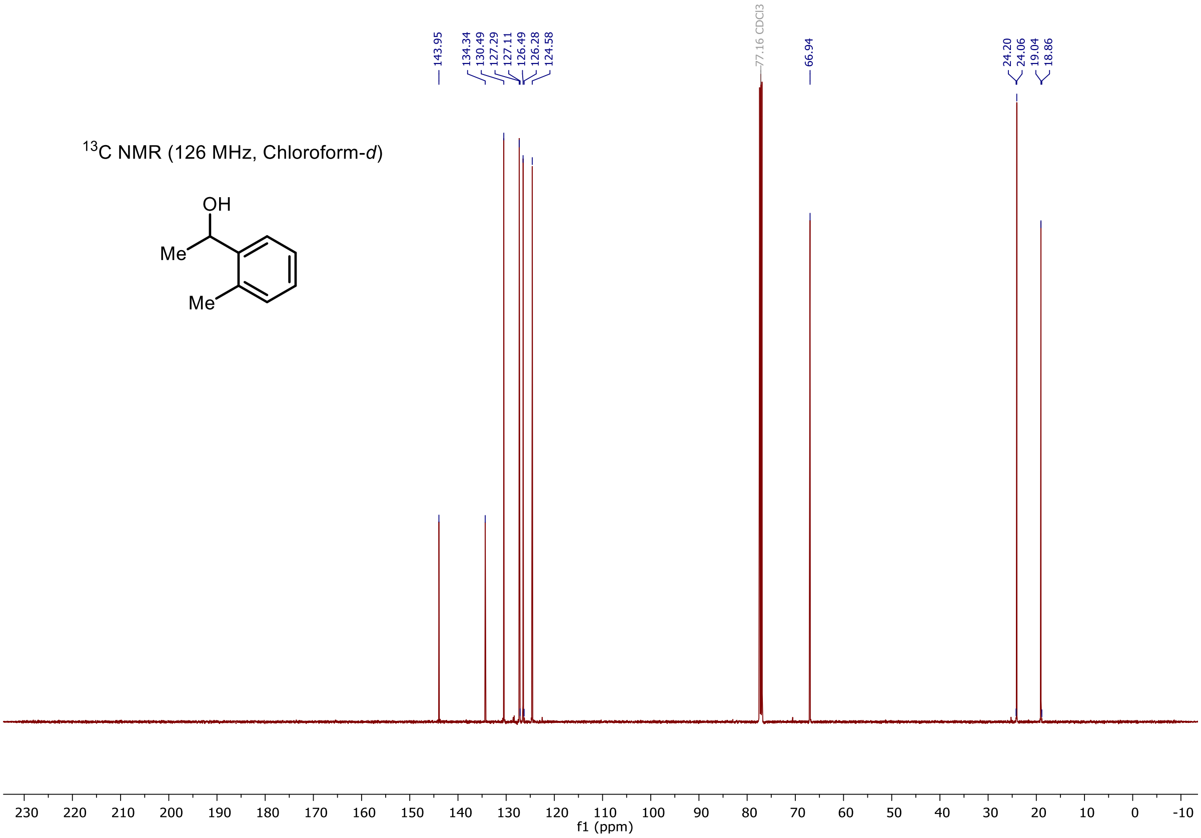

1-(m-tolyl)ethan-1-ol

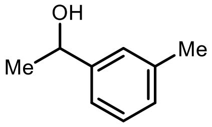

The title compound was prepared according to general procedure 1 using 3'-methylacetophenone (1 mL, 7.5 mmol) and obtained as a colourless oil (670 mg, 66%); R_f = 0.31 (eluent = 15% EtOAc in petroleum ether); **^{1}H NMR (500 MHz, Chloroform-*d*)** δ 7.27 – 7.23 (m, 1H), 7.21 – 7.14 (m, 2H), 7.10 (ddt, *J* = 7.6, 2.0, 0.9 Hz, 1H), 4.87 (q, *J* = 6.5 Hz, 1H), 2.37 (d, *J* = 0.8 Hz, 3H), 1.90 – 1.82 (m, 1H), 1.49 (d, *J* = 6.5 Hz, 3H); **^{13}C{^{1}H} NMR (126 MHz, Chloroform-*d*)** δ 145.9, 138.3, 128.5, 128.3, 126.2, 122.6, 70.6, 25.3, 21.6.

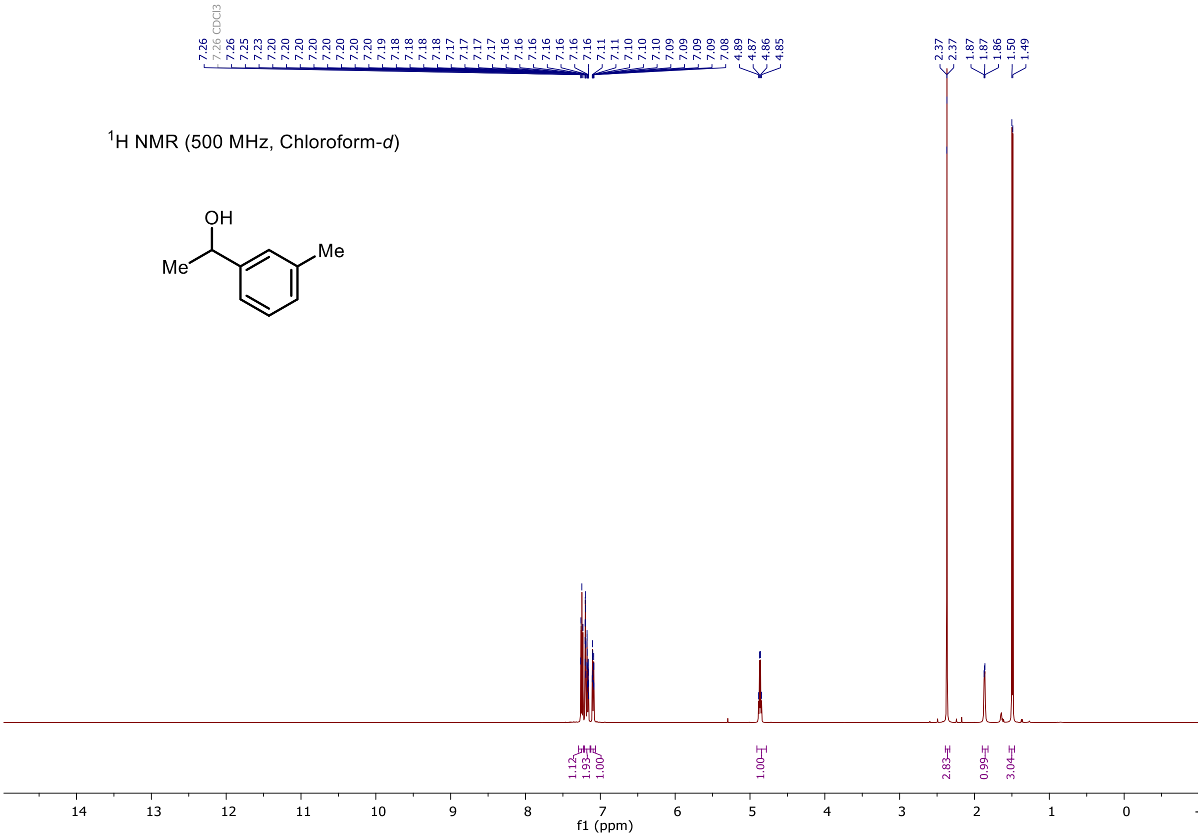

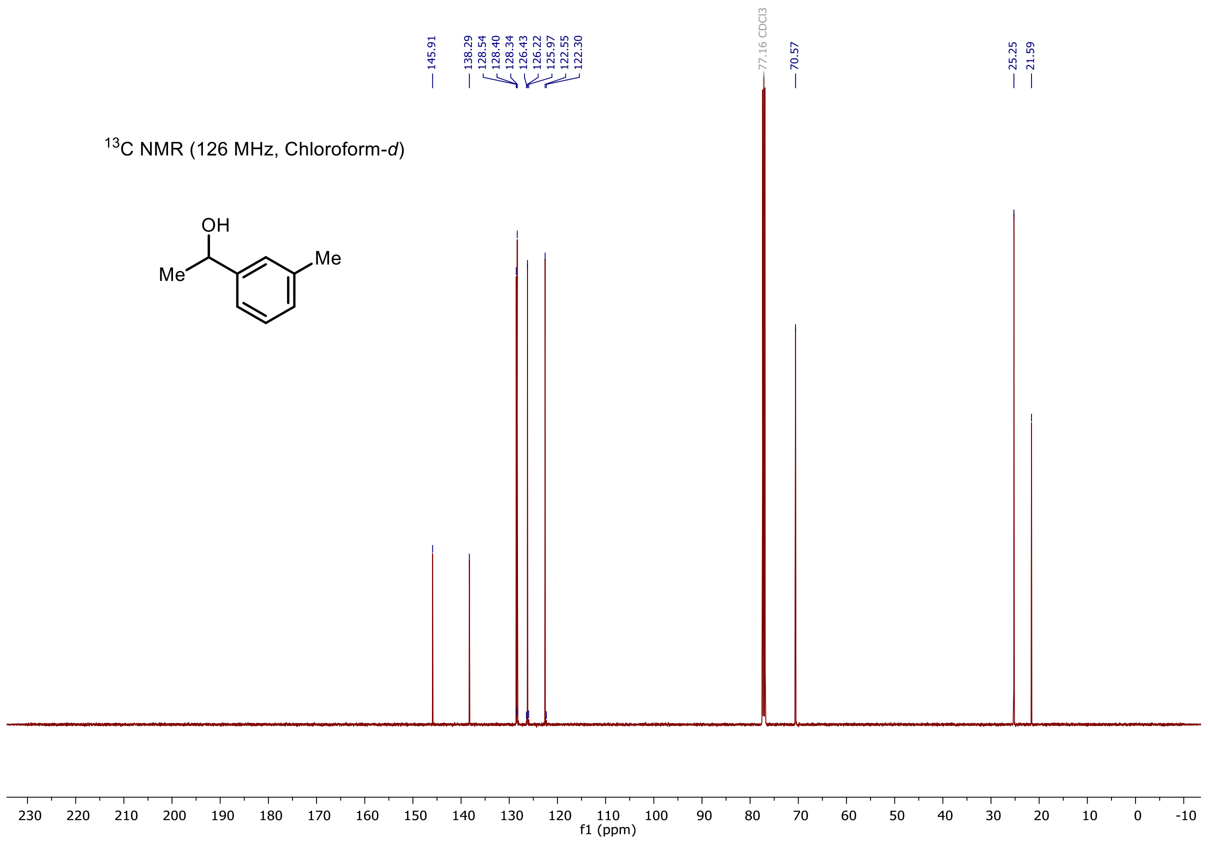

1-(p-tolyl)ethan-1-ol

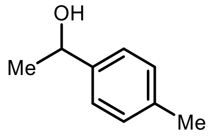

The title compound was prepared according to general procedure 1 using 4'-methylacetophenone (2 mL, 7.5 mmol) and obtained as a yellow oil (929 mg, 46%); R_f = 0.24 (eluent = 20% EtOAc in petroleum ether); **^{1}H NMR (500 MHz, Chloroform-*d*)** δ 7.27 (dd, *J* = 7.8, 1.6 Hz, 2H), 7.19 – 7.13 (m, 2H), 4.86 (q, *J* = 6.5 Hz, 1H), 2.35 (s, 3H), 1.88 (br s, 1H), 1.49 (d, *J* = 6.5 Hz, 3H); **^{13}C{^{1}H} NMR (126 MHz, Chloroform-*d*)** δ 143.0, 137.3, 129.3, 125.5, 70.4, 25.2, 21.2.

^{1}H NMR (500 MHz, Chloroform-*d*)

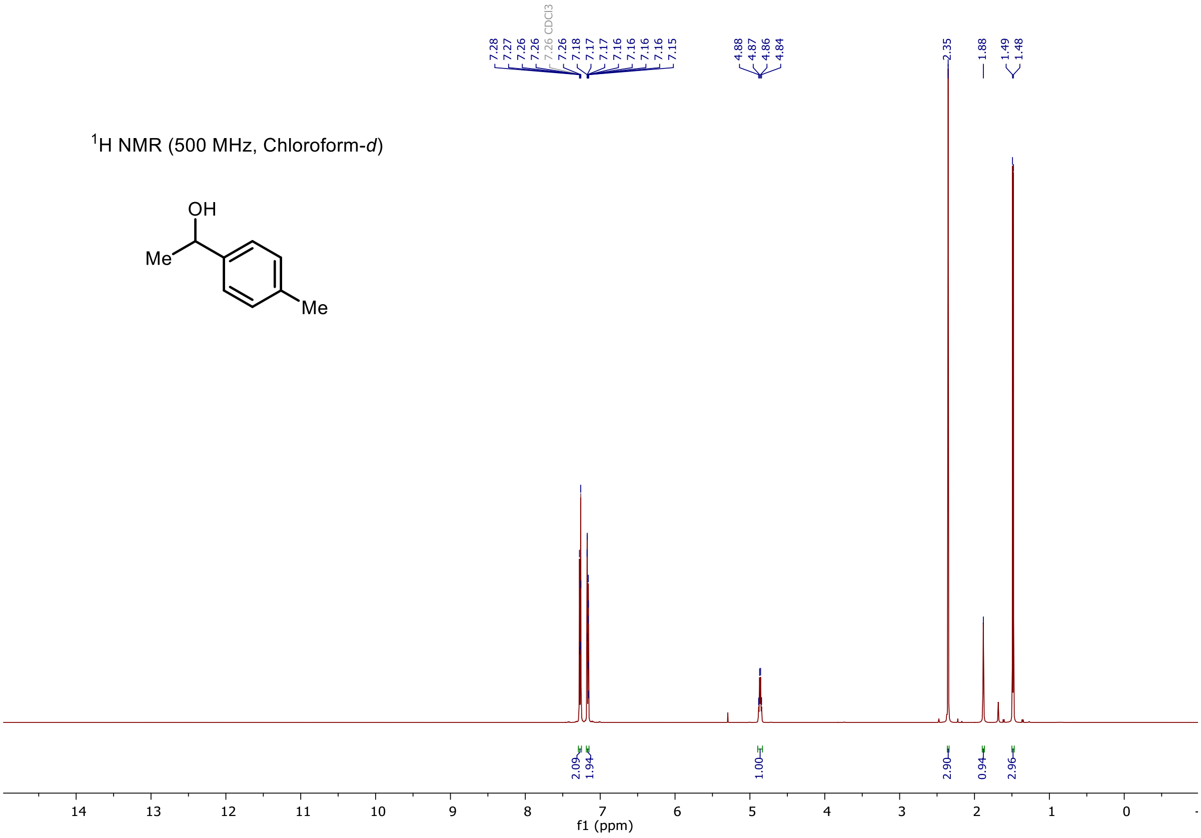

^{13}C NMR (126 MHz, Chloroform-*d*)

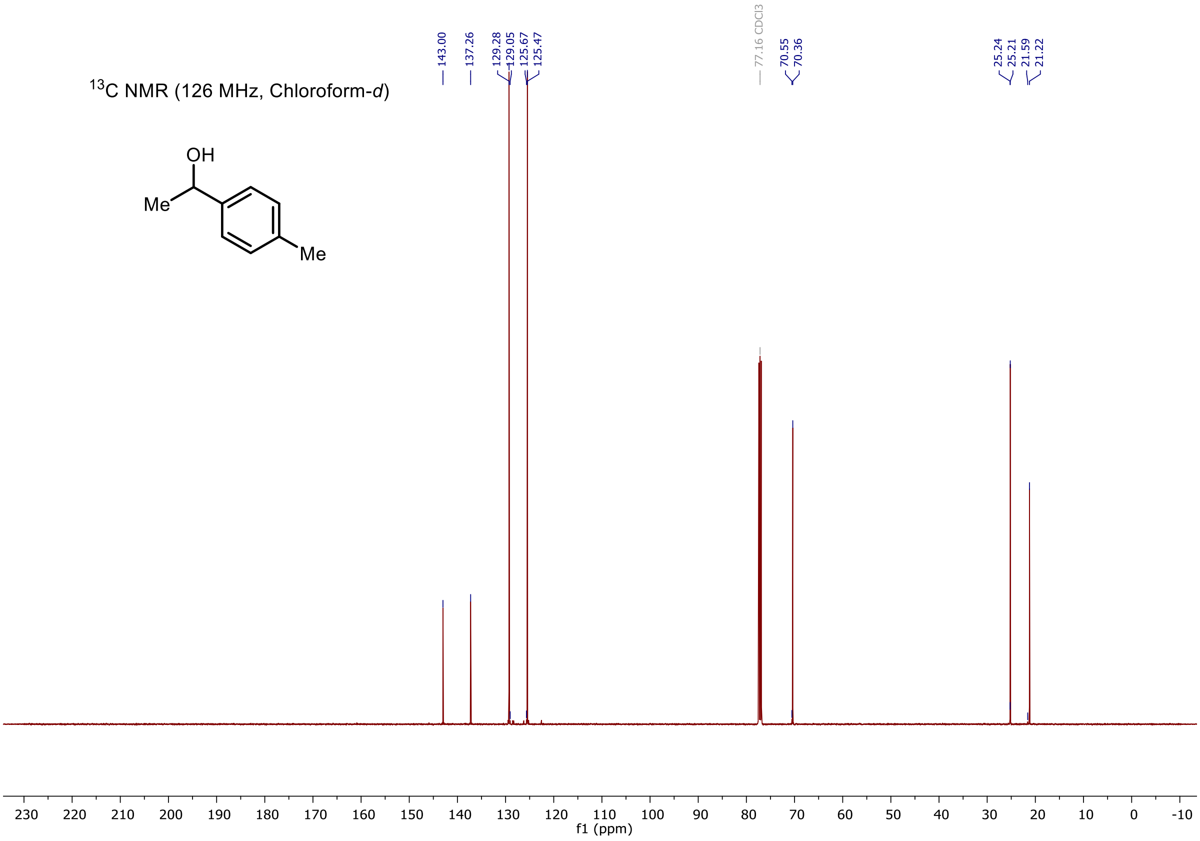

1-(3-methoxyphenyl)ethan-1-ol

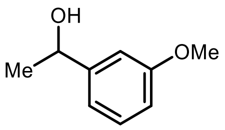

The title compound was prepared according to general procedure 1 using 3'-methoxyacetophenone (1.8 mL, 13.3 mmol) and obtained as a colourless oil (1.9 g, 99%); R_f = 0.38 (eluent = 20% EtOAc in petroleum ether); **^{1}H NMR (500 MHz, Chloroform-*d*)** δ 7.32 – 7.19 (m, 1H), 6.99 – 6.88 (m, 2H), 6.81 (ddd, *J* = 8.2, 2.5, 1.1 Hz, 1H), 4.87 (qd, *J* = 6.1, 3.3 Hz, 1H), 3.81 (s, 3H), 1.99 (d, *J* = 2.7 Hz, 1H), 1.48 (d, *J* = 6.5 Hz, 3H); **^{13}C{^{1}H} NMR (126 MHz, Chloroform-*d*)** δ 159.9, 147.7, 129.6, 117.8, 113.0, 111.0, 70.4, 55.3, 25.3.

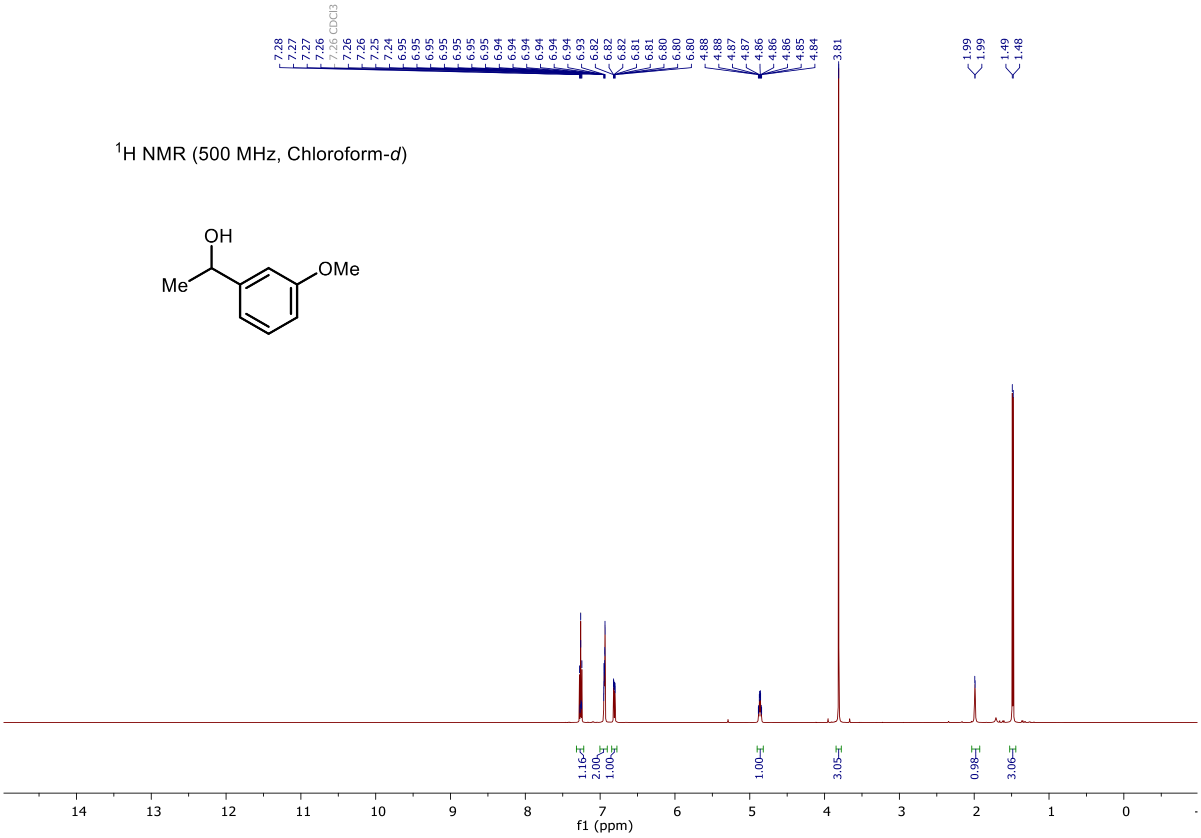

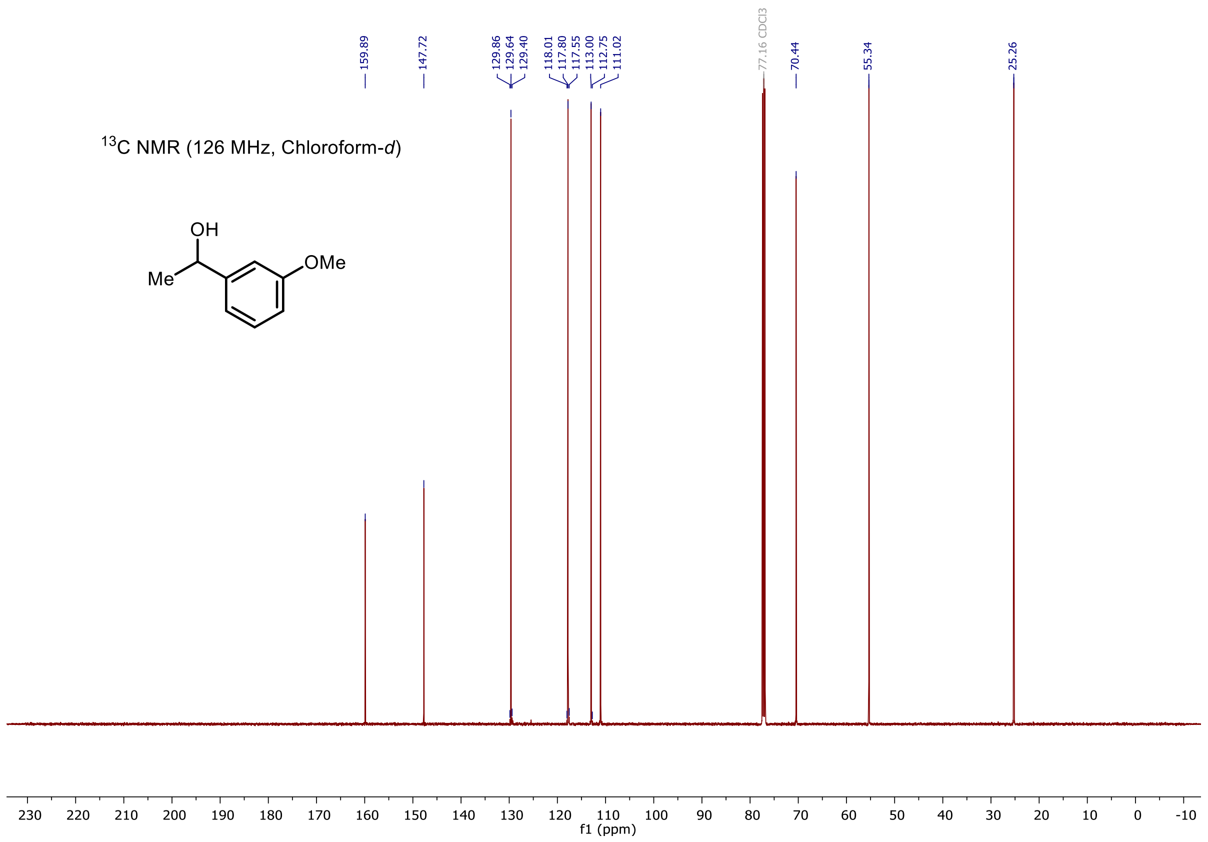

1-(4-methoxyphenyl)ethan-1-ol

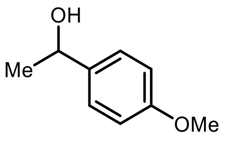

The title compound was prepared according to general procedure 1 using 4'-methoxyacetophenone (2 g, 13.3 mmol) and obtained as a colourless oil (1.8 g, 90%); R_f = 0.5 (eluent = 30% EtOAc in petroleum ether); **^{1}H NMR (500 MHz, Chloroform-*d*)** δ 7.33 – 7.28 (m, 2H), 6.96 – 6.81 (m, 2H), 4.85 (q, *J* = 6.4 Hz, 1H), 3.81 (d, *J* = 2.2 Hz, 2H), 1.85 (br s, 1H), 1.48 (dd, *J* = 6.5, 2.2 Hz, 3H); **^{13}C{^{1}H} NMR (126 MHz, Chloroform-*d*)** δ 159.1, 138.1, 126.8, 114.0, 70.1, 55.4, 25.2.

^{1}H NMR (500 MHz, Chloroform-*d*)

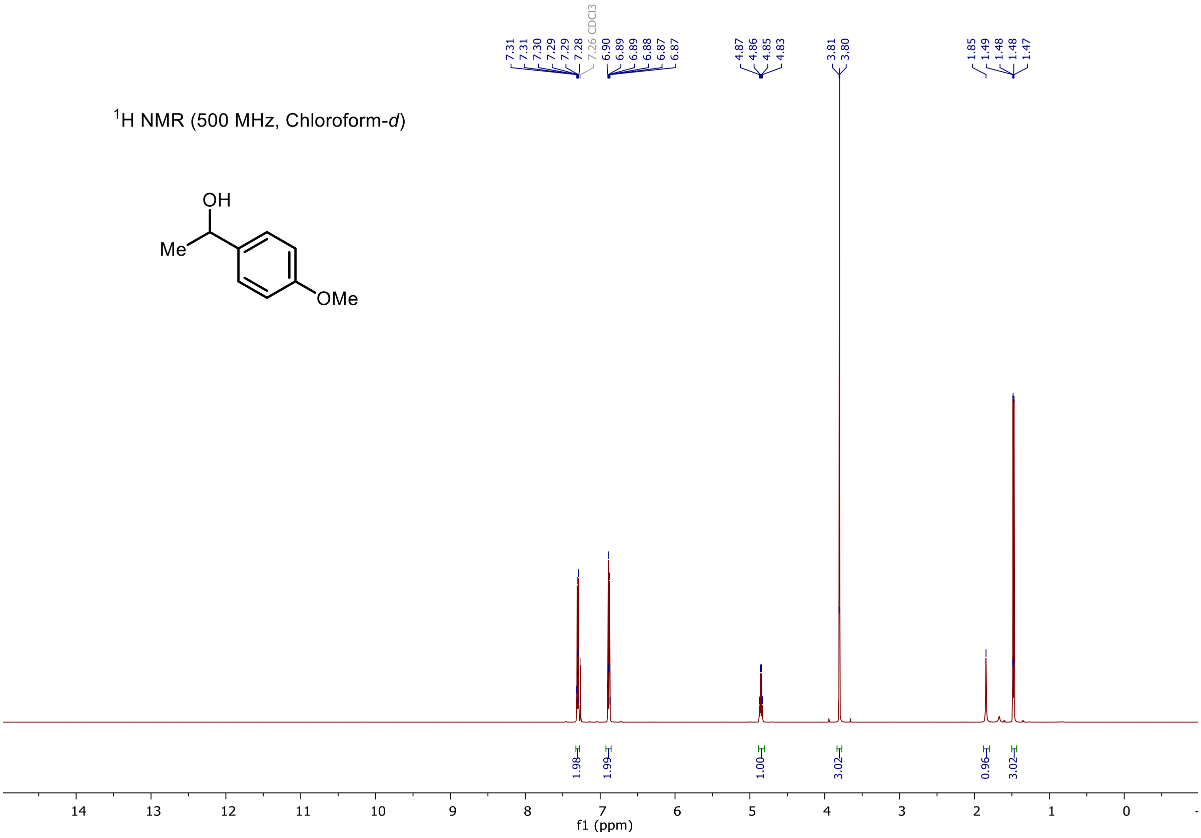

^{13}C NMR (126 MHz, Chloroform-*d*)

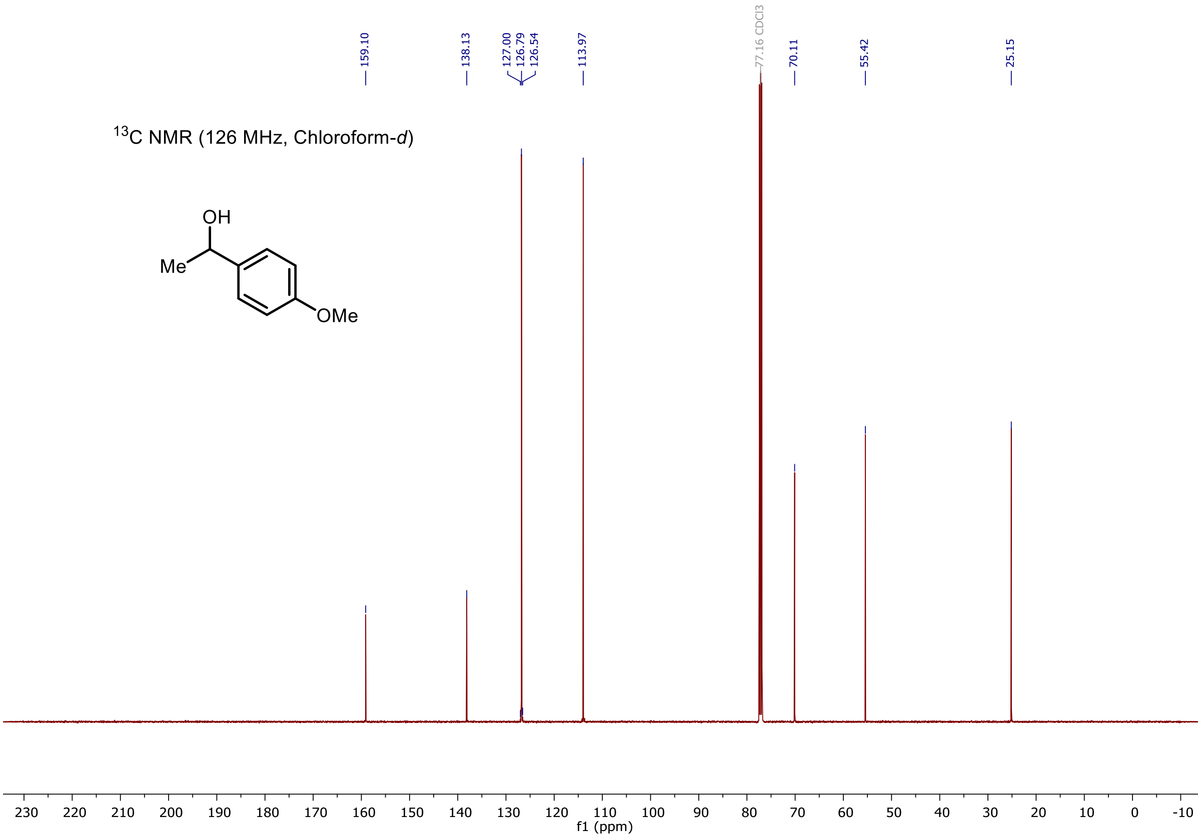

1-(4-(trifluoromethyl)phenyl)ethan-1-ol

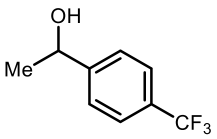

The title compound was prepared according to general procedure 1 using 4'-(trifluoromethyl)acetophenone (1 g, 5.3 mmol) and obtained as an amber oil (756 mg, 75%); R_f = 0.41 (eluent = 20% EtOAc in petroleum ether); **^{1}H NMR (500 MHz, Chloroform-*d*)** δ 7.65 – 7.56 (m, 2H), 7.50 – 7.44 (m, 2H), 4.95 (qd, *J* = 6.6, 2.8 Hz, 1H), 2.10 (d, *J* = 3.1 Hz, 1H), 1.50 (d, $J_{C\text{-}F}$ = 6.5 Hz, 3H); **^{13}C{^{1}H} NMR (126 MHz, Chloroform-*d*)** δ 149.8 (q, $J_{C\text{-}F}$ = 1.3 Hz), 129.8 (q, $J_{C\text{-}F}$ = 32.3 Hz), 127.5, 125.8, 125.6 (q, $J_{C\text{-}F}$ = 3.8 Hz), 125.4, 123.2, 121.1, 70.0, 25.5; **^{19}F NMR (471 MHz, Chloroform-*d*)** δ -62.47.

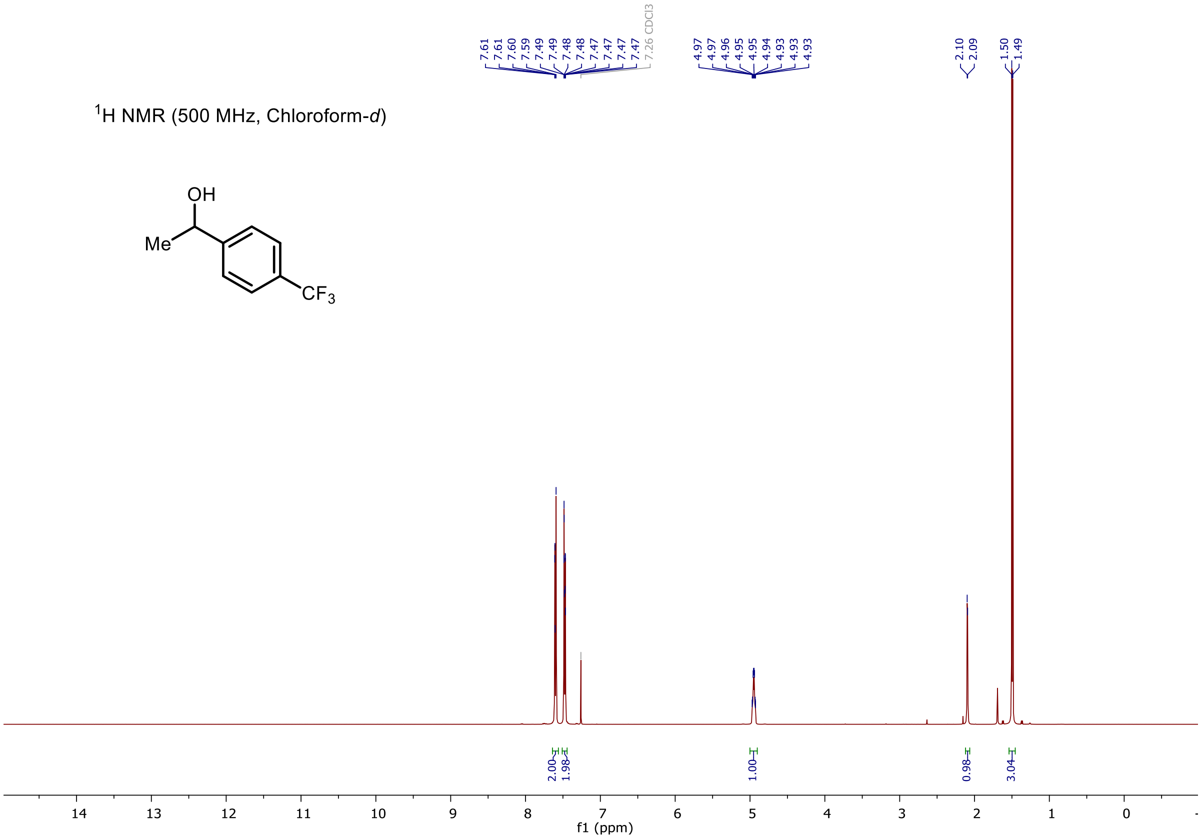

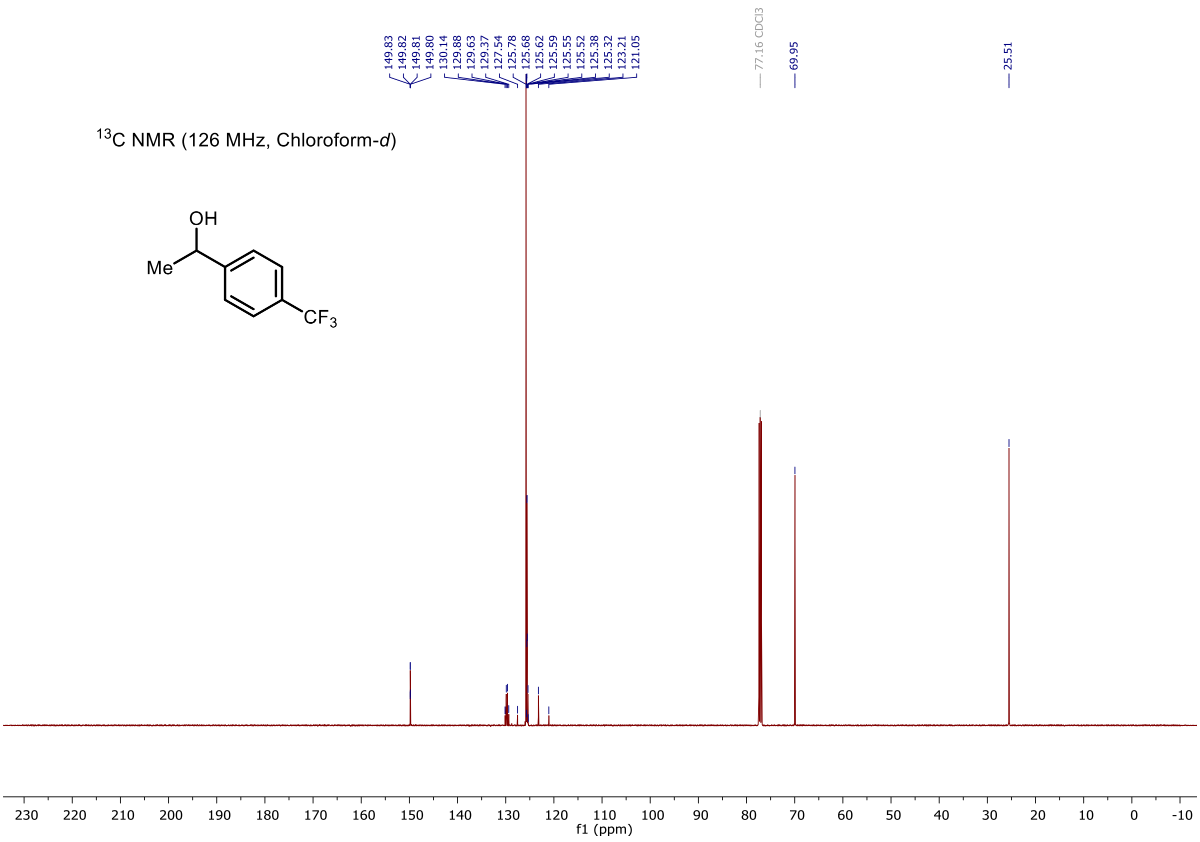

149.83
149.82
149.81
149.80
130.14
129.88
129.63
129.37
127.54
125.78
125.68
125.62
125.59
125.55
125.52
125.38
125.32
123.21
121.05
77.16 CDCl3
69.95
25.51
13C NMR (126 MHz, Chloroform-d)
OH
Me
CF3
230
220
210
200
190
180
170
160
150
140
130
120
110
100
90
80
70
60
50
40
30
20
10
0
-10
f1 (ppm)

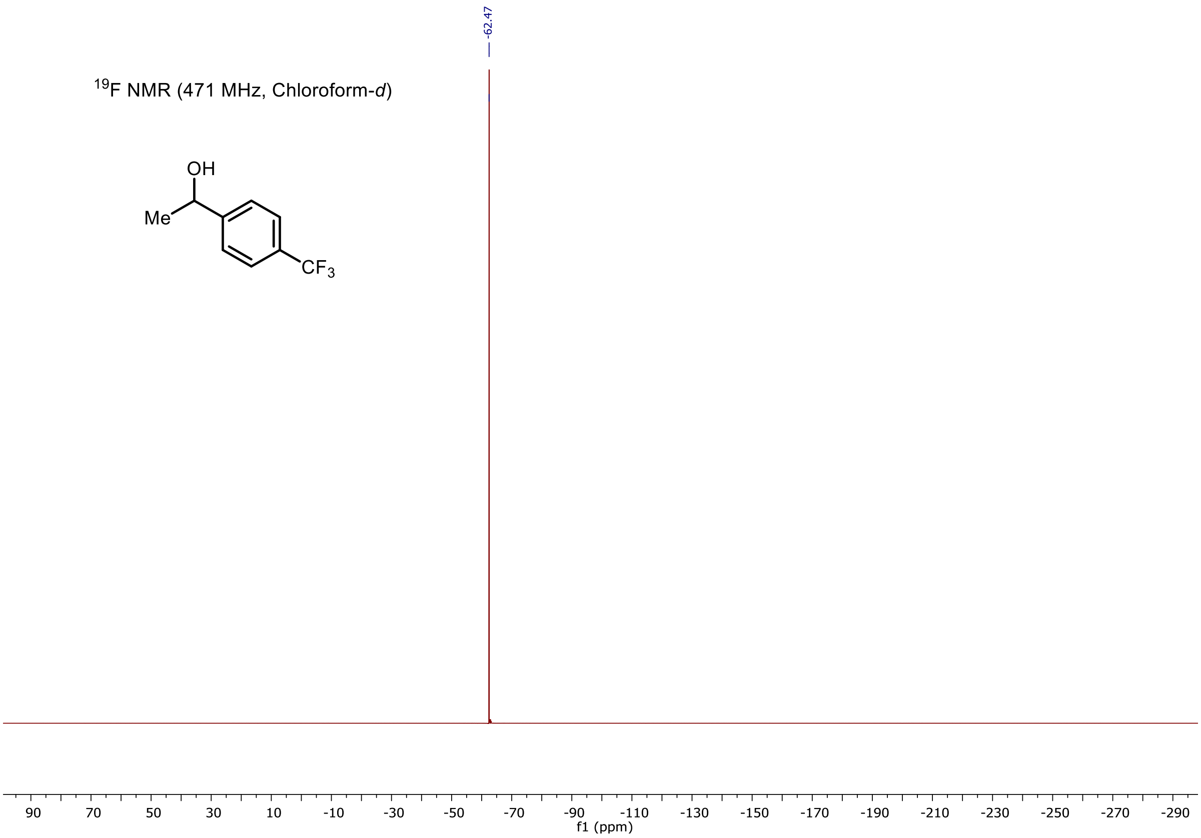

-62.47
19F NMR (471 MHz, Chloroform-d)
OH
Me
CF3
90
70
50
30
10
-10
-30
-50
-70
-90
-110
-130
-150
-170
-190
-210
-230
-250
-270
-290
f1 (ppm)

2.2. Synthesis of alkylating agents

Bis(4-methoxyphenyl)amine

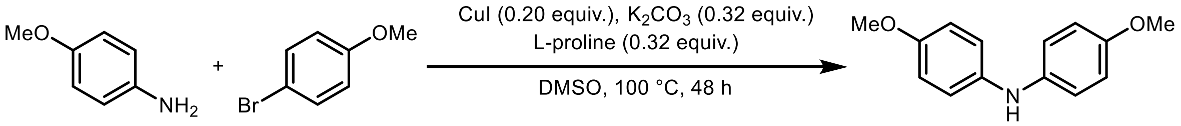

To a stirred solution of 4-aminoanisole (21.4 g, 174 mmol) and potassium carbonate (8.9 g, 64 mmol) in DMSO (250 mL), 4-bromoanisol (20 mL, 160 mmol), copper(I)iodide (7.6, 41 mmol) and L-proline (7.4 g, 64 mmol) were added and the mixture was stirred at 100 °C for 48 h. Then, it was allowed to cool, quenched with water (250 mL) and extracted with diethyl ether (3 x 200 mL). The organics were combined, washed with brine, dried over $MgSO_4$, filtered, and concentrated *in vacuo*. Purification by column chromatography on silica gel (eluent = 10% EtOAc in petroleum ether, 70 × 180 mm silica) gave the title compound as an off-white solid (13 g, 35%); mp 104–106 °C (Lit. 100–104 °C);[2] R_f = 0.49 (eluent = 20% EtOAc in petroleum ether); **^{1}H NMR (500 MHz, Chloroform-*d*)** δ 6.97 – 6.91 (m, 4H), 6.85 – 6.80 (m, 4H), 5.27 (s, 1H), 3.78 (s, 6H); **^{13}C{^{1}H} NMR (126 MHz, Chloroform-*d*)** δ 154.4, 138.1, 119.7, 114.9, 55.8.

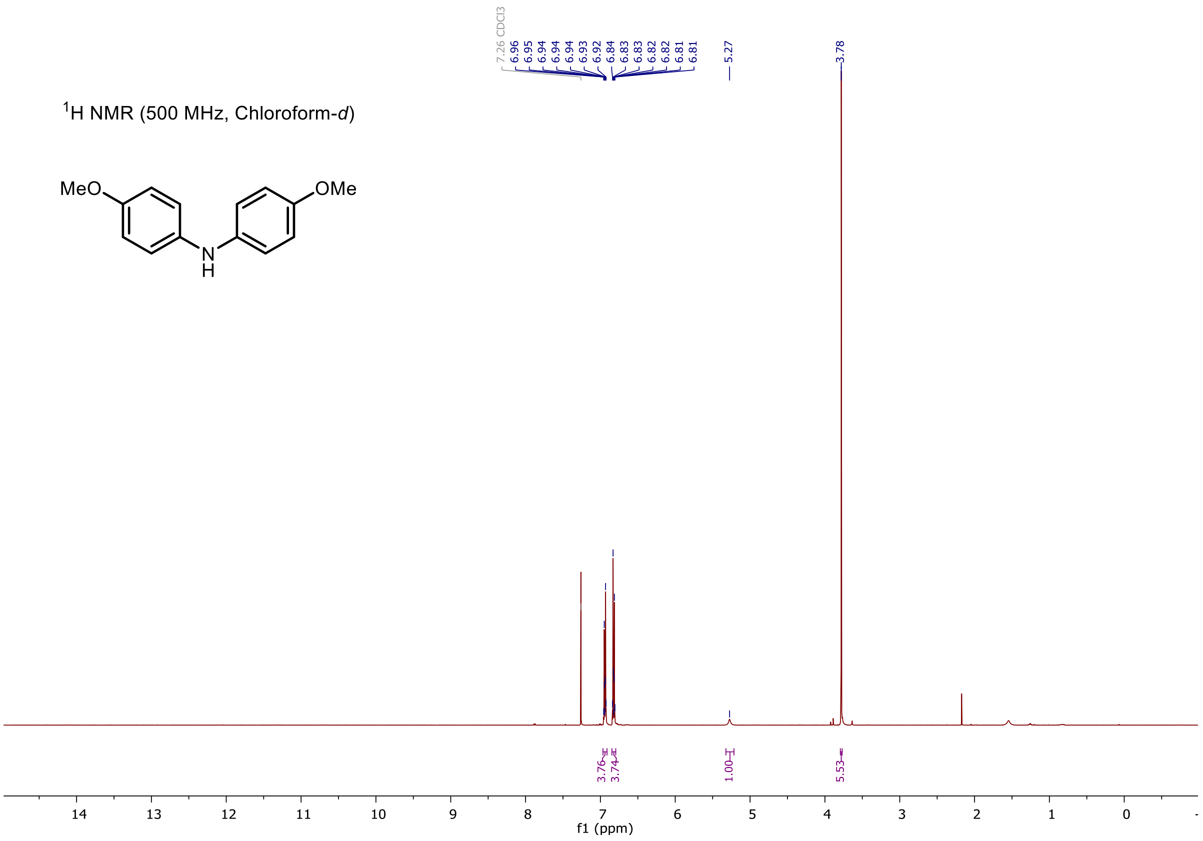

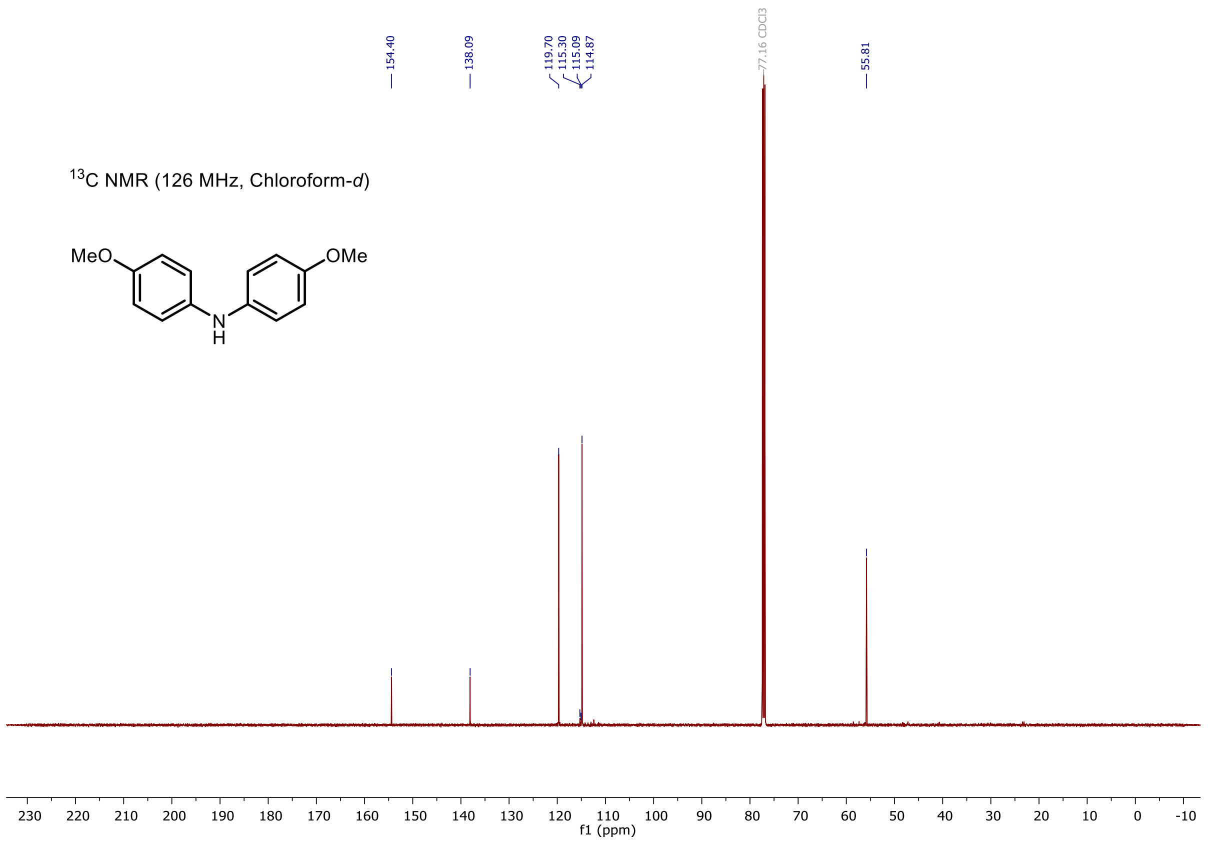

4-methoxy-2,6-dimethylaniline

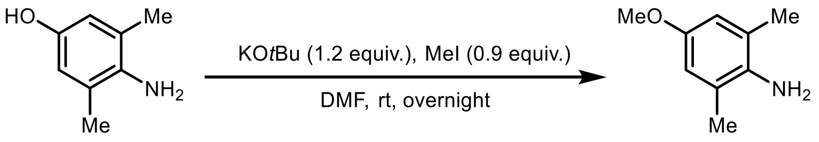

4-Amino-3,4-dimethylphenol (2 g, 14.6 mmol) and potassium tert-butoxide (1.96 g, 17.5 mmol) were dissolved in DMF (33 mL) under nitrogen. Then, a solution of methyl iodide (0.8 mL, 13.1 mmol) in DMF (6.7 mL) was added over a period of 6 h. After the addition was complete, the reaction mixture was stirred overnight at room temperature. DCM (100 mL) was added, and the combined organic layers were washed with KOH (1 M solution, 3 X 33 mL) and brine (7 mL), dried over $MgSO_4$, filtered, and concentrated *in vacuo*. Purification by column chromatography on silica gel (eluent = 45% EtOAc in hexanes, 70 × 180 mm silica) gave the title compound as a red oil (823 mg, 42%); R_f = 0.72 (eluent = 20% EtOAc in hexanes); **^{1}H NMR (500 MHz, Chloroform-*d*)** δ 6.56 (dd, *J* = 1.2, 0.6 Hz, 2H), 3.73 (s, 3H), 2.18 (q, *J* = 0.5 Hz, 6H); **^{13}C{^{1}H} NMR (126 MHz, Chloroform-*d*)** δ 152.2, 136.5, 123.3, 114.0, 55.9, 18.2. The spectroscopic data are in accordance with those described in the literature.[3]

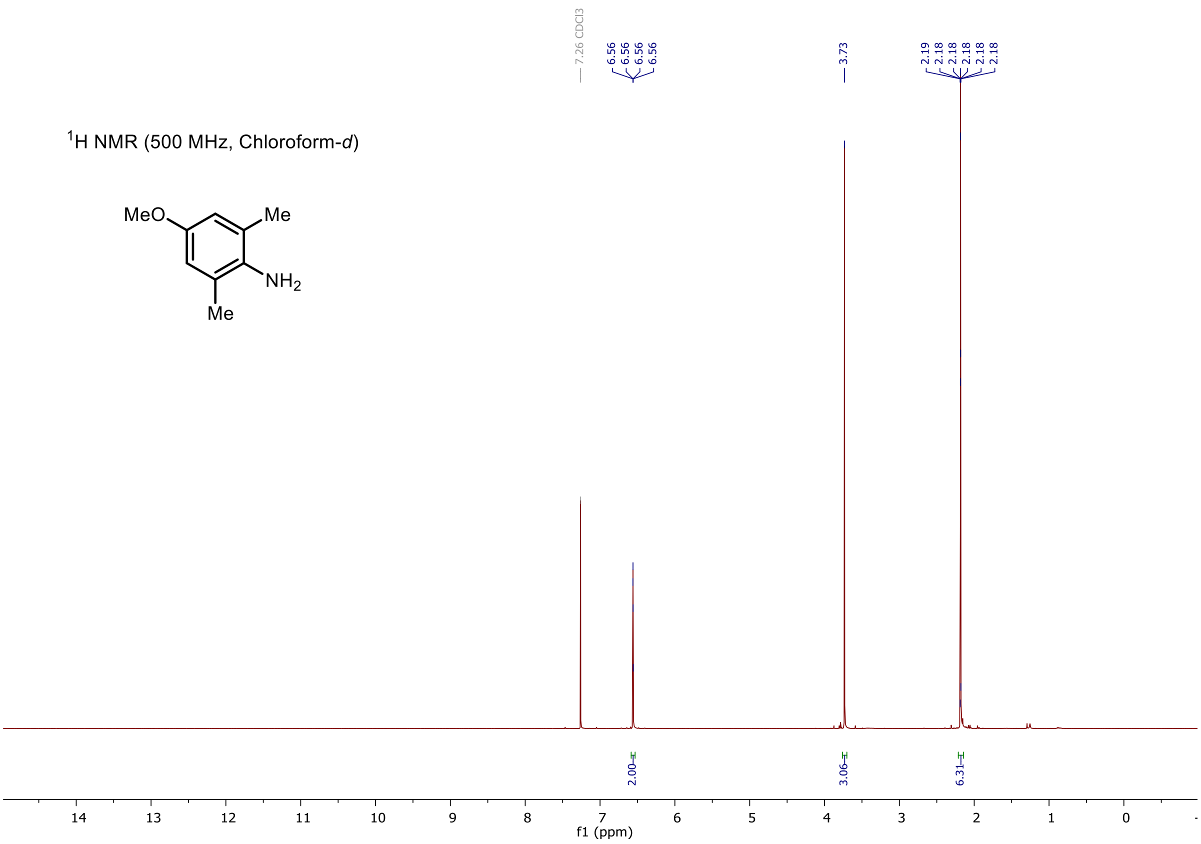

7.26 CDCl3
6.56
6.56
6.56
6.56
3.73
2.19
2.18
2.18
2.18
2.18
2.18
^{1}H NMR (500 MHz, Chloroform-d)
MeO
Me
NH2
Me
2.00
3.06
6.31
14
13
12
11
10
9
8
7
6
5
4
3
2
1
0
f1 (ppm)

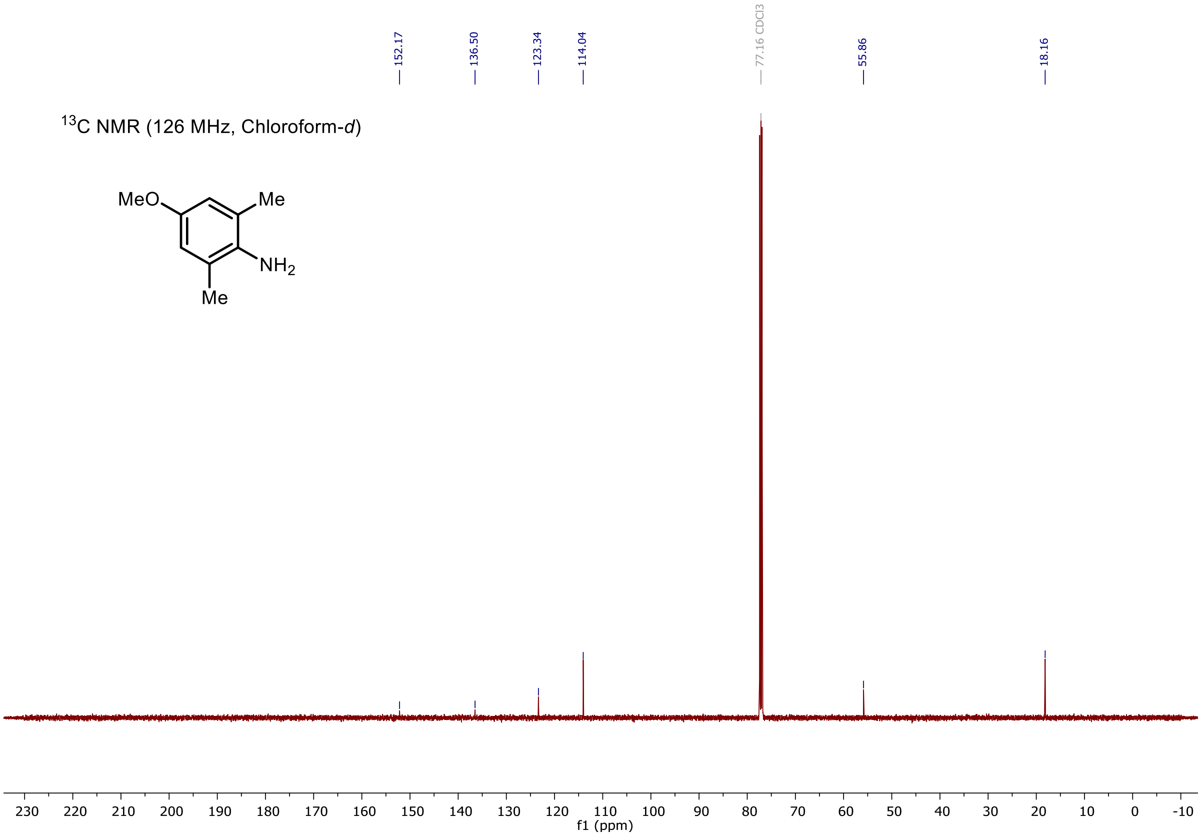

152.17
136.50
123.34
114.04
77.16 CDCl3
55.86
18.16
^{13}C NMR (126 MHz, Chloroform-d)
MeO
Me
NH2
Me
230
220
210
200
190
180
170
160
150
140
130
120
110
100
90
80
70
60
50
40
30
20
10
0
-10
f1 (ppm)

2.2.1. General procedure 2:

4-methoxy-N-(4-methoxyphenyl)-N-(1-phenylpropan-2-yl)aniline

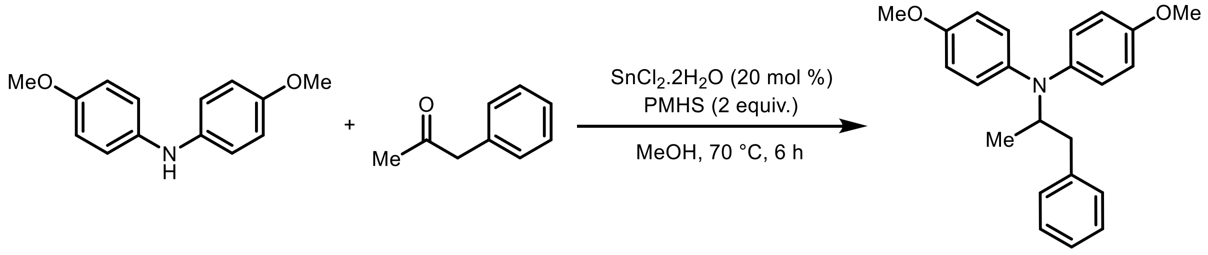

The title compound was prepared according to literature procedure.[4] To a stirred solution of tin(II)chloride dihydrate (366 mg, 1.6 mmol) in methanol (24 mL), bis(4-methoxyphenyl)amine (1 g, 4.4 mmol), phenylacetone (640 mg, 4.8 mmol) and polymethylhydrosiloxane (1 mL, 8.8 mmol) were added at room temperature. The reaction mixture was heated to 70 °C and stirred at the same temperature for 6 h. After completion of the reaction as indicated by TLC, the mixture was allowed to cool, filtered and concentrated *in vacuo*. Purification by column chromatography on silica gel (eluent = 15% EtOAc in petroleum ether, 40 × 180 mm silica) gave the title compound as a brown oil (1.24 g, 62%); R_f = 0.8 (eluent = 15% EtOAc in petroleum ether); vmax /cm-1 (film) 3029, 2931, 2902, 2833, 1500, 1460, 1440, 1381, 1238, 1178, 1035, 908, 815, 729; **^{1}H NMR (500 MHz, Chloroform-*d*)** δ 7.31 – 7.27 (m, 2H), 7.23 – 7.17 (m, 3H), 6.87 – 6.80 (m, 8H), 4.32 – 4.21 (m, 1H), 3.80 (s, 6H), 3.15 (dd, *J* = 13.4, 4.5 Hz, 1H), 2.47 (ddd, *J* = 13.6, 9.6, 4.5 Hz, 1H), 1.07 (d, *J* = 6.5 Hz, 3H); **^{13}C{^{1}H} NMR (126 MHz, Chloroform-*d*)** δ 154.8, 140.6, 140.0, 129.3, 128.5, 126.2, 124.0, 114.7, 55.8, 54.6, 41.6, 18.2; HRMS (ESI-TOF) calculated $[C_{23}H_{26}NO_2]^+$ $(M + H)^+$: m/z 348.1964, found 348.1964.

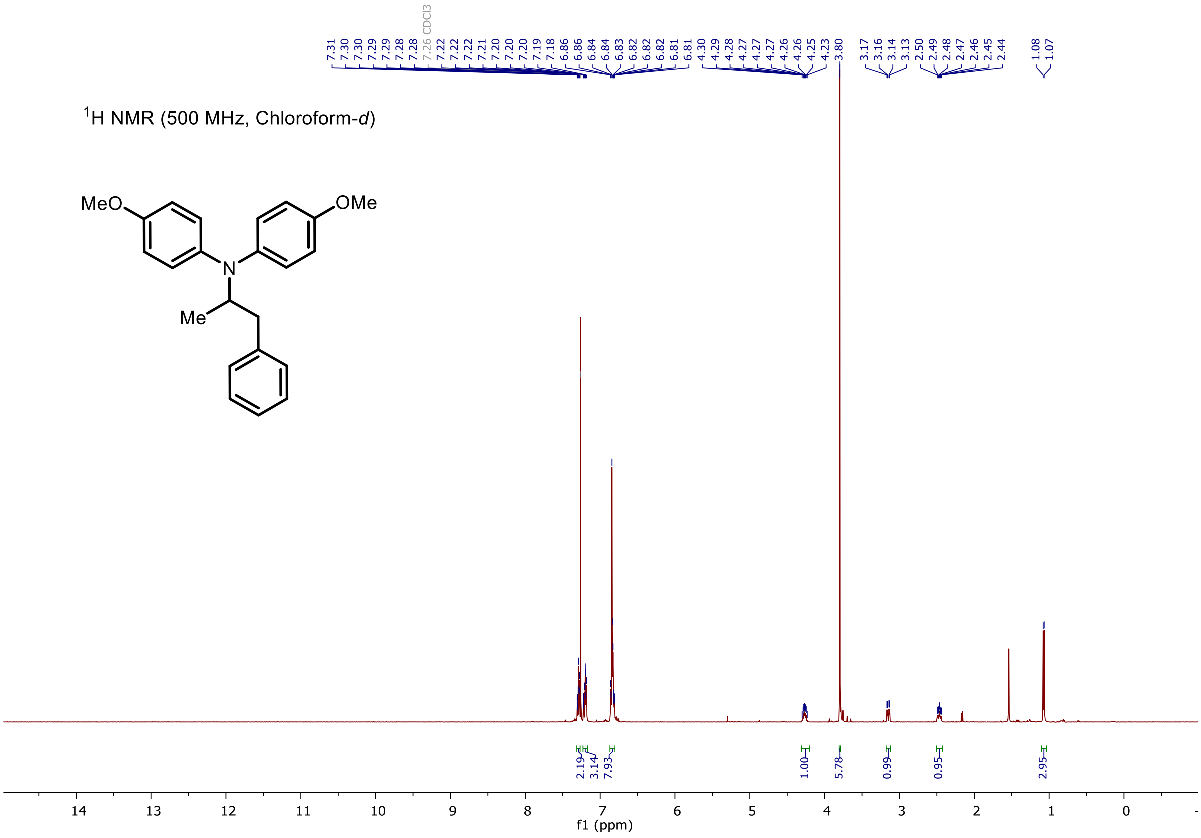

^{1}H NMR (500 MHz, Chloroform-d)
MeO
OMe
N
Me
7.31 7.30 7.30 7.29 7.29 7.28 7.28 7.26 CDCl3 7.22 7.22 7.22 7.22 7.21 7.20 7.20 7.20 7.19 7.18 6.86 6.86 6.84 6.84 6.83 6.82 6.82 6.82 6.81 6.81 4.30 4.29 4.28 4.27 4.27 4.26 4.26 4.25 4.23 3.80 3.17 3.16 3.14 3.13 2.50 2.49 2.48 2.47 2.46 2.45 2.44 1.08 1.07
2.19 3.14 7.93 1.00 5.78 0.99 0.95 2.95
f1 (ppm)

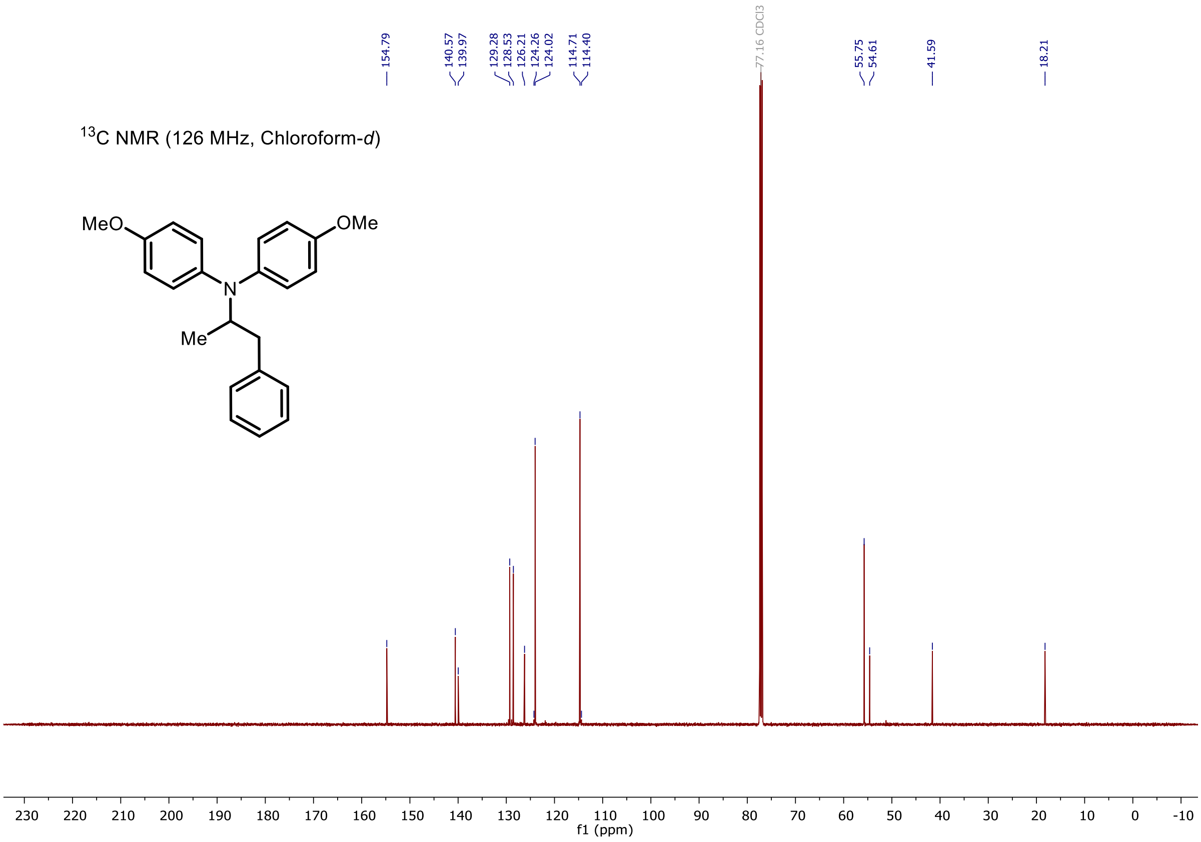

^{13}C NMR (126 MHz, Chloroform-d)
MeO
OMe
N
Me
154.79 140.57 139.97 129.28 128.53 126.21 124.26 124.02 114.71 114.40 77.16 CDCl3 55.75 54.61 41.59 18.21
f1 (ppm)

N,N-bis(4-methoxyphenyl)-2,3-dihydro-1H-inden-1-amine

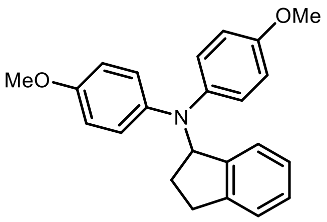

The title compound was prepared according to general procedure 2 using bis(4-methoxyphenyl)amine (500 mg, 2.18 mmol), 1-indanone (340 mg, 2.6 mmol) and polymethylhydrosiloxane as the reductant (0.49 mL, 4.36 mmol). Purification by column chromatography on silica gel (eluent = 15% EtOAc in petroleum ether, 40 × 180 mm silica) gave the title compound as a brown oil (128 mg, 17%); R_f = 0.53 (eluent = 15% EtOAc in petroleum ether); vmax / cm-1 (film) 3066, 2980, 2970, 2831, 1503, 1460, 1440, 1379, 1236, 1178, 817, 754; **^{1}H NMR (500 MHz, Chloroform-*d*)** δ 7.44 – 7.40 (m, 1H), 7.19 – 7.13 (m, 3H), 6.81 – 6.72 (m, 8H), 5.54 (t, *J* = 7.4 Hz, 1H), 3.75 (s, 6H), 2.82 – 2.73 (m, 1H), 2.70 – 2.63 (m, 1H), 2.36 (dddd, *J* = 13.0, 8.4, 7.9, 4.6 Hz, 1H), 2.22 (ddt, *J* = 13.0, 8.8, 7.1 Hz, 1H); **^{13}C{^{1}H} NMR (126 MHz, Chloroform-*d*)** δ 154.7, 144.1, 143.7, 141.8, 127.5, 126.3, 125.4, 125.0, 124.0, 114.5, 65.4, 55.7, 30.4, 29.9; HRMS (CI-QUADRUPOLE) calculated $[C_{23}H_{23}NO_2]^+$ $(M)^+$: m/z 345.1723, found 345.1725.

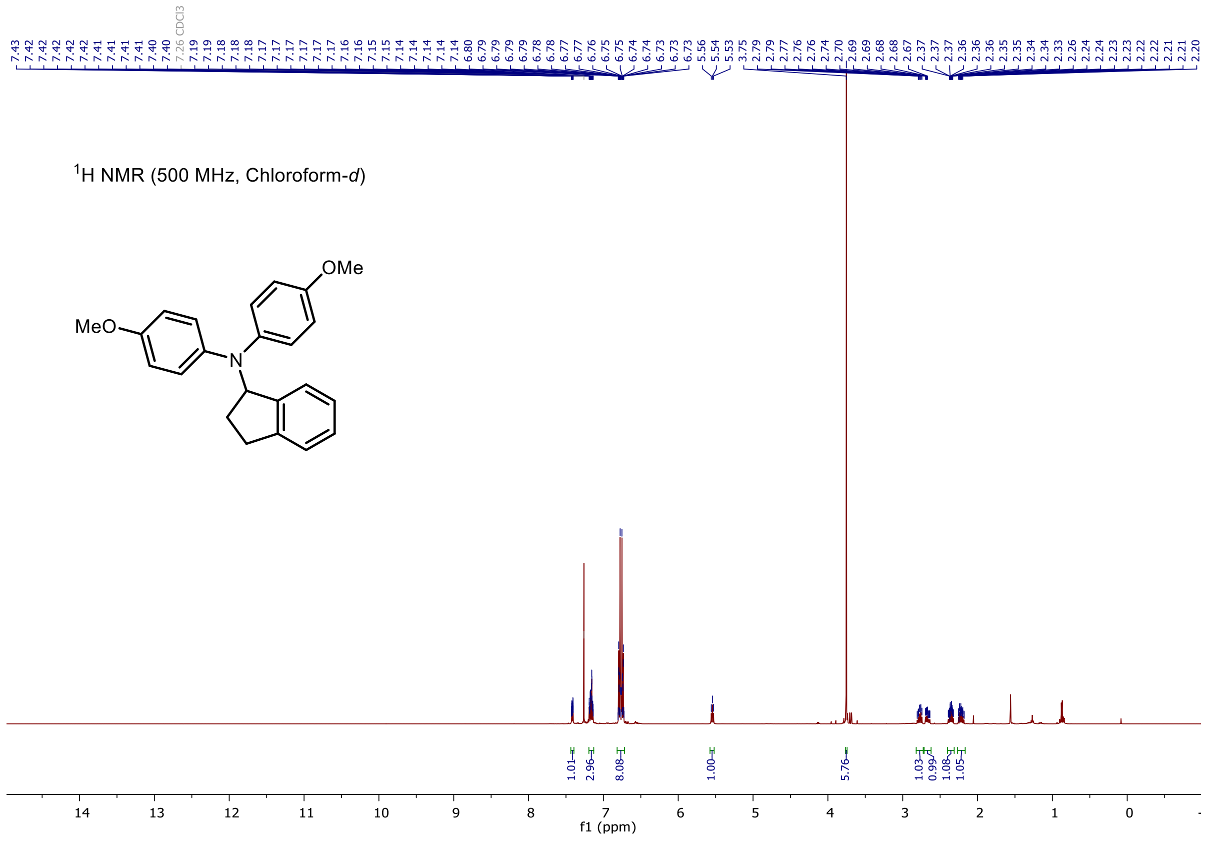

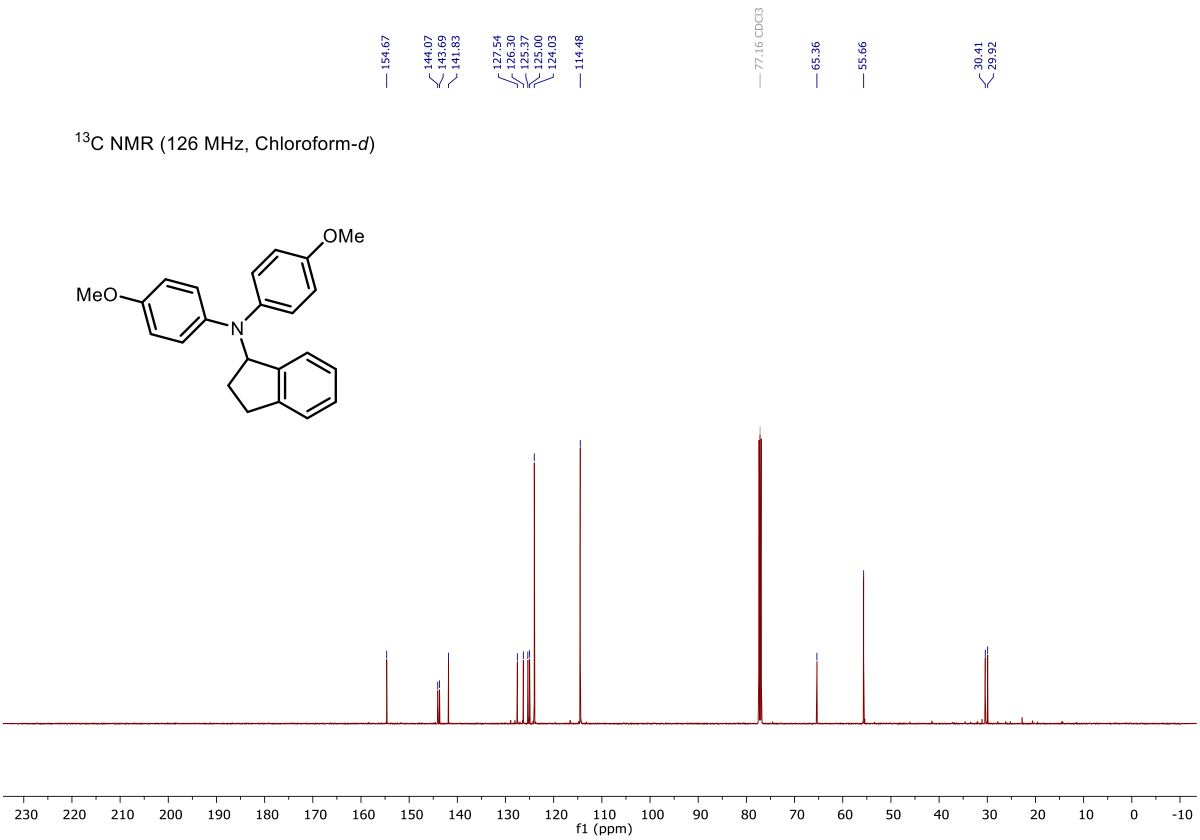

2.2.2. General procedure 3:

4-methoxy-N-(1-phenylethyl)aniline (3)

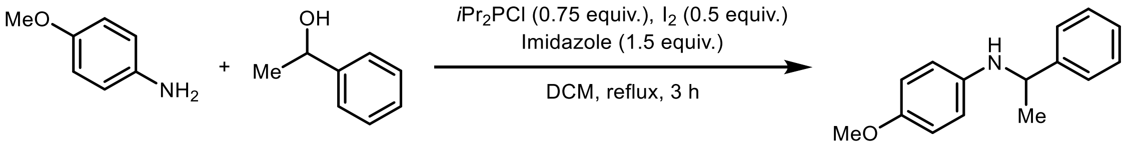

The title compound was prepared according to a modified literature procedure.[5] A flame-dried flask was charged with Iodine (1.27 g, 5 mmol), imidazole (1 g, 14.7 mmol) and chlorodiisopropylphosphine (1.19 mL, 7.5 mmol) and the mixture was stirred under N_2 for 1 min. Dry DCM was then added and the resulted yellow slurry was heated to reflux. 1-phenylethanol (1.2 mL, 10 mmol) was then added which turns the yellow colour of the solution to white and this was followed by the addition of 4-aminoanisole (1.23 g, 10 mmol). The reaction mixture was refluxed for 3h and after cooling, it was washed with saturated aqueous Na_2CO_3 (1 x 50 mL). The organic layer was separated, washed with aqueous sodium thiosulfate solution (1 x 50 mL) and then water (1 x 50 mL), dried over $MgSO_4$, filtered, and concentrated *in vacuo*. Purification by column chromatography on silica gel (eluent = 20% EtOAc in petroleum ether, 70 × 180 mm silica) gave the title compound as a light brown solid (839 mg, 73%); mp 63-66 °C; R_f = 0.58 (eluent = 20% EtOAc in petroleum ether); vmax /cm-1 (film) 1508, 1446, 1371, 1321, 1292, 1232, 1203, 1178, 1139, 1068, 1035, 1018, 817, 754, 700, 538, 518; **^{1}H NMR (500 MHz, Chloroform-*d*)** δ 7.38 – 7.34 (m, 2H), 7.34 – 7.29 (m, 2H), 7.24 – 7.20 (m, 1H), 6.74 – 6.66 (m, 2H), 6.52 – 6.44 (m, 2H), 4.42 (q, *J* = 6.7 Hz, 1H), 3.70 (s, 3H), 1.50 (d, *J* = 6.7 Hz, 3H); **^{13}C{^{1}H} NMR (126 MHz, Chloroform-*d*)** δ 152.0, 145.6, 141.7, 128.7, 127.0,

126.0, 114.9, 114.7, 55.9, 54.4, 25.3; HRMS (ESI-TOF) calculated $[C_{15}H_{18}NO]^+$ $(M + H)^+$: m/z 228.1388, found 228.1382.

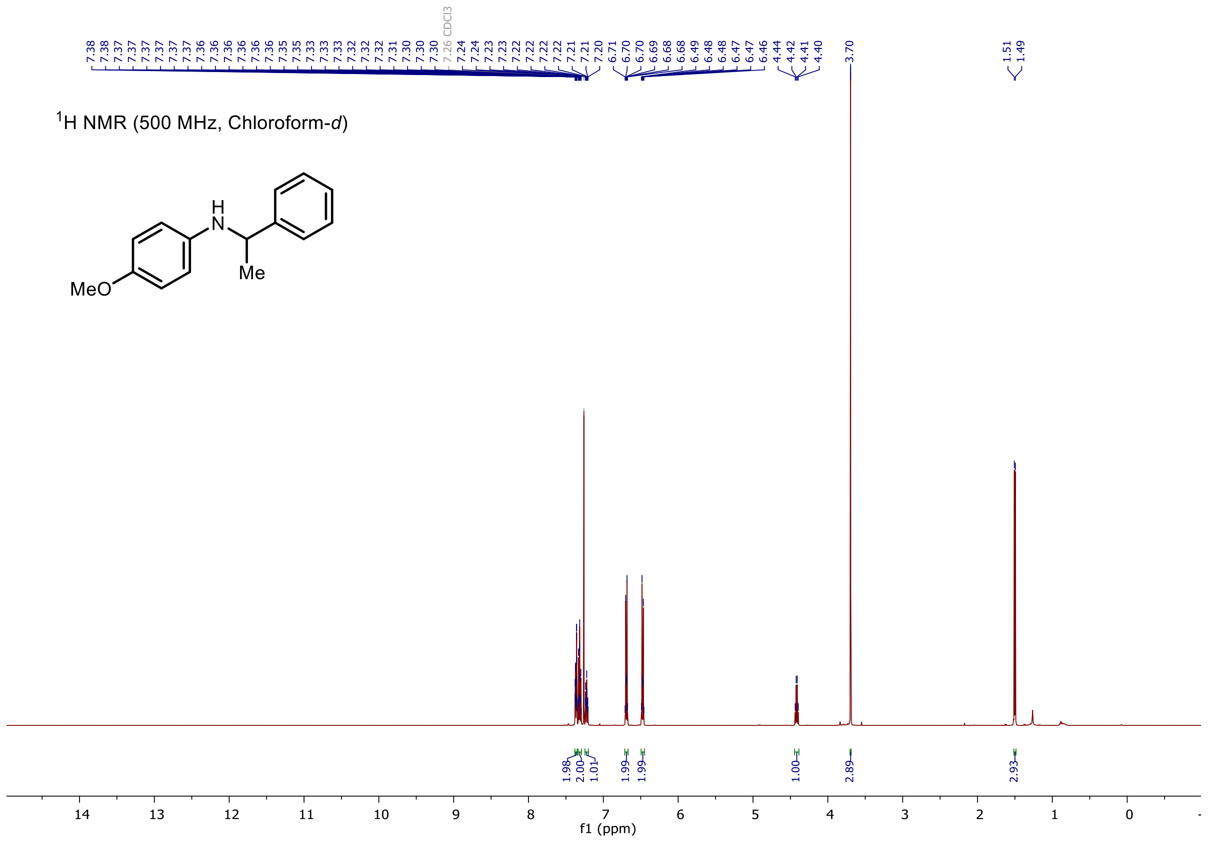

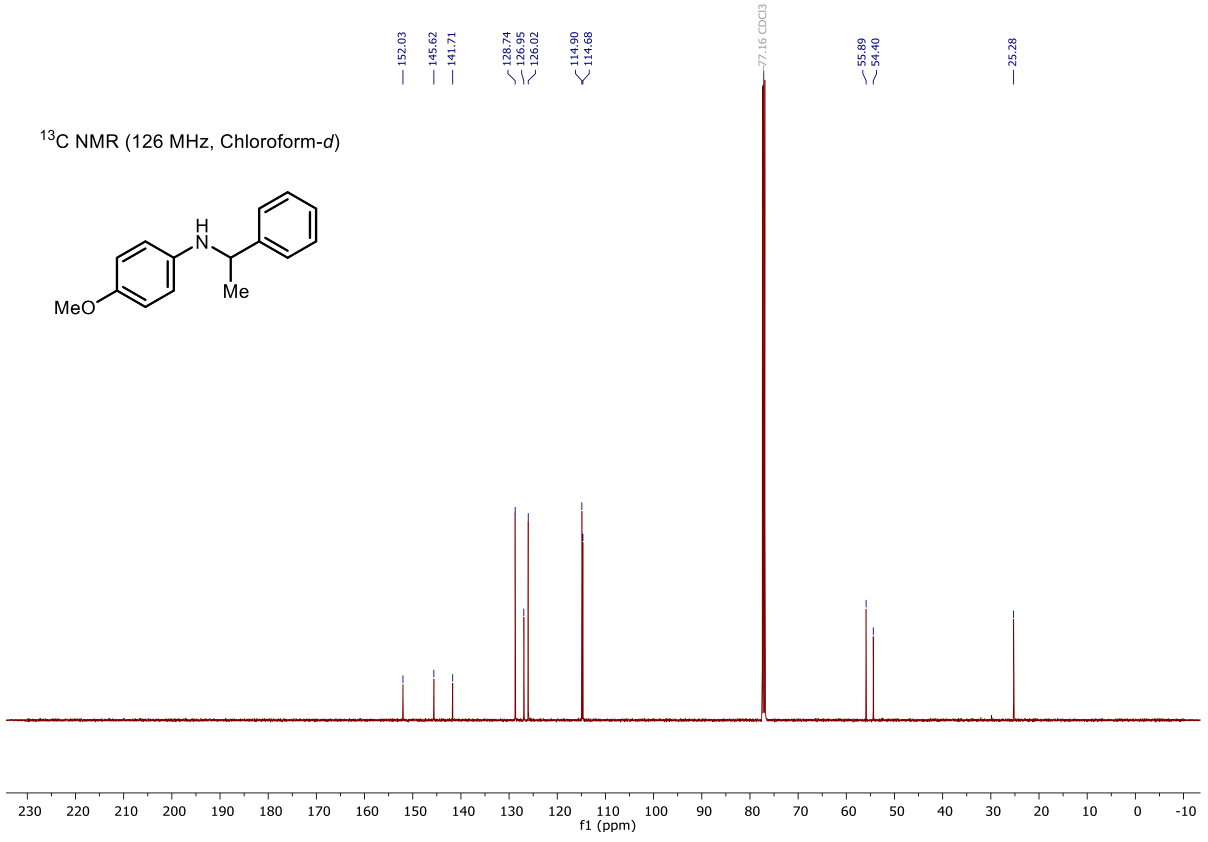

4-methoxy-2,6-dimethyl-N-(1-phenylethyl)aniline (4)

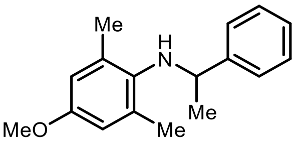

The title compound was prepared according to general procedure 3 using 4-methoxy-2,6-dimethylaniline (175 mg, 1.16 mmol) and chlorodiphenylphosphine (0.16 mL, 0.87 mmol). Purification by column chromatography on silica gel (eluent = 20% EtOAc in hexanes, 40 × 180 mm silica) gave the title compound as a red oil (97 mg, 66%); R_f = 0.83 (eluent = 20% EtOAc in hexanes); vmax / cm-1 (film) 2924, 1602, 1483, 1452, 1371, 1315, 1219, 1149, 1066; **^{1}H NMR (500 MHz, Chloroform-*d*)** δ 7.32 – 7.27 (m, 4H), 7.26 – 7.22 (m, 1H), 6.53 (q, *J* = 0.6 Hz, 2H), 4.14 (q, *J* = 6.8 Hz, 1H), 3.74 (s, 3H), 2.13 (t, *J* = 0.6 Hz, 6H), 1.50 (d, *J* = 6.8 Hz, 3H); **^{13}C{^{1}H} NMR (126 MHz, Chloroform-*d*)** δ 154.7, 132.1, 128.5, 127.1, 126.5, 114.0, 57.7, 55.4, 22.3, 19.1; HRMS (CI-QUADRUPOLE) calculated $[C_{17}H_{21}ON]^+$ $(M)^+$ m/z: 255.1618, found 255.1619.

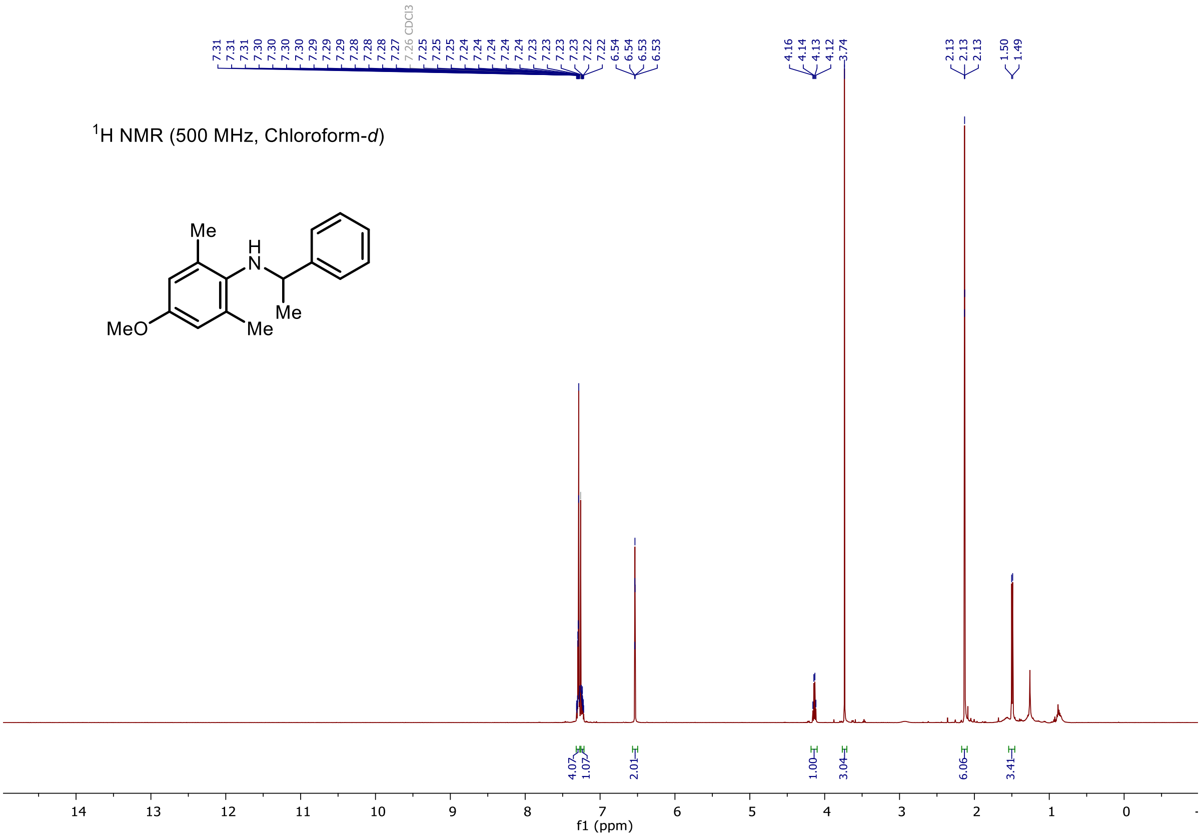

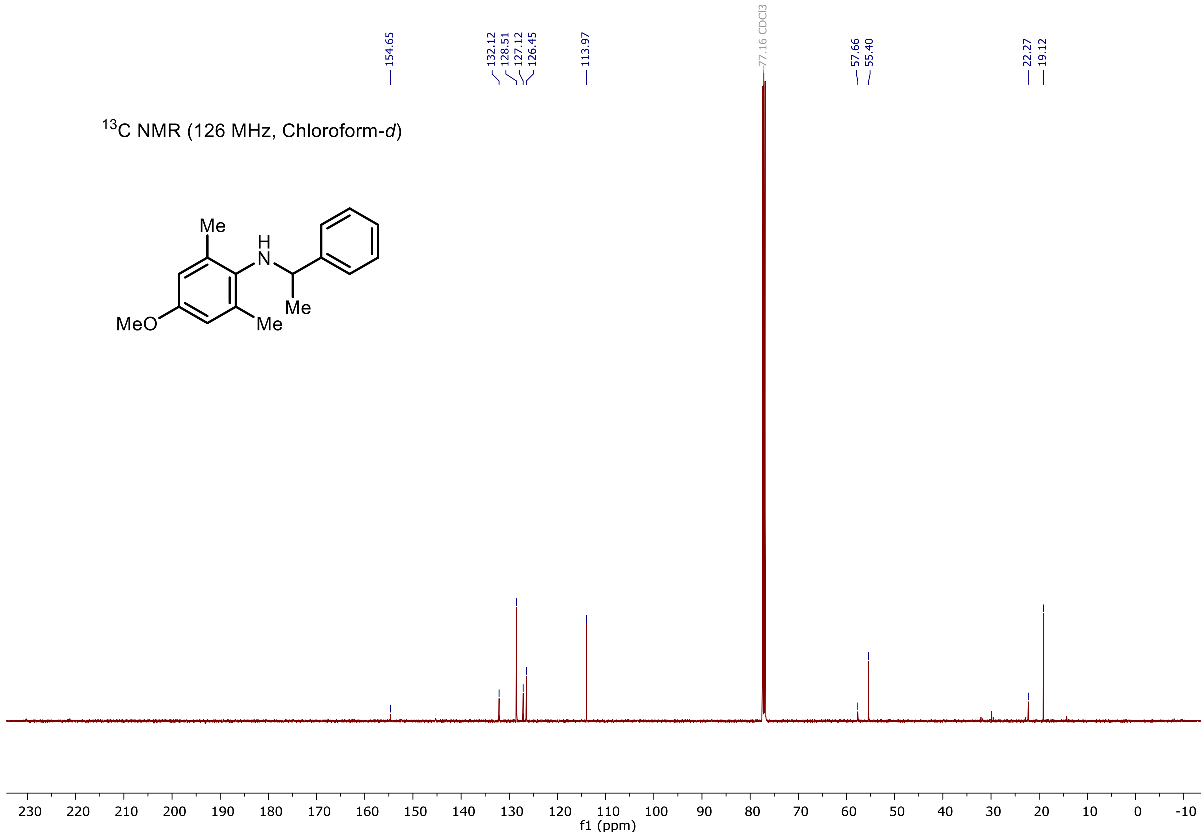

4-methyl-N-(1-phenylethyl)-N-(p-tolyl)aniline (6)

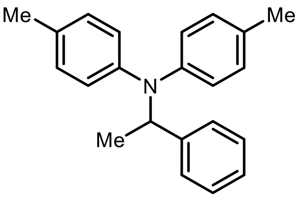

The title compound was prepared according to general procedure 3 using 4,4'-dimethyldiphenylamine (2 g, 10.24 mmol) and chlorodiphenylphosphine (1.38 mL, 7.7 mmol). Purification by column chromatography on silica gel (eluent = 10% EtOAc in petroleum ether, 40 × 180 mm silica) gave the title compound as an amber oil (829 mg, 54%); R_f = 0.86 (eluent = 10% EtOAc in petroleum ether); vmax /cm-1 (film) 3024, 2976, 1566, 1506, 1448, 1375, 1238, 1188, 1085, 1018, 802, 725, 698, 572; **^{1}H NMR (500 MHz, Chloroform-*d*)** δ 7.38 (ddt, *J* = 7.7, 1.4, 0.7 Hz, 2H), 7.32 – 7.26 (m, 2H), 7.24 – 7.17 (m, 1H), 7.02 – 6.98 (m, 4H), 6.79 – 6.75 (m, 4H), 5.23 (q, *J* = 7.0 Hz, 1H), 2.27 (s, 6H), 1.48 (d, *J* = 7.2 Hz, 3H); **^{13}C{^{1}H} NMR (126 MHz, Chloroform-*d*)** δ 144.9, 144.3, 131.1, 129.7, 128.5, 127.1, 126.8, 123.0, 58.0, 20.8, 19.9; HRMS (ESI-TOF) calculated $[C_{22}H_{24}N]^+$ $(M + H)^+$ m/z: 302.1909, found 302.1912.

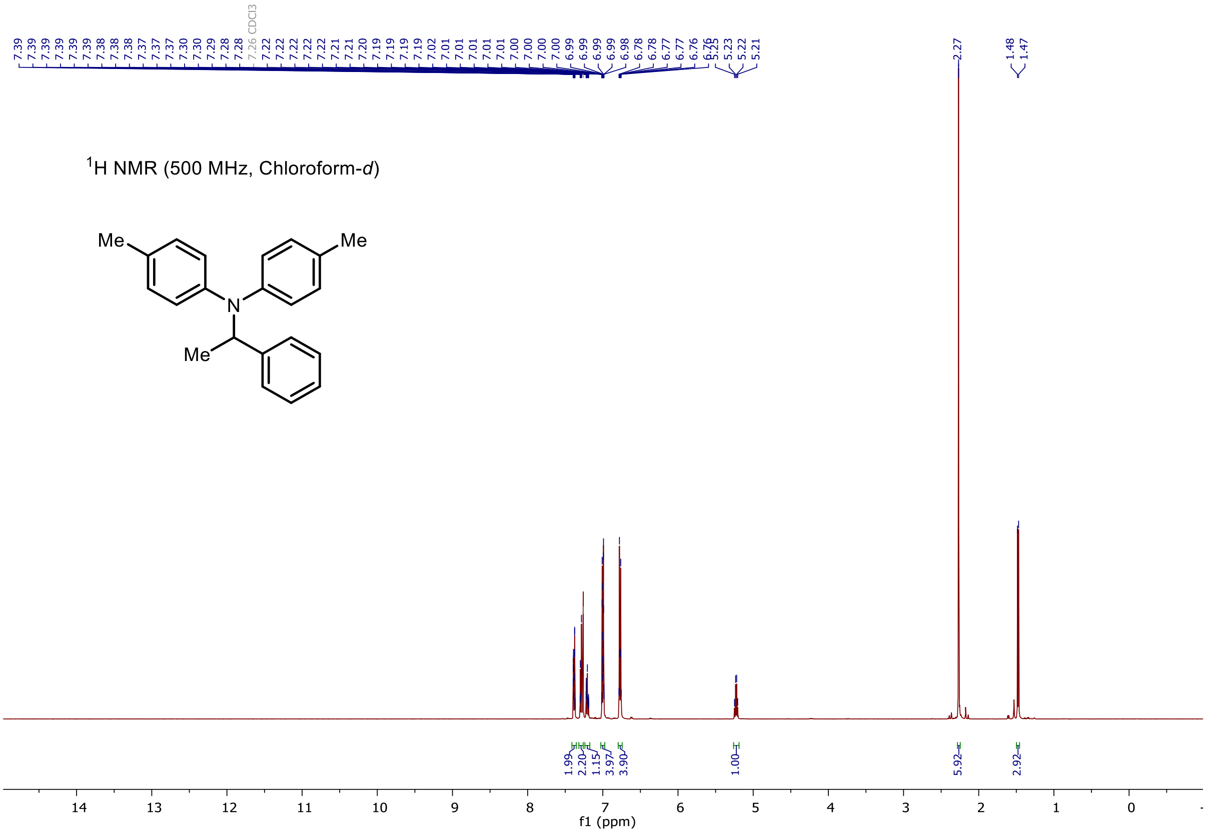
7.39 7.39 7.39 7.39 7.39 7.39 7.38 7.38 7.38 7.37 7.37 7.37 7.30 7.30 7.29 7.28 7.28 7.26 CDCl3 7.22 7.22 7.22 7.22 7.22 7.21 7.21 7.20 7.19 7.19 7.19 7.19 7.02 7.01 7.01 7.01 7.01 7.01 7.00 7.00 7.00 7.00 6.99 6.99 6.99 6.99 6.98 6.78 6.78 6.77 6.77 6.76 6.76 5.25 5.23 5.22 5.21
2.27
1.48 1.47
1H NMR (500 MHz, Chloroform-d)
Me
Me
N
Me
1.99 2.20 1.15 3.97 3.90
1.00
5.92
2.92
14 13 12 11 10 9 8 7 6 5 4 3 2 1 0
f1 (ppm)

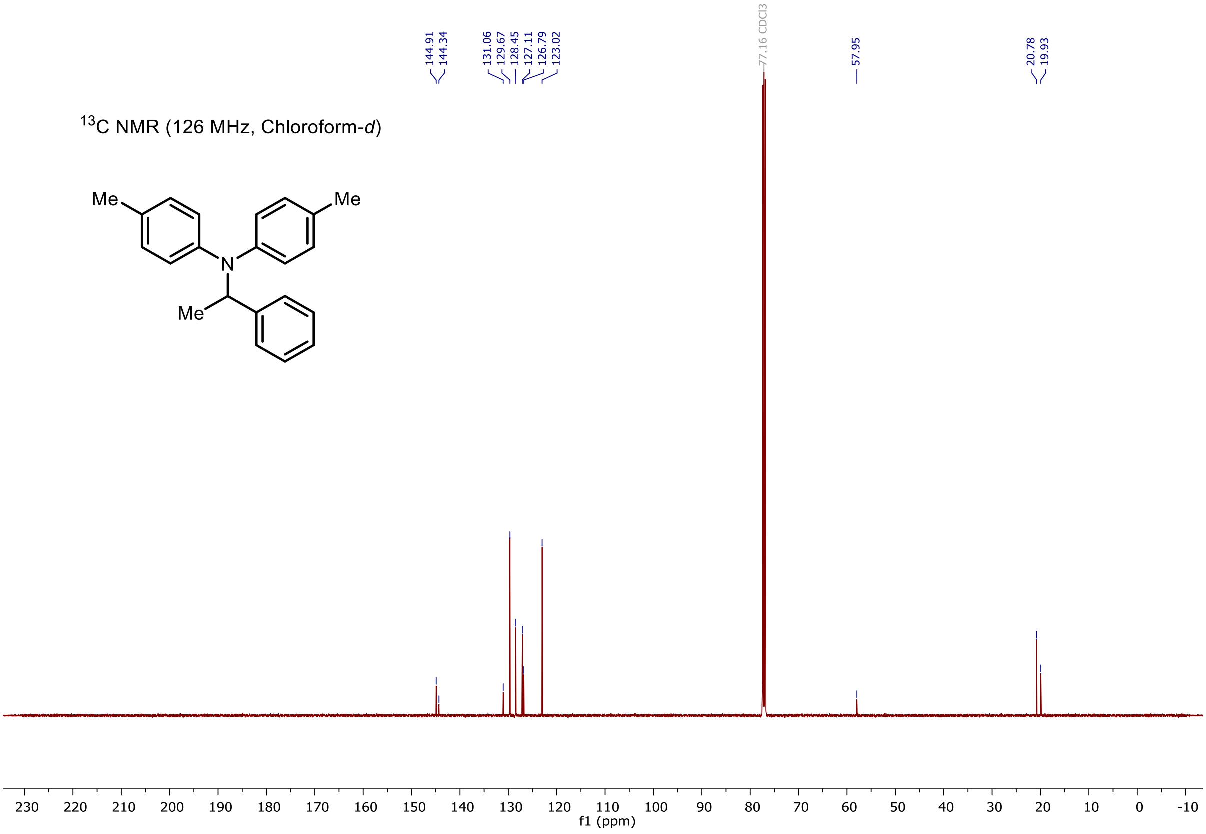
144.91 144.34
131.06 129.67 128.45 127.11 126.79 123.02
77.16 CDCl3
57.95
20.78 19.93
13C NMR (126 MHz, Chloroform-d)
Me
Me
N
Me
230 220 210 200 190 180 170 160 150 140 130 120 110 100 90 80 70 60 50 40 30 20 10 0 -10
f1 (ppm)

4-methoxy-N-(4-methoxyphenyl)-N-(1-phenylethyl)aniline (7)

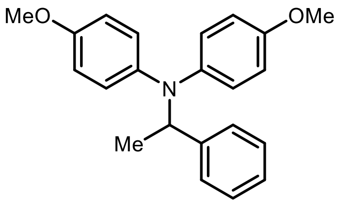

The title compound was prepared according to general procedure 3 using bis(4-methoxyphenyl)amine (1 g, 4.36 mmol). Purification by column chromatography on silica gel (eluent = 10% EtOAc in petroleum ether, 40 × 180 mm silica) gave the title compound as a brown oil (600 mg, 83%); R_f = 0.9 (eluent = 20% EtOAc in hexanes); **^{1}H NMR (500 MHz, Chloroform-*d*)** δ 7.38 – 7.35 (m, 2H), 7.31 – 7.27 (m, 2H), 7.23 – 7.18 (m, 1H), 6.81 – 6.73 (m, 8H), 5.14 (q, *J* = 7.0 Hz, 1H), 3.75 (s, 6H), 1.43 (d, *J* = 7.0 Hz, 3H); **^{13}C{^{1}H} NMR (126 MHz, Chloroform-*d*)** δ 154.6, 144.7, 141.4, 128.4, 127.1, 126.8, 124.3, 114.4, 58.1, 55.7, 20.4. The spectroscopic data are in accordance with those described in the literature.[6]

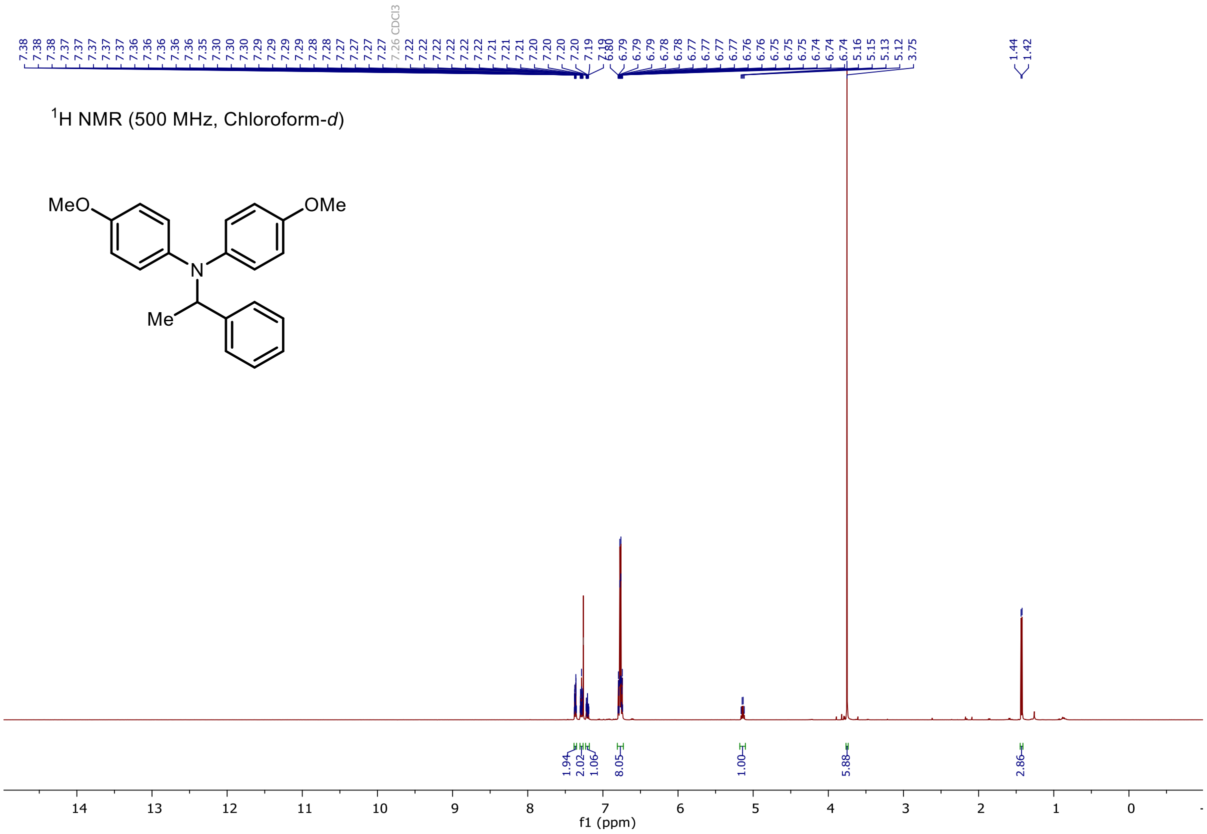

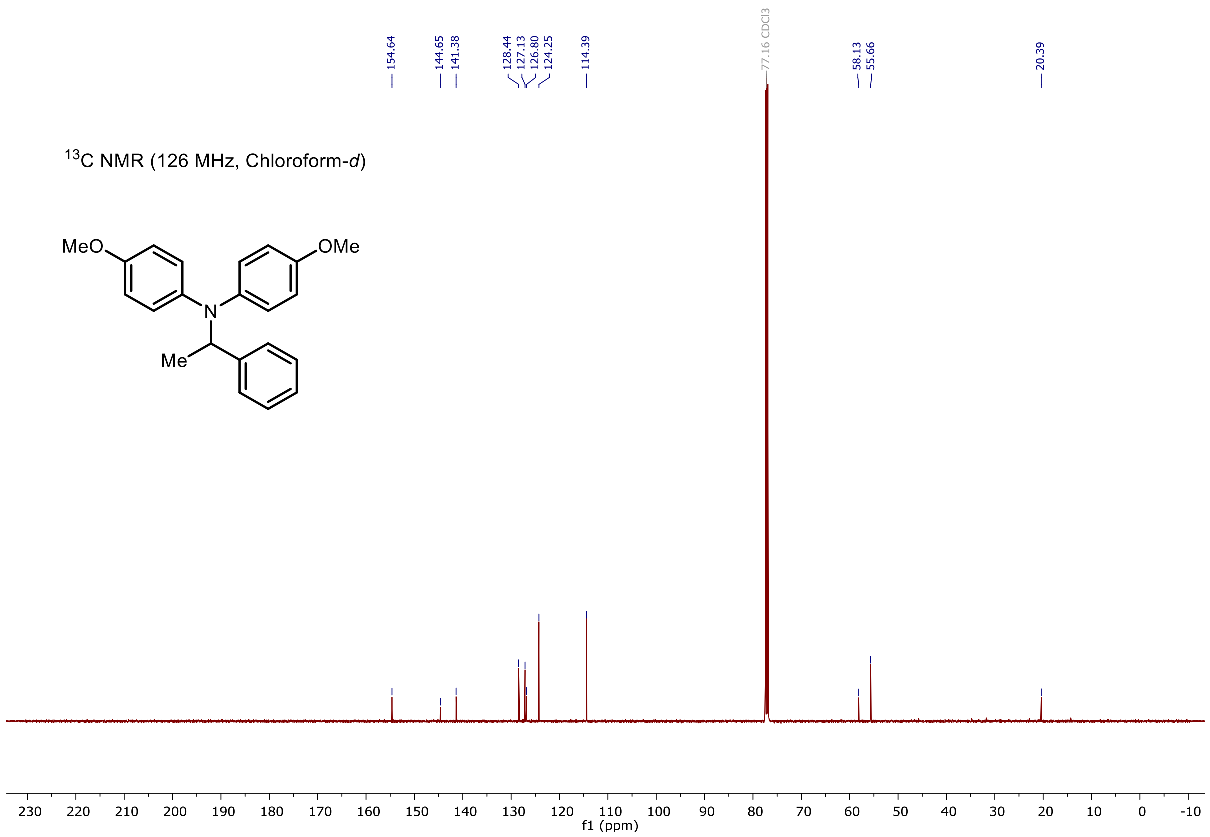

4-methoxy-N-(4-methoxyphenyl)-N-(1-phenylpropyl)aniline

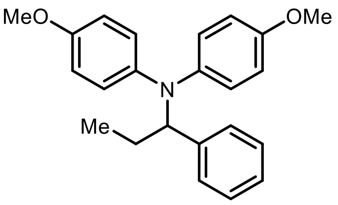

The title compound was prepared according to general procedure 3 using bis(4-methoxyphenyl)amine (500 mg, 2.18 mmol) and 1-phenyl-1-propanol (300 mg, 2.18 mmol). Purification by column chromatography on silica gel (eluent = 15% EtOAc in petroleum ether, 40 × 180 mm silica) gave the title compound as a brown oil (180 mg, 47%); R_f = 0.79 (eluent = 15% EtOAc in petroleum ether); vmax /cm-1 (film) 2980, 2970, 2831, 1503, 1460, 1440, 1379, 1236, 1035, 952, 815, 698; **^{1}H NMR (500 MHz, Chloroform-*d*)** δ 7.25 – 7.17 (m, 5H), 6.81 – 6.71 (m, 8H), 4.94 (dd, *J* = 7.9, 7.0 Hz, 1H), 3.75 (s, 6H), 2.02 – 1.95 (m, 1H), 1.94 – 1.86 (m, 1H), 0.92 (t, *J* = 7.3 Hz, 3H); **^{13}C{^{1}H} NMR (126 MHz, Chloroform-*d*)** δ 154.6, 142.4, 141.5, 128.2 (d, *J* = 2.9 Hz), 126.9, 124.3, 114.4, 64.5, 55.7, 25.6, 11.8; HRMS (ESI-TOF) calculated $[C_{23}H_{26}NO_2]^+$ $(M + H)^+$ m/z 348.1964, found 348.1957.

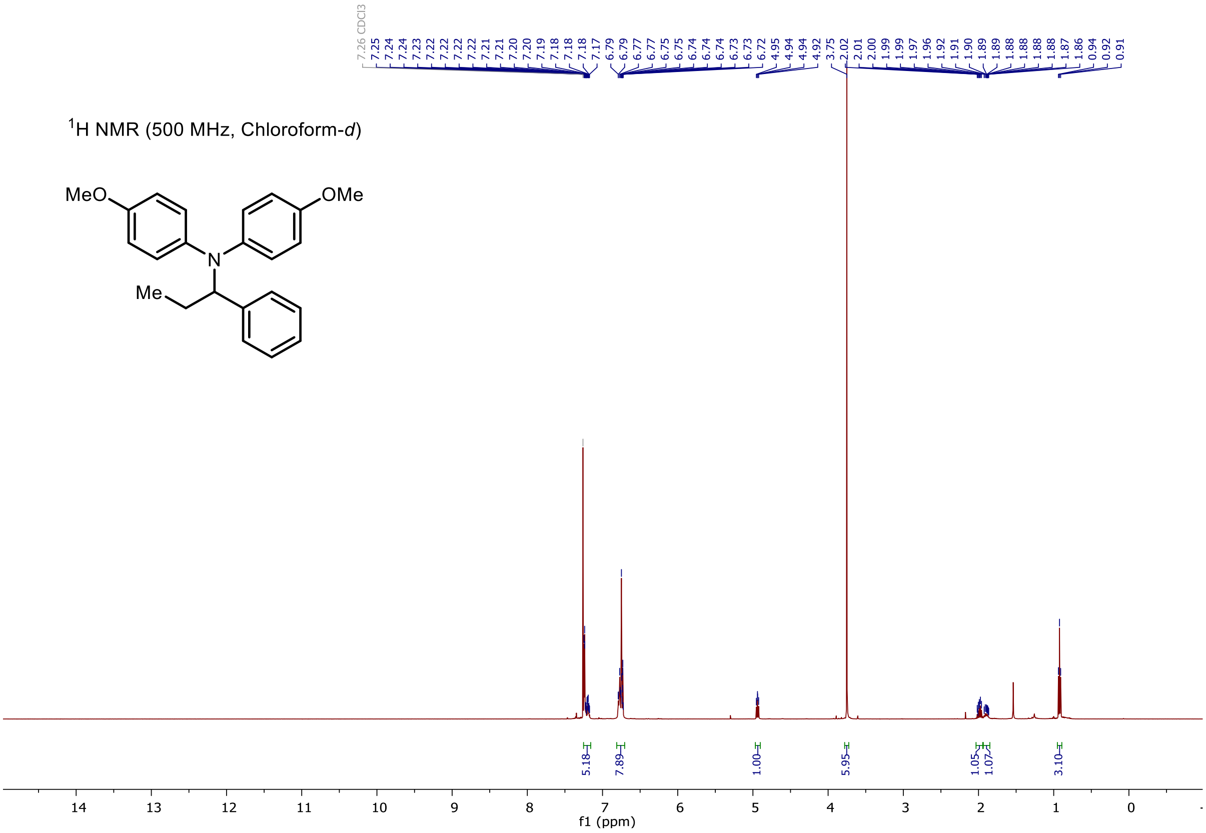
1H NMR (500 MHz, Chloroform-d)
MeO
OMe
N
Me
f1 (ppm)

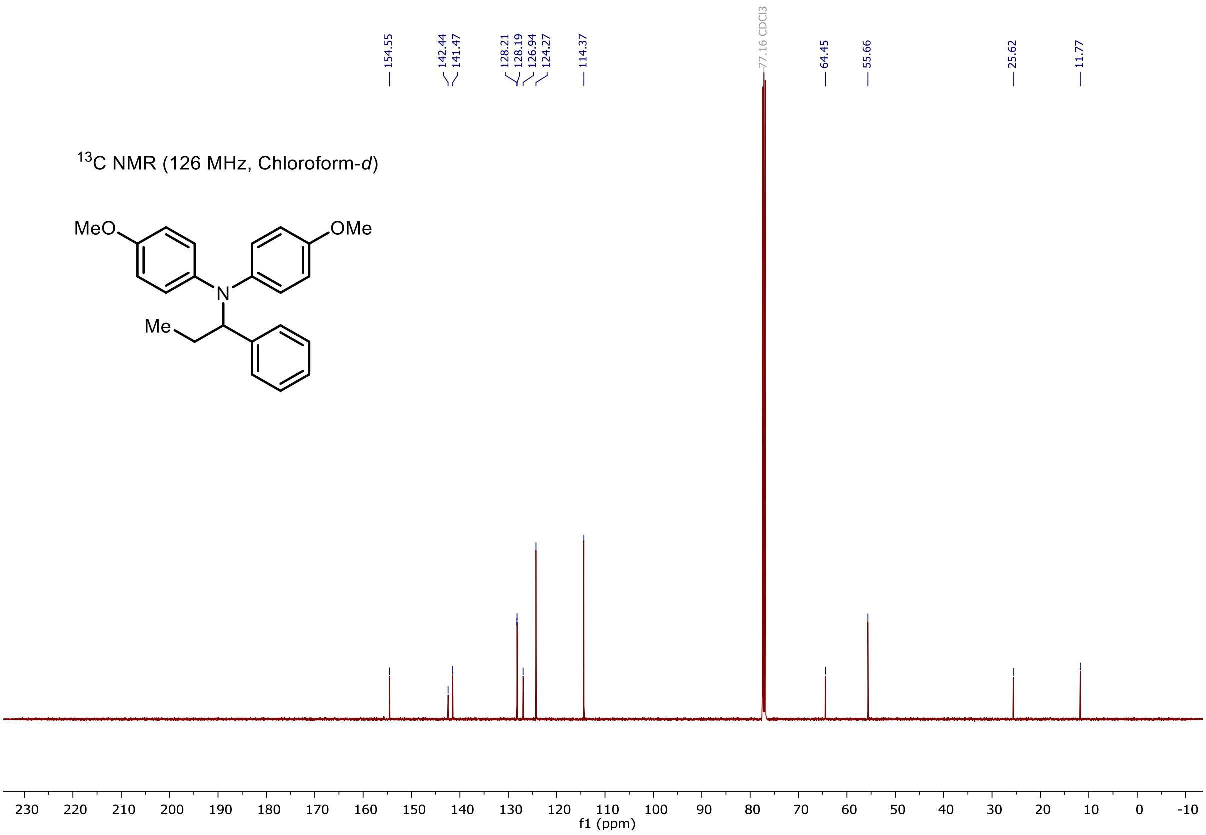
13C NMR (126 MHz, Chloroform-d)
MeO
OMe
N
Me
154.55
142.44
141.47
128.21
128.19
126.94
124.27
114.37
77.16 CDCl3
64.45
55.66
25.62
11.77
f1 (ppm)

N,N-bis(4-methoxyphenyl)-1,2,3,4-tetrahydronaphthalen-1-amine

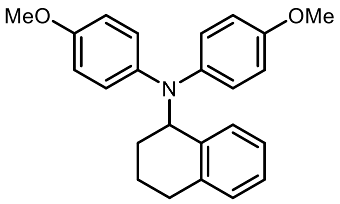

The title compound was prepared according to general procedure 3 using bis(4-methoxyphenyl)amine (500 mg, 2.18 mmol) and 1,2,3,4-tetrahydro-1-naphthol (323 mg, 2.18 mmol). Purification by column chromatography on silica gel (eluent = 15% EtOAc in petroleum ether, 40 × 180 mm silica) gave the title compound as a brown oil (348 mg, 87%); R_f = 0.64 (eluent = 15% EtOAc in petroleum ether); vmax /cm-1 (film) 2981, 2833, 1747, 1687, 1525, 1508, 1458, 1379, 1261, 1236, 1087, 1037, 987; **^{1}H NMR (500 MHz, Chloroform-*d*)** δ 7.57 – 7.54 (m, 1H), 7.16 – 7.08 (m, 3H), 6.85 – 6.80 (m, 4H), 6.77 – 6.73 (m, 4H), 5.13 (t, *J* = 8.4 Hz, 1H), 3.75 (s, 6H), 2.79 (tdd, *J* = 16.4, 11.9, 7.5 Hz, 2H), 2.11 (td, *J* = 9.9, 9.4, 3.8 Hz, 2H), 2.00 – 1.92 (m, 1H), 1.84 – 1.74 (m, 1H); **^{13}C{^{1}H} NMR (126 MHz, Chloroform-*d*)** δ 154.3, 142.0, 138.7, 137.5, 129.2, 128.0, 126.6, 126.1, 123.4, 114.6, 61.1, 55.7, 29.8, 27.4, 22.9; HRMS (ESI-TOF) calculated $[C_{24}H_{26}NO_2]^+$ $(M + H)^+$ m/z 360.1964, found 360.1961.

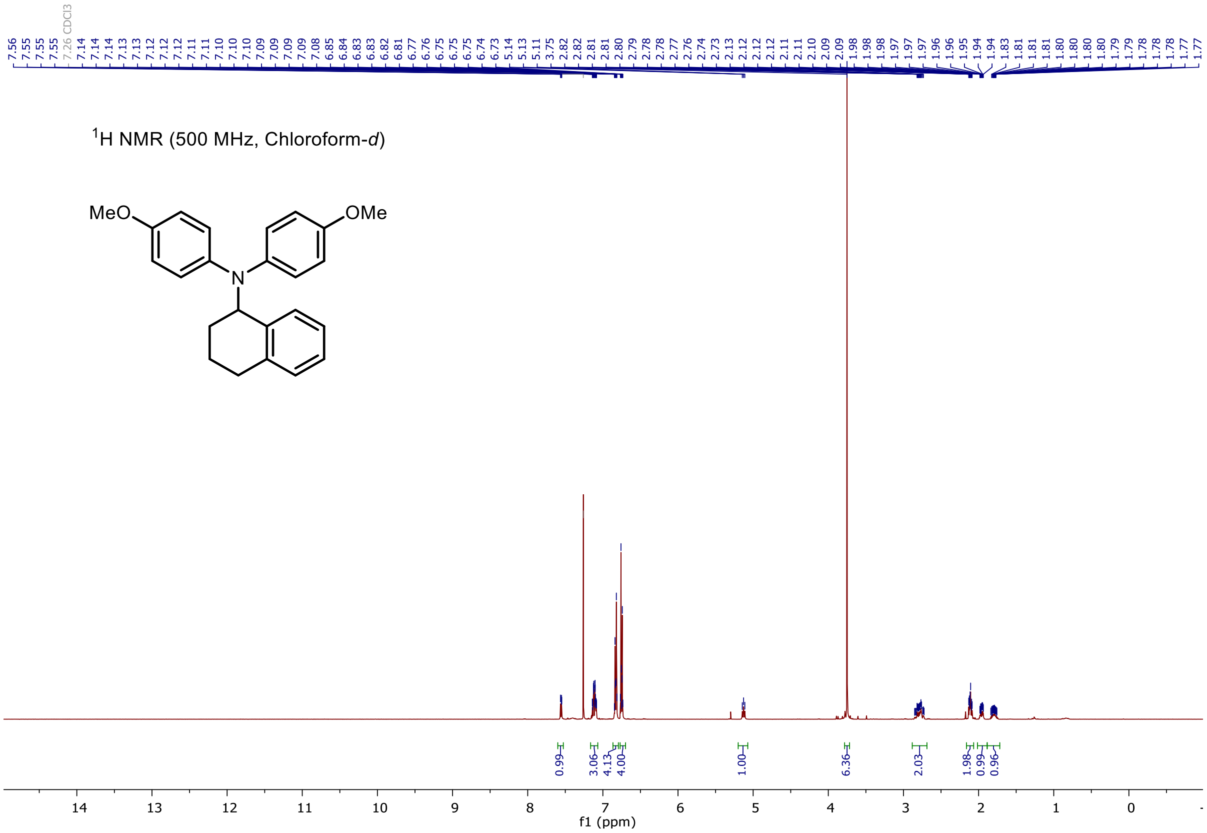

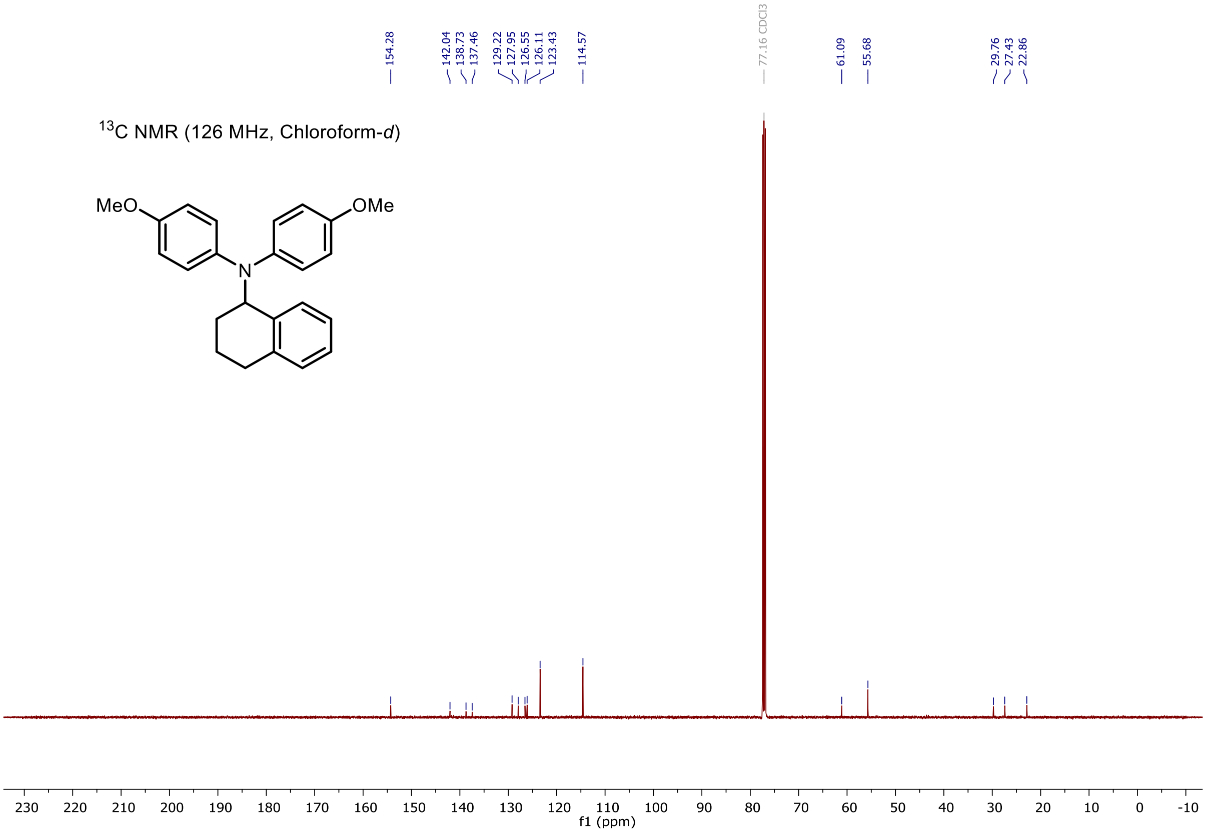

4-methoxy-N-(4-methoxyphenyl)-N-(1-(o-tolyl)ethyl)aniline

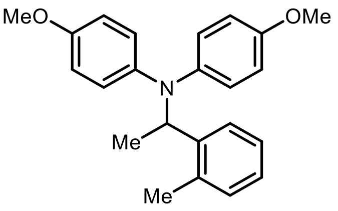

The title compound was prepared according to general procedure 3 using bis(4-methoxyphenyl)amine (500 mg, 2.18 mmol) and 1-(o-tolyl)ethan-1-ol (300 mg, 2.18 mmol). Purification by column chromatography on silica gel (eluent = 20% EtOAc in petroleum ether, 40 × 180 mm silica) gave the title compound as an off-white solid (300 mg, 78%); mp 96-98 °C; R_f = 0.73 (eluent = 20% EtOAc in petroleum ether); vmax / cm-1 (film) 2980, 2970, 2833, 1503, 1462, 1440, 1338, 1238, 1035, 952, 815, 758; **^{1}H NMR (500 MHz, Chloroform-*d*)** δ 7.17 (ddd, *J* = 8.1, 1.4, 0.7 Hz, 1H), 7.12 (ddd, *J* = 7.8, 6.2, 1.4 Hz, 2H), 7.05 – 7.00 (m, 1H), 6.75 – 6.71 (m, 4H), 6.68 – 6.64 (m, 4H), 5.23 (q, *J* = 6.8 Hz, 1H), 3.75 (s, 6H), 2.41 (s, 3H), 1.41 (d, *J* = 6.8 Hz, 3H); **^{13}C{^{1}H} NMR (126 MHz, Chloroform-*d*)** δ 154.7, 141.9, 141.2, 136.6, 130.4, 126.9, 126.4, 126.0, 124.2, 114.3, 55.6, 53.9, 29.9, 19.5, 17.5; HRMS (ESI-TOF) calculated $[C_{23}H_{26}NO_2]^+$ $(M + H)^+$ m/z 348.1964, found 348.1955.

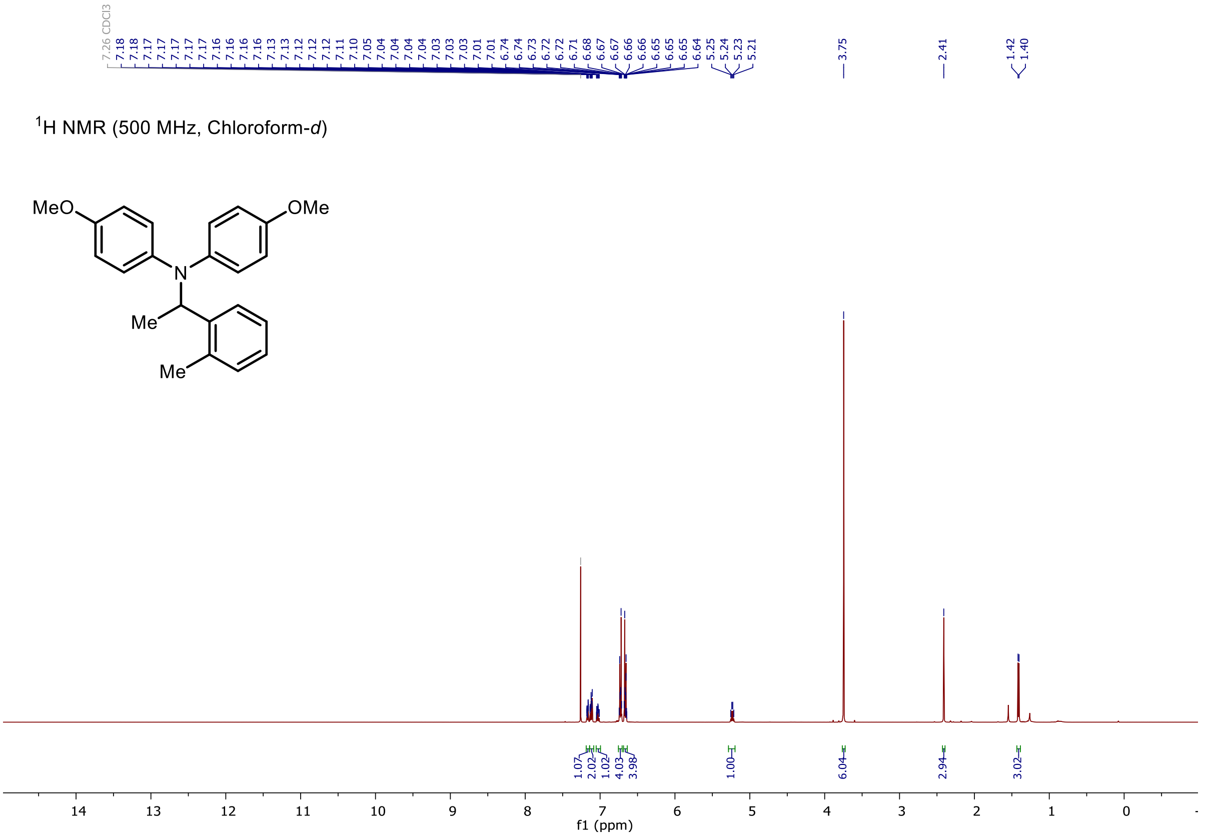
1H NMR (500 MHz, Chloroform-d)
7.26 CDCl3
7.18
7.18
7.17
7.17
7.17
7.17
7.16
7.16
7.16
7.13
7.13
7.12
7.12
7.12
7.11
7.10
7.05
7.04
7.04
7.04
7.03
7.03
7.03
7.01
7.01
6.74
6.74
6.73
6.72
6.72
6.71
6.68
6.67
6.67
6.66
6.66
6.65
6.65
6.65
6.64
5.25
5.24
5.23
5.21
3.75
2.41
1.42
1.40
MeO
OMe
N
Me
Me
1.07
2.02
1.02
4.03
3.98
1.00
6.04
2.94
3.02
14
13
12
11
10
9
8
7
6
5
4
3
2
1
0
f1 (ppm)

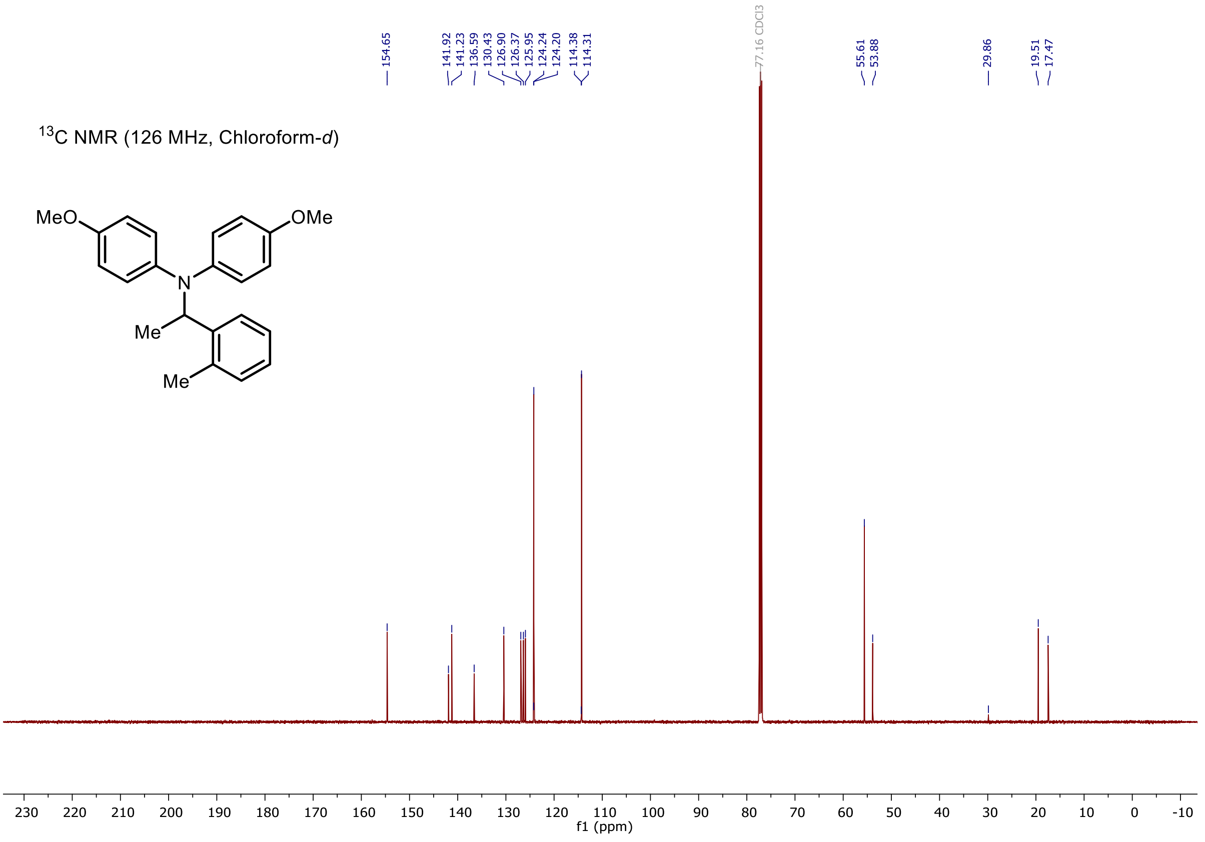
13C NMR (126 MHz, Chloroform-d)
154.65
141.92
141.23
136.59
130.43
126.90
126.37
125.95
124.24
124.20
114.38
114.31
77.16 CDCl3
55.61
53.88
29.86
19.51
17.47
MeO
OMe
N
Me
Me
230
220
210
200
190
180
170
160
150
140
130
120
110
100
90
80
70
60
50
40
30
20
10
0
-10
f1 (ppm)

4-methoxy-N-(4-methoxyphenyl)-N-(1-(m-tolyl)ethyl)aniline

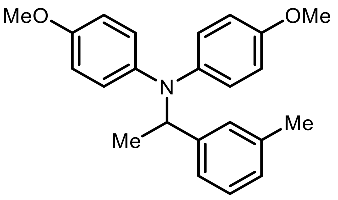

The title compound was prepared according to general procedure 3 using bis(4-methoxyphenyl)amine (500 mg, 2.18 mmol) and 1-(m-tolyl)ethan-1-ol (300 mg, 2.18 mmol). Purification by column chromatography on silica gel (eluent = 20% EtOAc in petroleum ether, 40 × 180 mm silica) gave the title compound as a brown oil (244 mg, 64%); R_f = 0.74 (eluent = 20% EtOAc in petroleum ether); vmax /cm-1 (film) 2980, 2831, 1500, 1460, 1440, 1375, 1338, 1236, 1178, 1101, 1035, 954, 817, 775, 704; **^{1}H NMR (500 MHz, Chloroform-*d*)** δ 7.22 – 7.12 (m, 3H), 7.03 (tt, *J* = 5.5, 3.1 Hz, 1H), 6.82 – 6.73 (m, 8H), 5.10 (q, *J* = 7.0 Hz, 1H), 3.76 (s, 6H), 2.33 (s, 3H), 1.42 (d, *J* = 7.0 Hz, 3H); **^{13}C{^{1}H} NMR (126 MHz, Chloroform-*d*)** δ 154.6, 144.7, 141.5, 137.9, 128.3, 127.9, 127.5, 124.2, 124.1, 114.4, 55.7, 21.7, 20.5; HRMS (ESI-TOF) calculated $[C_{23}H_{26}NO_2]^+$ $(M + H)^+$ m/z 348.1964, found 348.1958.

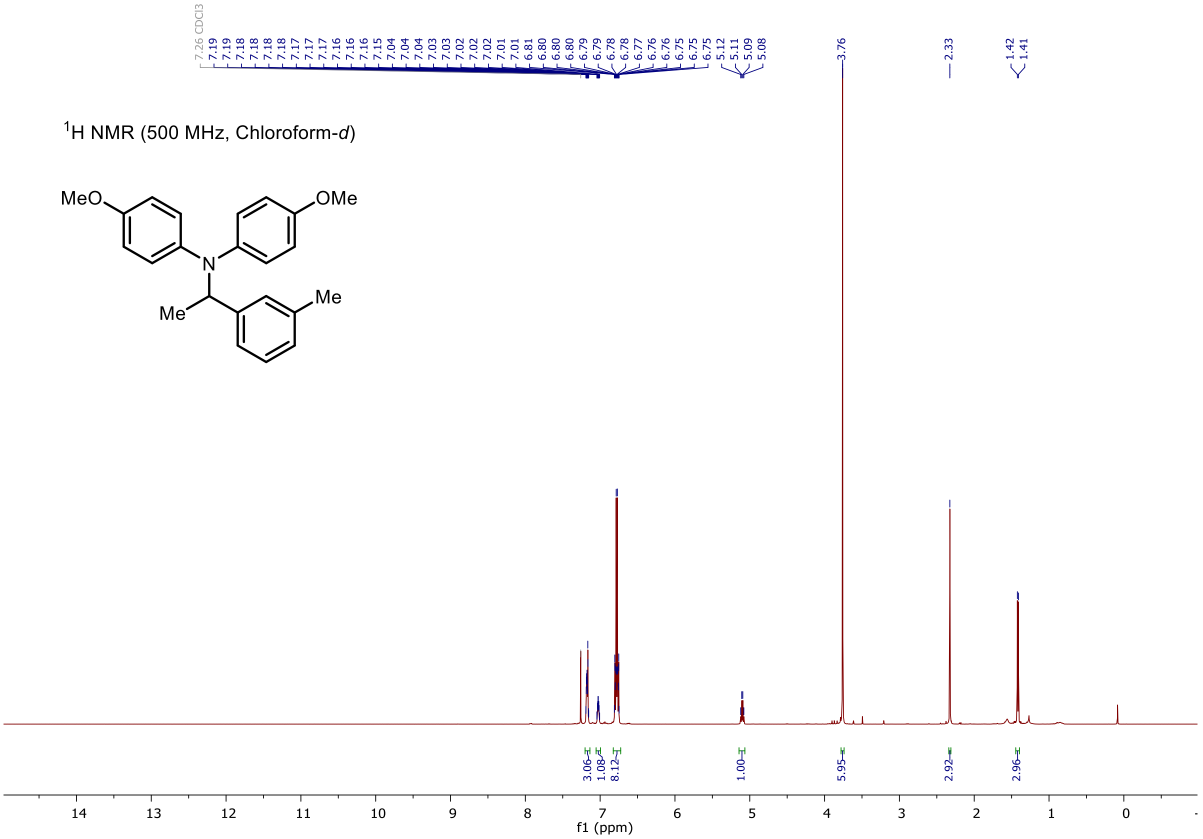

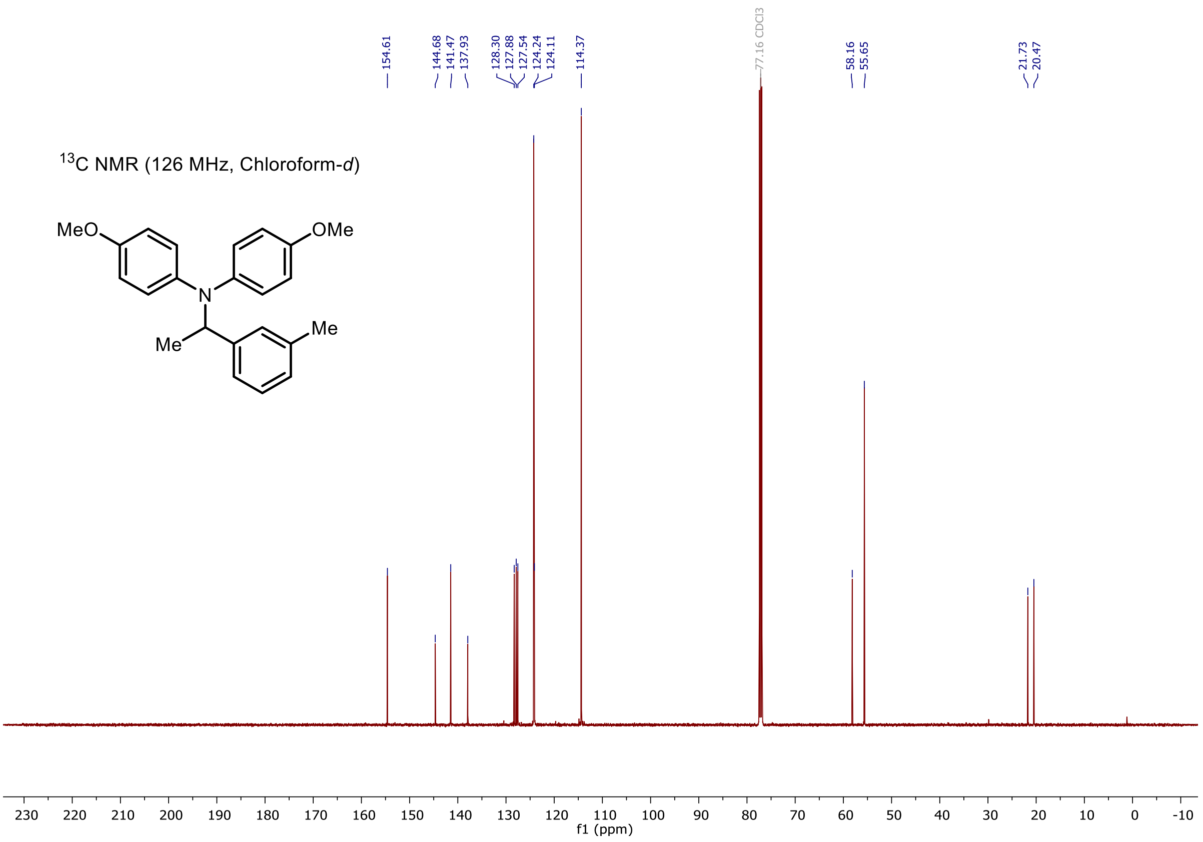

4-methoxy-N-(4-methoxyphenyl)-N-(1-(p-tolyl)ethyl)aniline

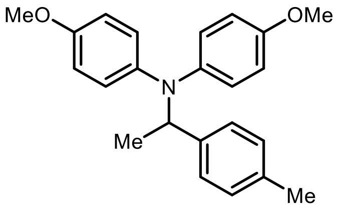

The title compound was prepared according to general procedure 3 using bis(4-methoxyphenyl)amine (500 mg, 2.18 mmol) and 1-(p-tolyl)ethan-1-ol (300 mg, 2.18 mmol). Purification by column chromatography on silica gel (eluent = 20% EtOAc in petroleum ether, 40 × 180 mm silica) gave the title compound as a brown oil (195 mg, 51%); R_f = 0.66 (eluent = 20% EtOAc in petroleum ether); vmax /cm-1 (film) 2831, 1508, 1460, 1336, 1240, 1085, 1037, 985, 912, 856, 819, 727, 412; **^{1}H NMR (500 MHz, Chloroform-*d*)** δ 7.27 – 7.24 (m, 2H), 7.10 – 7.06 (m, 2H), 6.83 – 6.73 (m, 8H), 5.11 (q, *J* = 7.0 Hz, 1H), 3.75 (s, 6H), 2.31 (s, 3H), 1.42 (d, *J* = 7.4 Hz, 2H); **^{13}C{^{1}H} NMR (126 MHz, Chloroform-*d*)** δ 154.6, 141.6, 141.4, 136.3, 129.1, 127.0, 124.2, 114.4, 57.9, 55.7, 21.2, 20.4; HRMS (ESI-TOF) calculated $[C_{23}H_{26}NO_2]^+$ $(M + H)^+$ m/z 348.1964, found 348.1955.

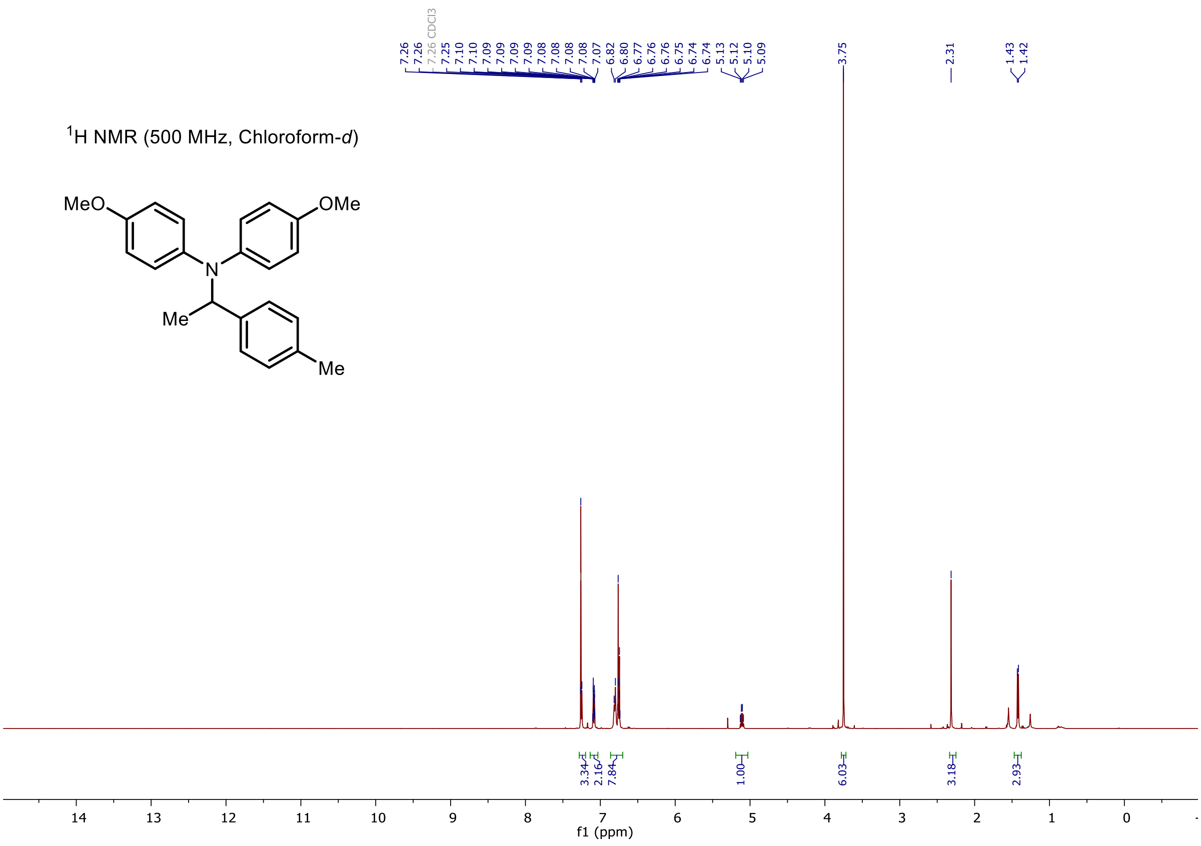
1H NMR (500 MHz, Chloroform-d)
MeO
OMe
N
Me
Me
f1 (ppm)

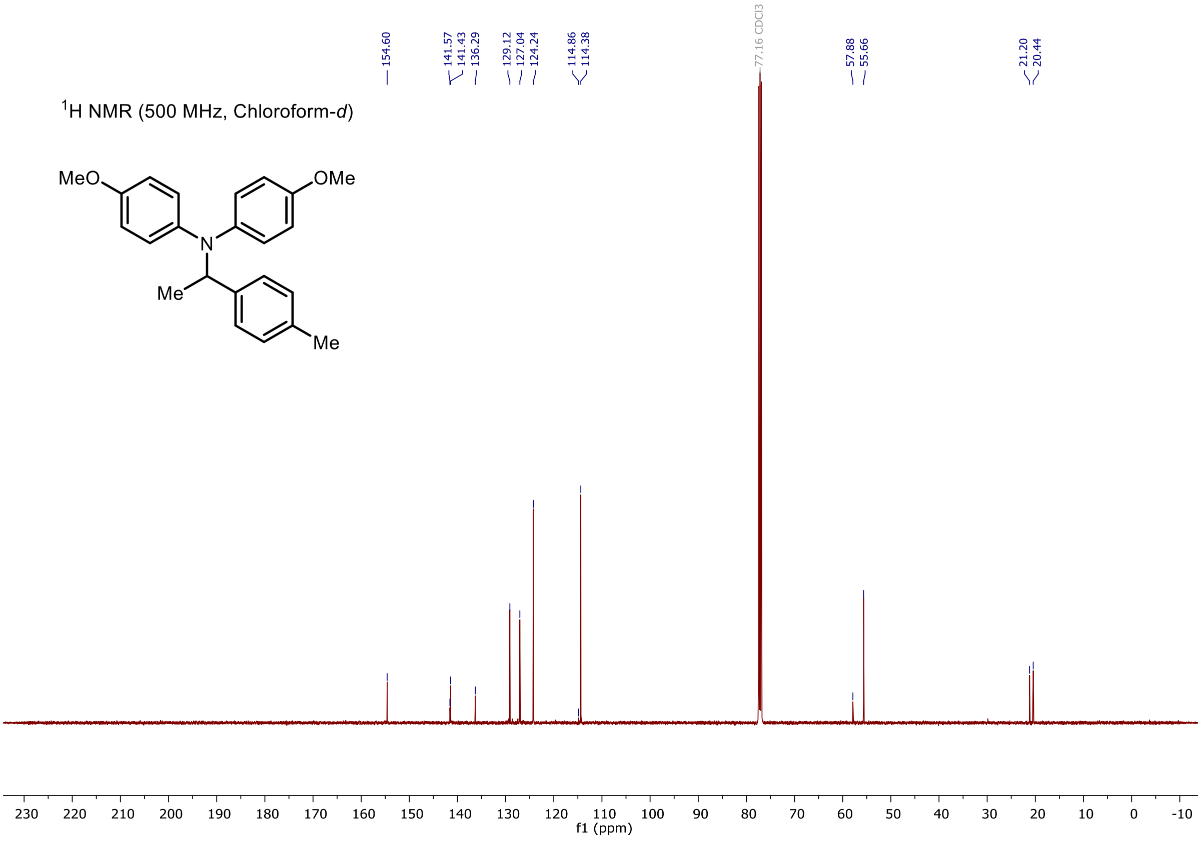
1H NMR (500 MHz, Chloroform-d)
MeO
OMe
N
Me
Me
f1 (ppm)

4-methoxy-N-(4-methoxyphenyl)-N-(1-(3-methoxyphenyl)ethyl)aniline

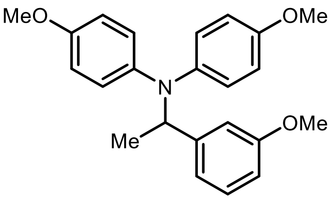

The title compound was prepared according to general procedure 3 using bis(4-methoxyphenyl)amine (500 mg, 2.18 mmol) and 1-(3-methoxyphenyl)ethan-1-ol (330 mg, 2.18 mmol). Purification by column chromatography on silica gel (eluent = 20% EtOAc in petroleum ether, 40 × 180 mm silica) gave the title compound as a yellow oil (160 mg, 40%); R_f = 0.6 (eluent = 20% EtOAc in petroleum ether); vmax /cm-1 (film) 2979, 2970, 2833, 1687, 1543, 1508, 1460, 1379, 1338, 1236, 1087, 952, 860; **^{1}H NMR (500 MHz, Chloroform-*d*)** δ 7.20 (t, *J* = 7.9 Hz, 1H), 6.99 – 6.92 (m, 2H), 6.81 – 6.77 (m, 4H), 6.77 – 6.73 (m, 5H), 5.09 (q, *J* = 7.0 Hz, 1H), 3.76 (s, 3H), 3.75 (s, 6H), 1.41 (d, *J* = 7.0 Hz, 3H); **^{13}C{^{1}H} NMR (126 MHz, Chloroform-*d*)** δ 159.8, 154.7, 146.6, 141.4, 129.4, 124.3, 119.5, 114.4, 113.1, 111.9, 58.3, 55.7, 55.3, 20.6; HRMS (ESI-TOF) calculated $[C_{23}H_{26}NO_3]^+$ $(M + H)^+$ m/z 364.1913, found 364.1909.

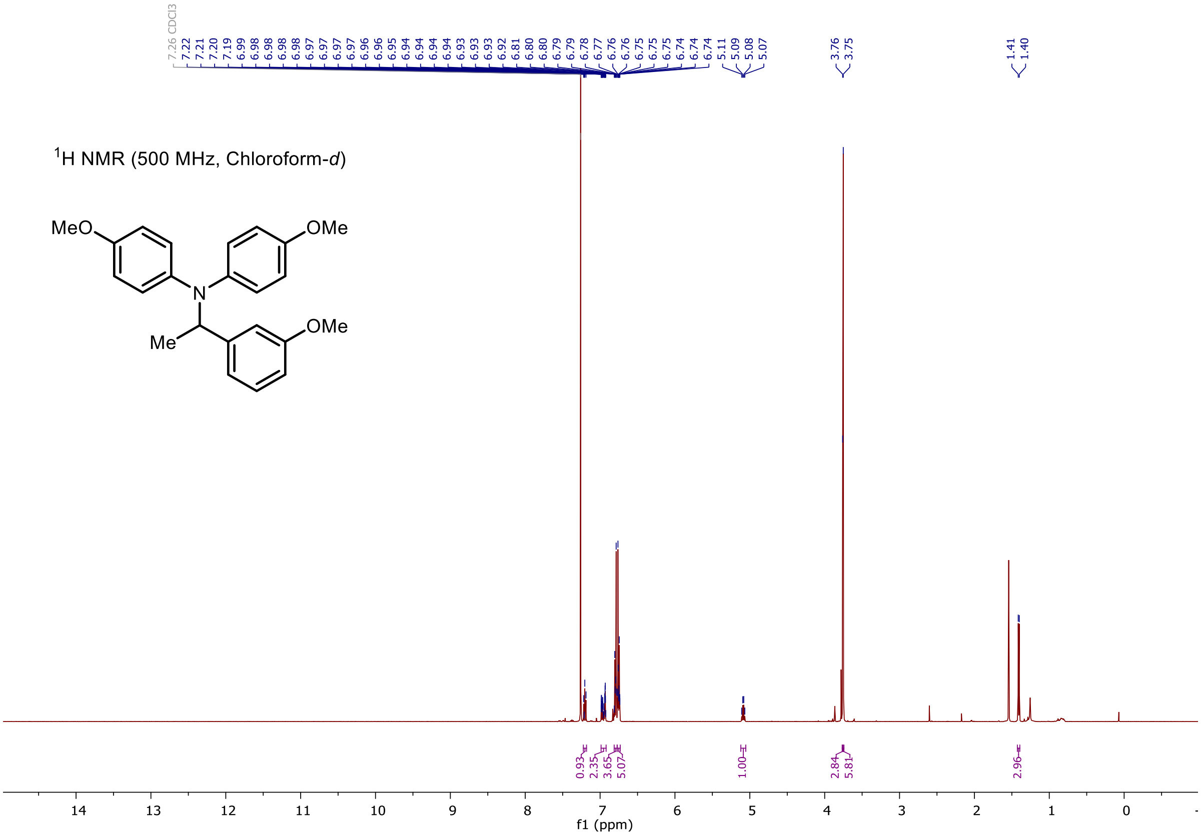

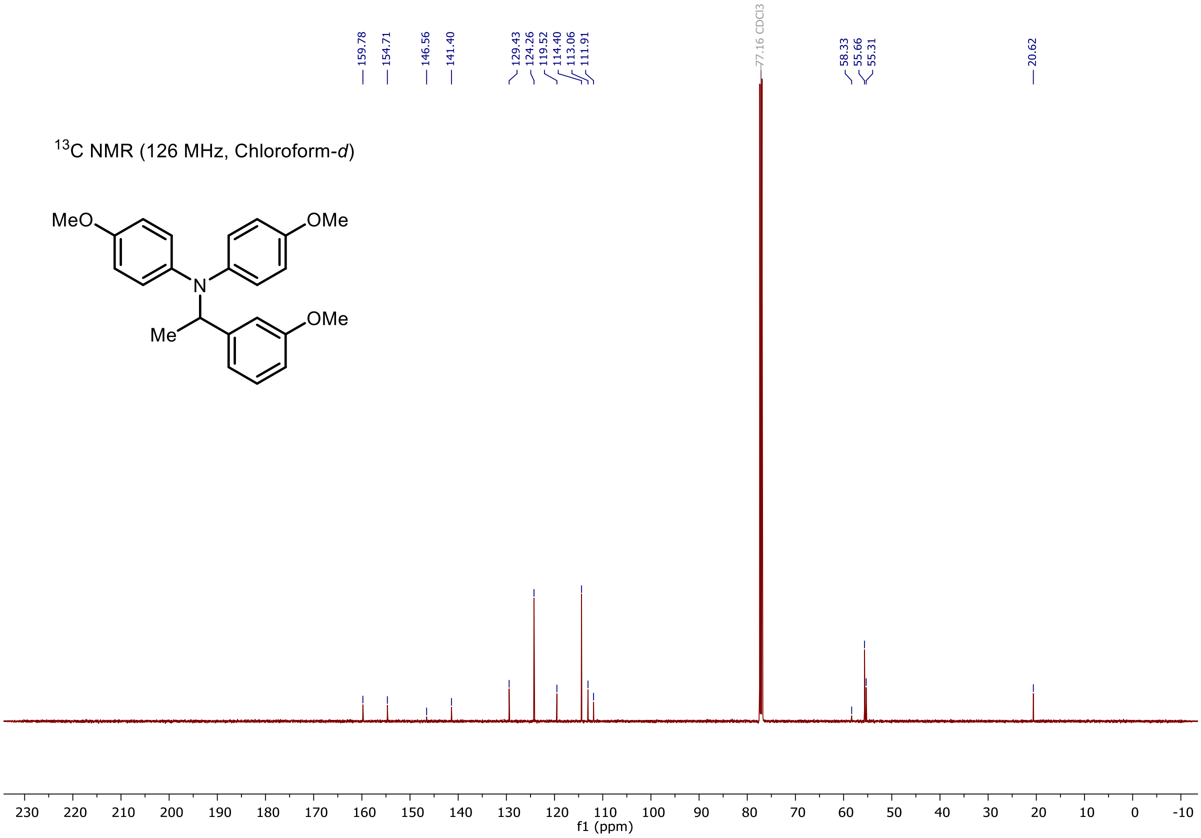

4-methoxy-N-(4-methoxyphenyl)-N-(1-(4-methoxyphenyl)ethyl)aniline

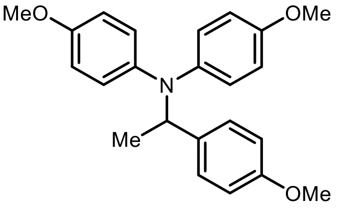

The title compound was prepared according to general procedure 3 using bis(4-methoxyphenyl)amine (500 mg, 2.18 mmol) and 1-(4-methoxyphenyl)ethan-1-ol (330 mg, 2.18 mmol). Purification by column chromatography on silica gel (eluent = 20% EtOAc in petroleum ether, 40 × 180 mm silica) gave the title compound as a brown oil (210 mg, 52%); R_f = 0.56 (eluent = 20% EtOAc in petroleum ether); vmax /cm-1 (film) 2980, 2889, 1724, 1693, 1668, 1606, 1566, 1502, 1452, 1359, 1215, 1028, 746, 683; **^{1}H NMR (500 MHz, Chloroform-*d*)** δ 7.26 – 7.23 (m, 2H), 6.83 – 6.79 (m, 2H), 6.78 – 6.73 (m, 8H), 5.12 (q, *J* = 6.9 Hz, 1H), 3.78 (s, 3H), 3.75 (s, 6H), 1.42 (d, *J* = 7.0 Hz, 3H); **^{13}C{^{1}H} NMR (126 MHz, Chloroform-*d*)** δ 158.4, 154.6, 141.4, 136.5, 128.3, 124.2, 114.4, 113.7, 57.3, 55.7, 55.4, 20.0; HRMS (ESI-TOF) calculated $[C_{23}H_{26}NO_3]^+$ $(M + H)^+$ m/z 364.1913, found 364.1912.

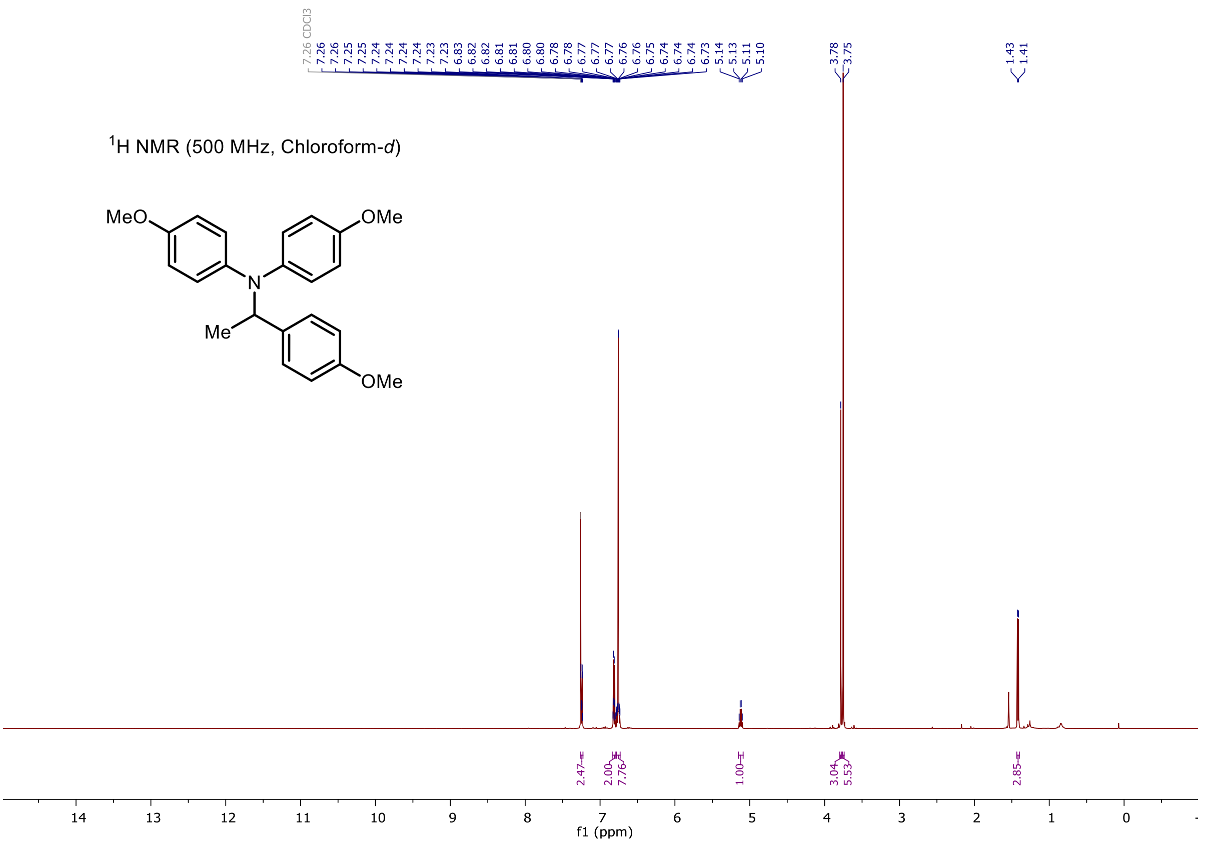
7.26 CDCl3
7.26
7.26
7.25
7.25
7.24
7.24
7.24
7.24
7.23
7.23
6.83
6.82
6.82
6.81
6.80
6.80
6.78
6.78
6.77
6.77
6.77
6.76
6.76
6.75
6.74
6.74
6.74
6.73
5.14
5.13
5.11
5.10
3.78
3.75
1.43
1.41
^{1}H NMR (500 MHz, Chloroform-*d*)
MeO
OMe
N
Me
OMe
2.47
2.00
7.76
1.00
3.04
5.53
2.85
14
13
12
11
10
9
8
7
6
5
4
3
2
1
0
f1 (ppm)

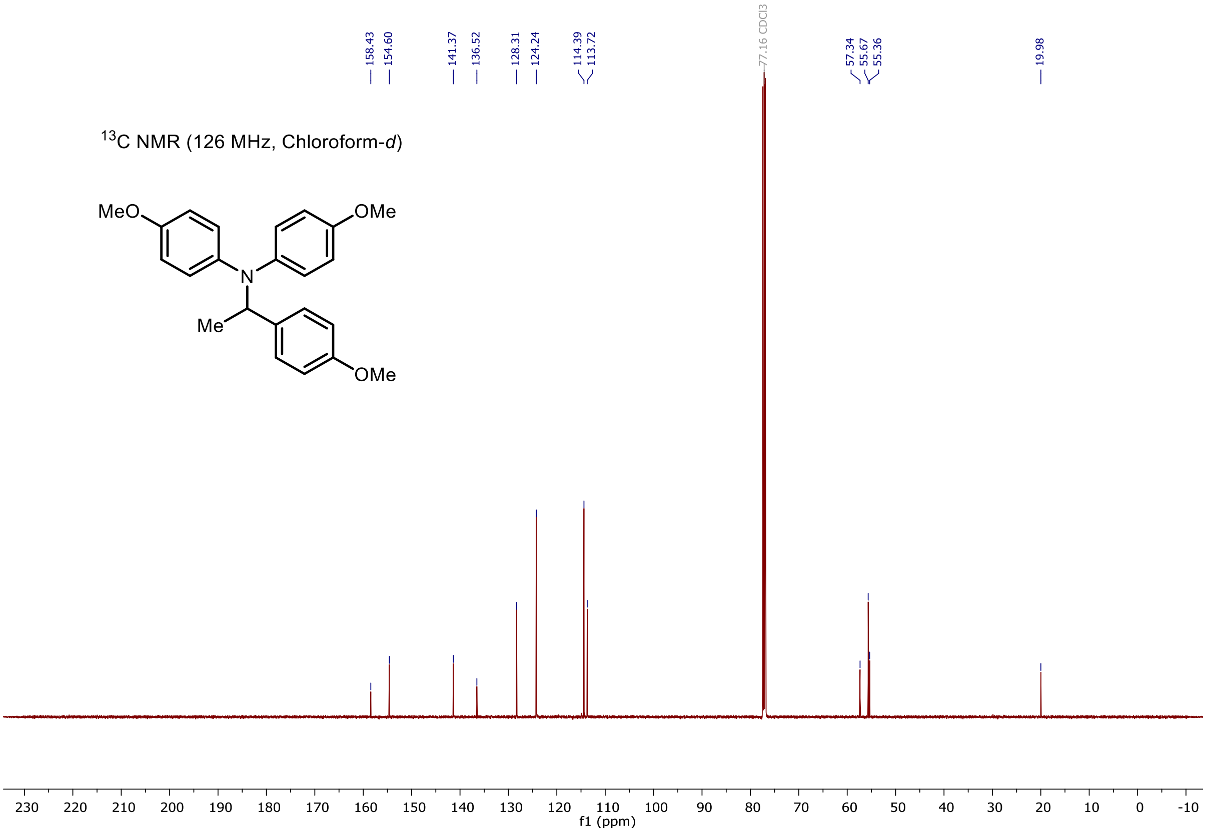
158.43
154.60
141.37
136.52
128.31
124.24
114.39
113.72
77.16 CDCl3
57.34
55.67
55.36
19.98
^{13}C NMR (126 MHz, Chloroform-*d*)
MeO
OMe
N
Me
OMe
230
220
210
200
190
180
170
160
150
140
130
120
110
100
90
80
70
60
50
40
30
20
10
0
-10
f1 (ppm)

4-methoxy-N-(4-methoxyphenyl)-N-(1-(4-(trifluoromethyl)phenyl)ethyl)aniline

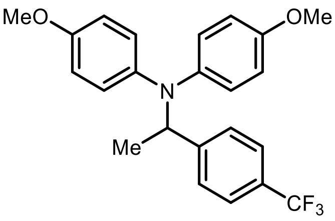

The title compound was prepared according to general procedure 3 using bis(4-methoxyphenyl)amine (500 mg, 2.18 mmol) and 1-(4-(trifluoromethyl)phenyl)ethan-1-ol (410 mg, 2.18 mmol). Purification by column chromatography on silica gel (eluent = 20% EtOAc in petroleum ether, 40 × 180 mm silica) gave the title compound as a brown oil (168 mg, 38%); R_f = 0.72 (eluent = 20% EtOAc in petroleum ether); vmax /cm-1 (film) 2970, 1508, 1465, 1327, 1240, 1159, 1126, 1035, 948, 817, 468, 412; **^{1}H NMR (500 MHz, Chloroform-*d*)** δ 7.58 – 7.47 (m, 4H), 6.81 – 6.73 (m, 8H), 5.15 (q, *J* = 7.0 Hz, 1H), 3.76 (s, 6H), 1.42 (d, *J* = 7.0 Hz, 3H); **^{13}C{^{1}H} NMR (126 MHz, Chloroform-*d*)** δ 154.9, 149.1, 141.1, 127.4, 125.5 (d, J_{C-F} = 3.8 Hz), 124.4, 119.7, 114.5, 58.1, 55.7, 20.9; **^{19}F NMR (471 MHz, Chloroform-*d*)** δ -62.33; HRMS (ESI-TOF) calculated $[C_{23}H_{23}NO_2F_3]^+$ $(M + H)^+$ m/z 402.1681, found 402.1680.

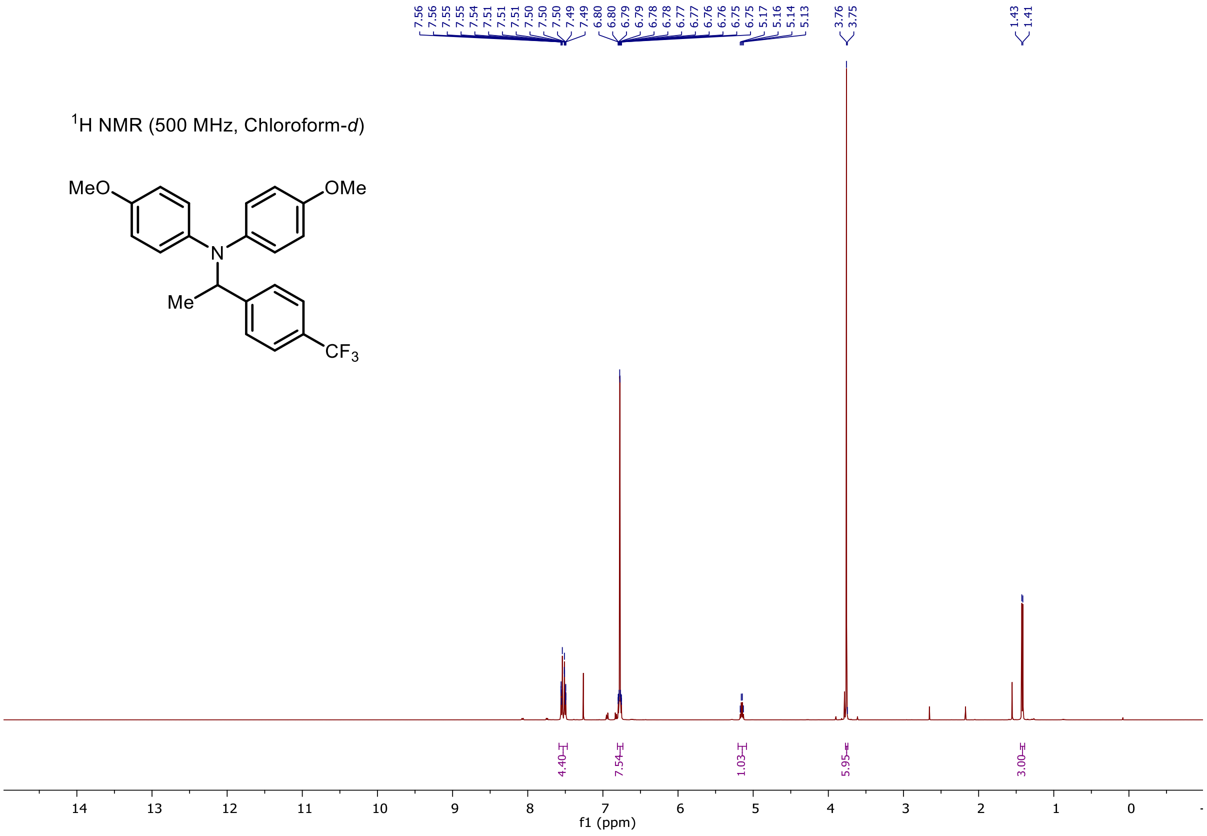

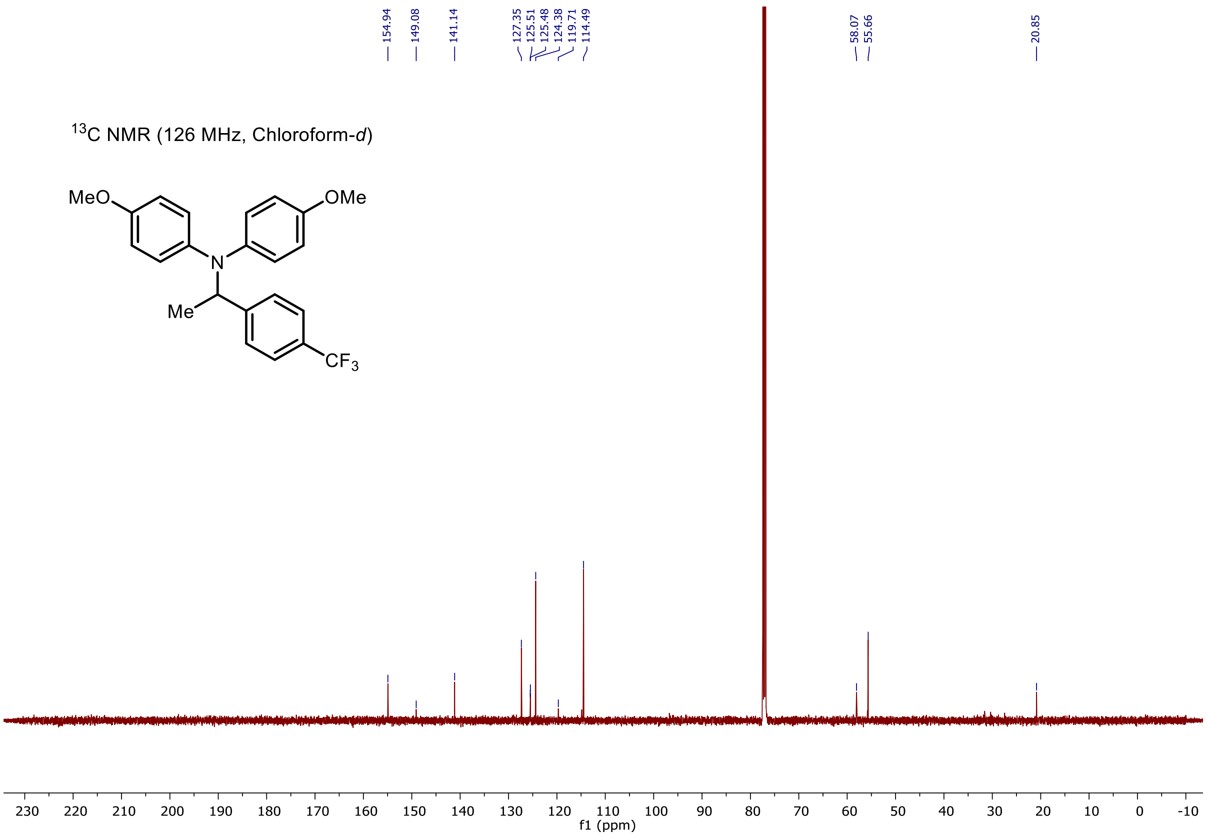
154.94
149.08
141.14
127.35
125.51
125.48
124.38
119.71
114.49
58.07
55.66
20.85
13C NMR (126 MHz, Chloroform-d)
MeO
OMe
N
Me
CF3
230 220 210 200 190 180 170 160 150 140 130 120 110 100 90 80 70 60 50 40 30 20 10 0 -10
f1 (ppm)

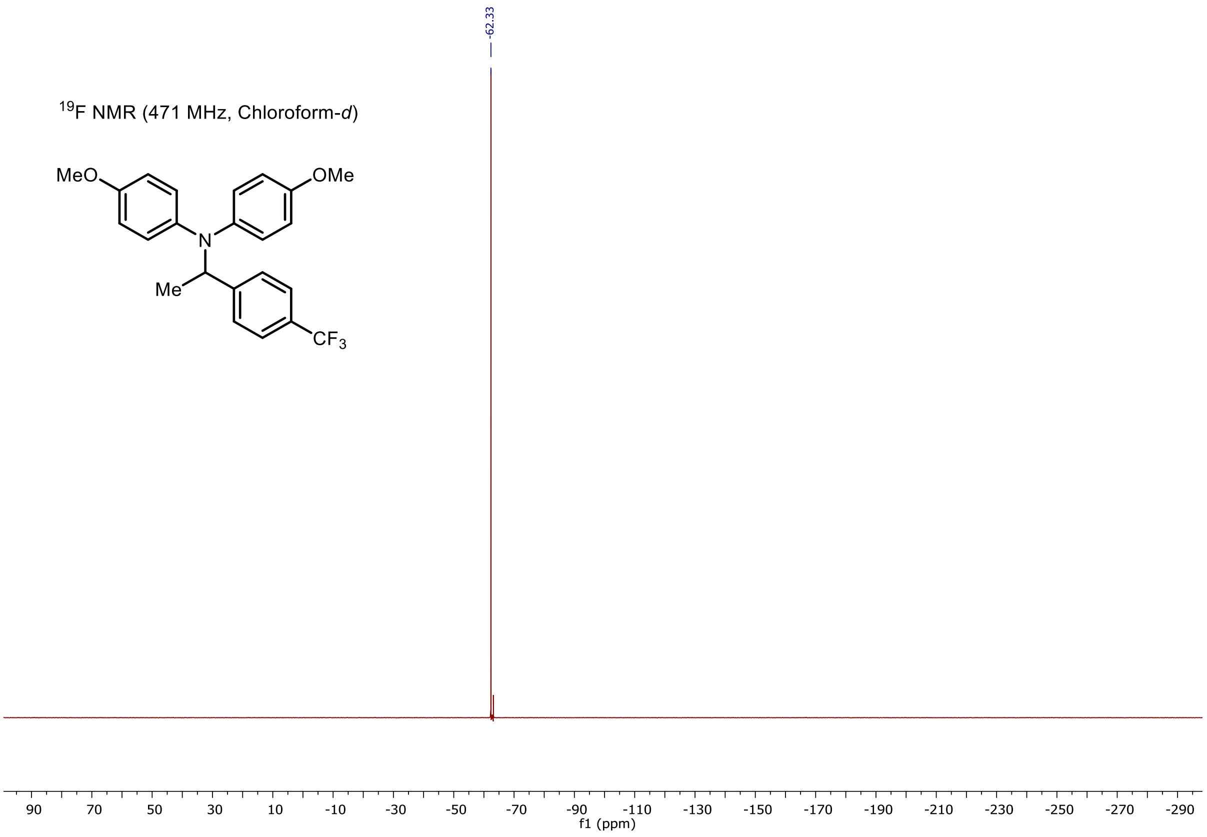
-62.33
19F NMR (471 MHz, Chloroform-d)
MeO
OMe
N
Me
CF3
90 70 50 30 10 -10 -30 -50 -70 -90 -110 -130 -150 -170 -190 -210 -230 -250 -270 -290
f1 (ppm)

N-phenyl-N-(1-phenylethyl)aniline (5)

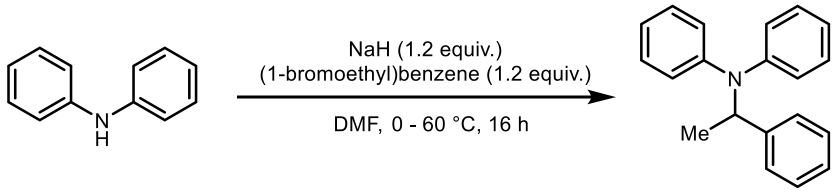

To a solution of diphenylamine (1 g, 5.9 mmol) in DMF (21 mL), NaH (60% in mineral oil) (280 mg, 7.1 mmol) was added at 0 °C. The mixture was warmed to room temperature and stirred for 1 h. Then, The mixture was cooled again to 0 °C and (1-bromoethyl)benzene (1.4 mL, 7.1 mmol) was added. After warming the mixture to room temperature, it was stirred at 60 °C for 16 h. The mixture was then cooled to room temperature and water (25 mL) and EtOAc (50 mL) were added. The aqueous layer was extracted with EtOAc (2 x 50 mL) and the combined organics were washed with brine (3 x 25 mL), dried over $MgSO_4$, filtered and concentrated under vacuo. Purification by column chromatography on silica gel (eluent = 10% EtOAc in petroleum ether, 70 × 180 mm silica) gave the title compound as a light brown solid (500 mg, 31%); mp 58-61 °C; R_f = 0.82 (eluent = 10% EtOAc in petroleum ether); **^{1}H NMR (500 MHz, Chloroform-*d*)** δ 7.41 – 7.36 (m, 2H), 7.30 (dd, *J* = 8.4, 6.8 Hz, 2H), 7.25 – 7.17 (m, 5H), 6.96 (tt, *J* = 7.3, 1.2 Hz, 2H), 6.91 – 6.85 (m, 4H), 5.32 (q, *J* = 7.0 Hz, 1H), 1.52 (d, *J* = 7.0 Hz, 3H); **^{13}C{^{1}H} NMR (126 MHz, Chloroform-*d*)** δ 147.1, 143.9, 129.1, 128.5, 127.1, 126.9, 123.1, 121.8, 57.8, 19.7. The spectroscopic data are in accordance with those described in the literature.[7]

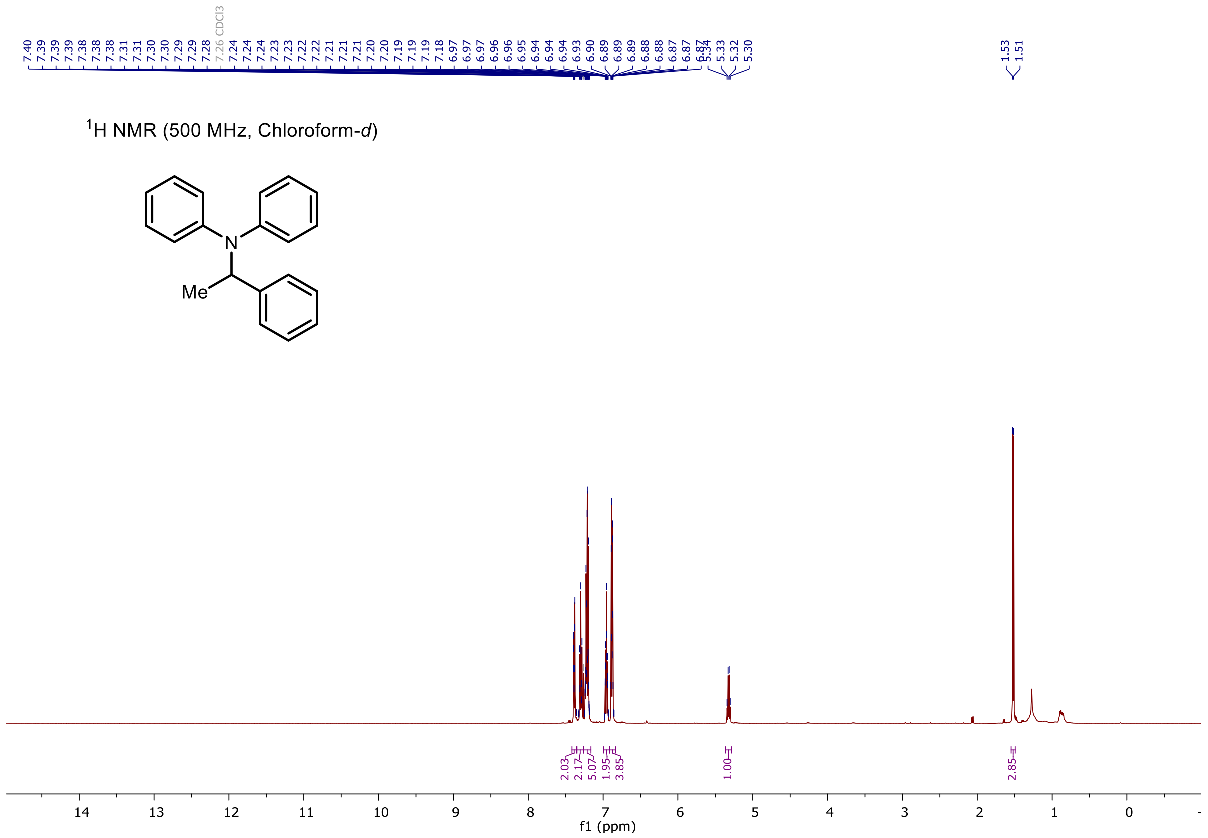

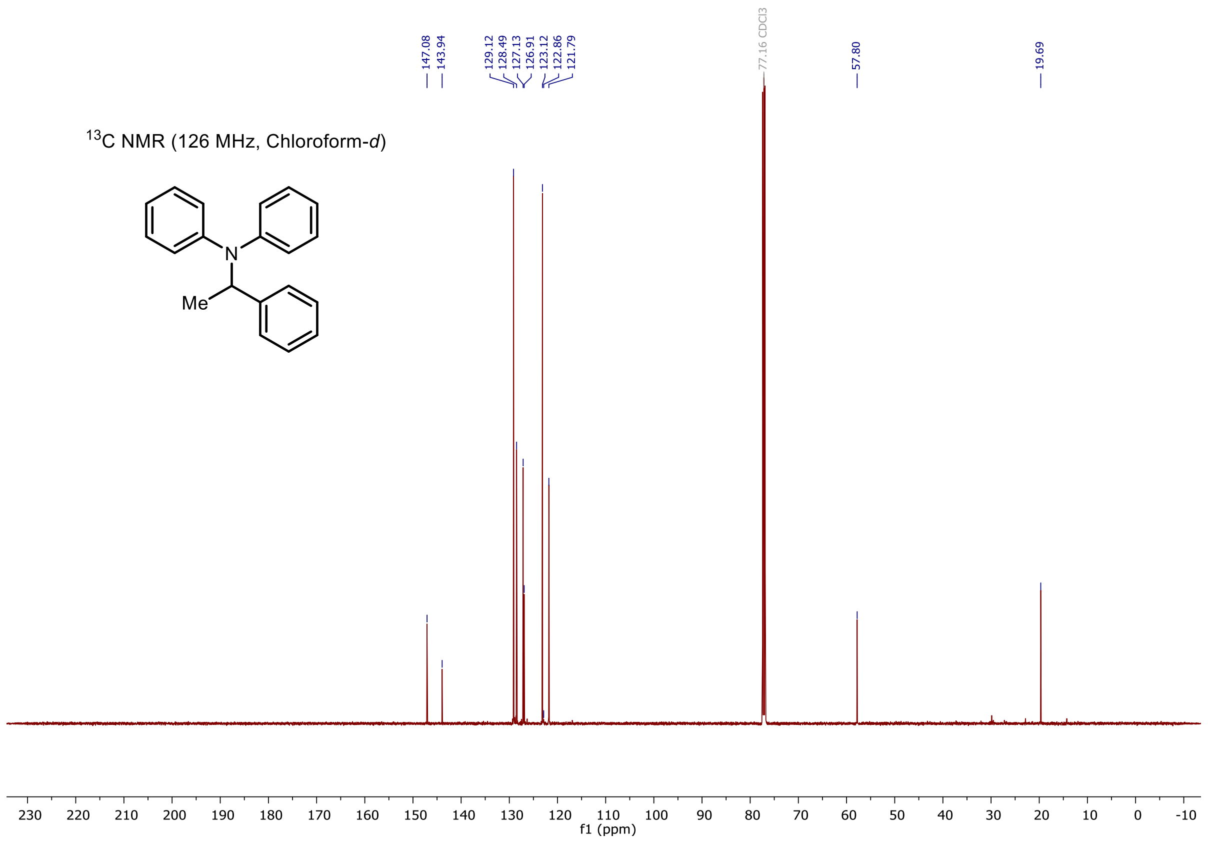

2.3. Synthesis of the indole substrates

2.3.1. General procedure 4: 1,2,5-trimethyl-1H-indole

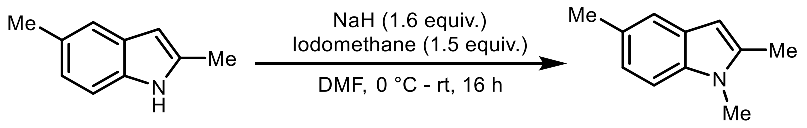

The title compound was prepared according to literature procedure.[8]To a solution of 2,5-dimethyl-1H-indole (1 g, 6.88 mmol, 1 equiv.) in DMF (27.5 mL) at 0 °C, NaH (60% in mineral oil) (440 mg, 11 mmol) was added. The mixture was warmed to room temperature and stirred for 1 h. Then, The mixture was cooled again to 0 °C and iodomethane (0.64 mL, 10.3 mmol) was added. After warming the mixture to room temperature, it was stirred for 16 h. Thereafter, water (25 mL) and EtOAc (50 mL) were added. The organic layer was separated and the aqueous layer was extracted with EtOAc (2 x 50 mL) and the combined organics were washed with brine (3 x 25 mL), dried over $MgSO_4$, filtered and concentrated under vacuo. Purification by column chromatography on silica gel (eluent = 10% EtOAc in petroleum ether, 70 × 180 mm silica) gave the title compound as a light brown solid (834 mg, 76%); mp 62-64 °C; R_f = 0.8 (eluent = 15% EtOAc in petroleum ether); vmax / cm-1 (film) 2908, 1508, 1489, 1396, 786; **^{1}H NMR (500 MHz, Chloroform-*d*)** δ 7.31 (h, *J* = 2.0, 1.4 Hz, 1H), 7.15 (dd, *J* = 9.1, 3.4 Hz, 1H), 6.98 (dt, *J* = 9.3, 2.8 Hz, 1H), 6.17 (ddt, *J* = 3.7, 1.7, 0.9 Hz, 1H), 3.64 (d, *J* = 1.3 Hz, 3H), 2.46 – 2.43 (m, 3H), 2.43 – 2.40 (m, 3H); **^{13}C{^{1}H} NMR (126 MHz, Chloroform-*d*)** δ 136.9, 135.9, 128.5, 128.3, 122.0, 119.5, 108.5, 99.1, 29.5, 21.6, 12.9; HRMS (ESI-TOF) calculated $[C_{11}H_{14}N]^+$ $(M + H)^+$ m/z 160.1126, found 160.1128.

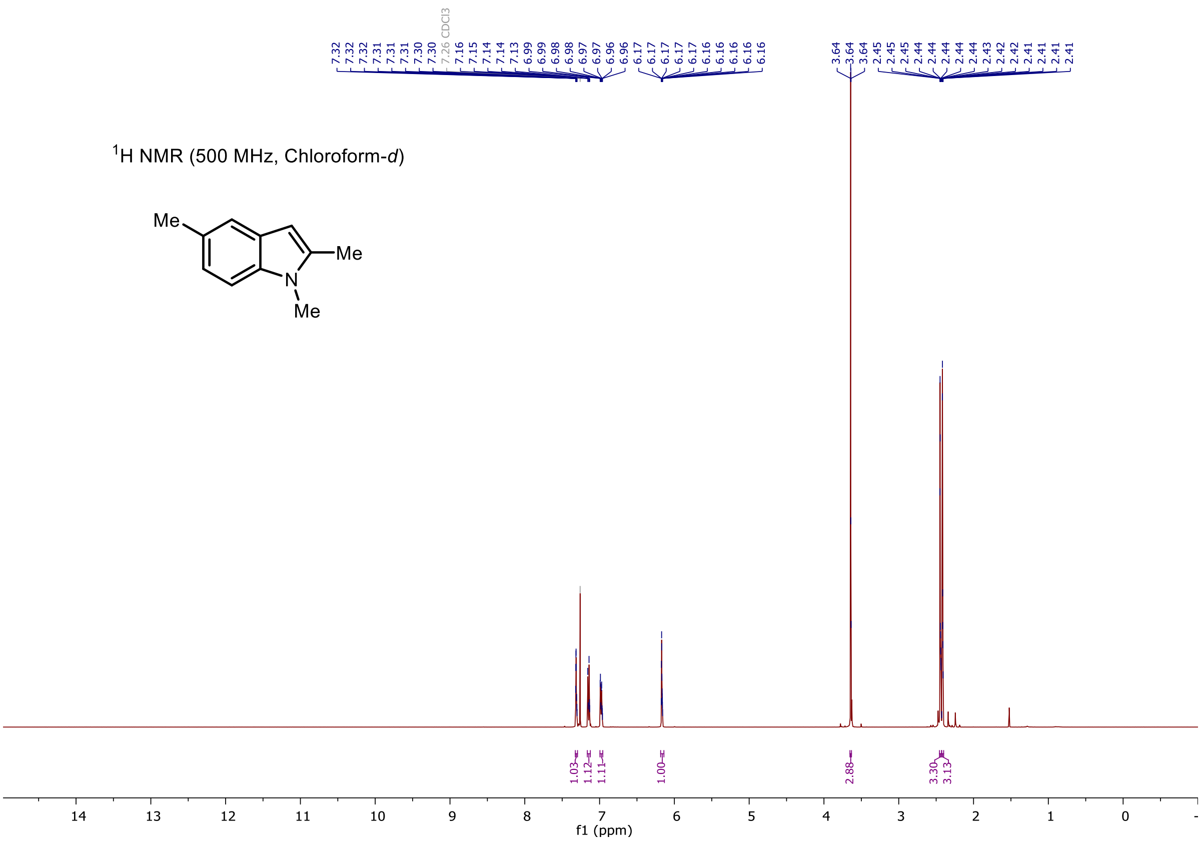

7.32
7.32
7.32
7.31
7.31
7.30
7.30
7.26 CDCl3
7.16
7.15
7.14
7.14
7.13
6.99
6.99
6.98
6.98
6.97
6.97
6.96
6.96
6.17
6.17
6.17
6.17
6.16
6.16
6.16
6.16
3.64
3.64
3.64
2.45
2.45
2.44
2.44
2.44
2.44
2.43
2.42
2.42
2.41
2.41
2.41
2.41
^{1}H NMR (500 MHz, Chloroform-*d*)
Me
Me
N
Me
1.03
1.12
1.11
1.00
2.88
3.30
3.13
14
13
12
11
10
9
8
7
6
5
4
3
2
1
0
f1 (ppm)

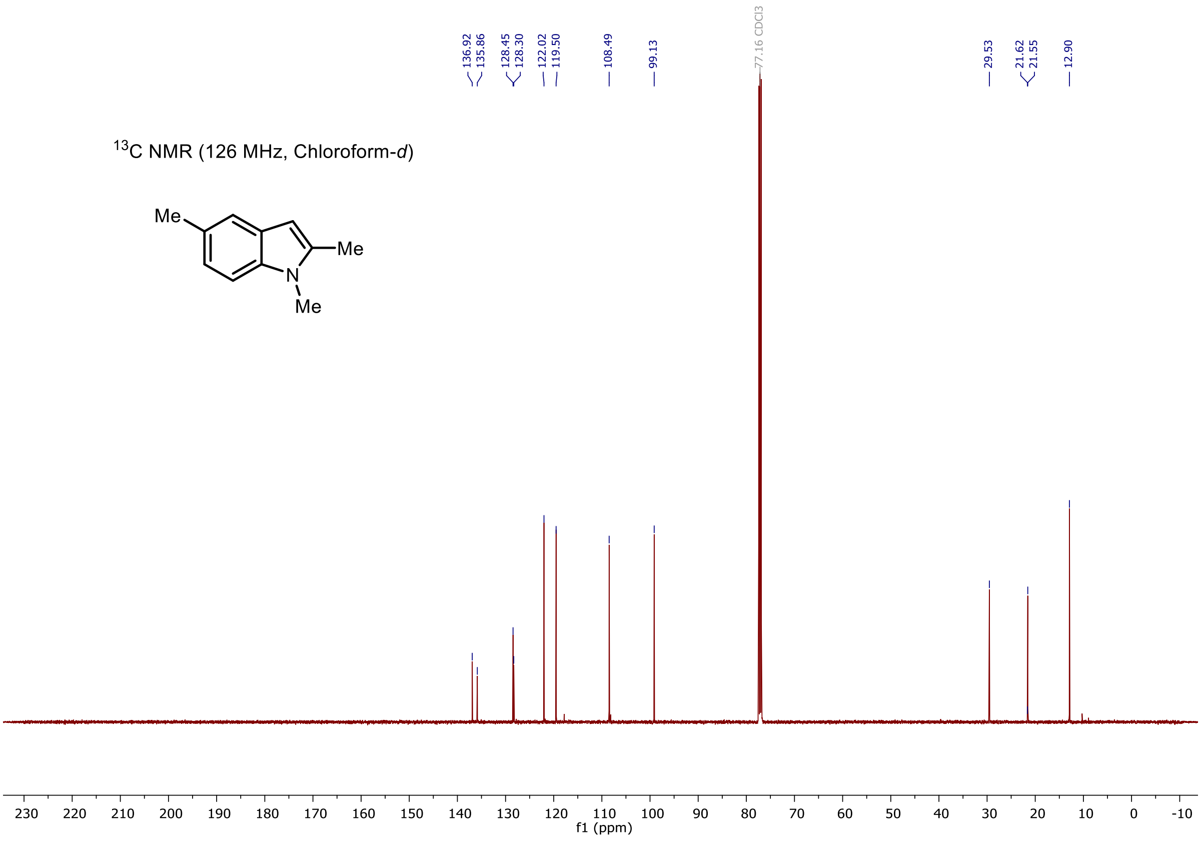

136.92
135.86
128.45
128.30
122.02
119.50
108.49
99.13
77.16 CDCl3
29.53
21.62
21.55
12.90
^{13}C NMR (126 MHz, Chloroform-*d*)
Me
Me
N
Me
230
220
210
200
190
180
170
160
150
140
130
120
110
100
90
80
70
60
50
40
30
20
10
0
-10
f1 (ppm)

5-bromo-1,2-dimethyl-1H-indole

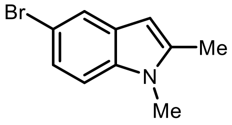

The title compound was prepared according to general procedure 4 using 5-bromo-2-methylindole (700 mg, 3.3 mmol). Purification by column chromatography on silica gel (eluent = 20% EtOAc in petroleum ether, 40 × 180 mm silica) gave the title compound as an off-white solid (637 mg, 86%); mp 76-79 °C; R_f = 0.6 (eluent = 20% EtOAc in petroleum ether); vmax / cm-1 (film) 2908, 1566, 1546, 1469, 1394, 1379, 1327, 1269, 1232, 1178, 1145, 1103, 1049, 908, 875, 779, 740; **^{1}H NMR (500 MHz, Chloroform-*d*)** δ 7.62 (d, *J* = 1.9 Hz, 1H), 7.21 (dd, *J* = 8.6, 1.9 Hz, 1H), 7.11 (d, *J* = 8.6 Hz, 1H), 6.18 (q, *J* = 1.0 Hz, 1H), 3.64 (d, *J* = 0.7 Hz, 3H), 2.42 (d, *J* = 0.8 Hz, 3H); **^{13}C{^{1}H} NMR (126 MHz, Chloroform-*d*)** δ 138.3, 136.1, 129.6, 123.2, 122.1, 112.6, 110.2, 99.3, 29.7, 13.0; HRMS (ESI-TOF) calculated $[C_{10}H_{11}NBr]^+$ $(M + H)^+$ m/z 224.0075, found 224.007.

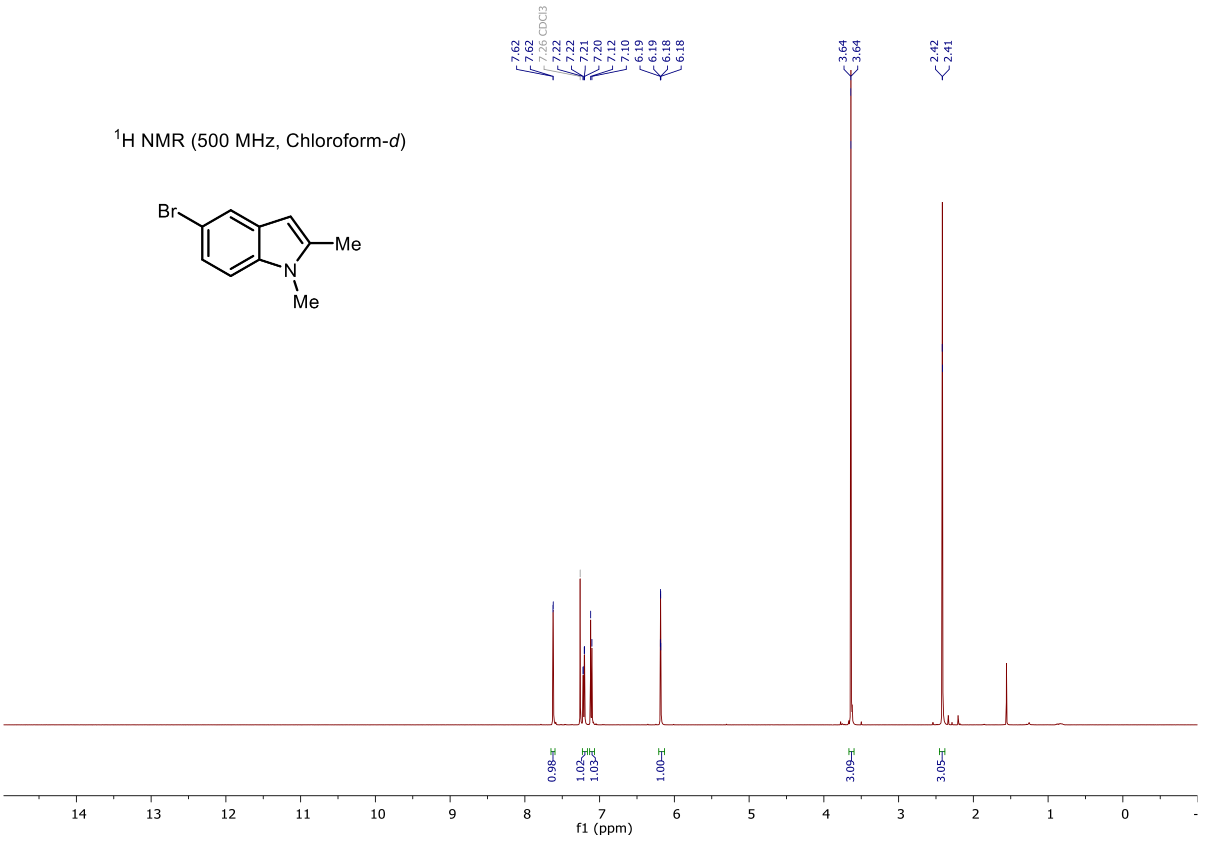

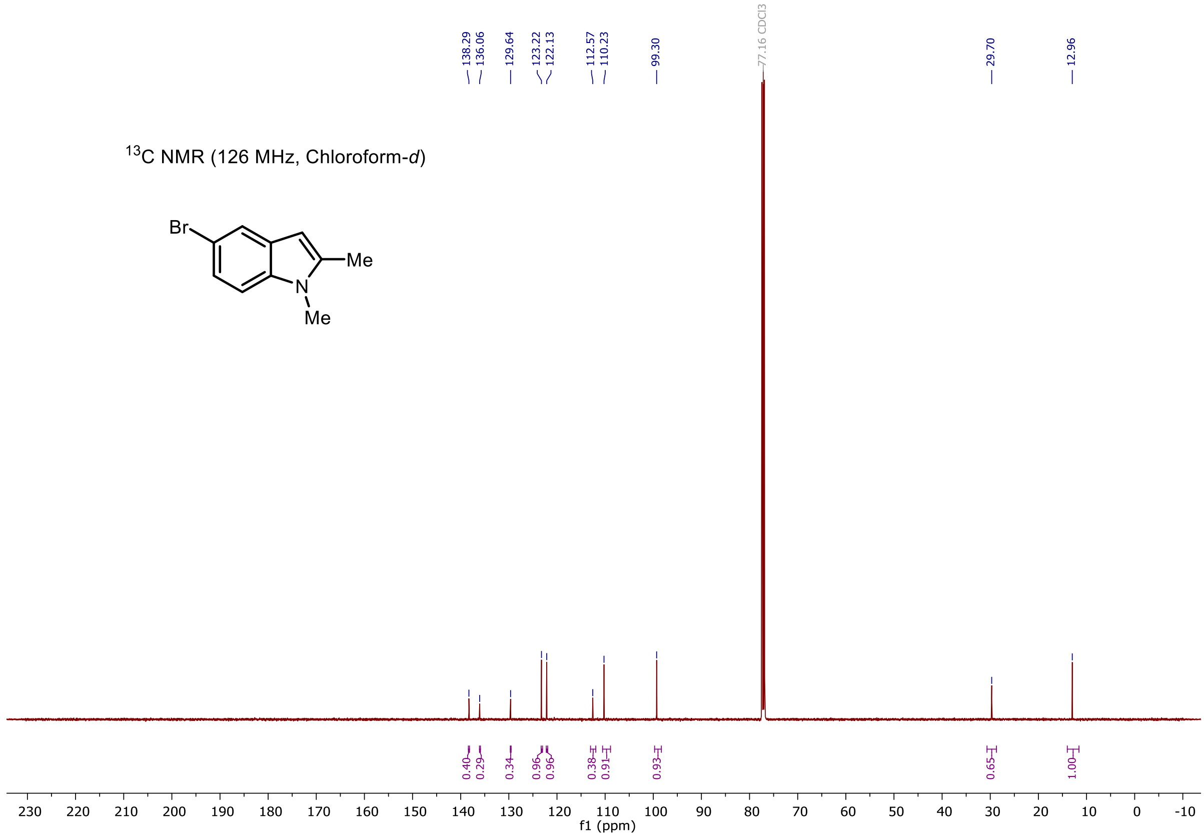

5-chloro-1,2-dimethyl-1H-indole

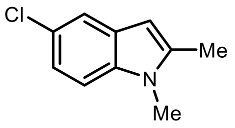

The title compound was prepared according to general procedure 4 using 5-chloro-2-methylindole (460 mg, 2.8 mmol). Purification by column chromatography on silica gel (eluent = 20% EtOAc in petroleum ether, 40 x 180 mm silica) gave the title compound as an off-white solid (263 mg, 52%); mp 52-55 °C (Lit. 56-58 °C);[9] R_f= 0.72 (eluent = 20% EtOAc in petroleum ether); **^{1}H NMR (500 MHz, Chloroform-*d*)** δ 7.47 (dd, *J* = 2.1, 0.5 Hz, 1H), 7.15 (dt, *J* = 8.6, 0.7 Hz, 1H), 7.08 (dd, *J* = 8.6, 2.0 Hz, 1H), 6.19 (p, *J* = 0.9 Hz, 1H), 3.64 (s, 3H), 2,41 (d, *J* = 1.0 Hz, 3H); **^{13}C{^{1}H} NMR (126 MHz, Chloroform-*d*)** δ 138.4, 135.9, 129.0, 125.0, 120.7, 119.1, 109.7, 99.5, 29.7, 13.0.

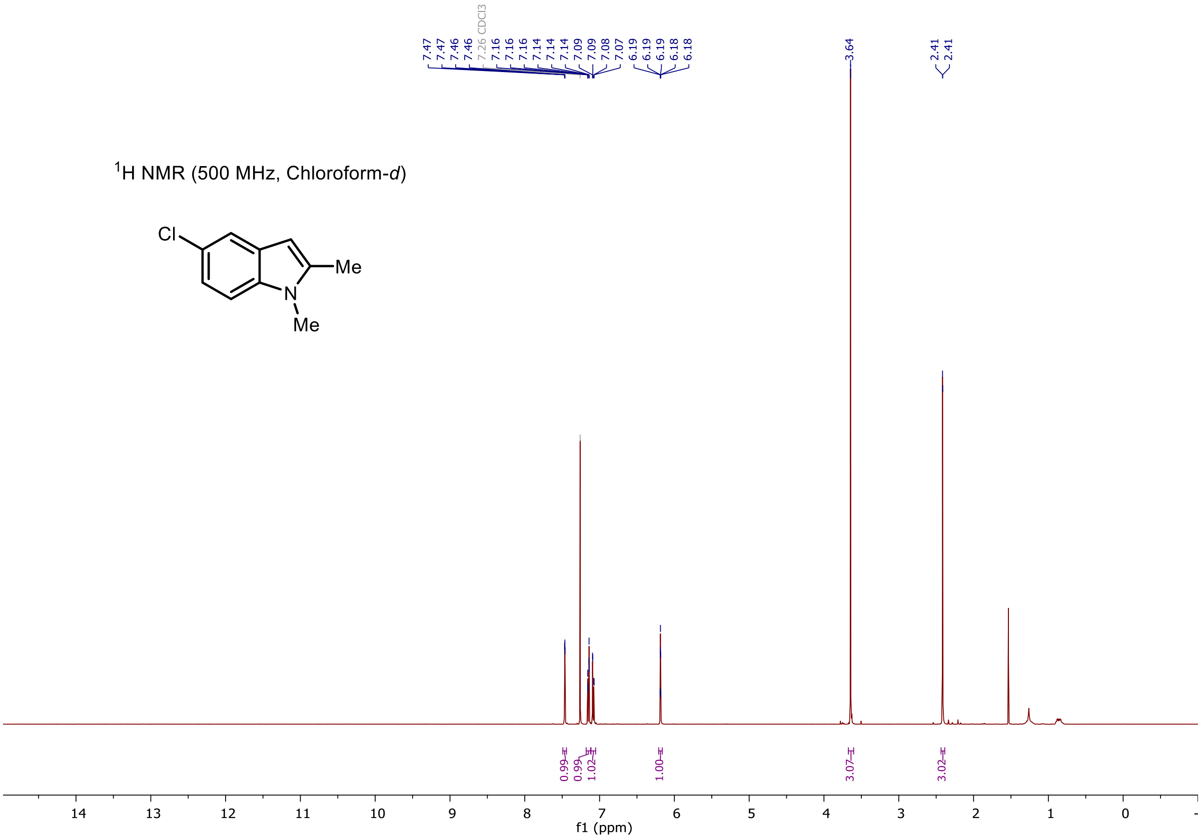

^{1}H NMR (500 MHz, Chloroform-*d*)
Cl
Me
N
Me
7.47
7.47
7.46
7.46
7.26 CDCl3
7.16
7.16
7.16
7.16
7.14
7.14
7.14
7.09
7.09
7.08
7.07
6.19
6.19
6.19
6.18
6.18
3.64
2.41
2.41
0.99
0.99
1.02
1.00
3.07
3.02
f1 (ppm)

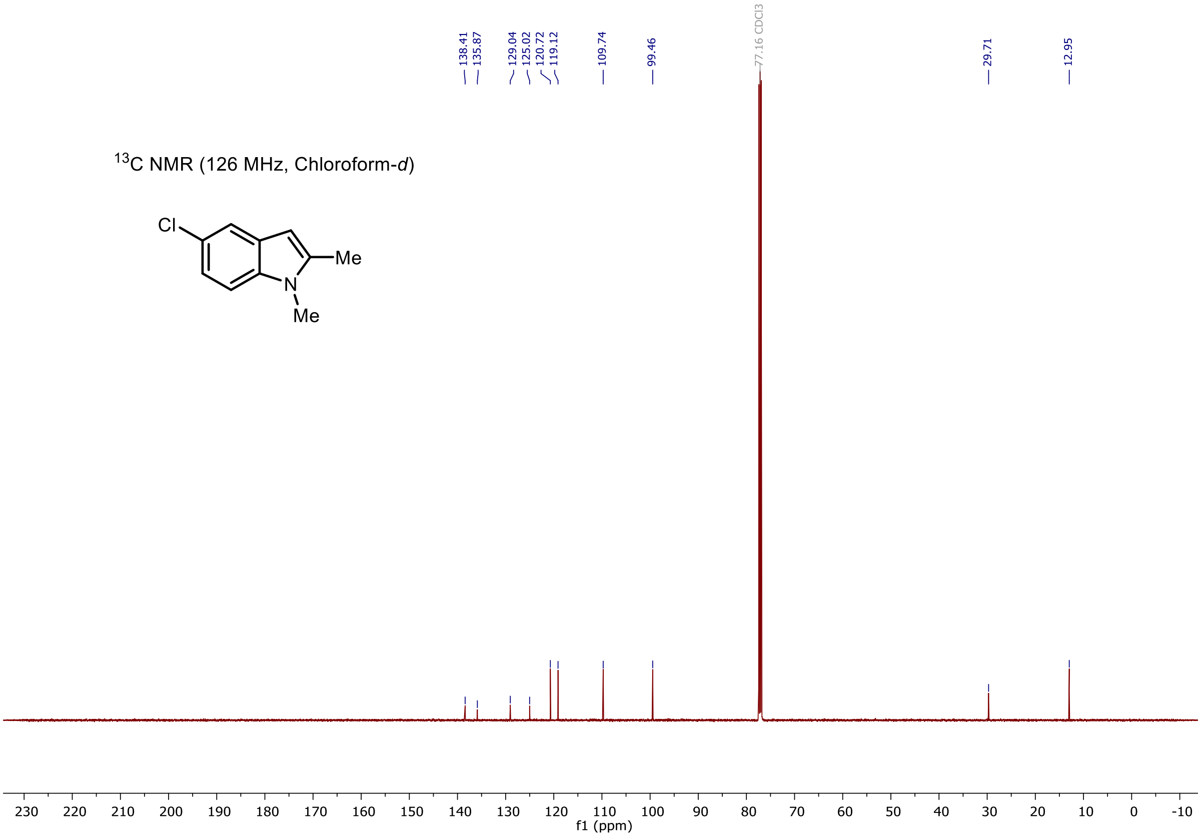

^{13}C NMR (126 MHz, Chloroform-*d*)
Cl
Me
N
Me
138.41
135.87
129.04
125.02
120.72
119.12
109.74
99.46
77.16 CDCl3
29.71
12.95
f1 (ppm)

5-methoxy-1,2-dimethyl-1H-indole

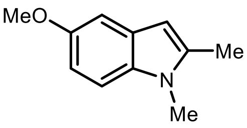

The title compound was prepared according to general procedure 4 using 5-methoxy-2-methylindole (250 mg, 1.6 mmol). Purification by column chromatography on silica gel (eluent = 10% EtOAc in petroleum ether, 40 × 180 mm silica) gave the title compound as a dark red oil (172 mg, 61%); R_f = 0.68 (eluent = 20% EtOAc in petroleum ether); **^{1}H NMR (500 MHz, Chloroform-*d*)** δ 7.16 – 7.12 (m, 1H), 7.01 (d, *J* = 2.5 Hz, 1H), 6.81 (dd, *J* = 8.8, 2.5 Hz, 1H), 6.18 – 6.16 (m, 1H), 3.85 (s, 3H), 3.63 (d, *J* = 0.4 Hz, 3H), 2.41 (d, *J* = 0.8 Hz, 3H); **^{13}C{^{1}H} NMR (126 MHz, Chloroform-*d*)** δ 154.1, 137.5, 132.8, 128.3, 110.3, 109.4, 102.1, 99.4, 56.1, 29.6, 12.9. The spectroscopic data are in accordance with those described in the literature.[8]

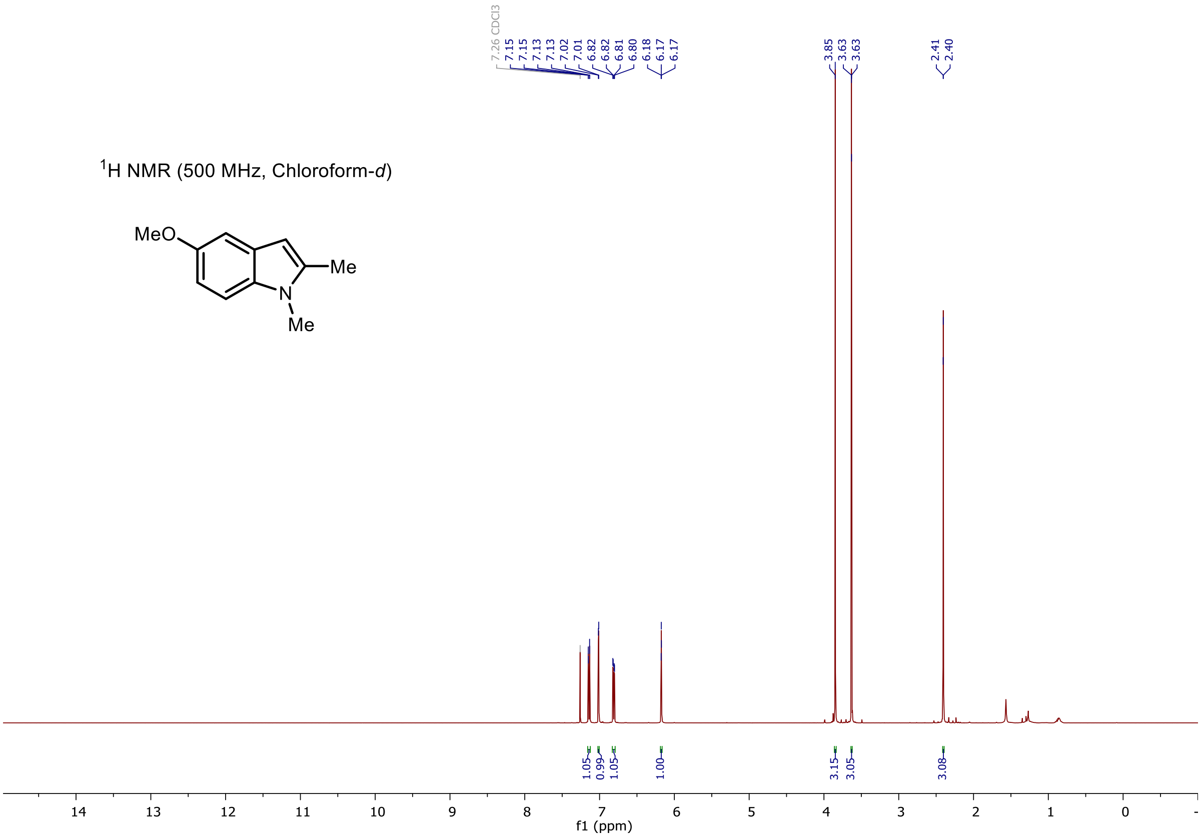

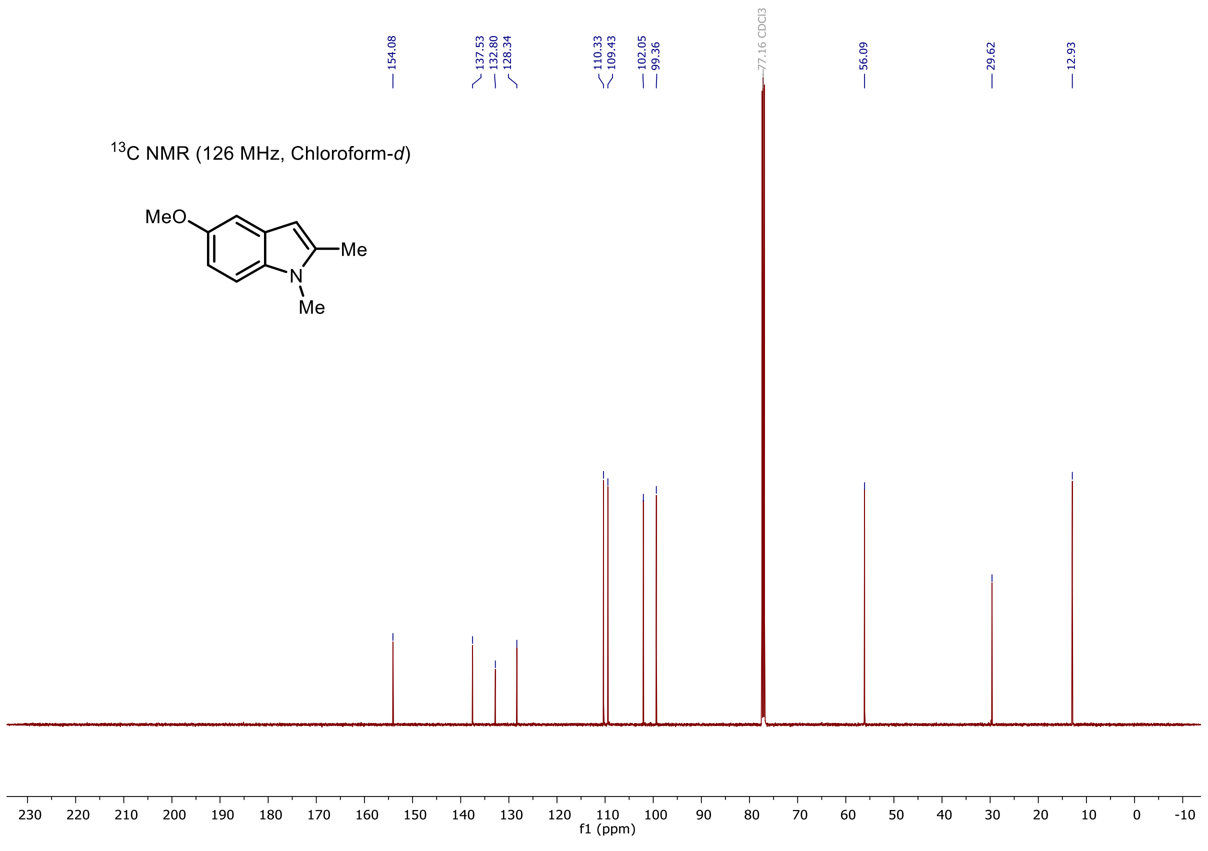

1,2-dimethyl-5-nitro-1H-indole

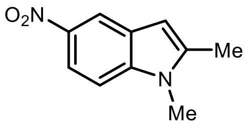

The title compound was prepared according to general procedure 4 using 2-methyl-5-nitroindole (500 mg, 2.18 mmol). Purification by column chromatography on silica gel (eluent = 50% EtOAc in petroleum ether, 40 × 180 mm silica) gave the title compound as a yellow solid (334 mg, 63%); mp 122-126 °C (Lit 128-130 °C);[10] R_f = 0.71 (eluent = 50% EtOAc in petroleum ether); **^{1}H NMR (500 MHz, Chloroform-*d*)** δ 8.46 (d, *J* = 2.3 Hz, 1H), 8.06 (dd, *J* = 9.0, 2.3 Hz, 1H), 7.28 – 7.23 (m, 1H), 6.42 (q, *J* = 1.0 Hz, 1H), 3.72 (d, *J* = 0.6 Hz, 3H), 2.46 (d, *J* = 1.0 Hz, 3H); **^{13}C{^{1}H} NMR (126 MHz, Chloroform-*d*)** δ 141.5, 140.6, 140.3, 127.2, 116.9, 116.5, 108.6, 102.3, 30.1, 13.1.

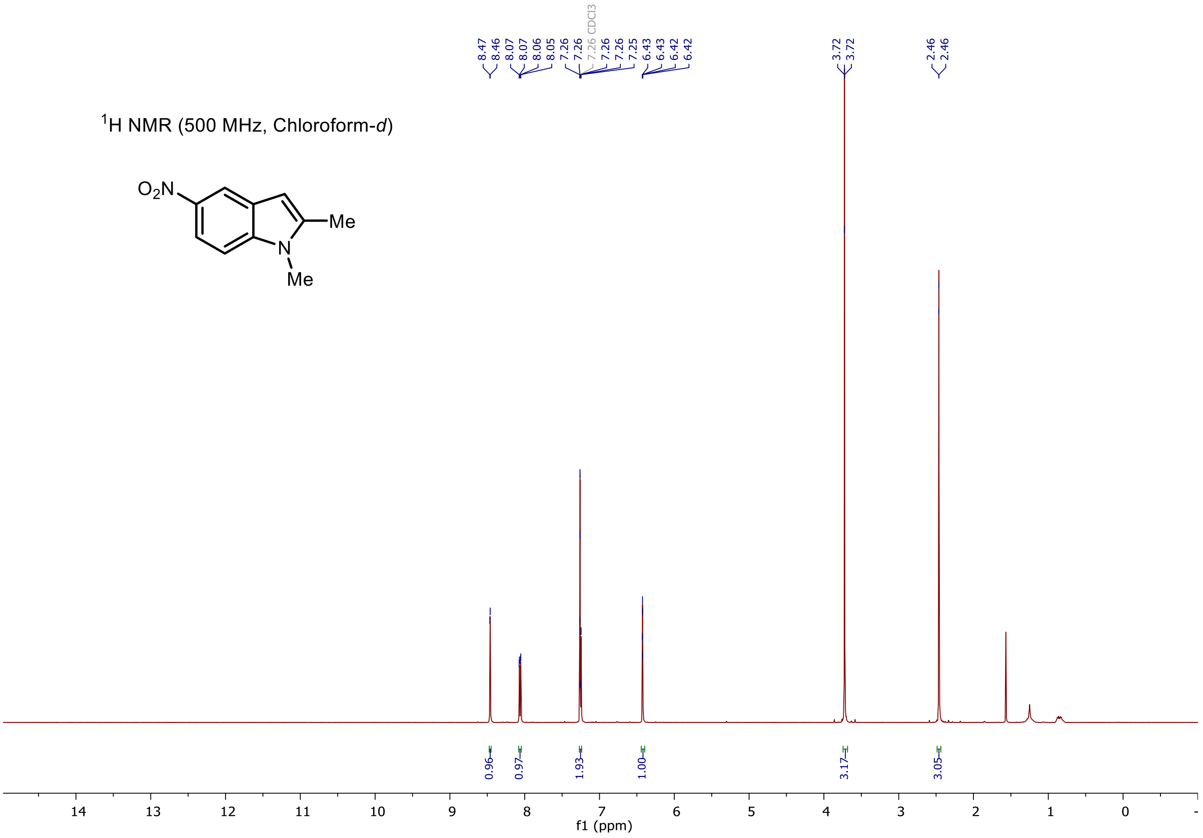

^{1}H NMR (500 MHz, Chloroform-d)
O_2N
Me
N
Me
8.47
8.46
8.07
8.07
8.06
8.05
7.26
7.26 CDCl3
7.26
7.26
7.25
6.43
6.43
6.42
6.42
3.72
3.72
2.46
2.46
0.96
0.97
1.93
1.00
3.17
3.05
f1 (ppm)

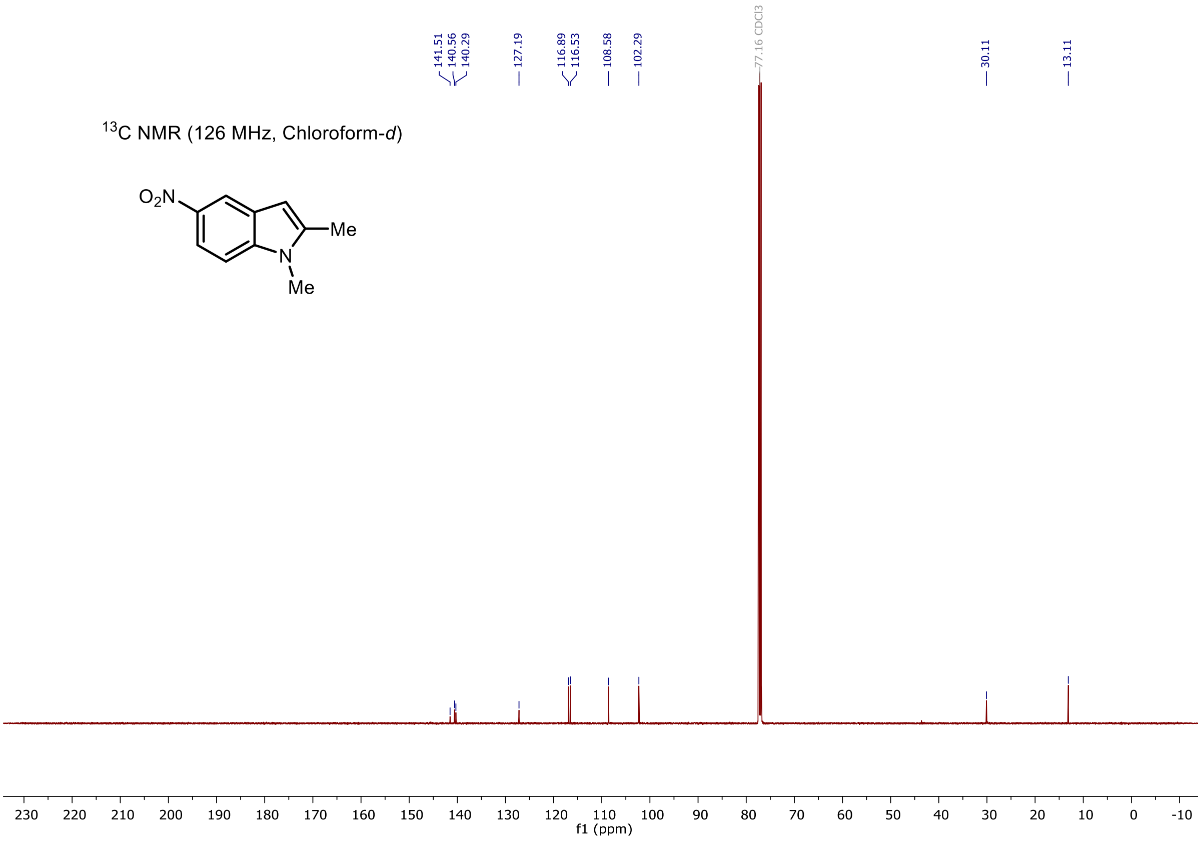

^{13}C NMR (126 MHz, Chloroform-d)
O_2N
Me
N
Me
141.51
140.56
140.29
127.19
116.89
116.53
108.58
102.29
77.16 CDCl3
30.11
13.11
f1 (ppm)

(4-chlorophenyl)(5-methoxy-2-methyl-1H-indol-1-yl)methanone

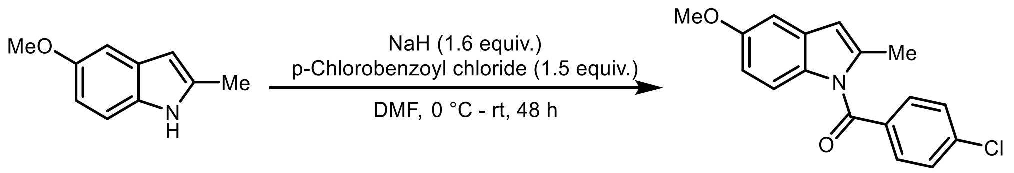

To a solution of 5-methoxy-2-methylindole (500 mg, 3.1 mmol, 1 equiv.) in DMF (12 mL) at 0 °C, NaH (60% in mineral oil) (200 mg, 5 mmol) was added. The mixture was warmed to room temperature and stirred for 1 h. Then, The mixture was cooled again to 0 °C and p-chlorobenzoyl chloride (0.6 mL, 4.7 mmol) was added. After warming the mixture to room temperature, it was stirred for 48 h. Thereafter, water (13 mL) and EtOAc (25 mL) were added. The organic layer was separated and the aqueous layer was extracted with EtOAc (2 x 25 mL) and the combined organics were washed with brine (3 x 13 mL), dried over $MgSO_4$, filtered and concentrated under vacuo. Purification by column chromatography on silica gel (eluent = 15% EtOAc in petroleum ether, 40 × 180 mm silica) gave the title compound as an off-white solid (572 mg, 62%); mp 60-63°C; R_f = 0.82 (eluent = 15% EtOAc in petroleum ether); vmax /cm-1 (film) 2981, 2831, 1680, 1591, 1543, 1477, 1367, 1317, 1205, 1174, 1149, 1089, 972, 908, 860; **^{1}H NMR (500 MHz, Chloroform-*d*)** δ 7.67 – 7.63 (m, 2H), 7.49 – 7.45 (m, 2H), 6.94 (d, *J* = 2.5 Hz, 1H), 6.90 (dt, *J* = 9.0, 0.6 Hz, 1H), 6.65 (dd, *J* = 9.0, 2.6 Hz, 1H), 6.36 (p, *J* = 1.1 Hz, 1H), 3.82 (s, 3H), 2.40 (d, *J* = 1.1 Hz, 3H); **^{13}C{^{1}H} NMR (126 MHz, Chloroform-*d*)** δ 168.6, 156.1, 139.3, 138.7, 134.1, 131.8, 131.2, 130.7, 129.2, 115.2, 111.4, 109.1, 102.9, 55.8, 16.1; HRMS (CI-QUADRUPOLE) calculated $[C_{17}H_{14}N^{35}O_2Cl]^+$ $(M)^+$ m/z 299.0708, found 299.0709.

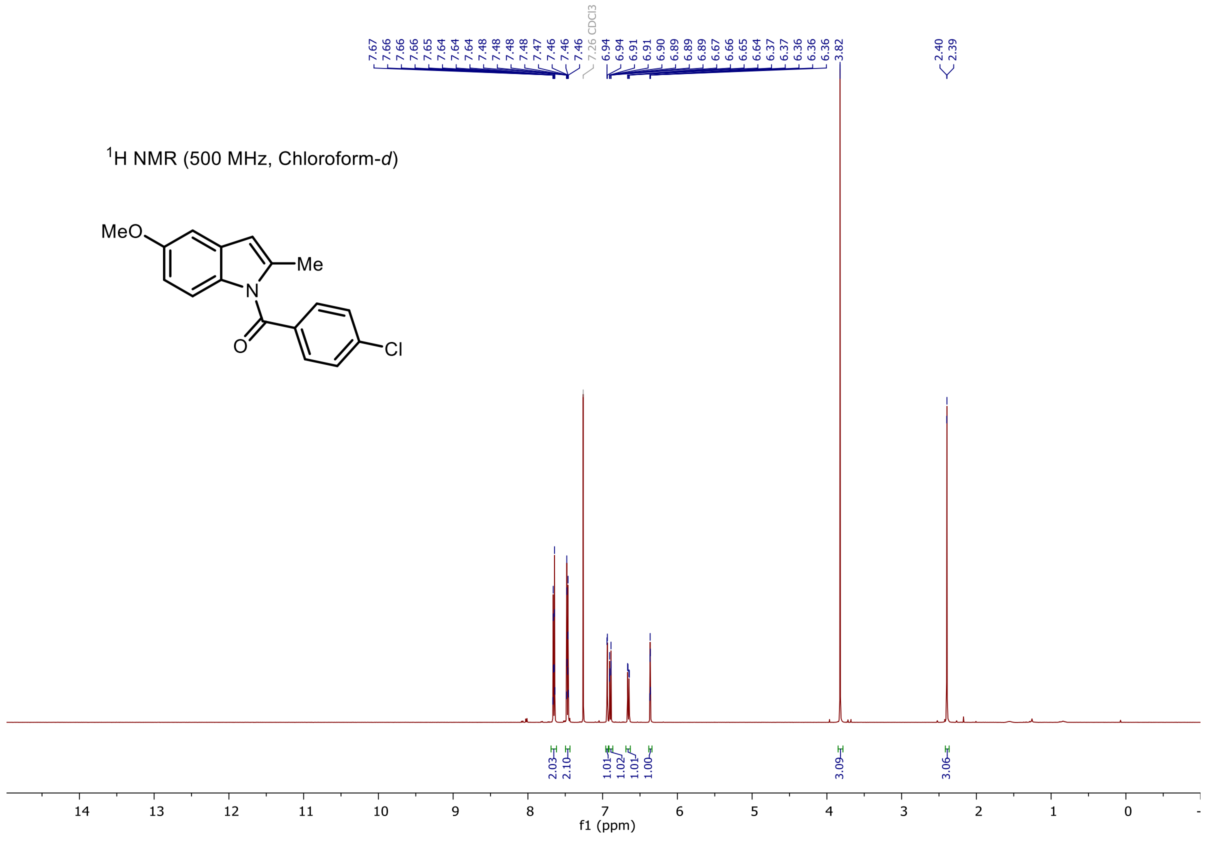

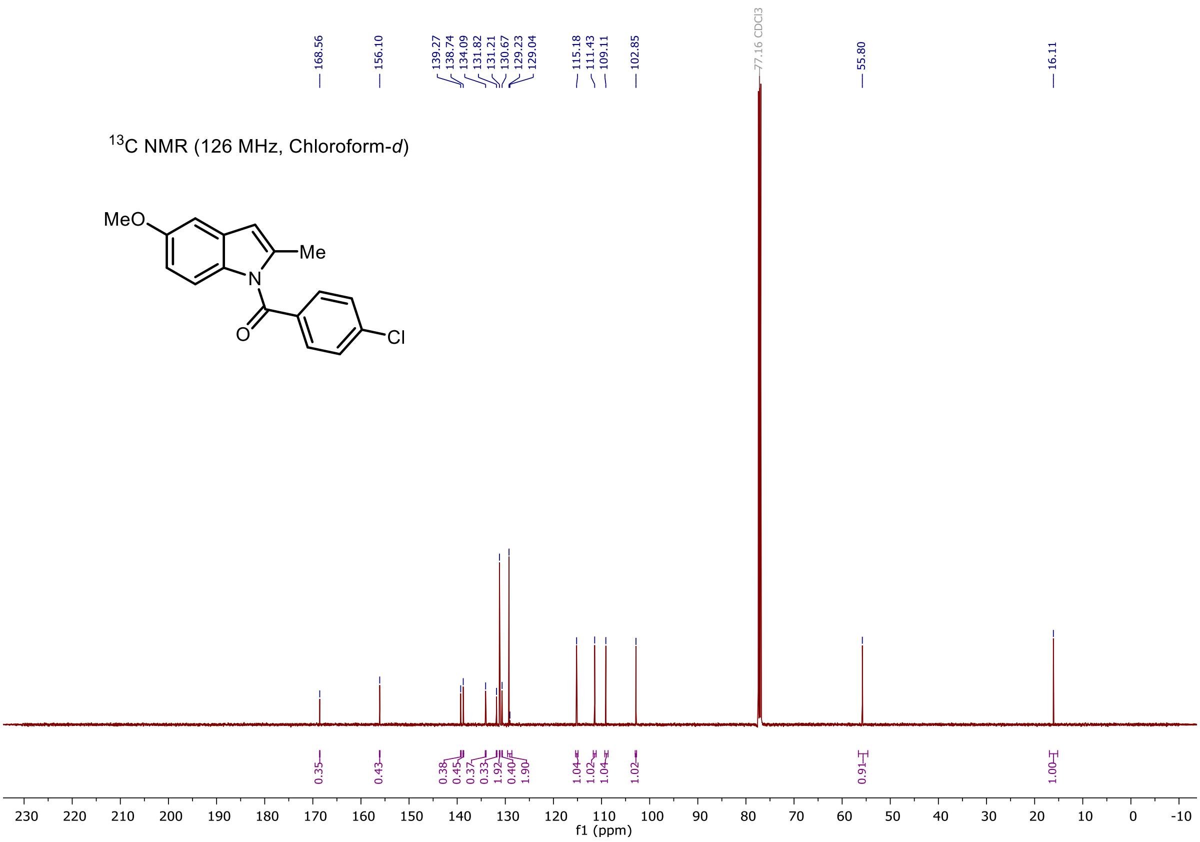

6-methoxy-2-methyl-1H-indole

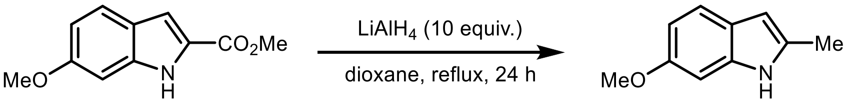

The title compound was prepared according to literature procedure.[11] To a stirred suspension of lithium aluminium hydride (1 g, 26 mmol) in anhydrous 1,4-dioxane (8 mL), 6-methoxy-1H-indole-2-carboxylic acid (500 mg, 2.6 mmol) was added. The mixture was refluxed for 24 h. The mixture was then cooled to 0 °C , quenched with water and extracted with EtOAc (3 x 26 mL). The organics were passed through a pad of celite and washed with water (1 x 26 mL) and brine (1 x 26 mL), dried over $MgSO_4$, filtered, and concentrated in vacuo. Purification by column chromatography on silica gel (eluent = 40% EtOAc in petroleum ether, 40 × 180 mm silica) gave the title compound as a red solid (140 mg, 33%); mp 102-105 °C (Lit. 106–108 °C);[12] R_f = 0.7 (eluent = 40% EtOAc in petroleum ether); vmax / cm-1 (film) 2980, 1612, 1462, 1251, 1155, 821; **^{1}H NMR (500 MHz, Chloroform-*d*)** δ 7.72 (s, 1H), 7.37 (dt, *J* = 8.5, 0.7 Hz, 1H), 6.81 (dt, *J* = 2.4, 0.7 Hz, 1H), 6.74 (dd, *J* = 8.5, 2.3 Hz, 1H), 6.13 (dp, *J* = 2.1, 1.0 Hz, 1H), 3.83 (s, 3H), 2.41 (d, *J* = 1.0 Hz, 3H); **^{13}C{^{1}H} NMR (126 MHz, Chloroform-*d*)** δ 155.8, 136.9, 133.9, 123.5, 120.2, 109.1, 100.2, 94.6, 55.9, 13.9; HRMS (EI-QUADRUPOLE) calculated $[C_{10}H_{11}NO]^+$ $(M)^+$ m/z 161.0835, found 161.0835.

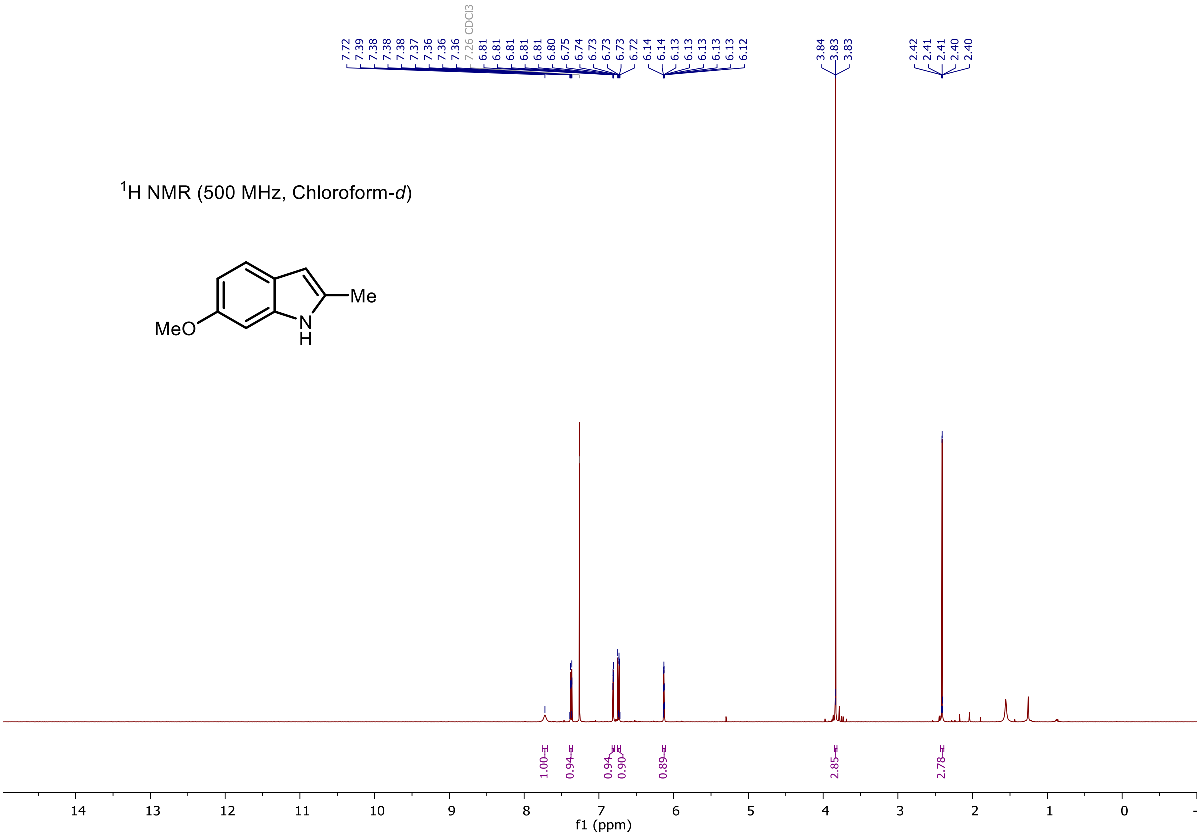
7.72
7.39
7.38
7.38
7.38
7.37
7.36
7.36
7.36
7.26 CDCl3
6.81
6.81
6.81
6.81
6.81
6.80
6.75
6.74
6.73
6.73
6.73
6.72
6.14
6.14
6.13
6.13
6.13
6.13
6.13
6.12
3.84
3.83
3.83
2.42
2.41
2.41
2.40
2.40
1H NMR (500 MHz, Chloroform-d)
Me
MeO
N
H
1.00
0.94
0.94
0.90
0.89
2.85
2.78
14
13
12
11
10
9
8
7
6
5
4
3
2
1
0
f1 (ppm)

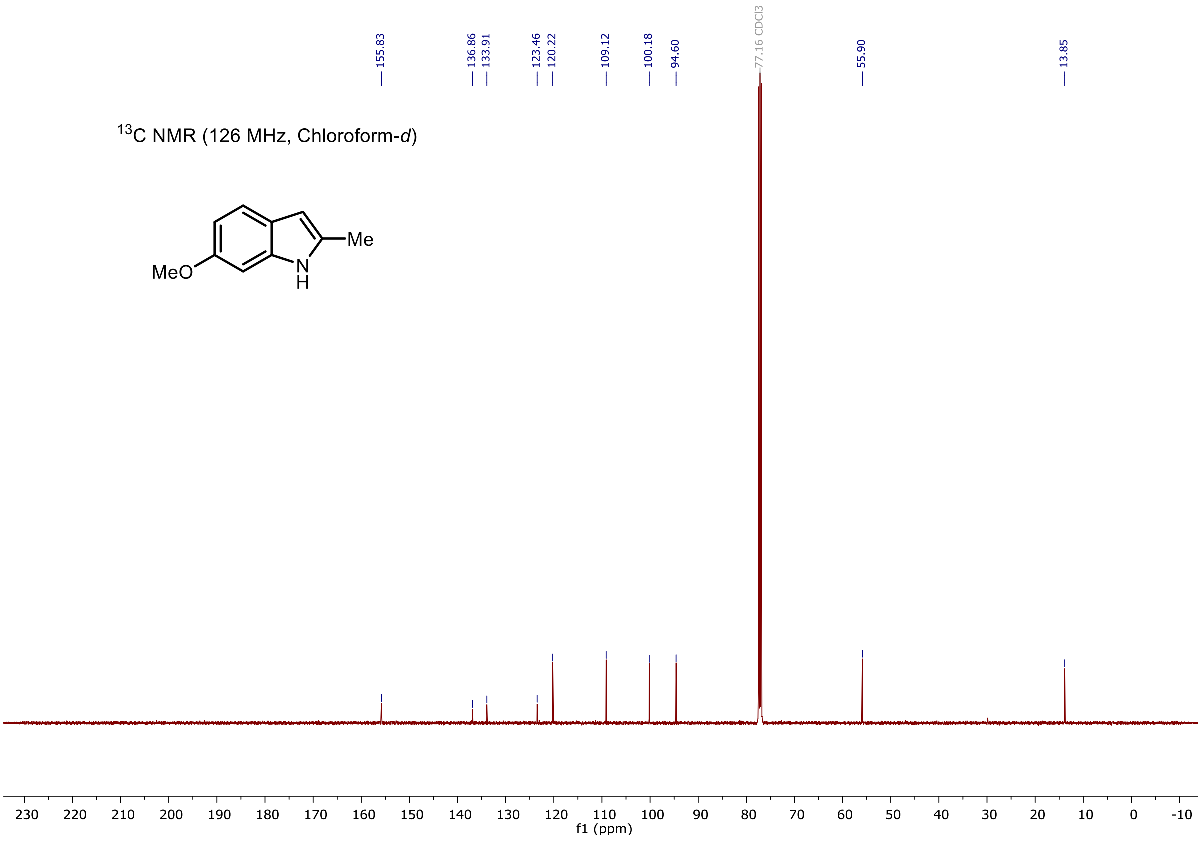
155.83
136.86
133.91
123.46
120.22
109.12
100.18
94.60
77.16 CDCl3
55.90
13.85
13C NMR (126 MHz, Chloroform-d)
Me
MeO
N
H
230
220
210
200
190
180
170
160
150
140
130
120
110
100
90
80
70
60
50
40
30
20
10
0
-10
f1 (ppm)

6-methoxy-1,2-dimethyl-1H-indole

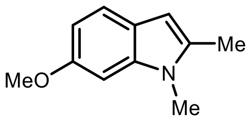

The title compound was prepared according to general procedure 3 using 6-methoxy-2-methyl-1H-indole (140 mg, 0.87 mmol). Purification by column chromatography on silica gel (eluent = 20% EtOAc in petroleum ether, 40 × 180 mm silica) gave the title compound as a red solid (114 mg, 75%); mp 67-71 °C; R_f = 0.51 (eluent = 20% EtOAc in petroleum ether); vmax / cm-1 (film) 2980, 1620, 1489, 1473, 1394, 1247, 1213, 1151, 1087, 954, 810; **^{1}H NMR (500 MHz, Chloroform-*d*)** δ 7.39 – 7.36 (m, 1H), 6.76 – 6.71 (m, 2H), 6.16 (q, *J* = 1.0 Hz, 1H), 3.87 (s, 3H), 3.61 (s, 3H), 2.39 (d, *J* = 1.0 Hz, 3H); **^{13}C{^{1}H} NMR (126 MHz, Chloroform-*d*)** δ 155.7, 138.1, 135.8, 122.3, 120.2, 108.7, 99.3, 93.3, 56.0, 29.6, 12.9; HRMS (CI-QUADRUPOLE) calculated $[C_{11}H_{13}ON]^+$ $(M)^+$ m/z 175.0992, found 175.0992.

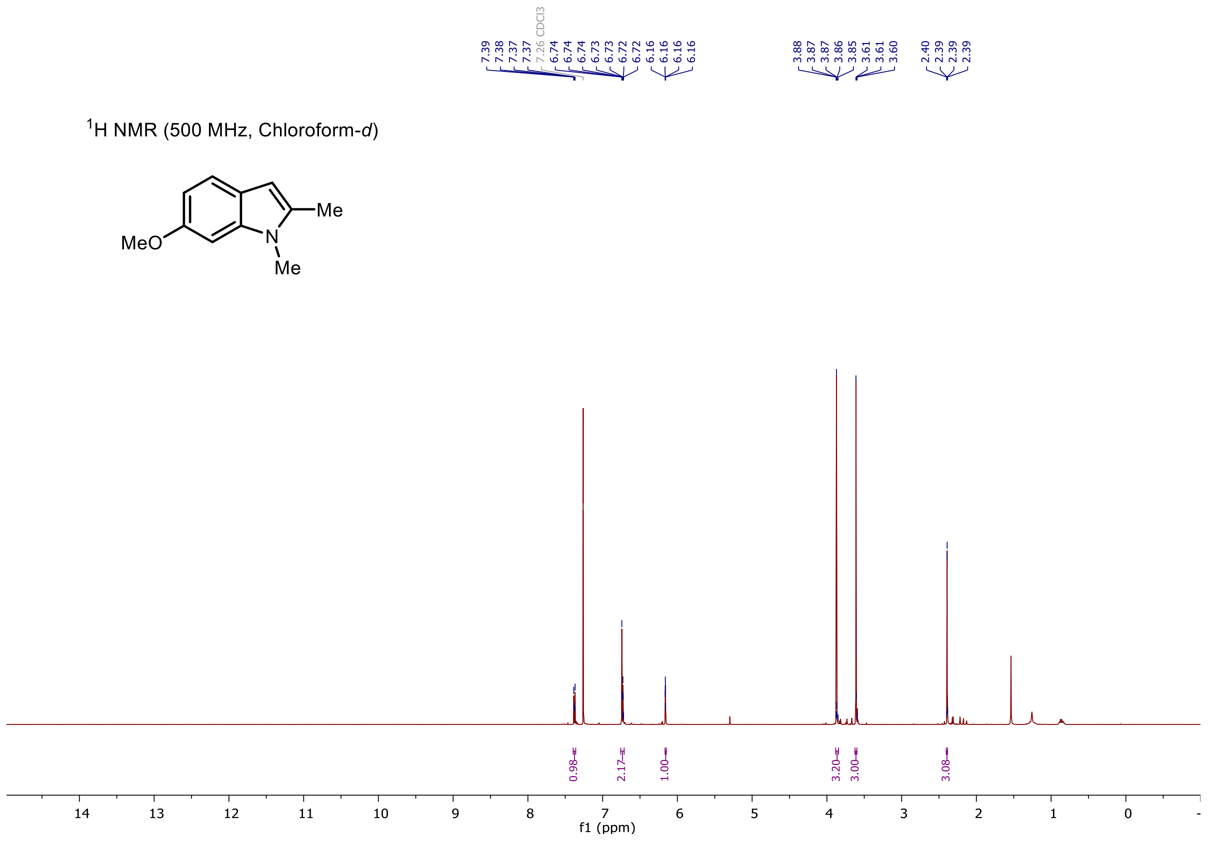

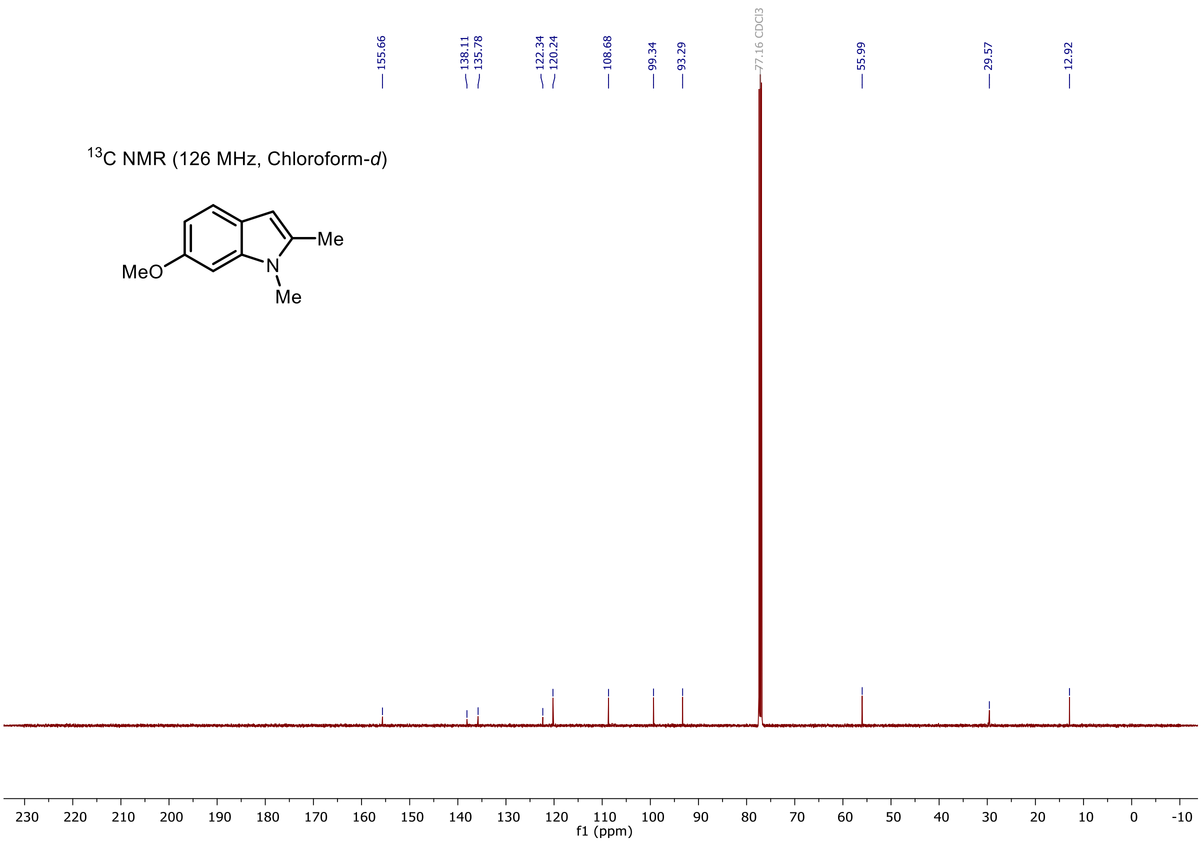

2.4. Synthesis of deuterated substrates

1,2-dimethyl-1*H*-indole-3-*d* (28)

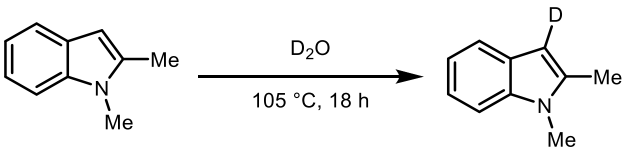

1,2-dimethylindole (500 mg) was dissolved in D_2O (1 mL/g of indole) and stirred vigorously at 105 °C for 18 h. Then, the reaction mixture was extracted with n-hexane (3 x 5 mL), dried over MgSO4, filtered, and concentrated in vacuo to give the title compound as a pink solid (400 mg, 79%, 75% *d*-incorporation); mp 49-53 °C; R_f = 0.82 (eluent = 10% EtOAc in petroleum ether); vmax / cm-1 (film) 3057, 3022, 2923, 2854, 1538, 1465, 1395, 1331, 1300, 1230, 1098, 1010, 904, 785, 732, 642, 563, 547, 506, 433; **^{1}H NMR (300 MHz, Chloroform-*d*)** δ 7.57 – 7.50 (m, 1H), 7.31 – 7.24 (m, 1H), 7.17 (ddt, *J* = 8.3, 7.0, 1.3 Hz, 1H), 7.09 (dddd, *J* = 8.5, 7.0, 1.5, 1.0 Hz, 1H), 6.27 (s, 0.23 H), 3.68 (s, 3H), 2.45 (s, 3H); **^{13}C{^{1}H} NMR (126 MHz, Chloroform-*d*) δ** 137.4, 136.8, 128.0, 120.5, 119.7, 119.3, 108.8, 99.7, 29.5, 12.9; HRMS (EI-QUADRUPOLE) calculated $[C_{10}H_{10}{}^{2}HN]^+$ (M^+) m/z 146.0949, found 146.0943. The spectroscopic data are in accordance with those described in the literature.[13]

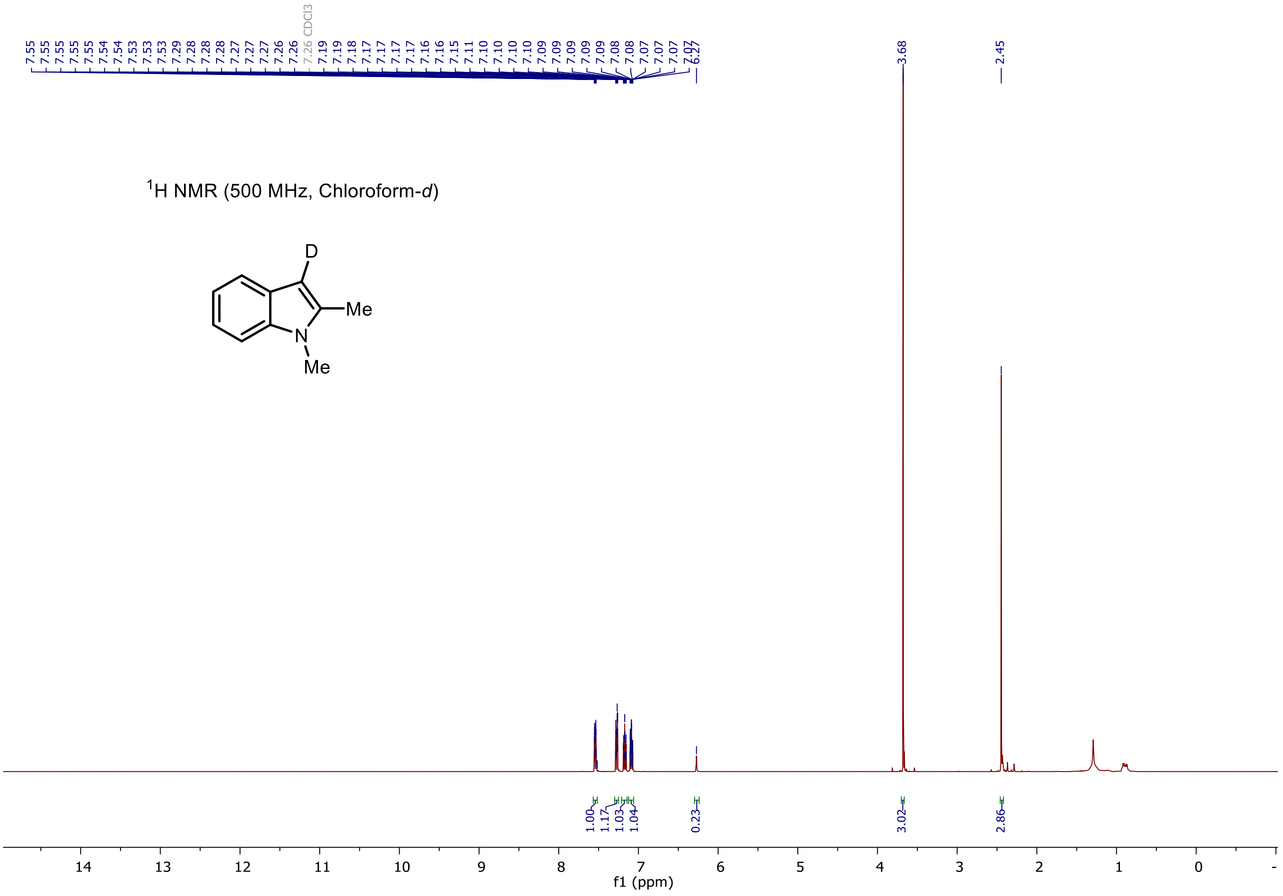

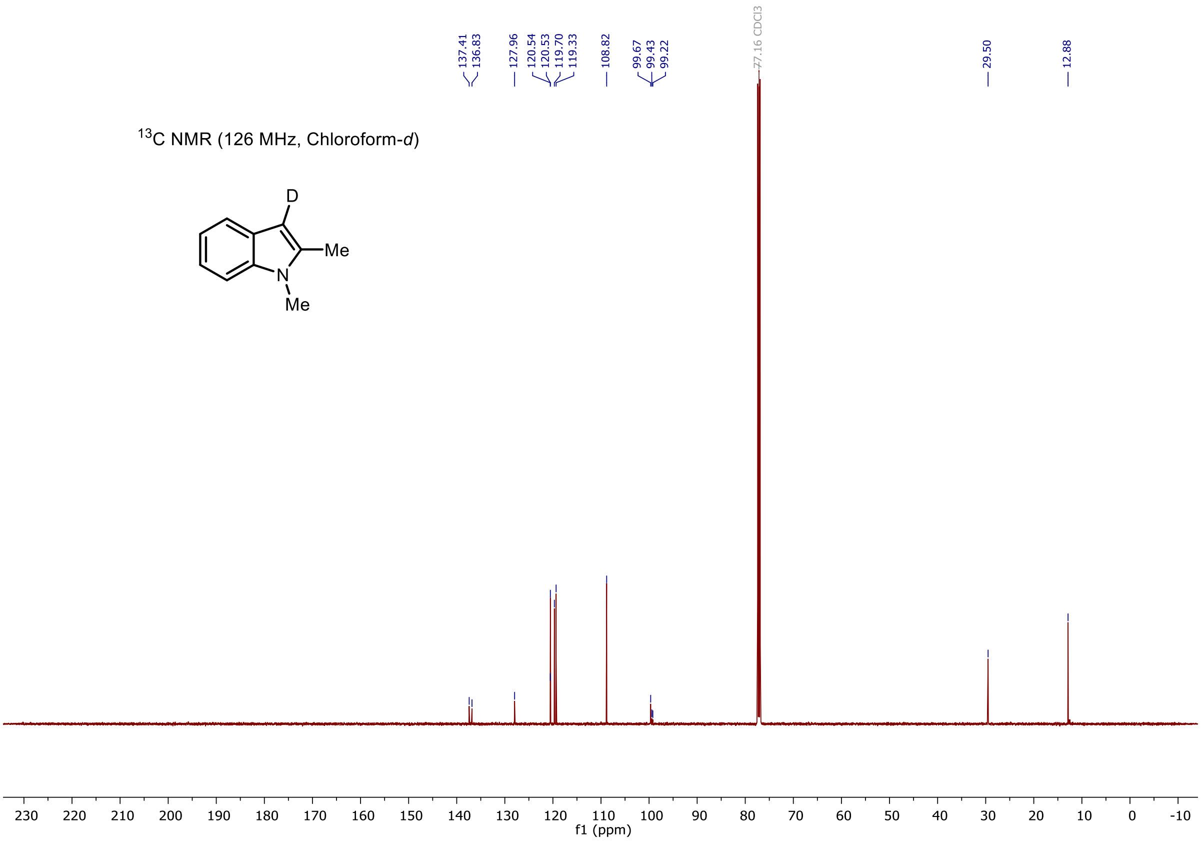

1-phenylethan-1-*d*-1-ol

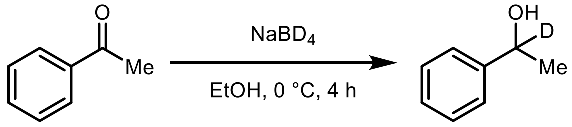

In a flame-dried flask, acetophenone (9 g, 74.9 mmol) was dissolved in ethanol (20 mL) and the solution was cooled to 0 °C. Sodium borodeuderide was then added portion wise over a period of 20 min. The mixture was stirred for 4 h at 0 °C. After completion, the reaction was quenched with sat. aq. NH_4Cl (200 mL) and extracted with EtOAc (3 x 100 mL), dried over $MgSO_4$, filtered, and concentrated in vacuo to give the title compound as an amber oil (8 g, 87%, >99% *d*-incorporation); R_f = 0.48 (eluent = 20% EtOAc in petroleum ether); vmax / cm-1 (film) 3300, 2980, 1543, 1508, 1490, 1446, 1338, 1240, 1134, 1074, 1026, 941, 835, 754, 698, 516; **^{1}H NMR (500 MHz, Chloroform-*d*)** δ 7.40 – 7.34 (m, 4H), 7.30 – 7.26 (m, 1H), 1.91 (s, 1H), 1.51 – 1.49 (m, 3H); **^{13}C{^{1}H} NMR (126 MHz, Chloroform-*d*)** δ 145.9, 128.6, 127.6, 125.5, 70.3 – 69.9 (m), 25.2; HRMS (EI-QUADRUPOLE) calculated $[C_8H_9{}^2HO]^+$ (M^+) m/z 123.0789, found 123.0787.

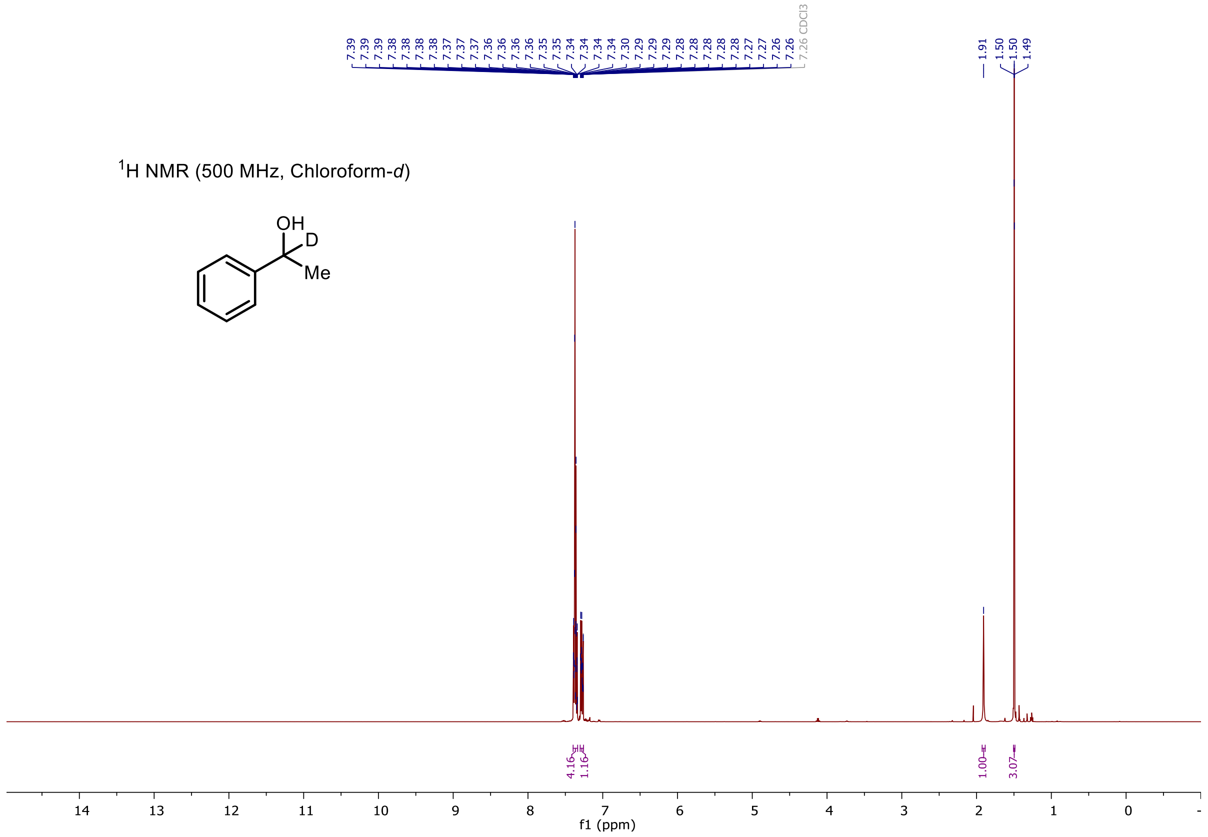

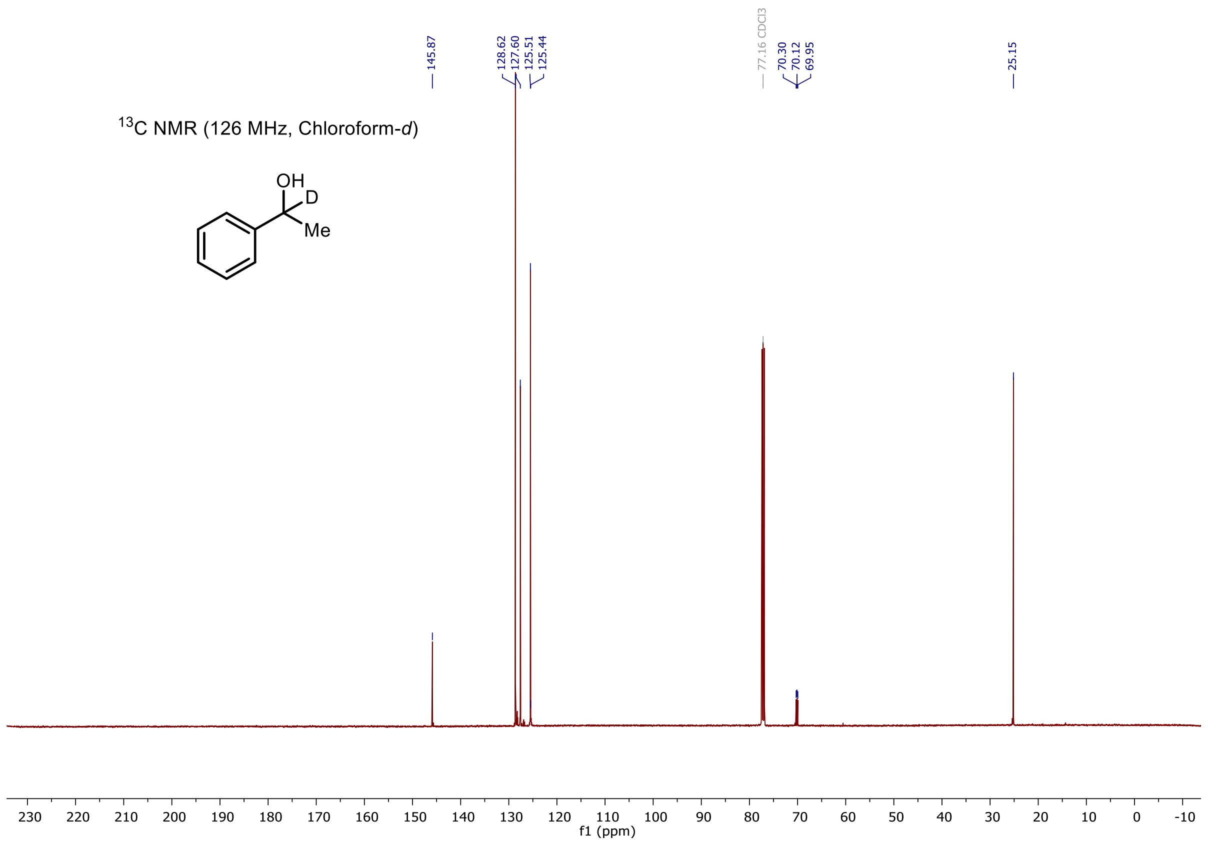

4-methoxy-N-(4-methoxyphenyl)-N-(1-phenylethyl-1-*d*)aniline (29)

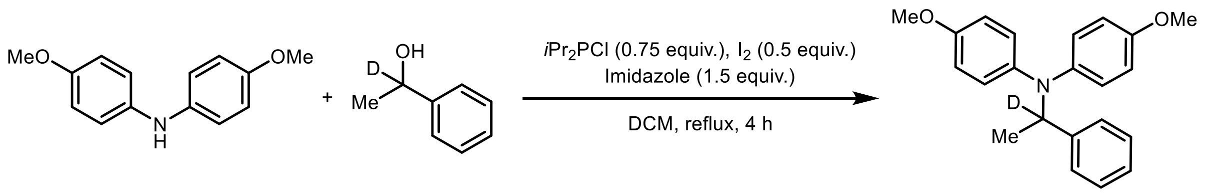

The title compound was prepared according to general procedure 2 using bis(4-methoxyphenyl)amine (1 g, 4.36 mmol). Purification by column chromatography on silica gel (eluent = 10% EtOAc in petroleum ether, 40 × 180 mm silica) gave the title compound as a red oil (326 mg, 45%, >99% *d*-incorporation); R_f = 0.41 (eluent = 10% EtOAc in petroleum ether); vmax / cm-1 (film) 2980, 2833, 1502, 1462, 1444, 1242, 1180, 1037, 819; **^{1}H NMR (500 MHz, Chloroform-*d*)** δ 7.38 – 7.34 (m, 2H), 7.31 – 7.26 (m, 2H), 7.23 – 7.17 (m, 1H), 6.80 – 6.72 (m, 8H), 3.75 (s, 6H), 1.43 (s, 3H); **^{13}C{^{1}H} NMR (126 MHz, Chloroform-*d*)** δ 154.6, 144.5, 141.4, 128.4, 127.2, 126.8, 124.2, 114.4, 55.7, 20.1; HRMS (EI-QUADRUPOLE) calculated $[C_{22}H_{23}{}^{2}HNO_2]^+$ $(M+H)^+$ m/z 335.1870, found 335.1870.

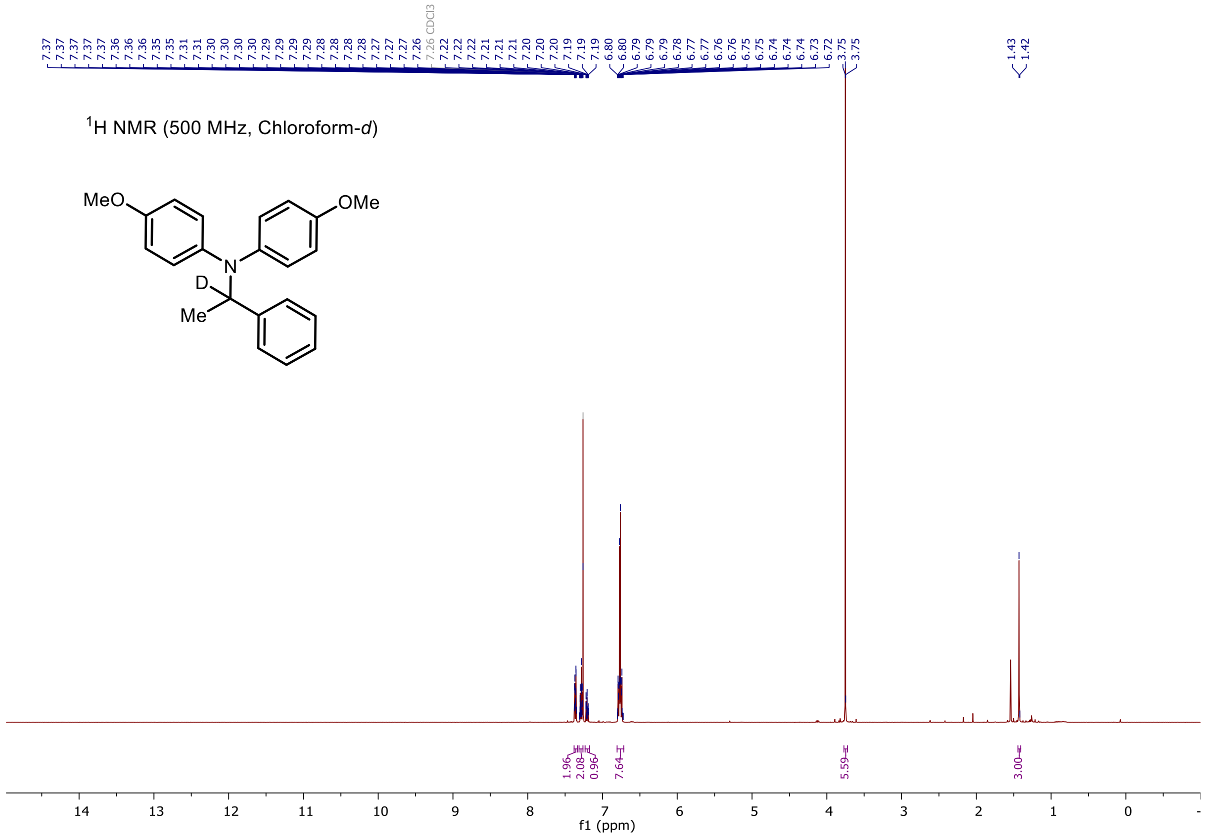

^{1}H NMR (500 MHz, Chloroform-*d*)
MeO
OMe
N
D
Me
1.43
1.42
3.75
3.75
7.26 CDCl3
f1 (ppm)

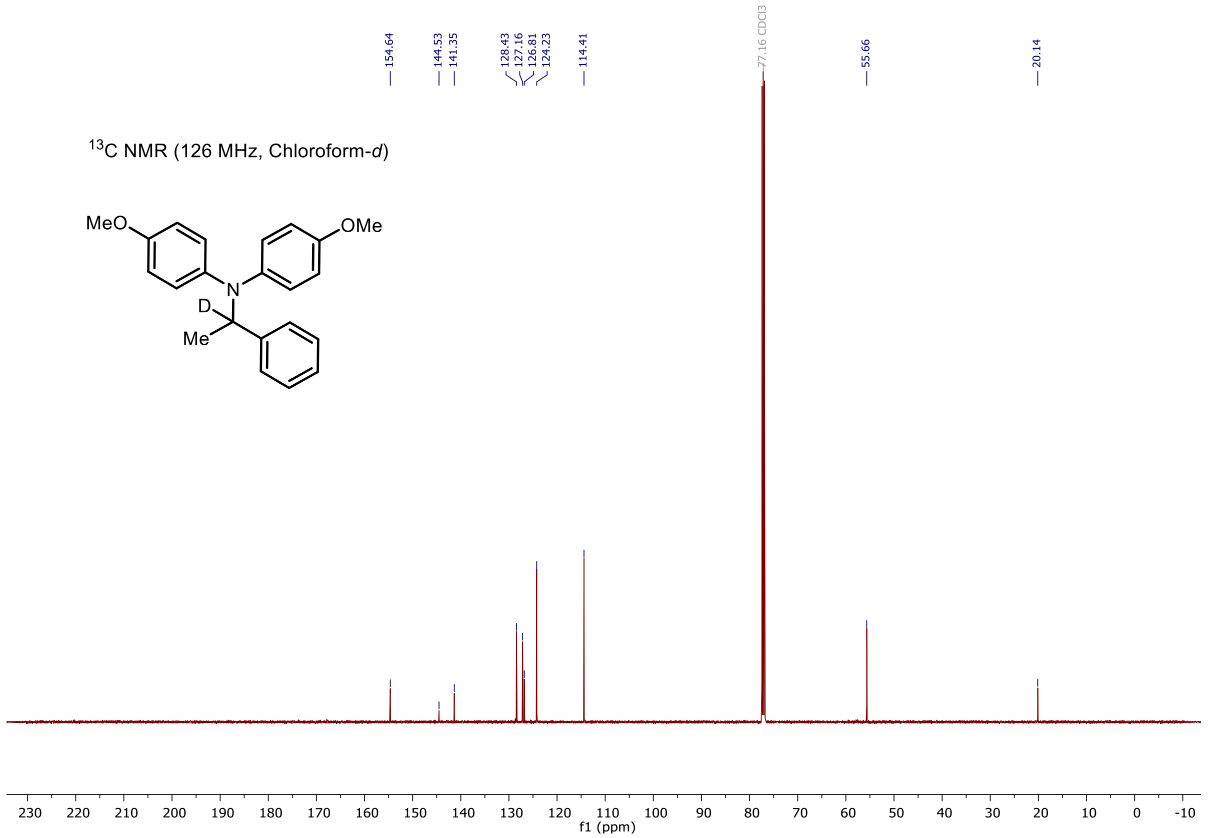

^{13}C NMR (126 MHz, Chloroform-*d*)
MeO
OMe
N
D
Me
154.64
144.53
141.35
128.43
127.16
126.81
124.23
114.41
77.16 CDCl3
55.66
20.14
f1 (ppm)

2.5. Optimization studies

Table S1. Screening of alkylating agents for C3 secondary alkylation

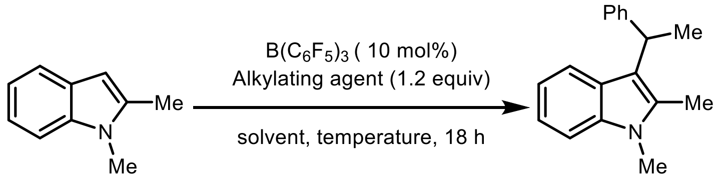

1 2

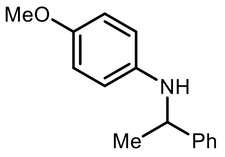

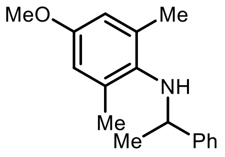

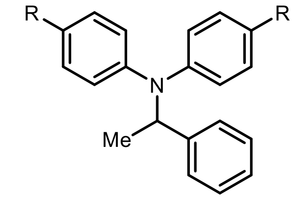

3 4 5, R = H; 6, R = Me; 7, R = OMe

Entry[a]	Alkylating agent, solvent and temperature	Yield[b] (%)
1	3, toluene, 110 °C	< 2%
2	4, toluene, 110 °C	< 2%
3	5, toluene, 110 °C	43
4	6, toluene, 110 °C	34
5	7, toluene, 110 °C	49
6	3, DCE, 25 °C	< 2%
7	3, DCE, 50 °C	< 2%
8	4, DCE, 50 °C	< 2%
9	5, DCE, 50 °C	62
10	6, DCE, 50 °C	54
11	7, DCE, 50 °C	50

[a]Reactions were performed using 0.1 mmol of 1,2-dimethylindole in its 0.5 [M] solution of DCE. [b] ^{1}HNMR yields determined from crude reaction mixtures using mesitylene as internal standard.

Table S2. Optimization of the C3 secondary alkylation of 1,2-disubstituted indole derivatives

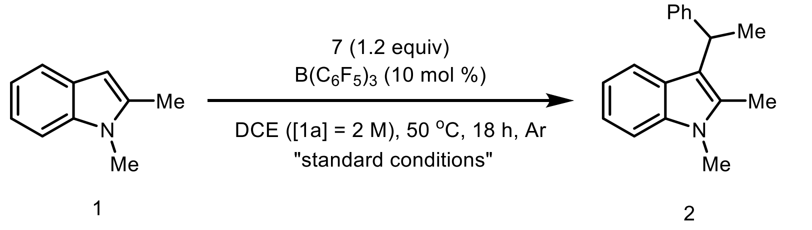

Entry[a]	Variation from standard conditions	Yield[b] (%)
1	None	84
2	No $B(C_6F_5)_3$	< 2%
3	6h	74
4	12h	79
5	24 h	80
6	$B(C_6F_5)_3$ (5 mol%)	55
7	$B(C_6F_5)_3$ (20 mol%)	80
8	DCE (1 [M])	69
9	DCE (4 [M])	75
10	No solvent	36
11	DCM (2 [M])	77
12	cyclohexane (2 [M])	80
13	toluene (2 [M])	70
14	8 (1 equiv.)	72
15	1,2-dimethylindole (0.12 mmole), 8 (1 equiv.)	65
16	30 °C in DCE	44
17	40 °C in DCE	66
18	60 °C in DCE	66

[a] Reactions were performed using 0.1 mmol of 1,2-dimethylindole in its 2 [M] solution of DCE. [b] ^{1}HNMR yields determined from crude reaction mixtures using mesitylene as internal standard

2.6. Substrate scope in the $B(C_6F_5)_3$-catalyzed alkylation

2.6.1. General procedure 5: C3 secondary alkylation of 1,2-disubstituted indole derivatives

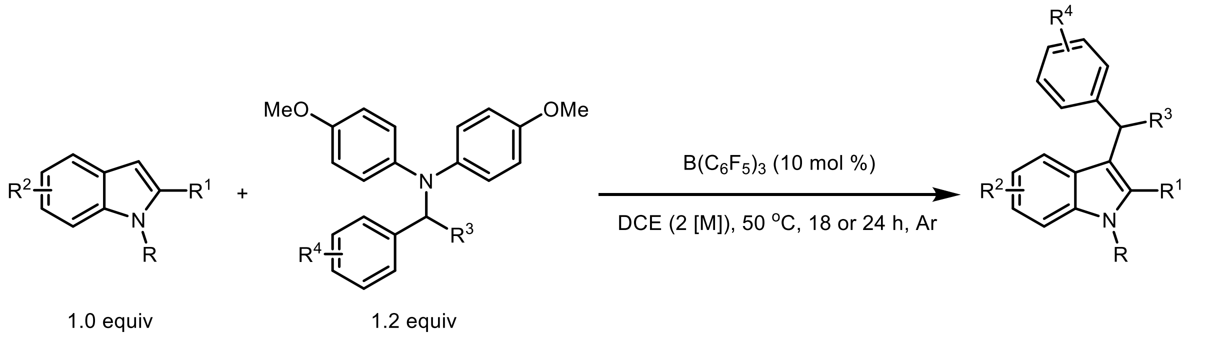

In an argon-filled glove box, a 10 mL vial equipped with a magnetic stirrer bar was charged with the 1,2-disubstituted indole (0.1 mmol), alkylating agent (0.12 mmol), $B(C_6F_5)_3$ (5 mg, 0.01 mmol) and DCE (50 µL). The vial was sealed with an aluminium crimped cap and was left to stir at 50 °C for 18 or 24 h. An NMR spectroscopic yield was obtained using 1,3,5-trimethylbenzene as an internal standard.

1,2-dimethyl-3-(1-phenylethyl)-1H-indole (2)

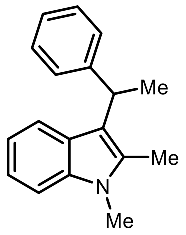

The title compound was prepared according to general procedure 5 using 1,2-dimethylindole (14.5 mg, 0.1 mmol) and 4-methoxy-N-(4-methoxyphenyl)-N-(1-phenylethyl)aniline (40 mg, 0.12 mmol) for 18 h. Yield determined by crude ^{1}H NMR using 1,3,5-trimethylbenzene as internal standard: 84%

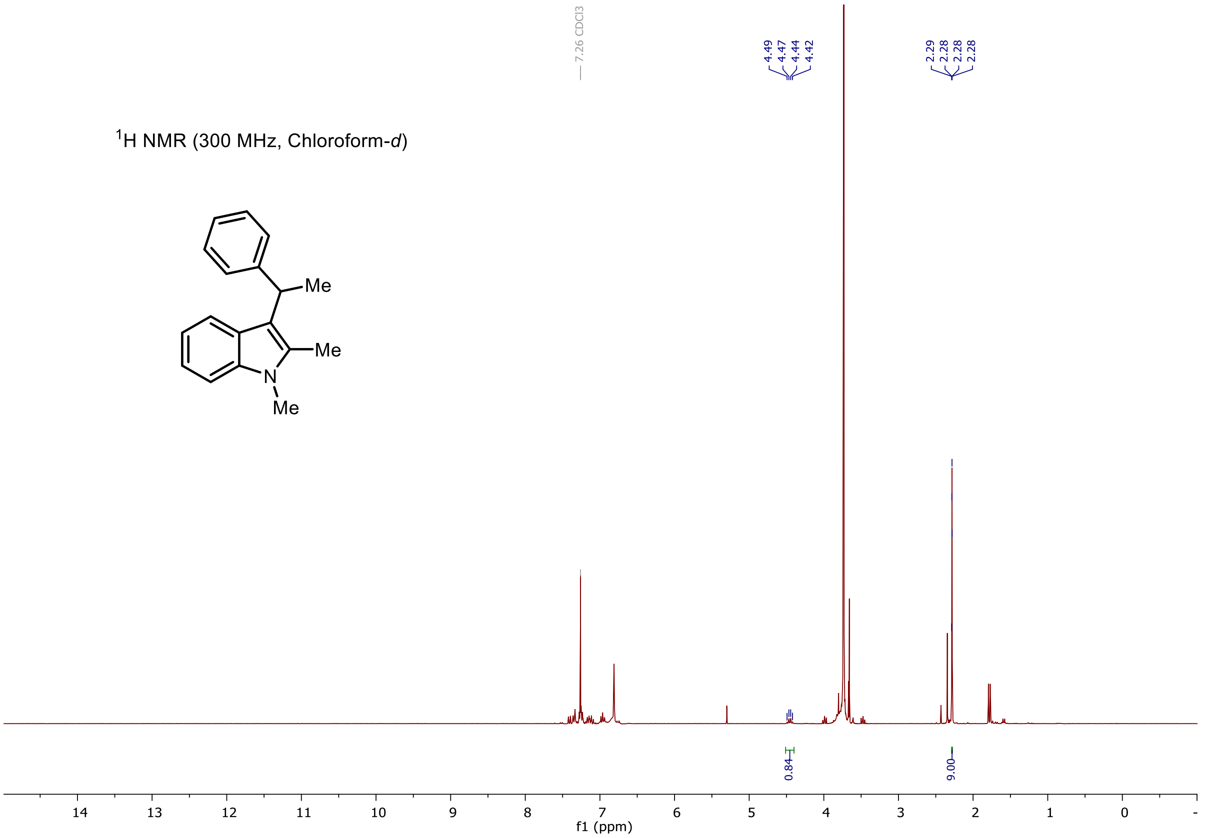

Purification by preparative TLC (eluent = 2.5% EtOAc in petroleum ether) gave the title compound as a brown oil (14.5 mg, 58%); R_f = 0.64 (eluent = 2.5% EtOAc in petroleum ether); vmax / cm-1 (film) 3055, 3024, 2964, 2929, 1490, 1469, 1446, 1406, 1367, 1332, 1251, 1178, 1068, 1022, 736, 698; **^{1}H NMR (500 MHz, Chloroform-*d*)** δ 7.42 (dt, *J* = 8.0, 0.9 Hz, 1H), 7.37 – 7.33 (m, 2H), 7.28 – 7.23 (m, 3H), 7.18 – 7.09 (m, 2H), 6.97 (ddt, *J* = 8.0, 7.0, 0.7 Hz, 1H), 4.46 (q, *J* = 7.3 Hz, 1H), 3.66 (d, *J* = 0.5 Hz, 3H), 2.35 (s, 3H), 1.79 (dd, *J* = 7.3, 0.5 Hz, 3H); **^{13}C{^{1}H}**

NMR (126 MHz, Chloroform-*d*) δ 146.5, 136.9, 132.6, 128.2, 127.5, 126.9, 125.6, 120.4, 119.5, 118.7, 115.6, 108.7, 35.8, 29.6, 20.9, 10.7; HRMS (CI-QUADRUPOLE) calculated $[C_{18}H_{19}N]^+$ (M^+) m/z 249.1512, found 249.1512. The spectroscopic data are in accordance with those described in the literature.[14]

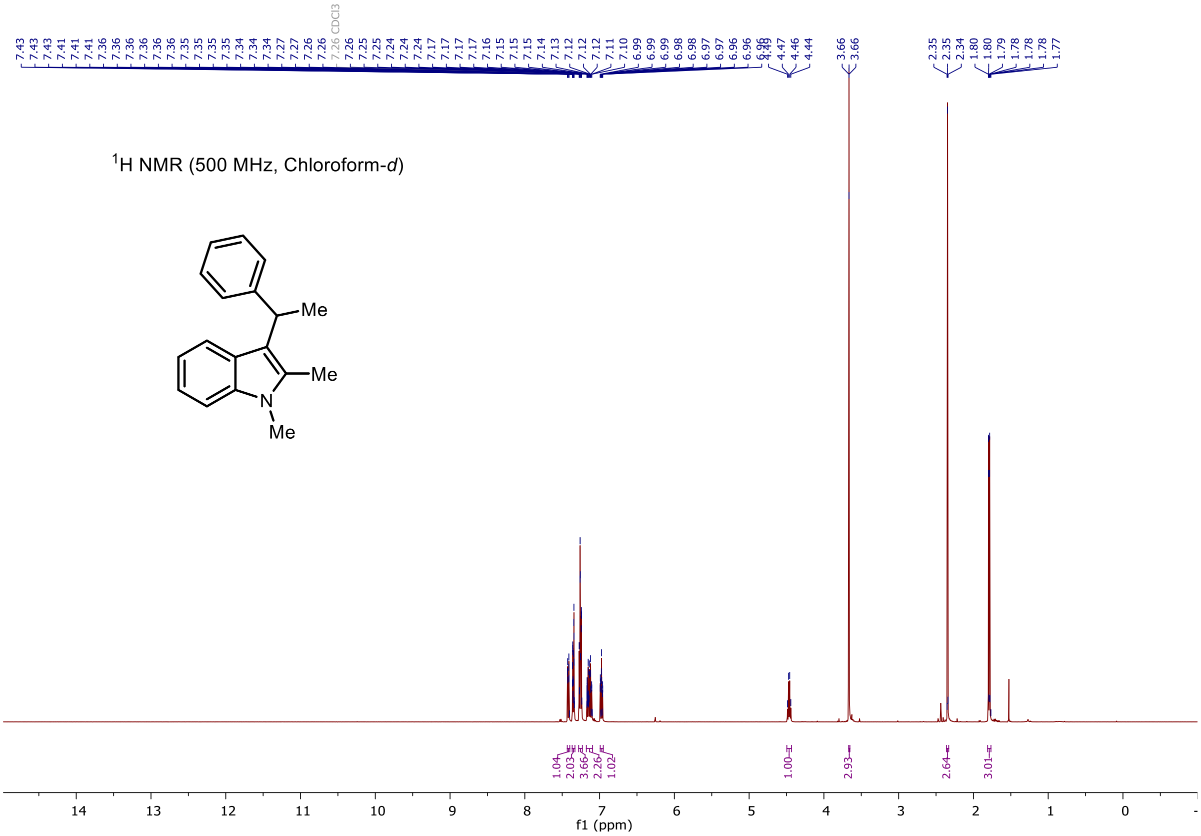

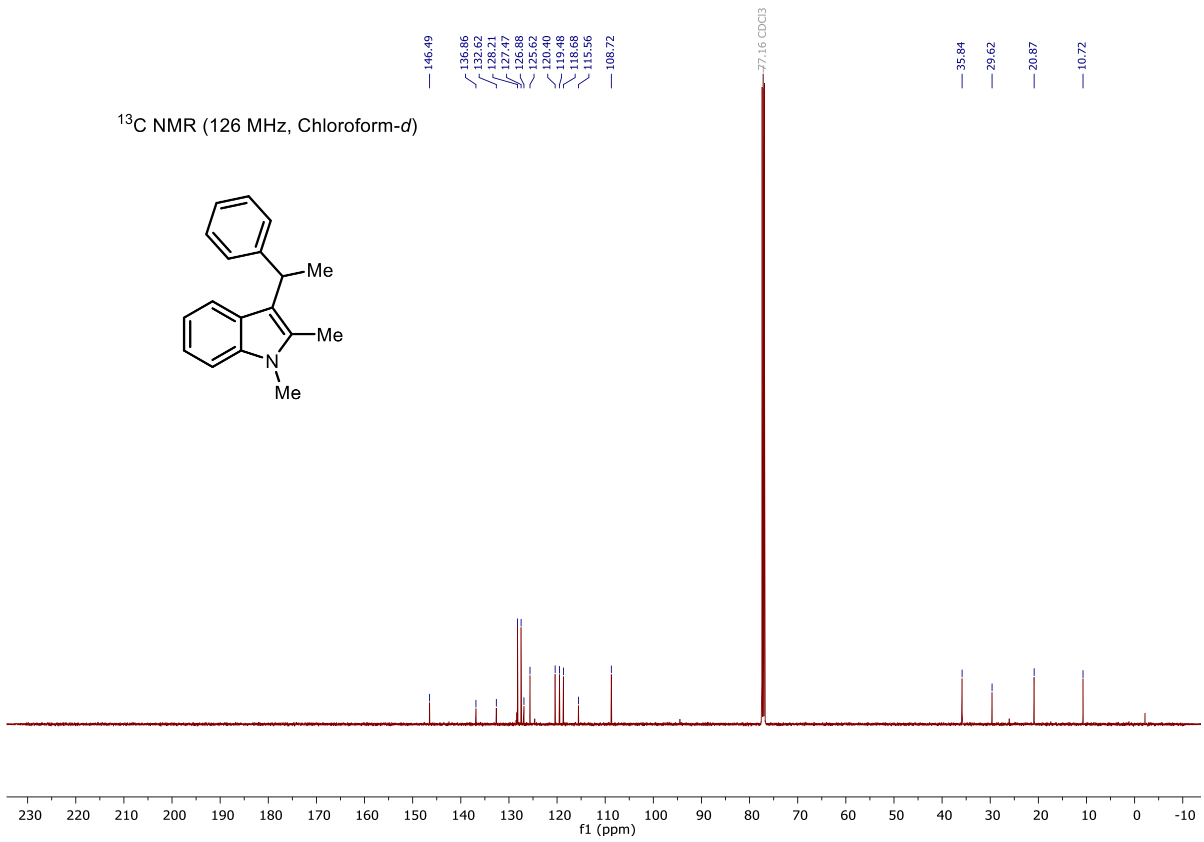

1,2-dimethyl-3-(1-(o-tolyl)ethyl)-1H-indole (8)

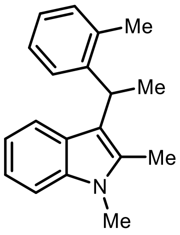

The title compound was prepared according to general procedure 5 using 1,2-dimethylindole (14.5 mg, 0.1 mmol) and 4-methoxy-N-(4-methoxyphenyl)-N-(1-(o-tolyl)ethyl)aniline (41.7 mg, 0.12 mmol) for 18 h. Yield determined by crude ^{1}H NMR using 1,3,5-trimethylbenzene as internal standard: 54%

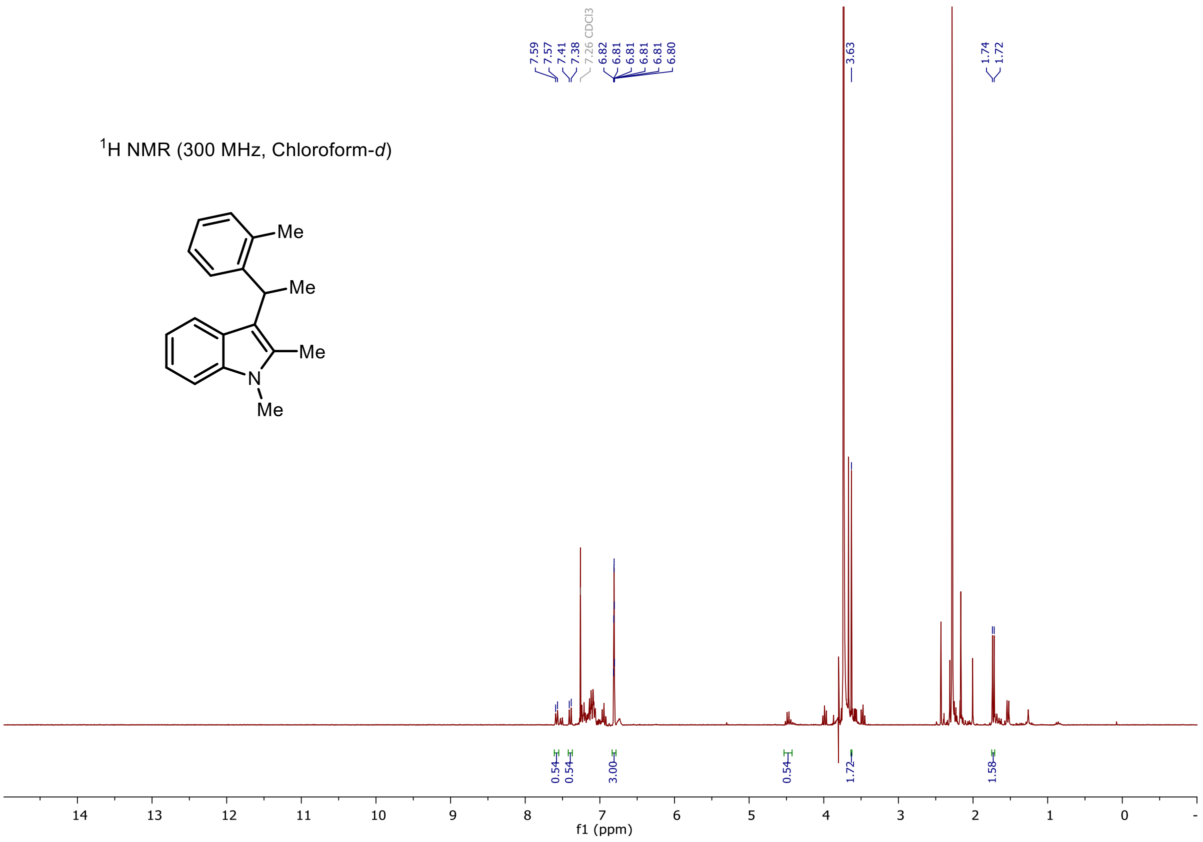

% yield = compound integral/standard integral x standard proton/compound proton x 100

= 0.18/1 x 3/1 x 100 = 54%

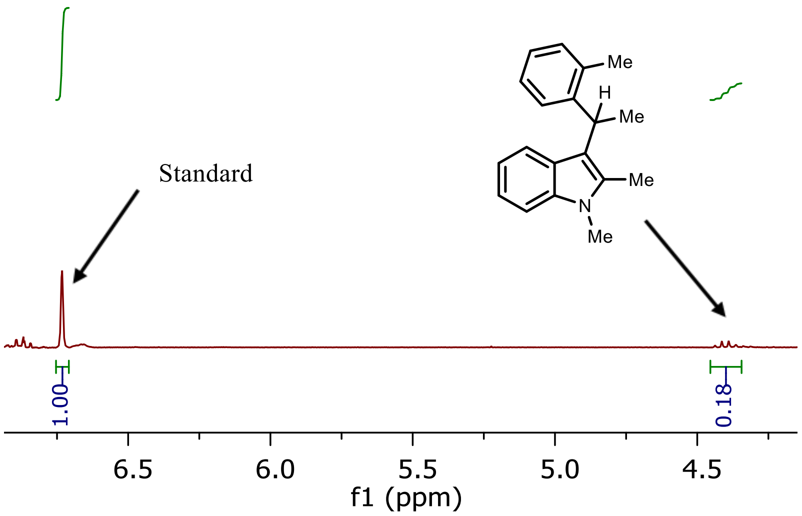

A part of the product was isolated using preparative TLC (eluent = 2.5% EtOAc in petroleum ether) as a brown oil; R_f = 0.4 (eluent = 2.5% EtOAc in petroleum ether); vmax / cm-1 (film) 2980, 1471, 1458, 1338, 1251, 1072, 952, 736, 418; **^{1}H NMR (500 MHz, Chloroform-*d*)** δ 7.61 – 7.58 (m, 1H), 7.42 (dt, *J* = 8.0, 0.9 Hz, 1H), 7.25 – 7.22 (m, 1H), 7.15 – 7.08 (m, 4H), 6.97 (ddd, *J* = 8.0, 7.0, 1.1 Hz, 1H), 4.50 (q, *J* = 7.3 Hz, 1H), 3.64 (s, 3H), 2.29 (s, 3H), 2.19 (s, 3H), 1.75 (d,

J = 7.3 Hz, 3H); **^{13}C{^{1}H} NMR (126 MHz, Chloroform-*d*)** δ 144.1, 136.70, 136.65, 132.5, 130.5, 127.1, 126.7, 125.9, 125.8, 120.3, 119.1, 118.6, 114.5, 108.6, 33.6, 29.6, 21.1, 19.8, 10.6; HRMS (CI-QUADRUPOLE) calculated $[C_{19}H_{21}N]^+$ (M^+) m/z 263.1669, found 263.1669.

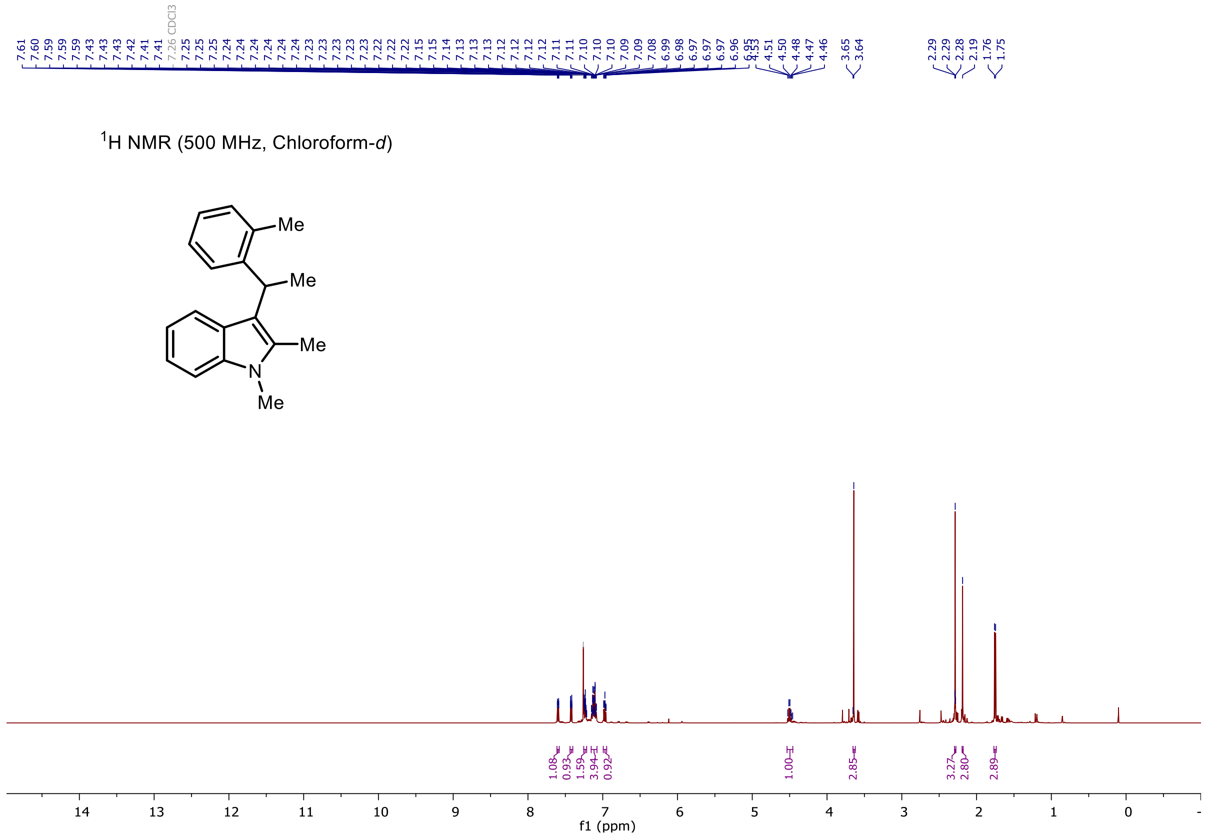

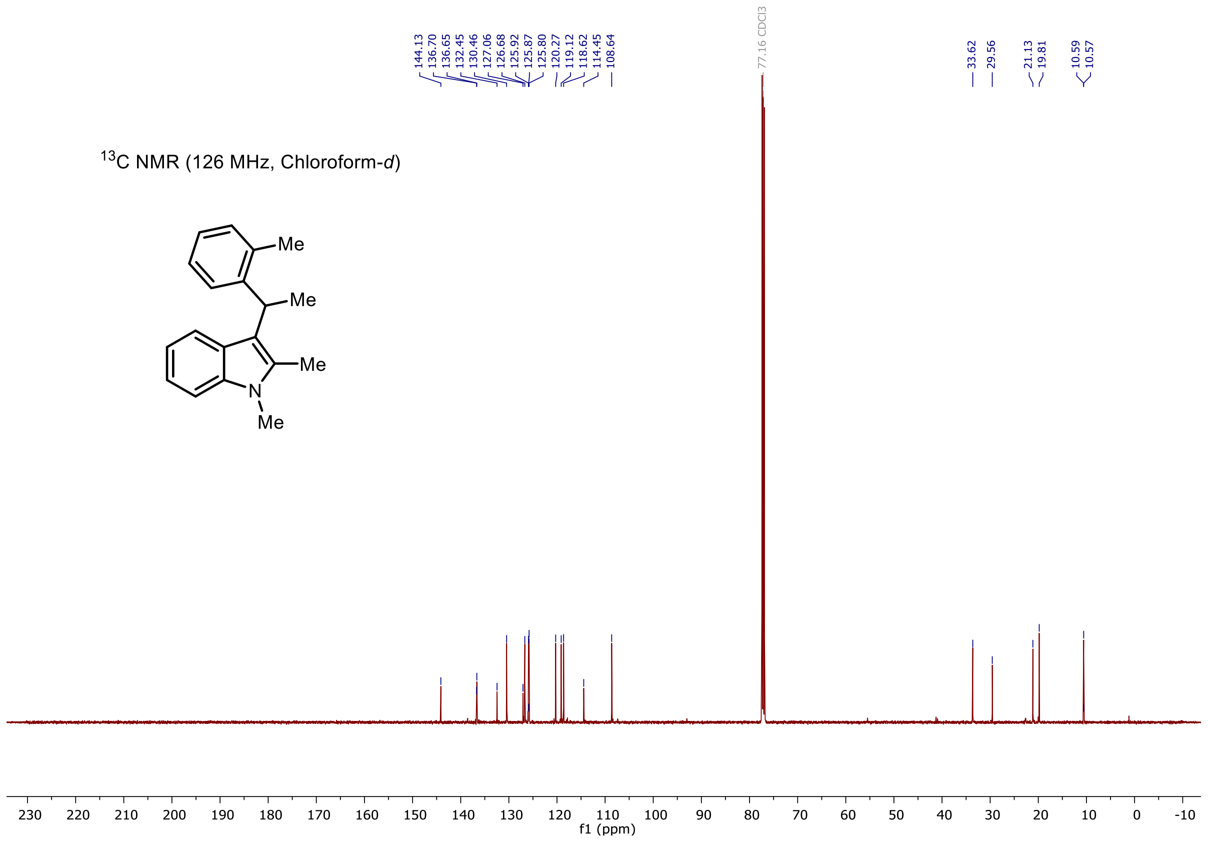

1,2-dimethyl-3-(1-(m-tolyl)ethyl)-1H-indole (9)

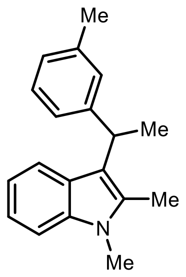

The title compound was prepared according to general procedure 5 using 1,2-dimethylindole (14.5 mg, 0.1 mmol) and 4-methoxy-N-(4-methoxyphenyl)-N-(1-(m-tolyl)ethyl)aniline (41.7 mg, 0.12 mmol) for 18 h. Yield determined by crude ^{1}H NMR using 1,3,5-trimethylbenzene as internal standard: 75%

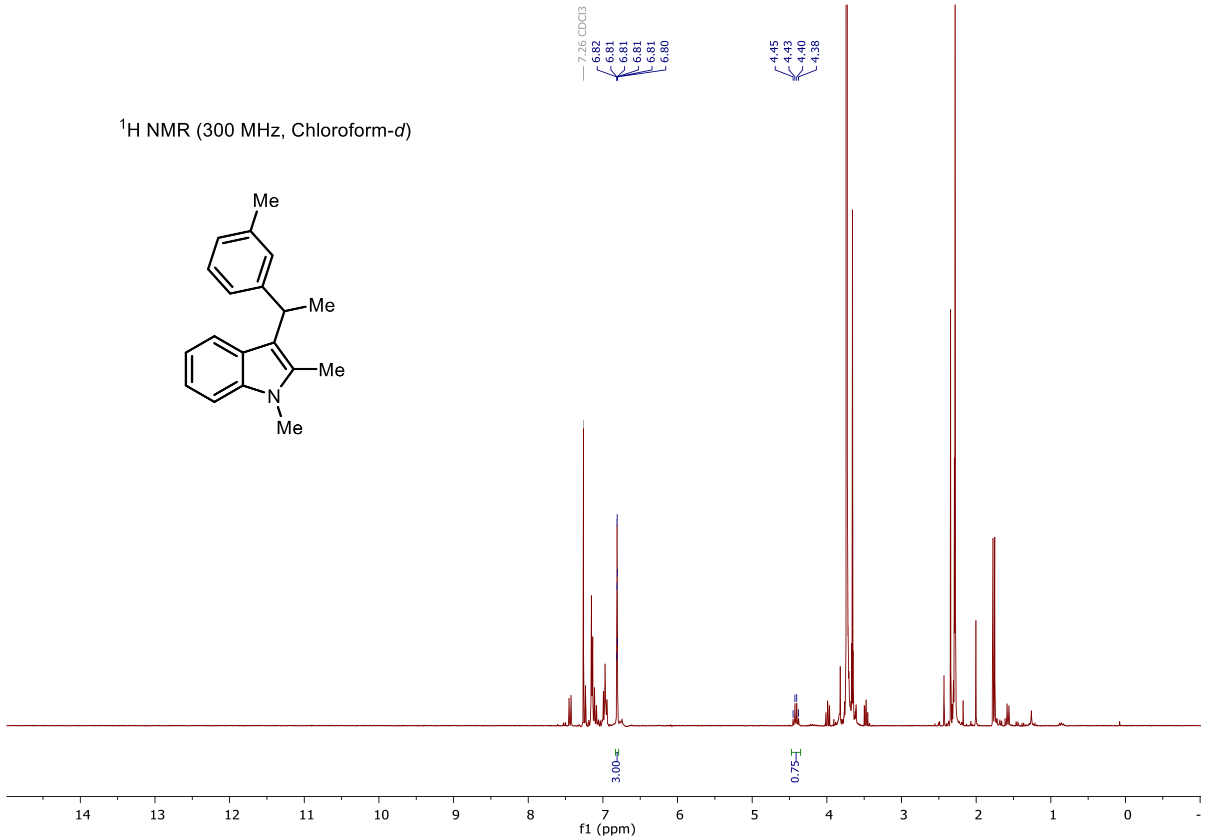

Purification by preparative TLC (eluent = 2.5% EtOAc in petroleum ether) gave the title compound as a light brown oil (13.6 mg, 52%); R_f = 0.38 (eluent = 2.5% EtOAc in petroleum ether); vmax / cm-1 (film) 2980, 1610, 1471, 1369, 1251, 1157, 956, 786, 740; **^{1}H NMR (500 MHz, Chloroform-*d*)** δ 7.55 (dt, *J* = 7.9, 1.0 Hz, 1H), 7.38 – 7.34 (m, 1H), 7.27 – 7.19 (m, 4H), 7.11 – 7.05 (m, 2H), 4.57 – 4.47 (m, 1H), 3.76 (s, 3H), 2.45 (s, 3H), 2.40 (d, *J* = 0.7 Hz, 3H), 1.87 (d, *J* = 7.3 Hz, 3H); **^{13}C{^{1}H} NMR (126 MHz, Chloroform-*d*)** δ 146.4, 137.7, 136.8, 132.6, 128.2 (d, J = 26.7 Hz), 126.9, 126.4, 124.4, 120.3, 119.5, 118.6, 115.6, 108.7, 35.8, 29.6, 21.7, 20.9, 10.8; HRMS (CI-QUADRUPOLE) calculated $[C_{19}H_{21}N]^+$ (M^+) m/z 263.1669, found 263.1671.

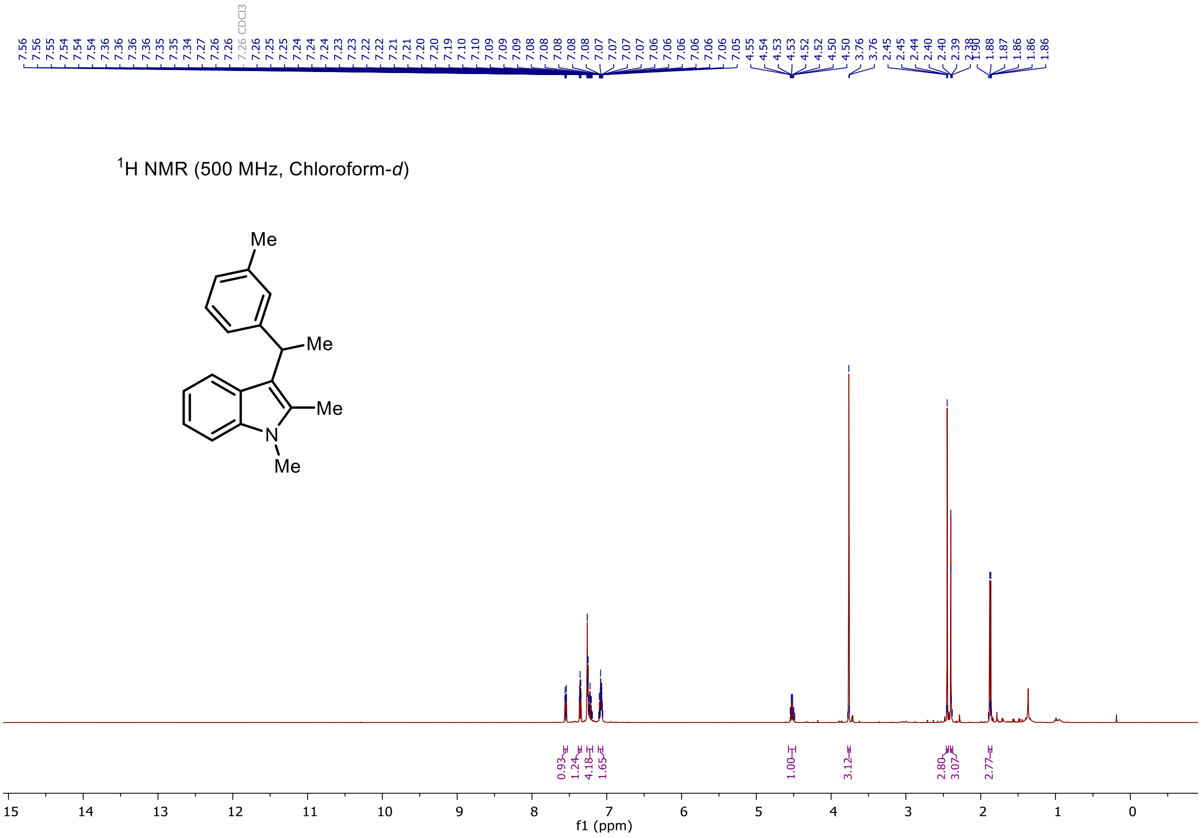

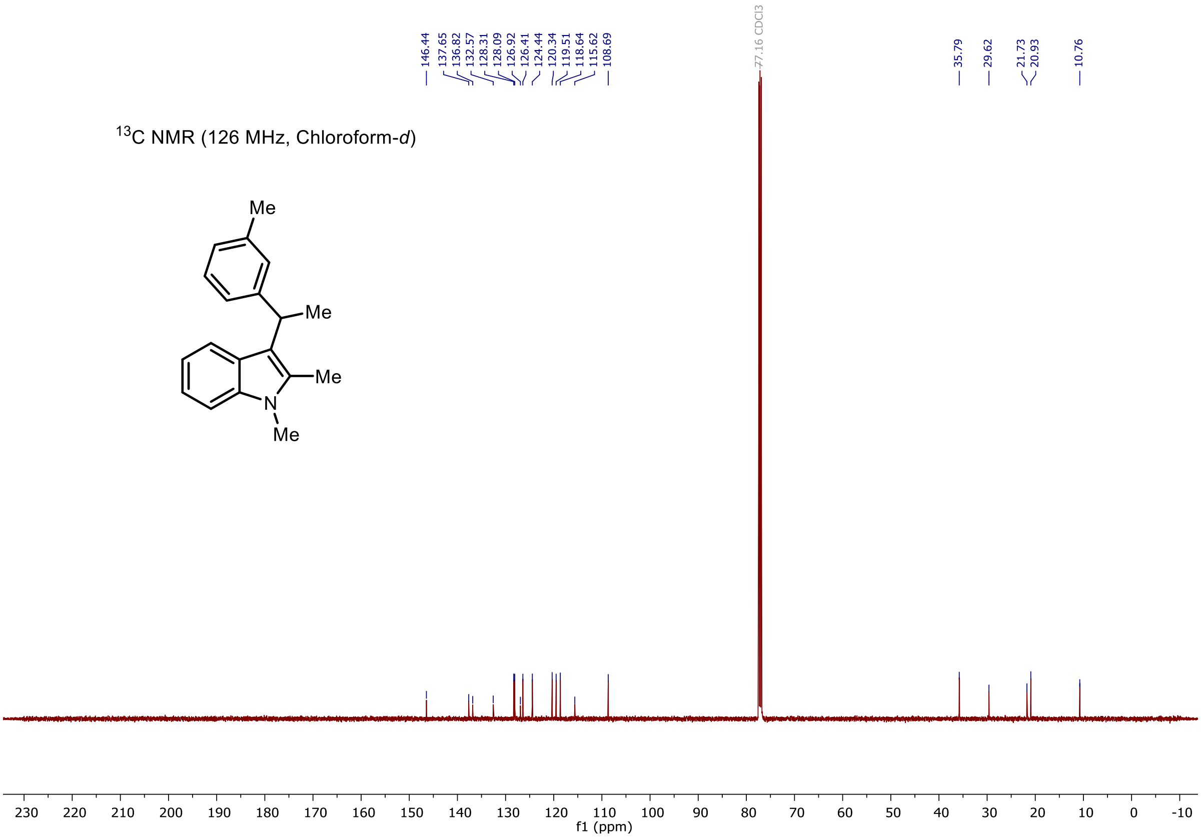

3-(1-(3-methoxyphenyl)ethyl)-1,2-dimethyl-1H-indole (10)

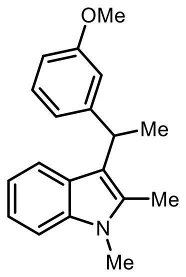

The title compound was prepared according to general procedure 5 using 1,2-dimethylindole (14.5 mg, 0.1 mmol) and 4-methoxy-N-(3-methoxyphenyl)-N-(1-(4-methoxyphenyl)ethyl)aniline (43.6 mg, 0.12 mmol) for 24 h. Yield determined by crude ^{1}H NMR using 1,3,5-trimethylbenzene as internal standard: 67%

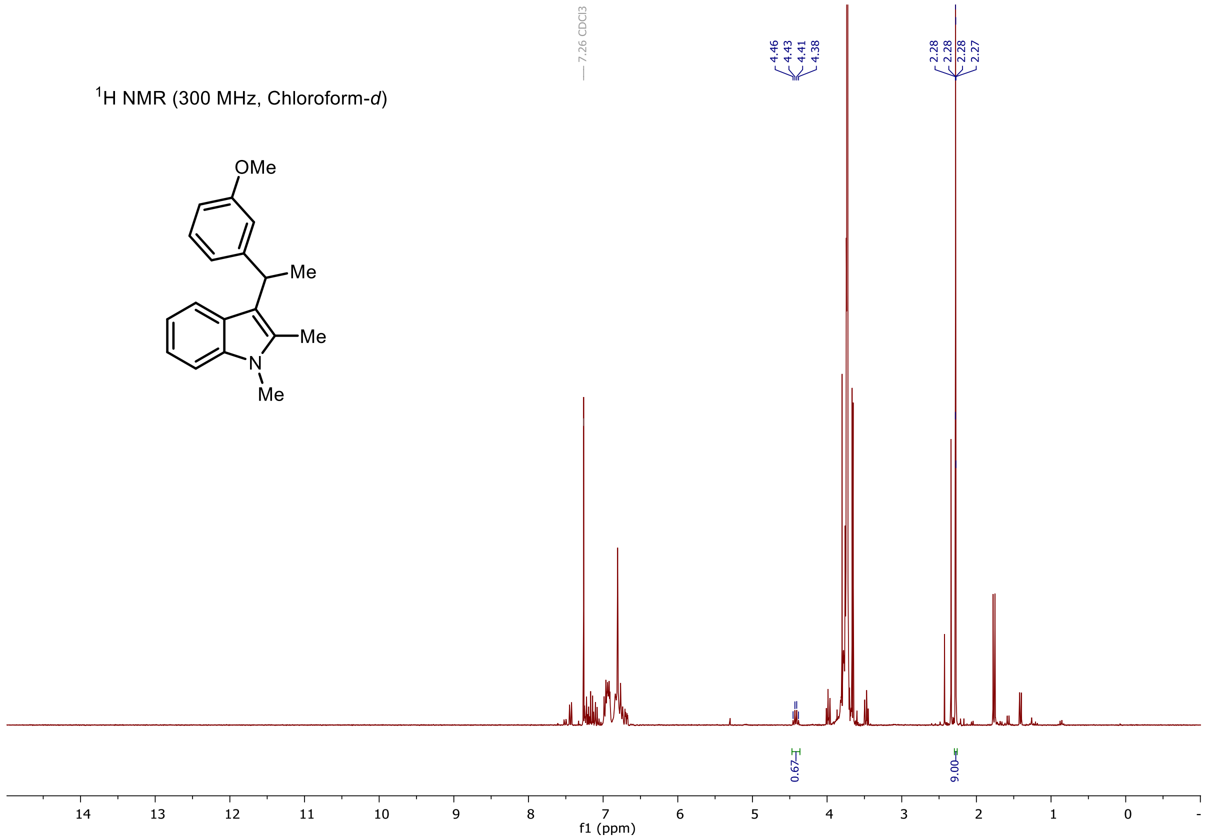

A part of the product was isolated using preparative TLC (eluent = 2.5% EtOAc in petroleum ether) as a brown oil; R_f = 0.36 (eluent = 2.5% EtOAc in petroleum ether); vmax / cm-1 (film) 2980, 2889, 1543, 1508, 1489, 1473, 1338, 1153, 1072, 740; **^{1}H NMR (500 MHz, Chloroform-d)** δ 7.44 (dt, J = 7.9, 0.9 Hz, 1H), 7.24 (dt, J = 8.1, 0.9 Hz, 1H), 7.17 (t, J = 7.9 Hz, 1H), 7.11 (ddd, J = 8.2, 7.0, 1.2 Hz, 1H), 7.00 – 6.88 (m, 3H), 6.69 (dd, J = 8.2, 2.6 Hz, 1H), 4.42 (q, J = 7.3 Hz, 1H), 3.74 (s, 3H), 3.65 (s, 3H), 2.34 (s, 3H), 1.76 (d, J = 7.3 Hz, 3H). **^{13}C{^{1}H} NMR (126 MHz, Chloroform-*d*)** δ 148.3, 142.9, 136.8, 132.1, 129.1, 126.9, 120.4, 120.1, 119.5, 118.7, 115.4, 113.8, 110.4, 108.7, 55.3, 35.9, 29.6, 20.9, 10.7; HRMS (ESI-TOF) calculated $[C_{19}H_{22}NO]^+$ $(M+H)^+$ m/z 280.1704, found 280.1701.

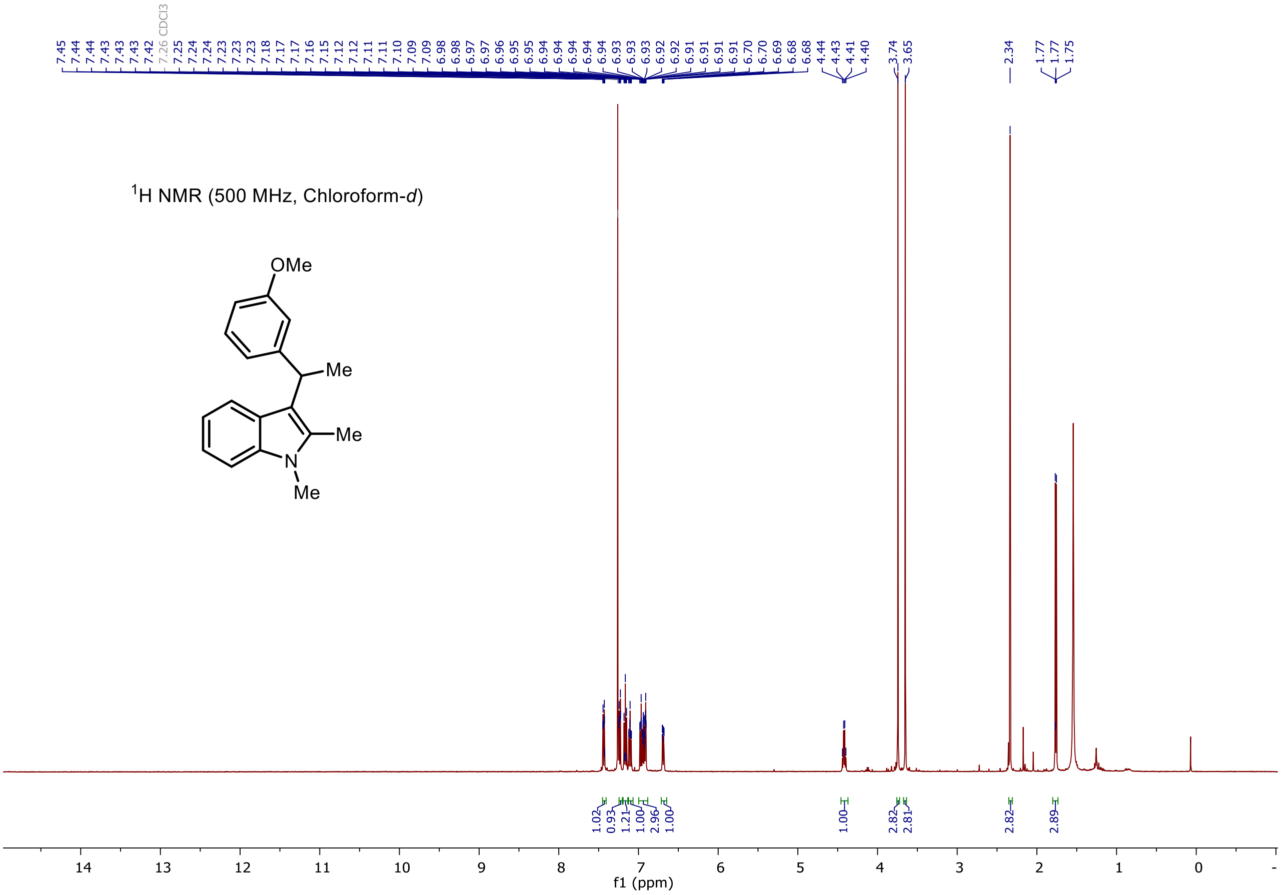

^{1}H NMR (500 MHz, Chloroform-*d*)
OMe
Me
Me
N
Me
f1 (ppm)

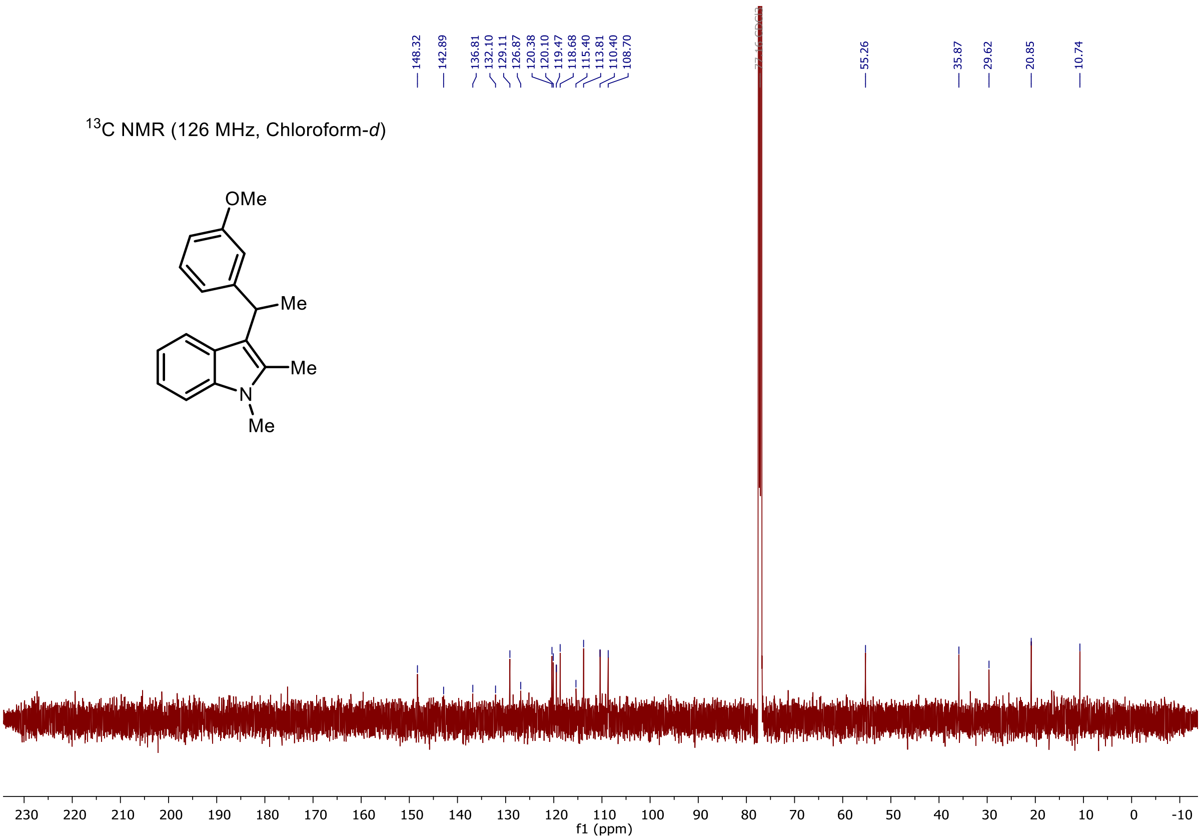

^{13}C NMR (126 MHz, Chloroform-*d*)
OMe
Me
Me
N
Me
148.32
142.89
136.81
132.10
129.11
126.87
120.38
120.10
119.47
118.68
115.40
113.81
110.40
108.70
55.26
35.87
29.62
20.85
10.74
f1 (ppm)

1,2-dimethyl-3-(1-(p-tolyl)ethyl)-1H-indole (11)

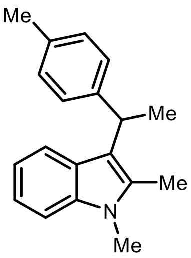

The title compound was prepared according to general procedure 5 using 1,2-dimethylindole (14.5 mg, 0.1 mmol) and 4-methoxy-N-(4-methoxyphenyl)-N-(1-(p-tolyl)ethyl)aniline (41.7 mg, 0.12 mmol) for 18 h. Yield determined by crude ^{1}H NMR using 1,3,5-trimethoxybenzene as internal standard: 65%

Resolved signals of 1,2-dimethyl-3-(1-(p-tolyl)ethyl)-1H-indole:[15]

^{1}H NMR (300 MHz, Chloroform-*d*) δ 7.42 (d, *J* = 7.9 Hz, 1H), 4.41 (q, *J* = 7.3 Hz, 1H), 1.76 (d, *J* = 7.3 Hz, 3H).

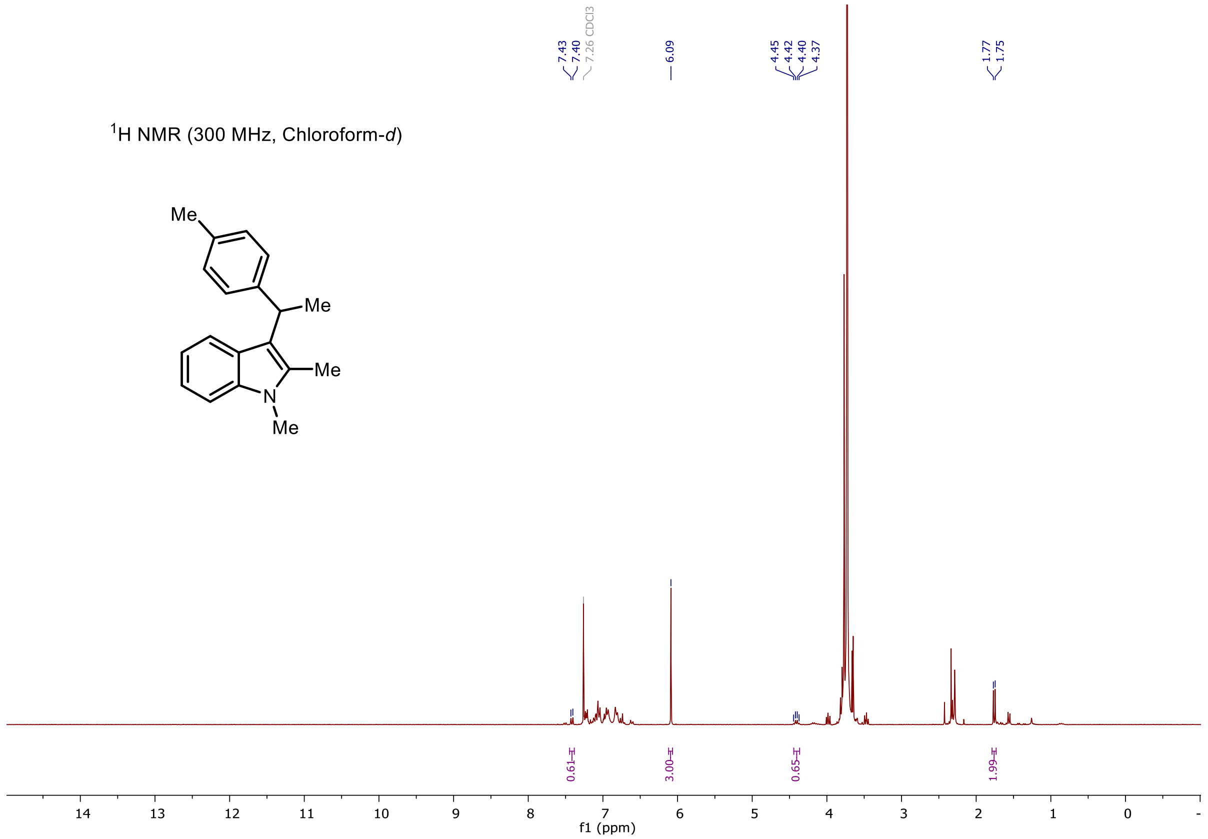

3-(1-(4-methoxyphenyl)ethyl)-1,2-dimethyl-1H-indole (12)

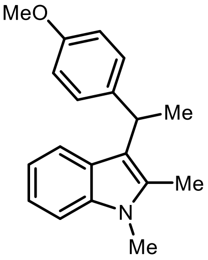

The title compound was prepared according to general procedure 5 using 1,2-dimethylindole (14.5 mg, 0.1 mmol) and 4-methoxy-N-(4-methoxyphenyl)-N-(1-(4-methoxyphenyl)ethyl)aniline (43.6 mg, 0.12 mmol) for 24 h. Yield determined by crude ^{1}H NMR using 1,3,5-trimethylbenzene as internal standard: 76%

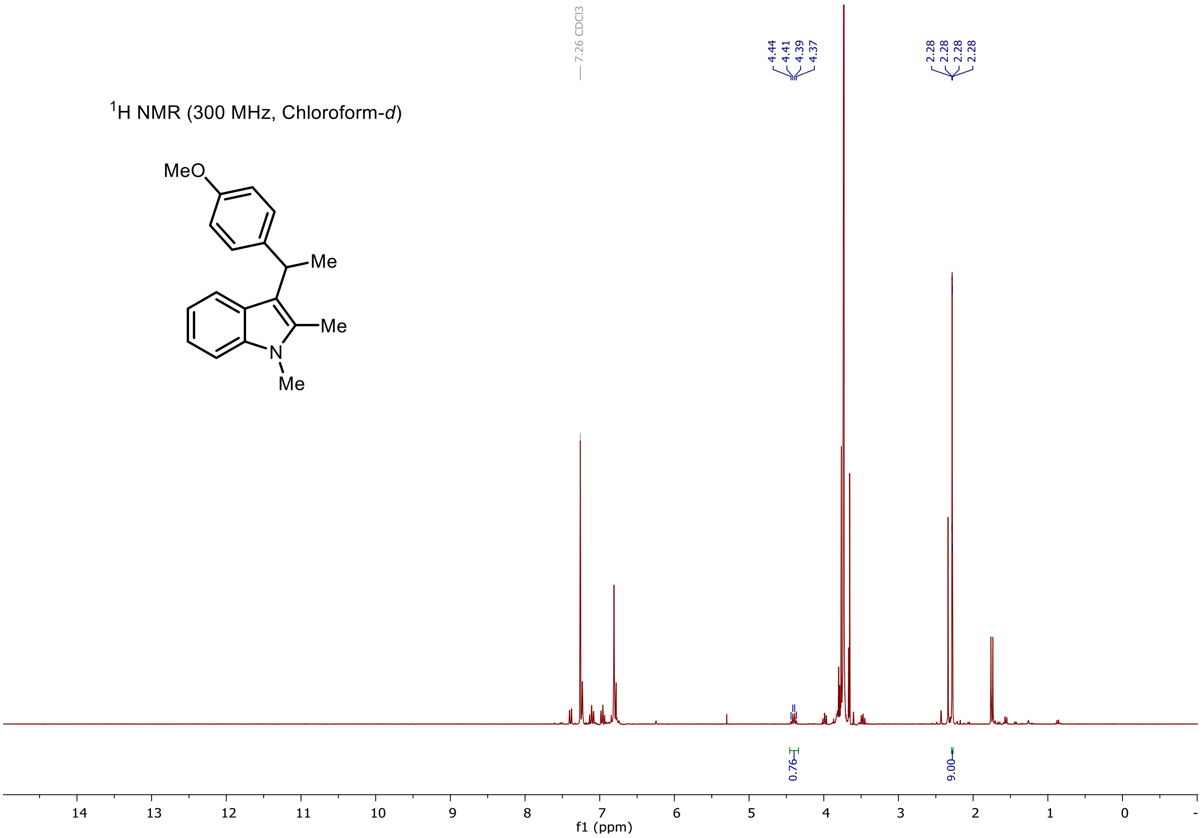

Purification by preparative TLC (eluent = 2.5% EtOAc in petroleum ether) gave the title compound as a brown oil (19 mg, 68%); R_f = 0.41 (eluent = 2.5% EtOAc in petroleum ether); vmax / cm-1 (film) 2980, 2929, 2833, 1608, 1579, 1508, 1469, 1367, 1332, 1242, 1174, 1068, 1033, 945, 829, 734, 561; **^{1}H NMR (500 MHz, Chloroform-*d*)** δ 7.42 (dt, *J* = 8.0, 1.0 Hz, 1H), 7.31 – 7.24 (m, 3H), 7.13 (ddd, *J* = 8.2, 7.0, 1.2 Hz, 1H), 6.99 (ddd, *J* = 8.0, 7.0, 1.1 Hz, 1H), 6.85 – 6.78 (m, 2H), 4.47 – 4.40 (m, 1H), 3.79 (s, 3H), 3.67 (s, 3H), 2.35 (s, 3H), 1.78 (d, *J* = 7.3 Hz, 3H); **^{13}C{^{1}H} NMR (126 MHz, Chloroform-d)** δ 157.6, 138.6, 136.8, 132.5, 128.3, 126.8, 120.4, 119.5, 118.6, 115.8, 113.6, 108.7, 55.3, 35.0, 29.6, 21.1, 10.7; HRMS (CI-QUADRUPOLE) calculated $[C_{19}H_{21}NO]^+$ (M^+) m/z 279.1618, found 279.1620.

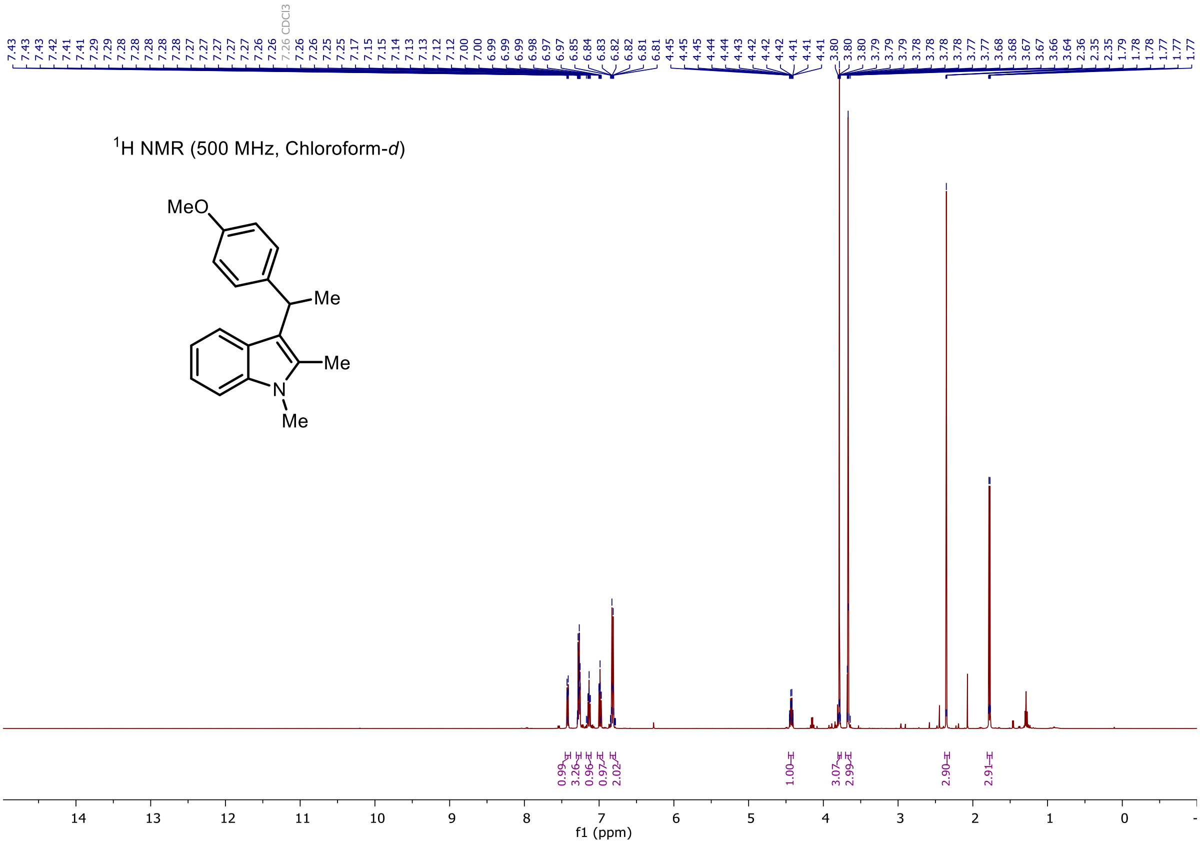
7.43 7.43 7.43 7.42 7.41 7.41 7.29 7.29 7.28 7.28 7.28 7.28 7.28 7.27 7.27 7.27 7.27 7.27 7.26 7.26 CDCl3 7.26 7.26 7.25 7.25 7.17 7.15 7.15 7.14 7.13 7.13 7.12 7.12 7.00 7.00 6.99 6.99 6.99 6.98 6.97 6.97 6.85 6.84 6.83 6.82 6.82 6.81 6.81 4.45 4.45 4.44 4.44 4.43 4.42 4.42 4.42 4.41 4.41 4.41 3.80 3.80 3.80 3.79 3.79 3.79 3.78 3.78 3.78 3.78 3.77 3.77 3.68 3.68 3.67 3.67 3.66 3.64 2.36 2.35 2.35 1.79 1.78 1.78 1.77 1.77 1.77
1H NMR (500 MHz, Chloroform-d)
MeO
Me
Me
N
Me
0.99 3.26 0.96 0.97 2.02 1.00 3.07 2.99 2.90 2.91
14 13 12 11 10 9 8 7 6 5 4 3 2 1 0
f1 (ppm)

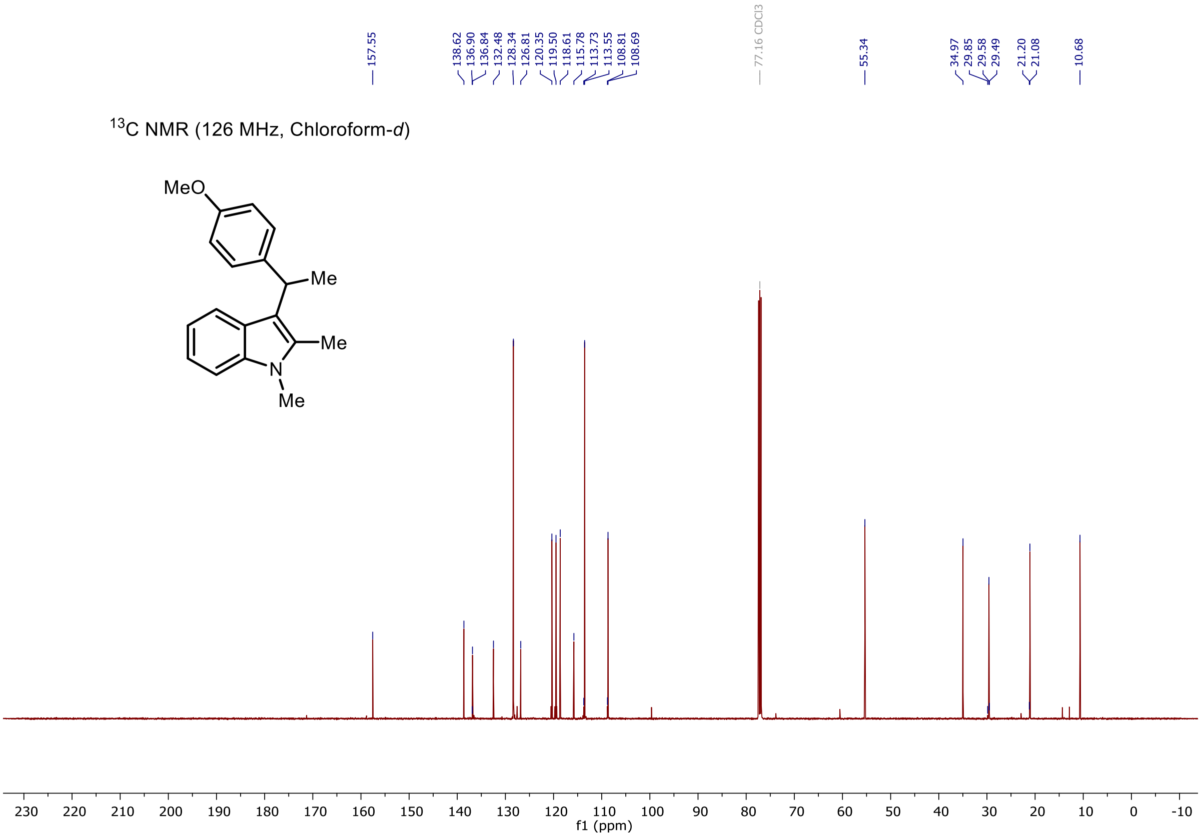
157.55
138.62 136.90 136.84 132.48 128.34 126.81 120.35 119.50 118.61 115.78 113.73 113.55 108.81 108.69
77.16 CDCl3
55.34
34.97 29.85 29.58 29.49 21.20 21.08
10.68
13C NMR (126 MHz, Chloroform-d)
MeO
Me
Me
N
Me
230 220 210 200 190 180 170 160 150 140 130 120 110 100 90 80 70 60 50 40 30 20 10 0 -10
f1 (ppm)

1,2-dimethyl-3-(1-phenylpropyl)-1H-indole (14)

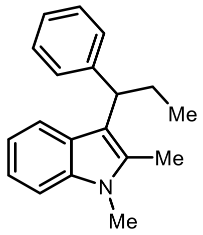

The title compound was prepared according to general procedure 5 using 1,2-dimethylindole (14.5 mg, 0.1 mmol) and 4-methoxy-N-(4-methoxyphenyl)-N-(1-phenylpropyl)aniline (42 mg, 0.12 mmol) for 18 h. Yield determined by crude ^{1}H NMR using 1,3,5-trimethylbenzene as internal standard (51%). HRMS (EI-QUADRUPOLE) calculated $[C_{19}H_{21}N]^{+}$ (M^{+}) m/z 263.1669, found 263.167.

<u>Resolved signals of 1,2-dimethyl-3-(1-phenylpropyl)-1H-indole:</u>

^{1}H NMR (300 MHz, Chloroform-*d*) δ 7.52 (d, *J* = 7.9 Hz, 1H), 7.37 – 7.31 (d, *J* = 7.7 Hz, 2H), 4.09 (dd, *J* = 9.3, 6.5 Hz, 1H), 3.65 (s, 3H), 2.37 (s, 3H), 0.90 (t, *J* = 7.3 Hz, 3H).

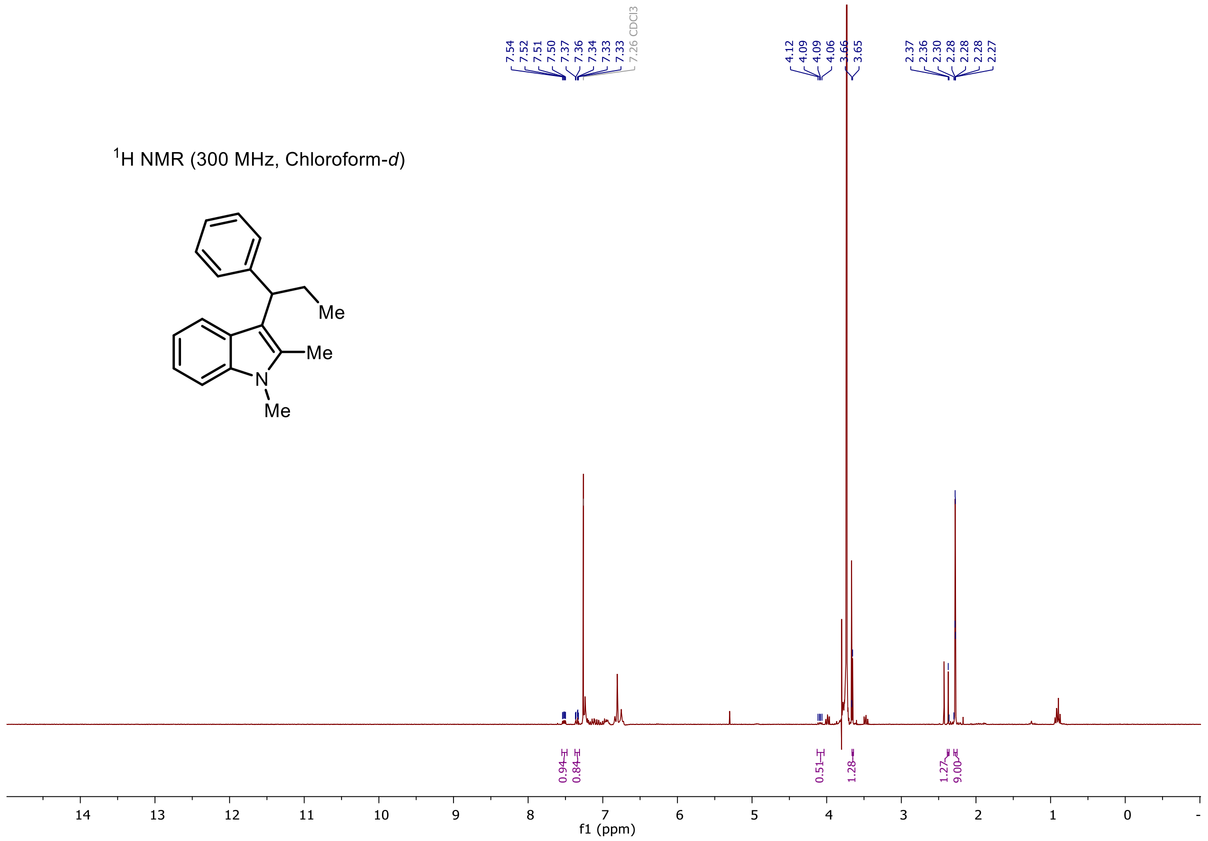

3-(2,3-dihydro-1H-inden-1-yl)-1,2-dimethyl-1H-indole (16)

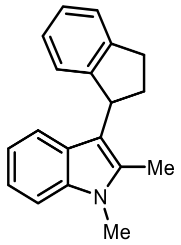

The title compound was prepared according to general procedure 5 using 1,2-dimethylindole (14.5 mg, 0.1 mmol) and N,N-bis(4-methoxyphenyl)-2,3-dihydro-1H-inden-1-amine (41 mg, 0.12 mmol) for 18 h. Yield determined by crude ^{1}H NMR using 1,3,5-trimethylbenzene as internal standard (59%). HRMS (EI-QUADRUPOLE) calculated $[C_{19}H_{19}N]^{+}$ (M^{+}) m/z 261.1512, found 261.1514.

Resolved signals of 3-(2,3-dihydro-1H-inden-1-yl)-1,2-dimethyl-1H-indole:

^{1}H NMR (300 MHz, Chloroform-*d*) δ 7.35 – 7.29 (m, 2H), 7.22 – 7.15 (m, 2H), 7.13 – 7.05 (m, 2H), 4.69 – 4.57 (m, 1H), 3.11 – 2.92 (m, 2H), 2.48 (ddd, *J* = 7.9, 5.0, 2.7 Hz, 2H), 2.35 (s, 3H).

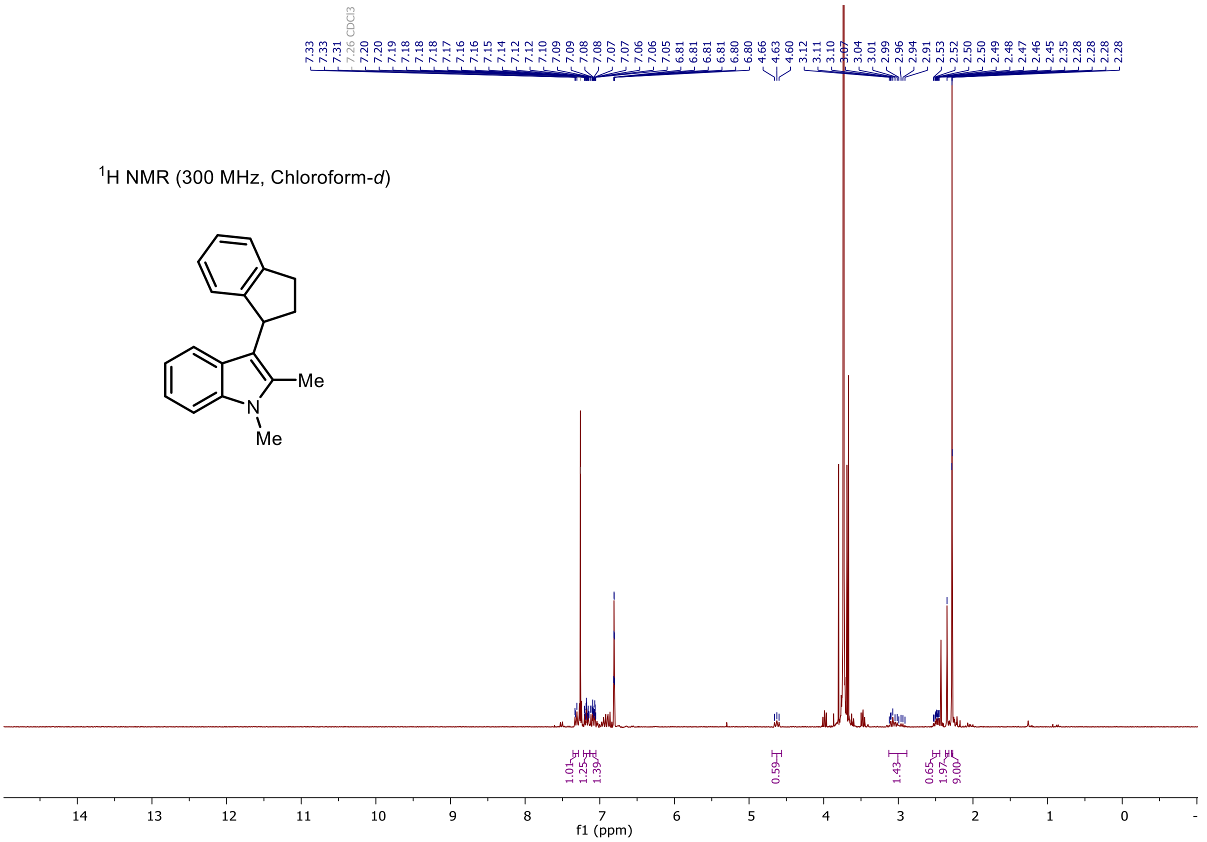

1,2-dimethyl-3-(1,2,3,4-tetrahydronaphthalen-1-yl)-1H-indole (17)

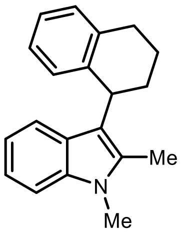

The title compound was prepared according to general procedure 5 using 1,2-dimethylindole (14.5 mg, o.1 mmol) and N,N-bis(4-methoxyphenyl)-1,2,3,4-tetrahydronaphthalen-1-amine (43.9 mg, 0.12 mmol) for 18 h. Yield determined by crude ^{1}H NMR using 1,3,5-trimethylbenzene as internal standard (61%). HRMS (EI-QUADRUPOLE) calculated $[C_{20}H_{21}N]^+$ (M^+) m/z 275.1669, found 275.167.

<u>Resolved signals of 1,2-dimethyl-3-(1,2,3,4-tetrahydronaphthalen-1-yl)-1H-indole:</u>

^{1}H NMR (300 MHz, Chloroform-*d*) δ 7.25 – 7.22 (m, 1H), 7.18 – 7.00 (m, 3H), 4.35 (t, *J* = 7.9 Hz, 1H), 3.03 – 2.79 (m, 2H), 2.14 – 1.78 (m, 4H).

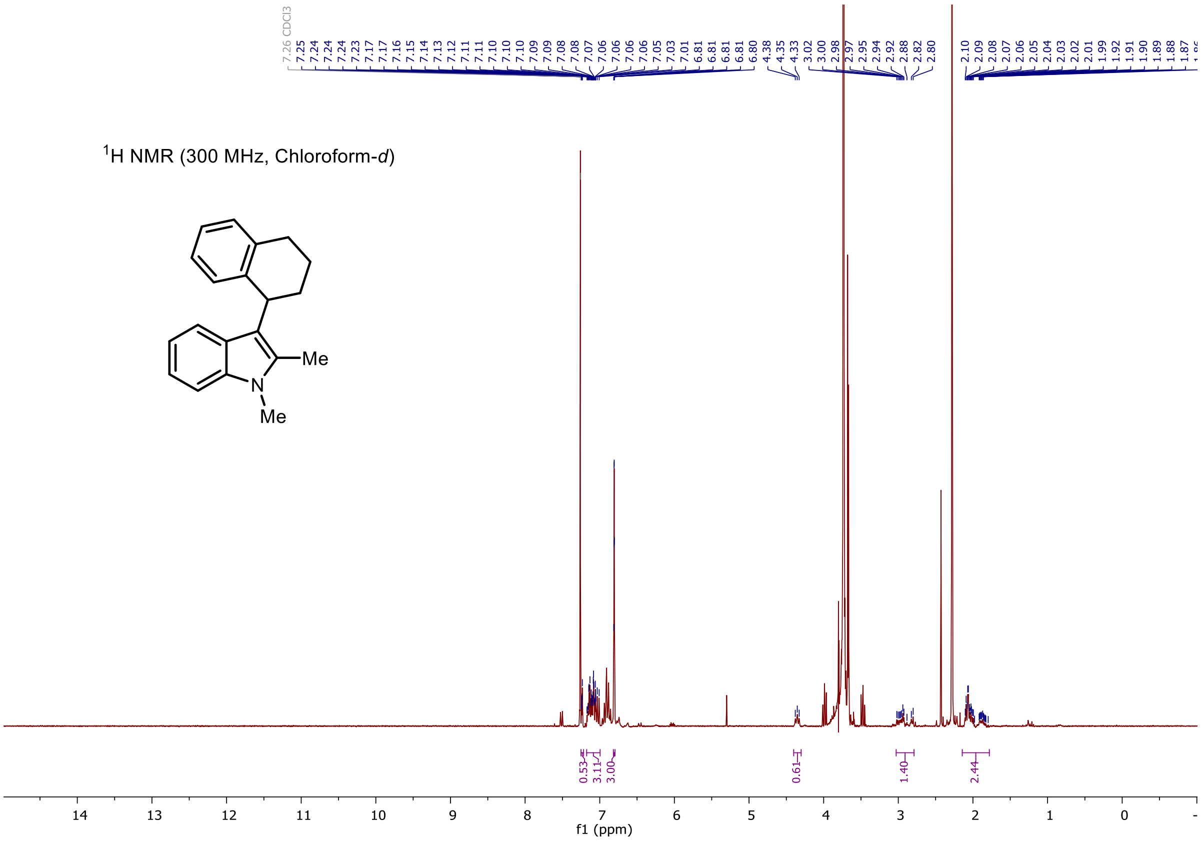

1,2,5-trimethyl-3-(1-phenylethyl)-1H-indole (18)

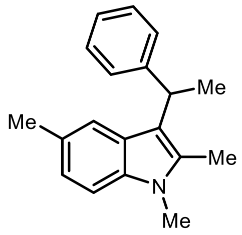

The title compound was prepared according to general procedure 5 using 1,2,5-trimethylindole (16 mg, 0.1 mmol) and 4-methoxy-N-(4-methoxyphenyl)-N-(1-phenylethyl)aniline (40 mg, 0.12 mmol) for 18. Yield determined by crude ^{1}H NMR using 1,3,5-trimethylbenzene as internal standard (40%). HRMS (EI-QUADRUPOLE) calculated $[C_{19}H_{21}N]^+$ (M^+) m/z 263.1669, found 263.1667.

<u>Resolved signals of 1,2,5-trimethyl-3-(1-phenylethyl)-1H-indole:</u>

^{1}H NMR (300 MHz, Chloroform-*d*) δ 7.40 – 7.37 (m, 1H), 4.43 (q, *J* = 7.3 Hz, 1H), 2.37 (d, *J* = 0.7 Hz, 3H), 1.77 (d, *J* = 7.3 Hz, 3H).

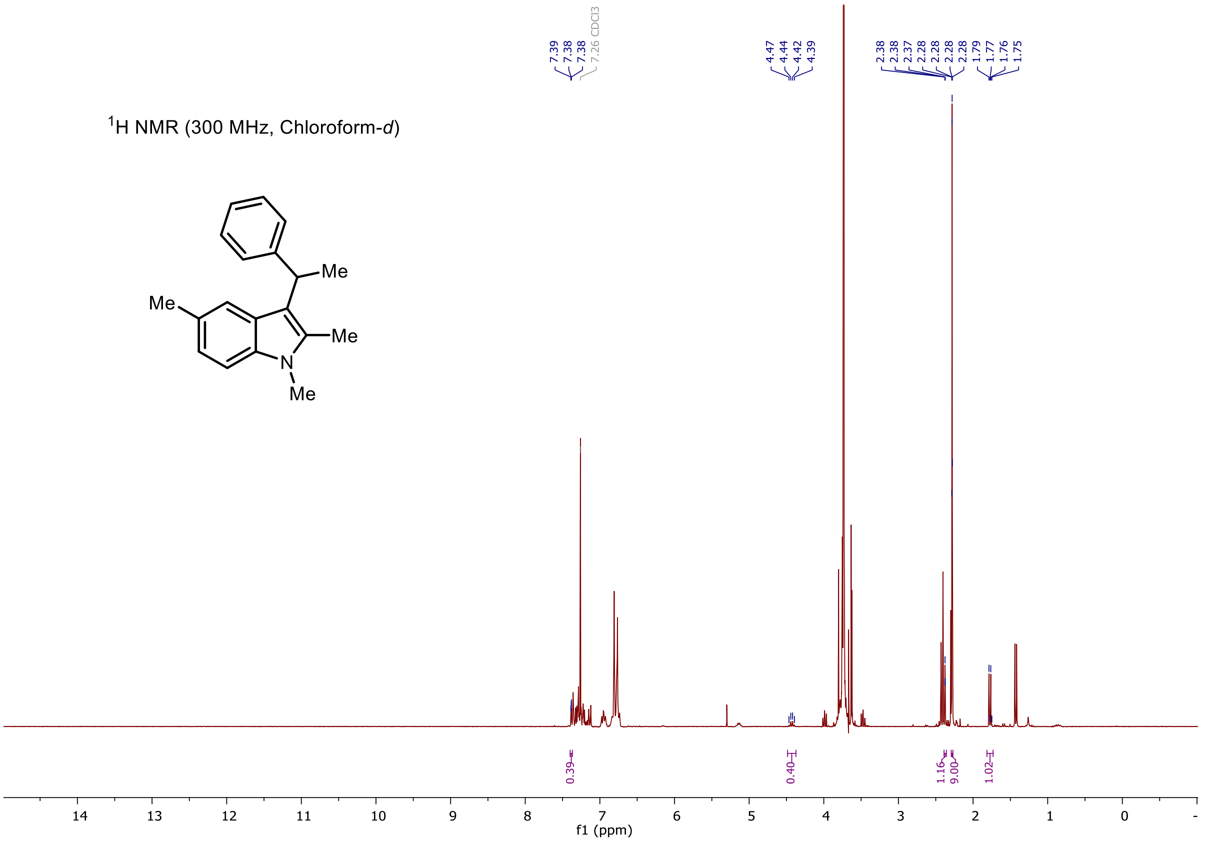

5-bromo-1,2-dimethyl-3-(1-phenylethyl)-1H-indole (19)

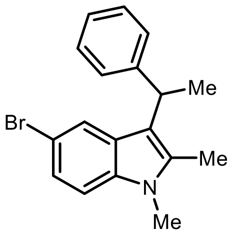

The title compound was prepared according to general procedure 5 using 5-bromo-1,2-dimethylindole (22 mg, 0.1 mmol) and 4-methoxy-N-(4-methoxyphenyl)-N-(1-phenylethyl)aniline (40 mg, 0.12 mmol) for 18 h. Yield determined by crude ^{1}H NMR using 1,3,5-trimethylbenzene as internal standard (60%). HRMS (EI-QUADRUPOLE) calculated $[C_{18}H_{18}N^{79}Br]^+$ (M^+) m/z 327.0617, found 327.0620.

<u>Resolved signals of 5-bromo-1,2-dimethyl-3-(1-phenylethyl)-1H-indole:</u>

^{1}H NMR (300 MHz, Chloroform-*d*) δ 7.52 (d, *J* = 1.8 Hz, 1H), 4.40 (q, *J* = 7.4 Hz, 1H), 2.32 (s, 3H), 1.75 (d, *J* = 7.3 Hz, 3H).

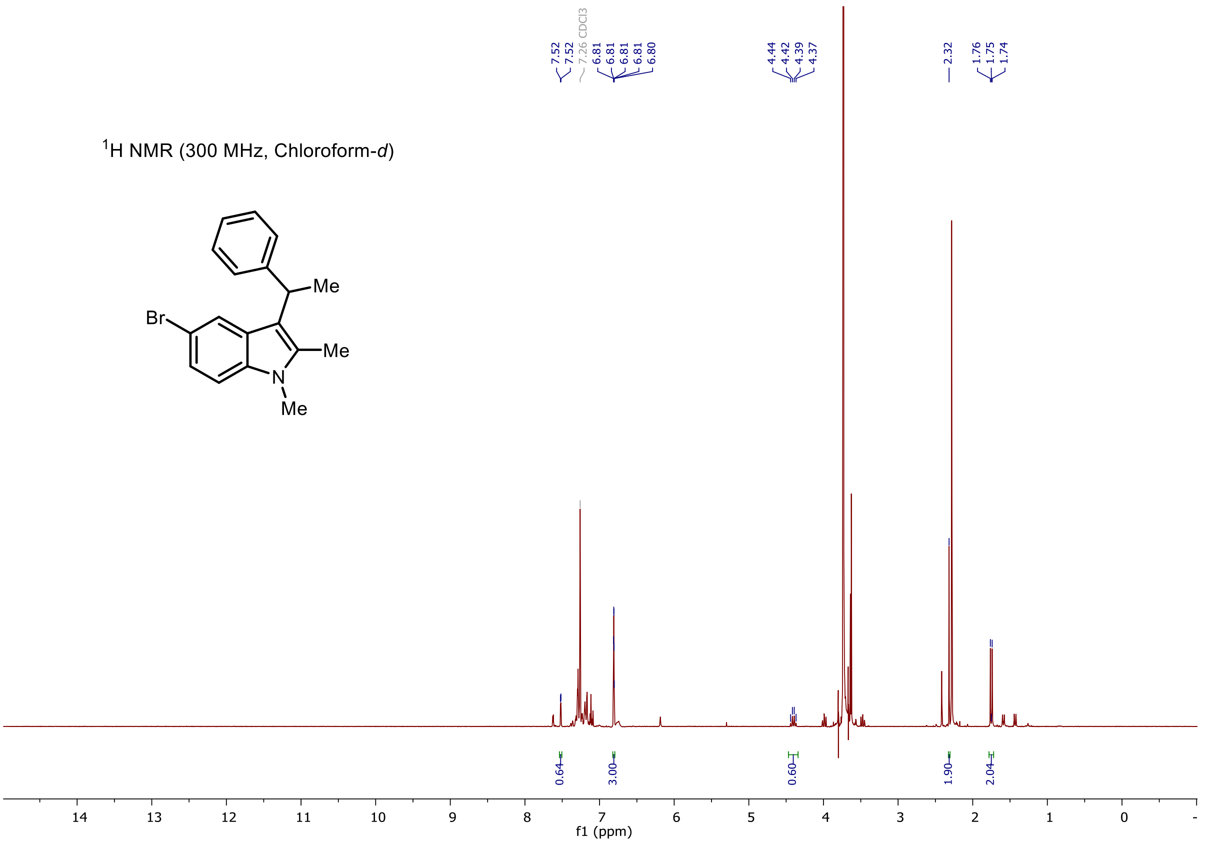

5-Chloro-1,2-dimethyl-3-(1-phenylethyl)-1H-indole (20)

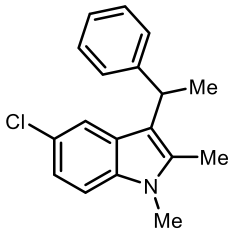

The title compound was prepared according to general procedure 5 using 5-chloro-1,2-dimethylindole (18 mg, 0.1 mmol) and 4-methoxy-N-(4-methoxyphenyl)-N-(1-phenylethyl)aniline (40 mg, 0.12 mmol) for 18 h. Yield determined by crude ^{1}H NMR using 1,3,5-trimethylbenzene as internal standard (61%). HRMS (EI-QUADRUPOLE) calculated $[C_{18}H_{18}N^{35}Cl]^+$ (M^+) m/z 283.1122, found 283.1124.

<u>Resolved signals of 5-Chloro-1,2-dimethyl-3-(1-phenylethyl)-1H-indole:</u>

^{1}H NMR (300 MHz, Chloroform-*d*) δ 7.37 – 7.34 (m, 1H), 4.40 (q, *J* = 7.3 Hz, 1H), 3.63 (s, 2H), 2.32 (s, 3H), 1.75 (d, *J* = 7.3 Hz, 3H).

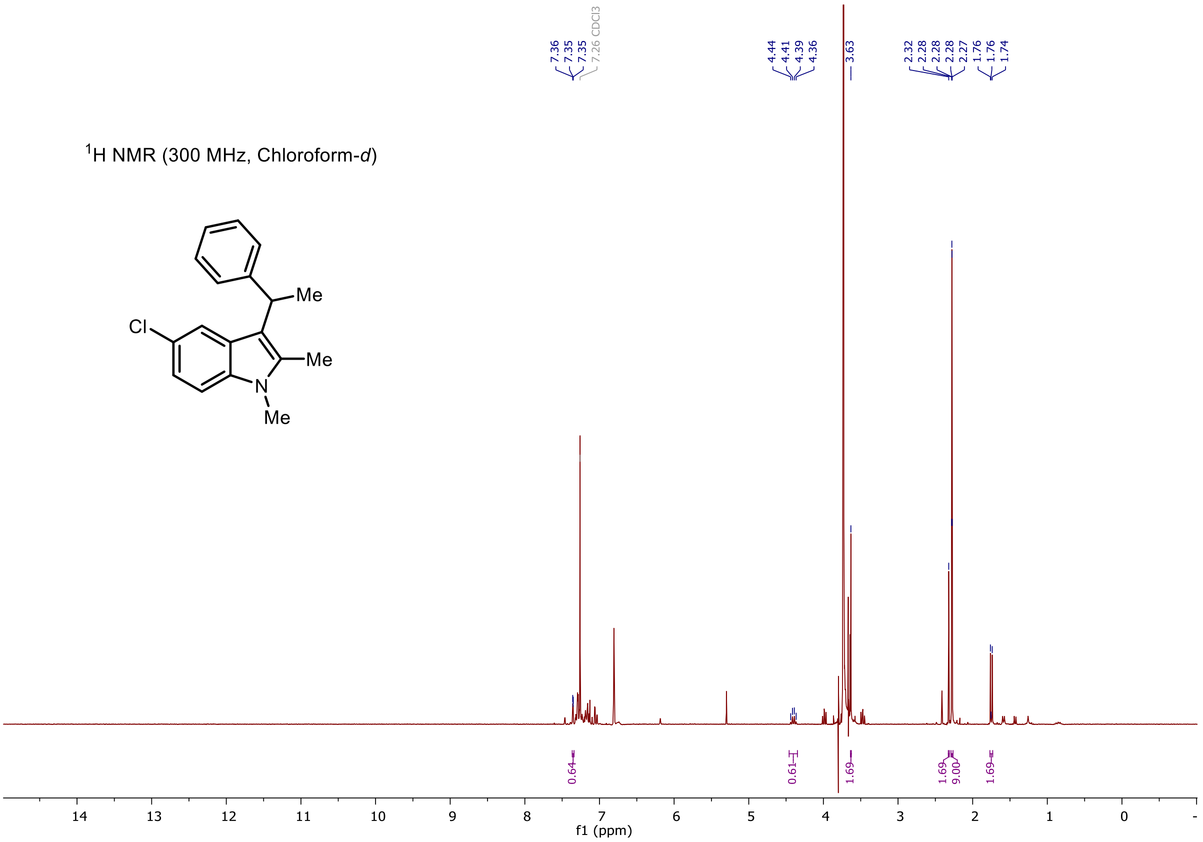

5-methoxy-1,2-dimethyl-3-(1-phenylethyl)-1H-indole (21)

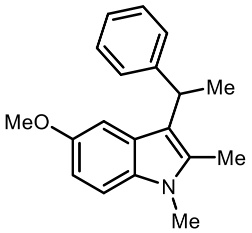

The title compound was prepared according to general procedure 5 using 5-methoxy-1,2-dimethylindole (18 mg, 0.1 mmol) and 4-methoxy-N-(4-methoxyphenyl)-N-(1-phenylethyl)aniline (40 mg, 0.12 mmol) for 18 h. Yield determined by crude ^{1}H NMR using 1,3,5-trimethylbenzene as internal standard (31%). HRMS (EI-QUADRUPOLE) calculated $[C_{19}H_{21}ON]^{+}$ (M^{+}) m/z 279.1618, found 279.162.

<u>Resolved signals of 5-methoxy-1,2-dimethyl-3-(1-phenylethyl)-1H-indole:</u>

^{1}H NMR (300 MHz, Chloroform-*d*) δ 7.31 (t, *J* = 1.6 Hz, 1H), 7.21 – 7.19 (m, 1H), 5.14 (q, *J* = 7.0 Hz, 1H), 4.42 (q, *J* = 7.3 Hz, 1H), 3.62 (s, 3H), 2.31 (s, 3H), 1.76 (d, *J* = 7.3 Hz, 3H).

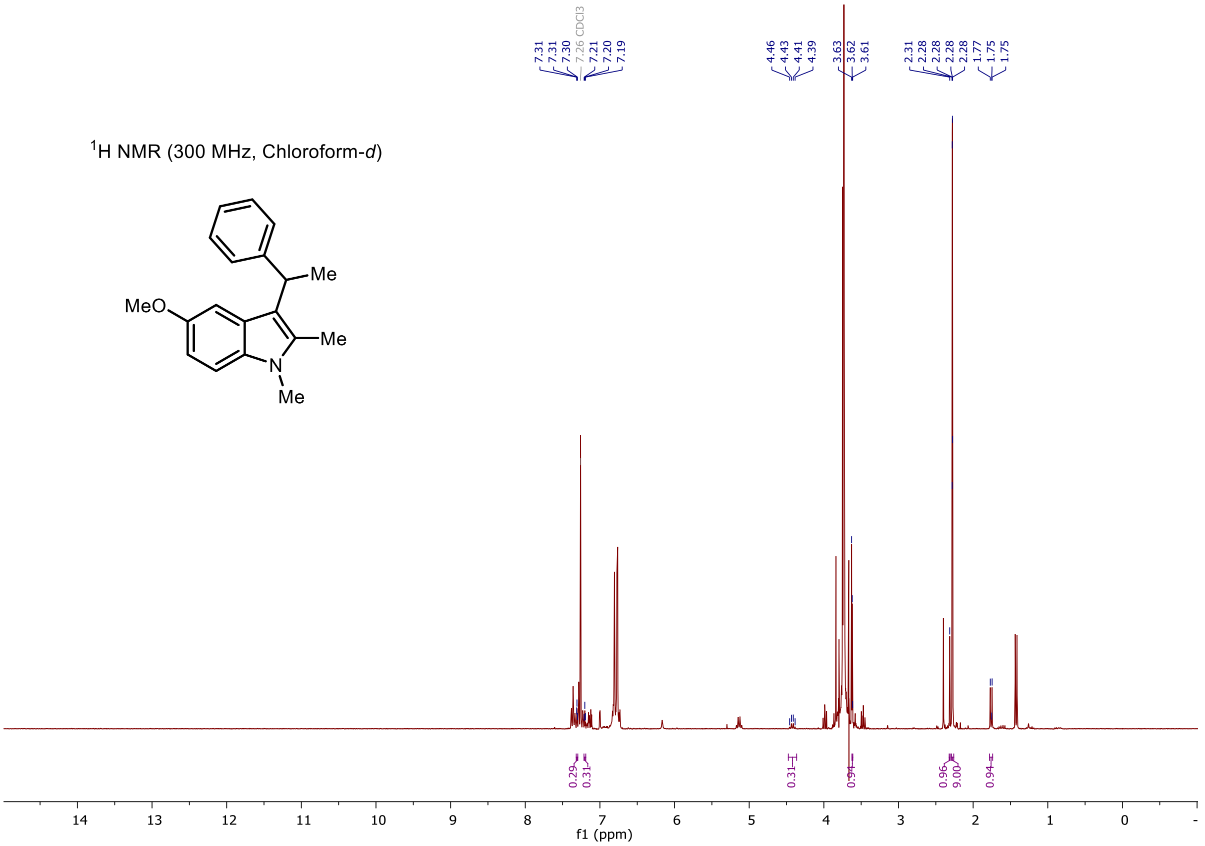

6-methoxy-1,2-dimethyl-3-(1-phenylethyl)-1H-indole (22)

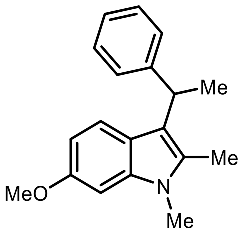

The title compound was prepared according to general procedure 5 using 6-methoxy-1,2-dimethylindole (18 mg, 0.1 mmol) and 4-methoxy-N-(4-methoxyphenyl)-N-(1-phenylethyl)aniline (40 mg, 0.12 mmol) for 24 h. Yield determined by crude ^{1}H NMR using 1,3,5-trimethylbenzene as internal standard (38%). HRMS (EI-QUADRUPOLE) calculated $[C_{19}H_{21}ON]^+$ (M^+) m/z 279.1618, found 279.1619.

<u>Resolved signals of 6-methoxy-1,2-dimethyl-3-(1-phenylethyl)-1H-indole:</u>

^{1}H NMR (300 MHz, Chloroform-*d*) δ 6.63 (dd, *J* = 8.7, 2.3 Hz, 1H), 4.40 (q, *J* = 7.3 Hz, 1H), 3.85 (s, 3H), 2.32 (s, 3H), 1.76 (d, *J* = 7.3 Hz, 3H).

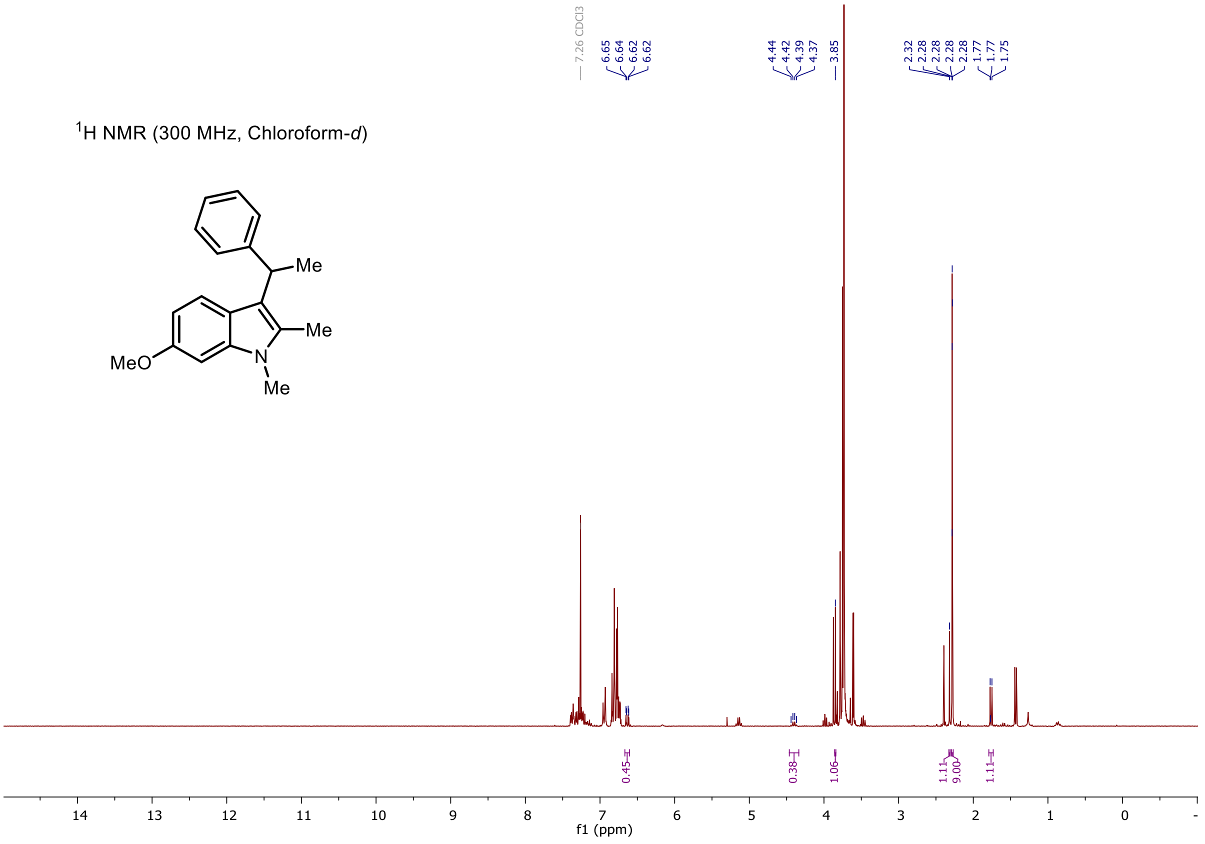

1-methyl-2-phenyl-3-(1-phenylethyl)-1H-indole (24)

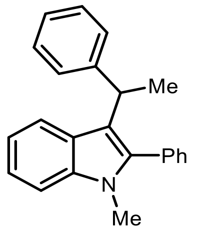

The title compound was prepared according to general procedure 5 using 1-methyl-2-phenylindole (21 mg, 0.1 mmol) and 4-methoxy-N-(4-methoxyphenyl)-N-(1-phenylethyl)aniline (40 mg, 0.12 mmol) for 18 h. Yield determined by crude ^{1}H NMR using 1,3,5-trimethylbenzene as internal standard: 75 %

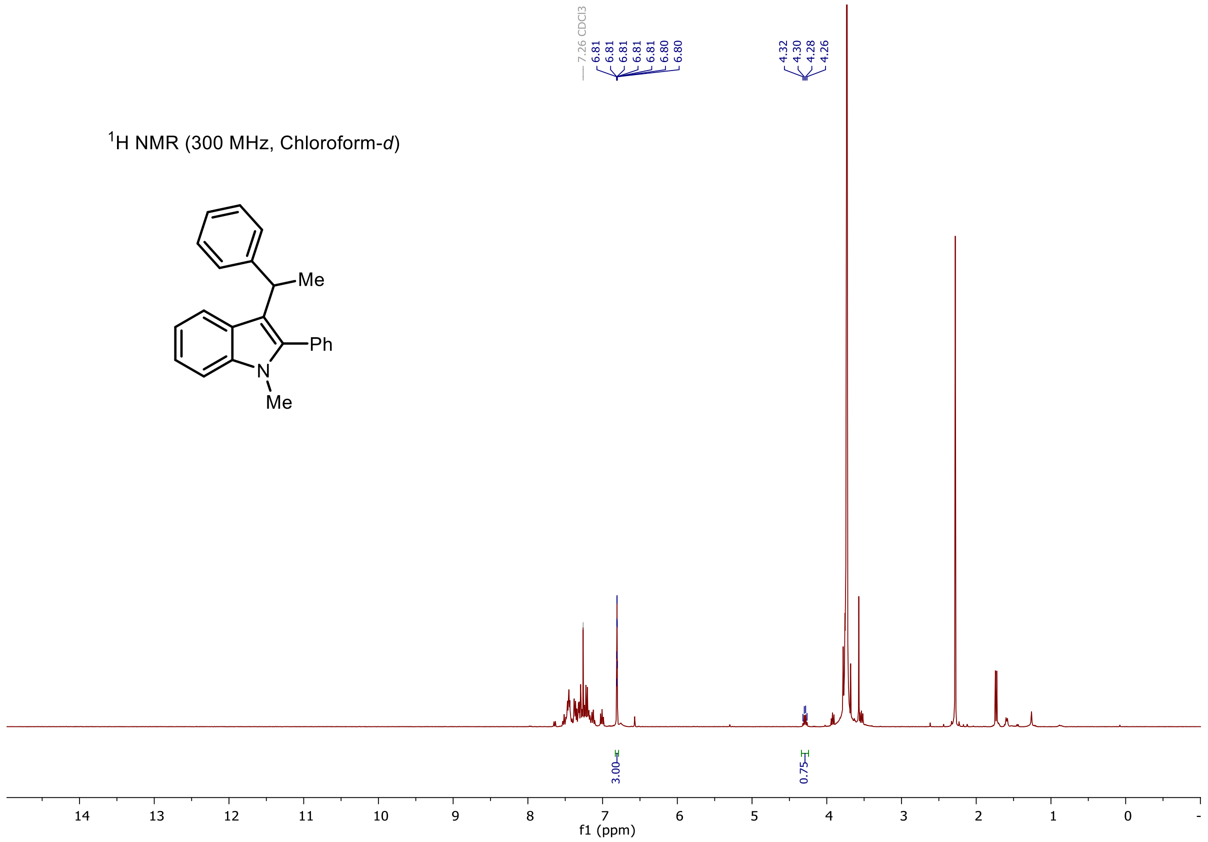

A part of the product was isolated using preparative TLC (eluent = 2.5% EtOAc in petroleum ether) as a brown oil; R_f = 0.51 (eluent = 2.5% EtOAc in petroleum ether); vmax / cm-1 (film) 2980, 1543, 1153, 1072, 952, 740, 700, 418; **^{1}H NMR (500 MHz, Chloroform-*d*)** δ 7.47 – 7.42 (m, 4H), 7.38 – 7.35 (m, 2H), 7.33 (dt, *J* = 8.2, 0.9 Hz, 1H), 7.32 – 7.28 (m, 2H), 7.25 – 7.18 (m, 3H), 7.15 – 7.10 (m, 1H), 7.01 (ddd, *J* = 8.0, 7.0, 1.0 Hz, 1H), 4.29 (q, *J* = 7.3 Hz, 1H), 3.57 (s, 3H), 1.73 (d, *J* = 7.3 Hz, 3H); **^{13}C{^{1}H} NMR (126 MHz, Chloroform-*d*)** δ 146.5, 137.9, 137.6, 132.4, 131.0, 128.5, 128.3, 128.2, 127.5, 126.5, 125.6, 121.6, 120.9, 119.1, 117.4, 109.5, 35.9, 30.9, 21.2; HRMS (CI-QUADRUPOLE) calculated $[C_{23}H_{21}N]^+$ (M^+) m/z 311.1669, found 311.1669.

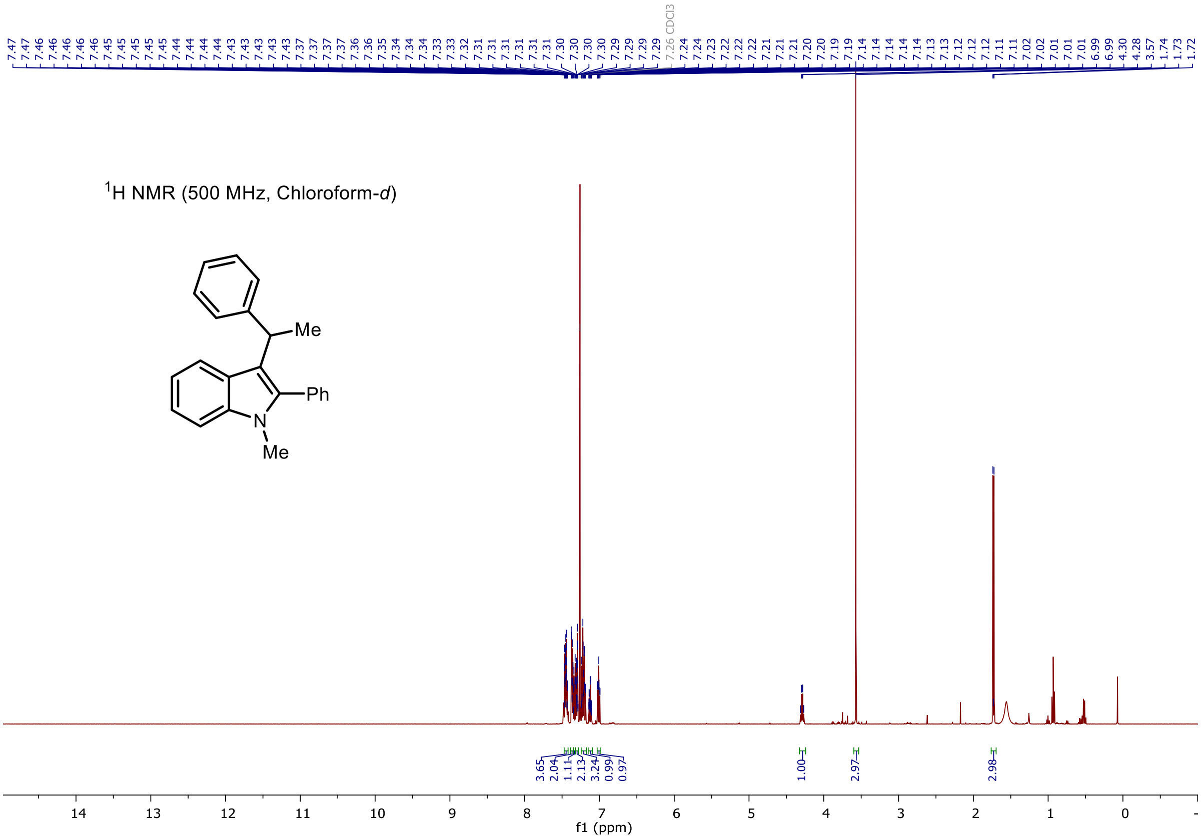

1H NMR (500 MHz, Chloroform-d)
Me
Ph
N
Me
f1 (ppm)

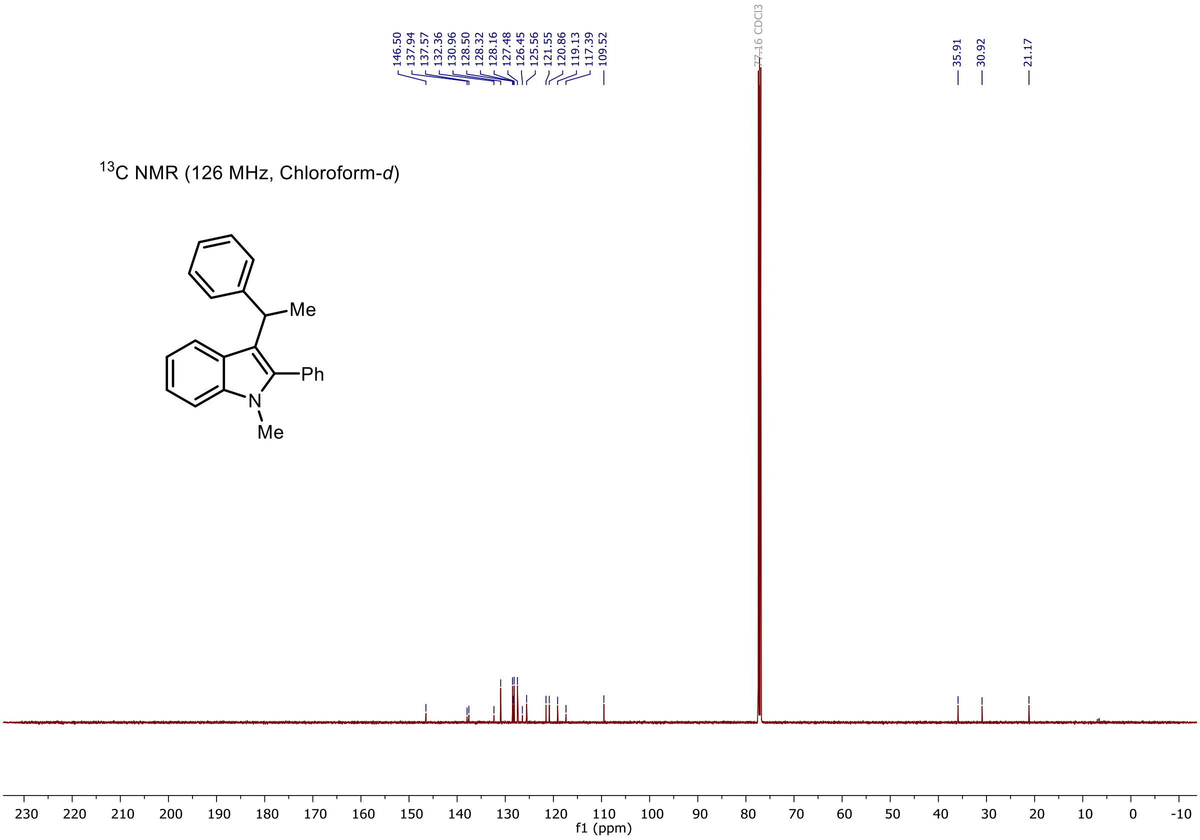

13C NMR (126 MHz, Chloroform-d)
Me
Ph
N
Me
146.50
137.94
137.57
132.36
130.96
128.50
128.32
128.16
127.48
126.45
125.56
121.55
120.86
119.13
117.39
109.52
77.16 CDCl3
35.91
30.92
21.17
f1 (ppm)

3-(1-(4-methoxyphenyl)ethyl)-2-methyl-1H-indole (26)

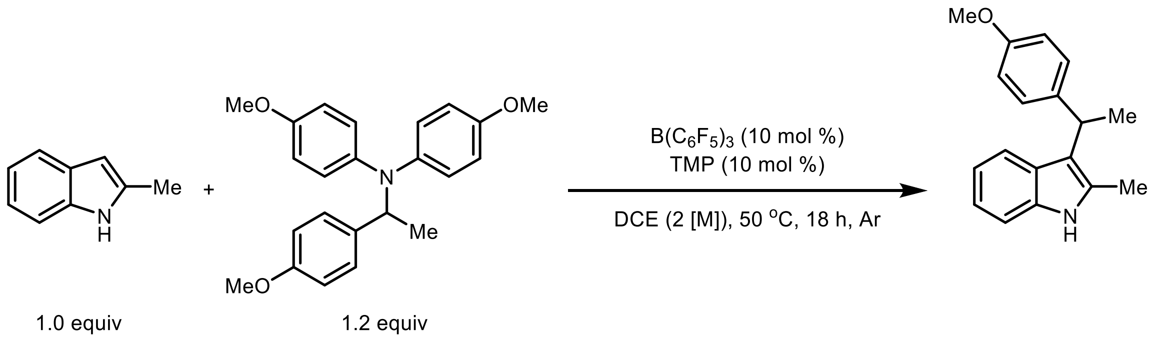

In an argon-filled glove box, a 10 mL vial equipped with a magnetic stirrer bar was charged with the 2-methylindole (13 mg, 0.1 mmol) and 4-methoxy-N-(4-methoxyphenyl)-N-(1-(4-methoxyphenyl)ethyl)aniline (43.6 mg , 0.12 mmol) followed by 2,2,6,6-tetramethylpiperidine (TMP) (1.7 µl, 0.01 mmol), $B(C_6F_5)_3$ (5 mg, 0.01 mmol) and DCE (50 µL). The vial was sealed with an aluminium crimped cap and was left to stir at 50 °C for 24 h. Yield determined by crude ^{1}H NMR using 1,3,5-trimethylbenzene as internal standard: 77 %

<u>Resolved signals of 3-(1-(4-methoxyphenyl)ethyl)-2-methyl-1H-indole:</u>[16]

^{1}H NMR (300 MHz, Chloroform-*d*) δ 7.37 (d, *J* = 7.9 Hz, 1H), 7.10 – 7.02 (m, 1H), 6.96 (ddd, *J* = 8.2, 7.1, 1.1 Hz, 1H), 4.37 (q, *J* = 7.3 Hz, 1H), 2.34 (s, 3H), 1.74 (d, *J* = 7.3 Hz, 3H).

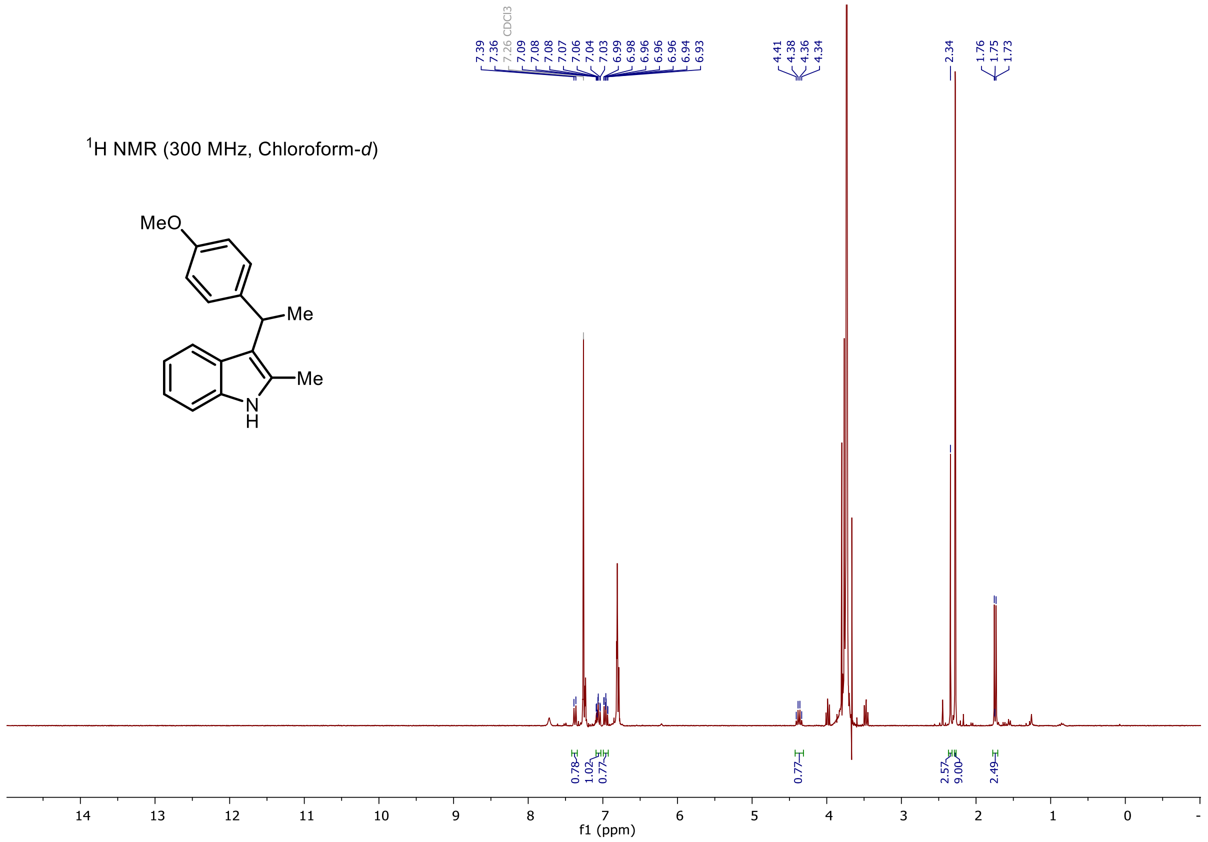

(4-chlorophenyl)(5-methoxy-3-(1-(4-methoxyphenyl)ethyl)-2-methyl-1H-indol-1-yl)methanone (27)

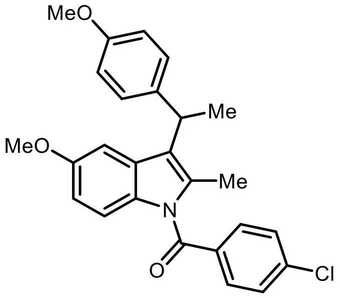

The title compound was prepared according to general procedure 5 using (4-chlorophenyl)(5-methoxy-2-methyl-1H-indol-1-yl)methanone (30 mg, 0.1 mmol) and 4-methoxy-N-(4-methoxyphenyl)-N-(1-(4-methoxyphenyl)ethyl)aniline (43.6 mg, 0.12 mmol) for 24 h. Yield determined by crude ^{1}H NMR using 1,3,5-trimethylbenzene as internal standard (26%). HRMS (ESI-TOF) calculated $[C_{26}H_{25}NO_3Cl]^+$ $(M+H)^+$ m/z 434.1523, found 434.1524.

Resolved signals of (4-chlorophenyl)(5-methoxy-3-(1-(4-methoxyphenyl)ethyl)-2-methyl-1H-indol-1-yl)methanone:

^{1}H NMR (300 MHz, Chloroform-*d*) δ 6.59 (dd, *J* = 9.0, 2.5 Hz, 1H), 4.38 (q, *J* = 7.3 Hz, 1H), 2.35 (s, 2H), 1.74 (d, *J* = 7.4 Hz, 2H).

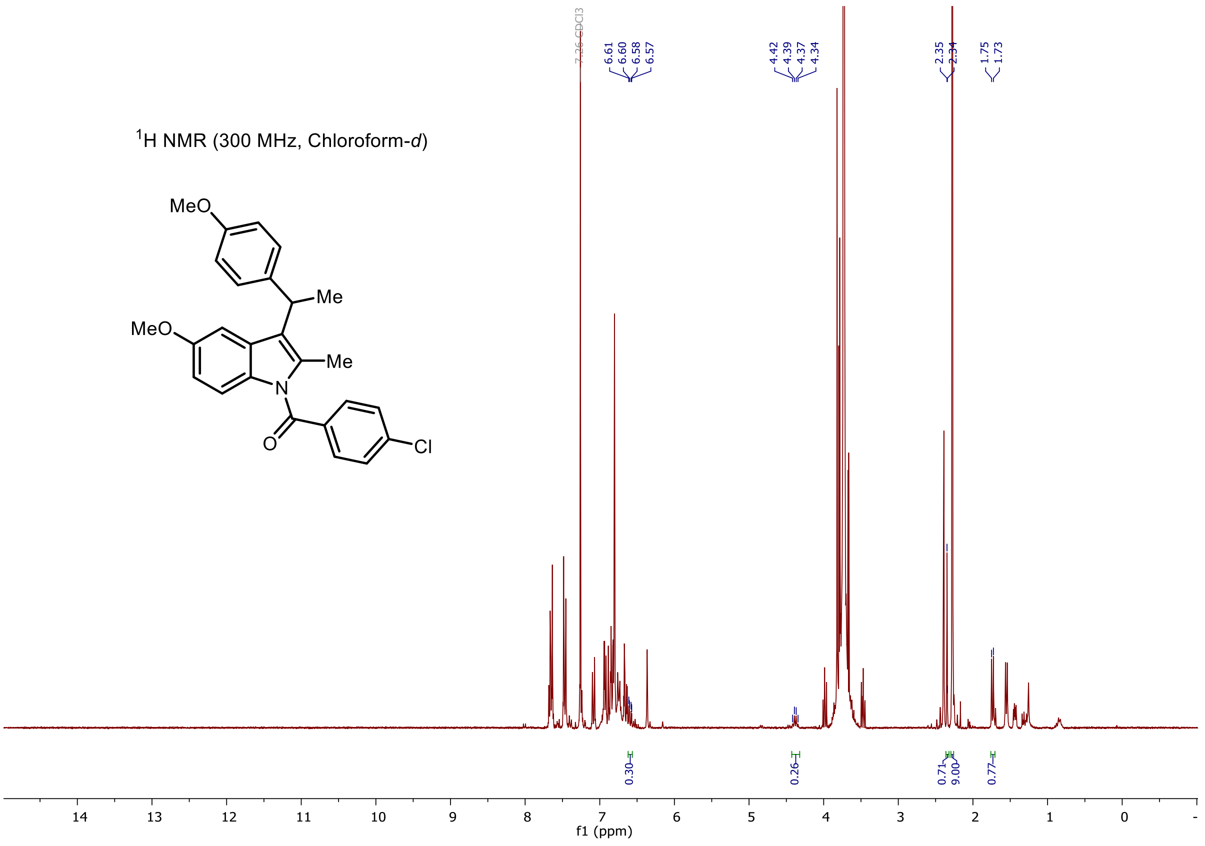

2.7. The use of $H_2O{\cdot}B(C_6F_5)_3$ in the borane-catalyzed alkylation of indoles

2.7.1. General procedure 6: C3 alkylation of indoles

The following procedure was used to prepare active $B(C_6F_5)_3$ to be used directly in the fume cupboard rather than sublimation and using the glovebox.

An oven-dried microwave vial equipped with a stir bar and sealed with a septa was cooled under vacuum and charged with N_2 gas. $B(C_6F_5)_3$ (5 mg, 0.01 mmol) as received from the supplier was added to the microwave vial quickly and the atmosphere cycled three times via vacuum-N_2 backfills. Using 100 µl glass syringe, anhydrous DCE (50 µl, degassed using thaw-freeze cycle) was added and the mixture was stirred for 5 min. Then upon the addition of Et_3SiH (3.2 µl, 0.02 mmol), effervescence was observed and ceased within 15 seconds and the mixture was further stirred for 5 minutes.

Meanwhile, Indole or oxindole (0.1 mmol) and alkylating agent (0.12 mmol) were weighed into a vial and sealed with an aluminium cap. The atmosphere in the vial was cycled three times via vacuum-N_2 backfills. Using 100 µl glass syringe, active $B(C_6F_5)_3$ solution (50 µl) was subsequently added to the indole/alkylating agent mixture. The mixture was stirred for the stated time at 50 °C. An NMR spectroscopic yield was obtained using 1,3,5-trimethylbenzene as an internal standard.

Typically, an excess of active BCF solution is prepared where the amounts for two extra reactions are measured and only the required volume for the number of the reactions undertaken is used while the remaining is discarded.

1,2-dimethyl-3-(1-phenylethyl)-1H-indole (2)

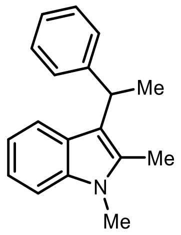

The title compound was prepared according to general procedure 6 using 1,2-dimethylindole (14.5 mg, 0.1 mmol), 4-methoxy-N-(4-methoxyphenyl)-N-(1-phenylethyl)aniline (40 mg, 0.12 mmol), $B(C_6F_5)_3$ (5 mg, 0.01 mmol) and Et_3SiH (3.2 µL, 0.02 mmol) in DCE (50 µL) at 50 ° C for 18 h and obtained in 75% spectroscopic yield.

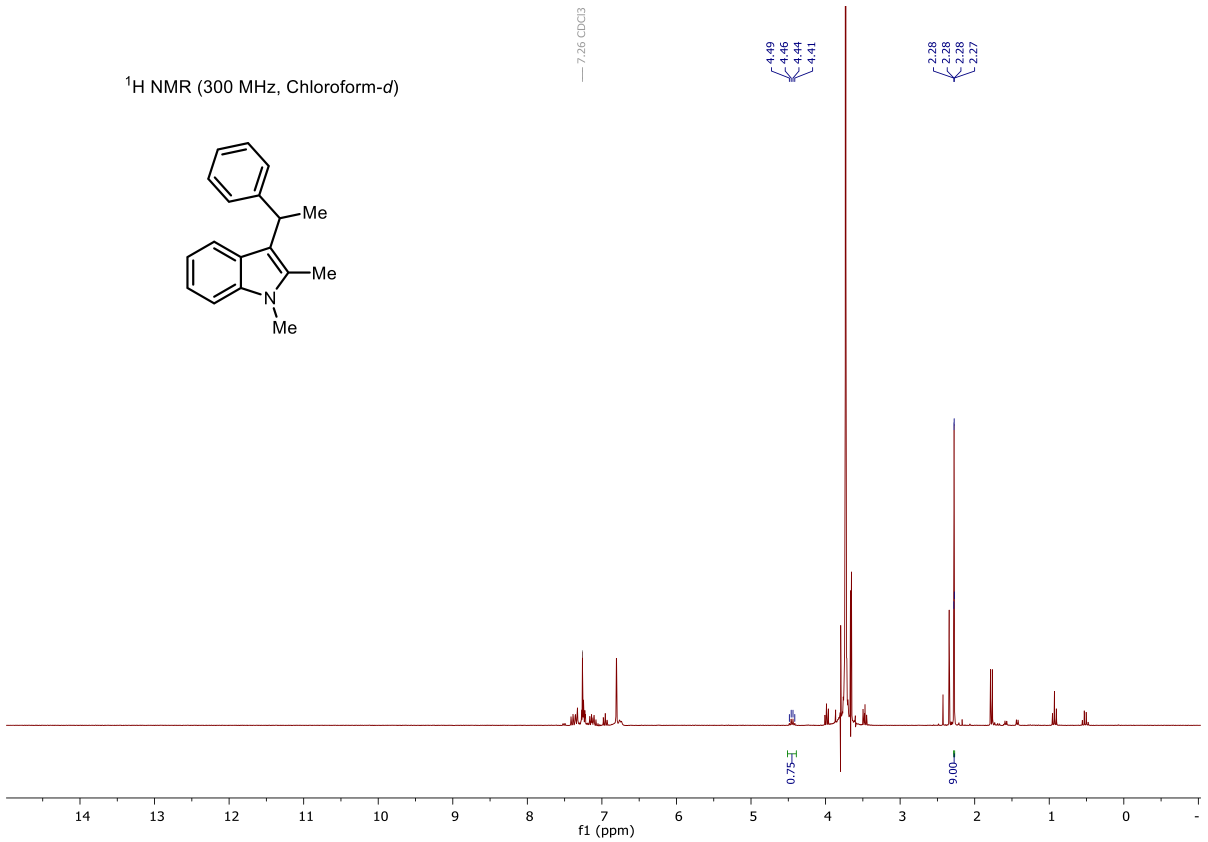

3-(1-(4-methoxyphenyl)ethyl)-1-methyl-1H-indole (25)

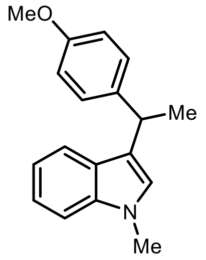

The title compound was prepared according to general procedure 6 using 1-methylindole (13 mg, 0.1 mmol), 4-methoxy-N-(4-methoxyphenyl)-N-(1-(4-methoxyphenyl)ethyl)aniline (43.6 mg , 0.12 mmol), $B(C_6F_5)_3$ (5 mg, 0.01 mmol) and Et_3SiH (3.2 µL, 0.02 mmol) in DCE (50 µL) at 50 ° C for 18 h. Yield determined by crude ^{1}H NMR using 1,3,5-trimethylbenzene as internal standard: 66 %

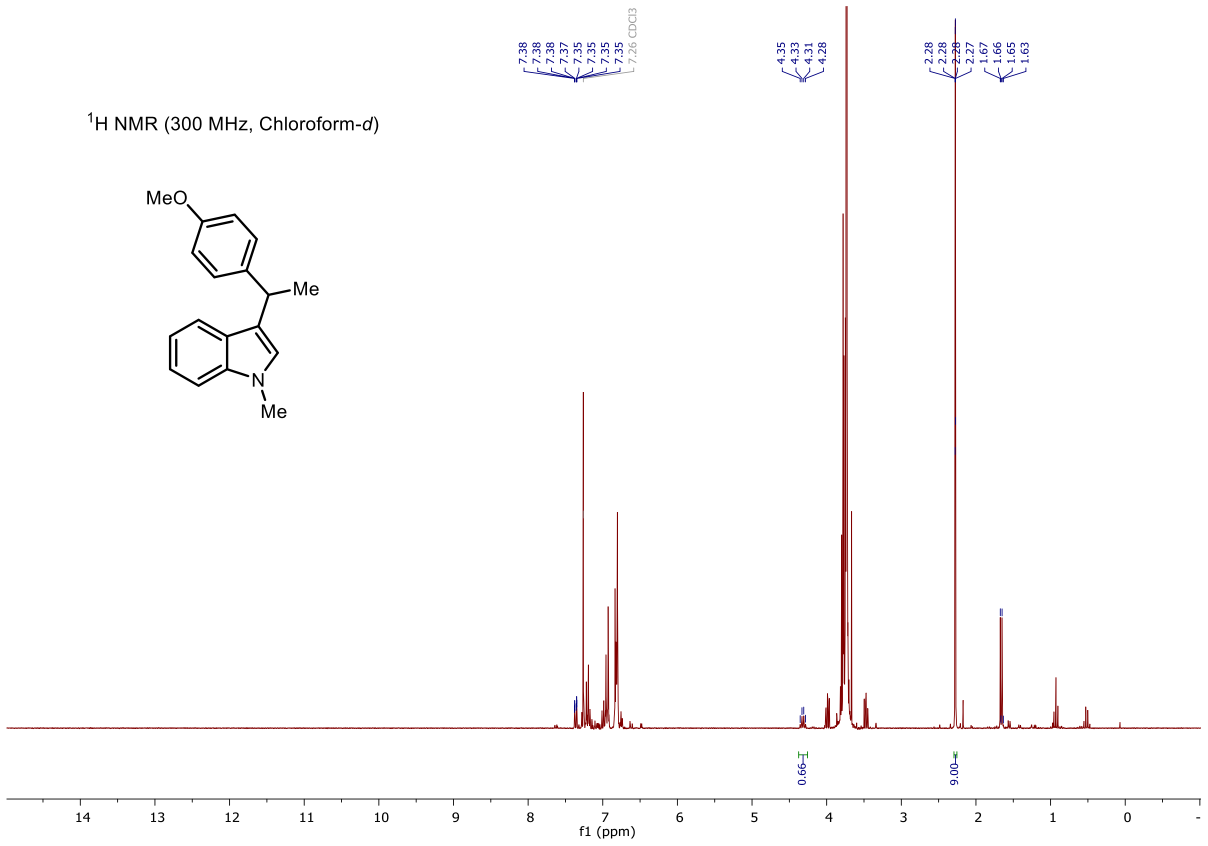

A part of the product was isolated using preparative TLC (eluent = 2.5% EtOAc in petroleum ether) as a brown oil; R_f = 0.2 (eluent =2.5% EtOAc in petroleum ether); vmax / cm-1 (film) 2980, 1610, 1543, 1508, 1473, 1242, 1174, 1155, 1033, 954, 831, 740, 418; **^{1}H NMR (500 MHz, Chloroform-*d*)** δ 7.37 (ddd, *J* = 8.0, 1.2, 0.7 Hz, 1H), 7.29 – 7.25 (m, 1H), 7.23 – 7.20 (m, 2H), 7.20 – 7.15 (m, 1H), 7.00 (ddd, *J* = 8.0, 7.0, 1.0 Hz, 1H), 6.84 – 6.79 (m, 3H), 4.38 – 4.27 (m, 1H), 3.78 (s, 3H), 3.75 (s, 3H), 1.67 (d, *J* = 7.1 Hz, 3H); **^{13}C{^{1}H} NMR (126 MHz, Chloroform-*d*)** δ 157.8, 139.3, 137.4, 128.4, 127.3, 126.0, 121.6, 120.5, 119.9, 118.7, 113.8, 109.2, 55.4, 36.2, 32.8, 22.8; HRMS (CI-QUADRUPOLE) calculated $[C_{18}H_{19}ON]^+$ (M^+) m/z 265.1461, found 265.1460. The spectroscopic data are in accordance with those described in the literature.[17]

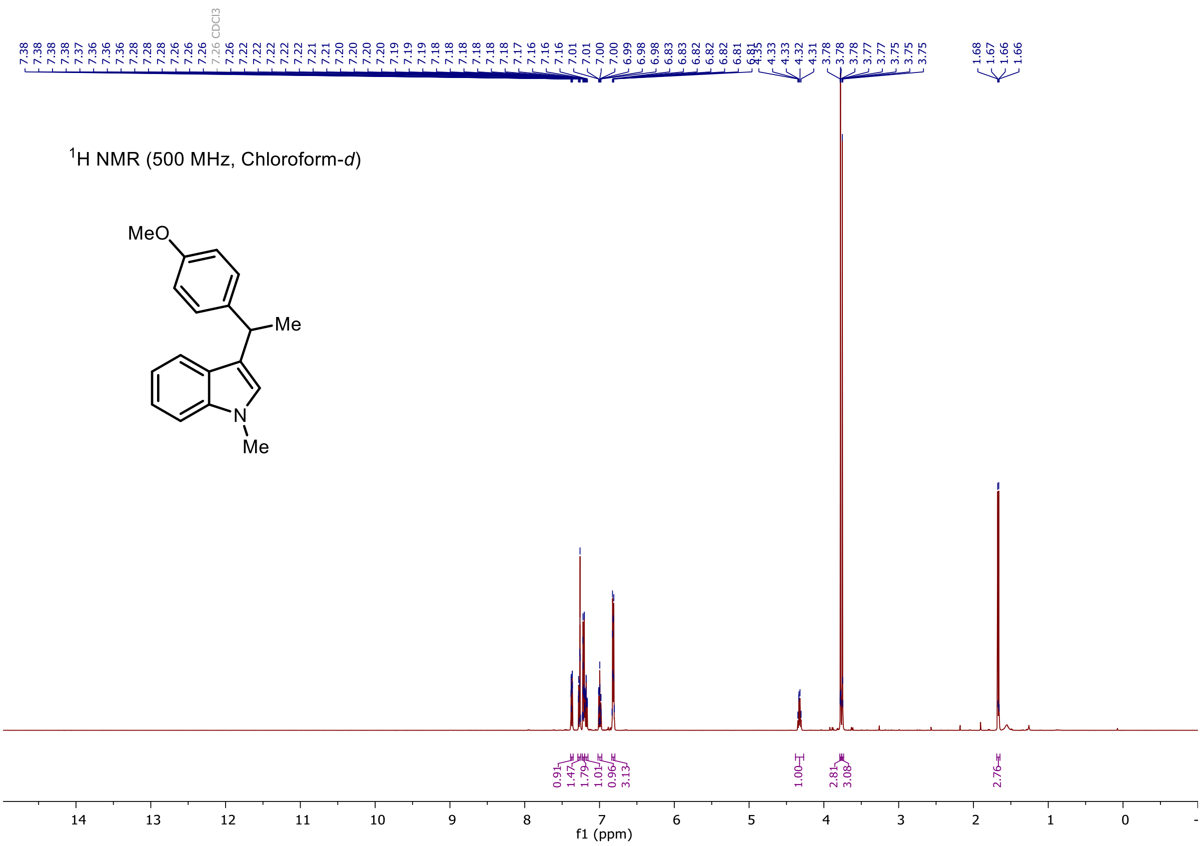
1H NMR (500 MHz, Chloroform-d)
MeO
Me
N
Me
f1 (ppm)

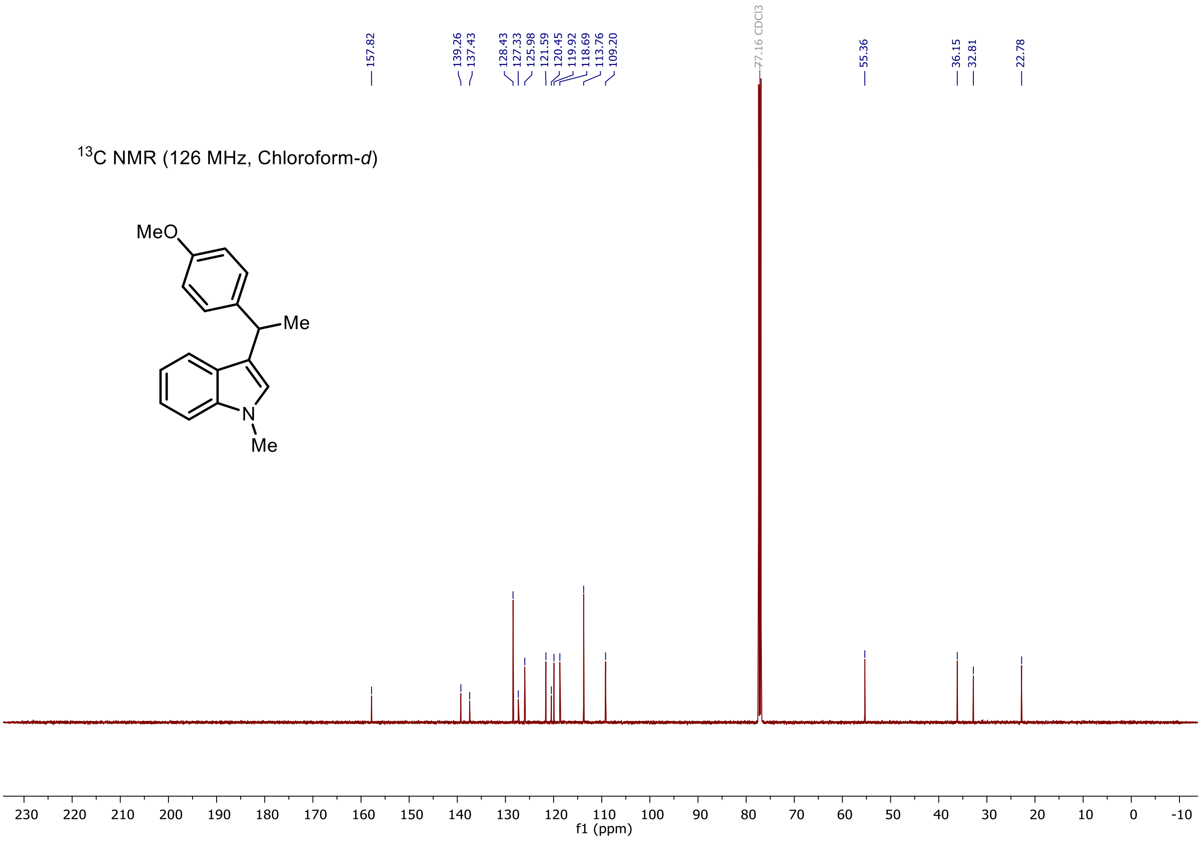
157.82
139.26
137.43
128.43
127.33
125.98
121.59
120.45
119.92
118.69
113.76
109.20
77.16 CDCl3
55.36
36.15
32.81
22.78
13C NMR (126 MHz, Chloroform-d)
MeO
Me
N
Me
f1 (ppm)

2.8. Scale up experiment

1,2-dimethyl-3-(1-phenylethyl)-1H-indole (2)

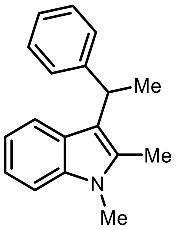

The title compound was prepared according to general procedure 6 using 1,2-dimethylindole (145 mg, 1 mmol), 4-methoxy-N-(4-methoxyphenyl)-N-(1-phenylethyl)aniline (400 mg, 1.2 mmol), $B(C_6F_5)_3$ (50 mg, 0.1 mmol) and Et_3SiH (32 µL, 0.02 mmol) in DCE (0.5 mL) at 50 ° C for 18 h and obtained in 66% spectroscopic yield. Purification by flash column chromatography on silica gel (eluent = 2.5-5% EtOAc in petroleum ether) followed by preparative TLC (eluent = 2.5% EtOAc in petroleum ether) gave the titled compound as red liquid (118 mg, 47%).

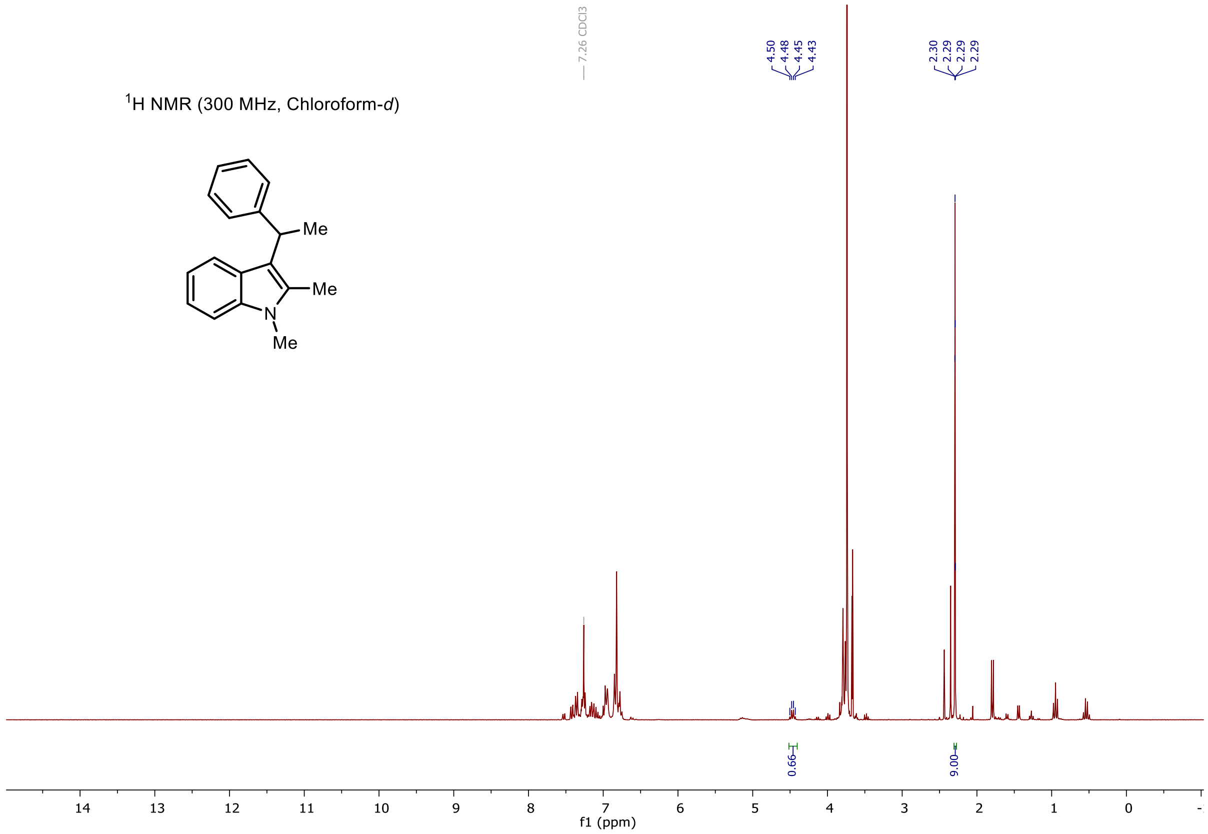

2.9. Mechanistic studies

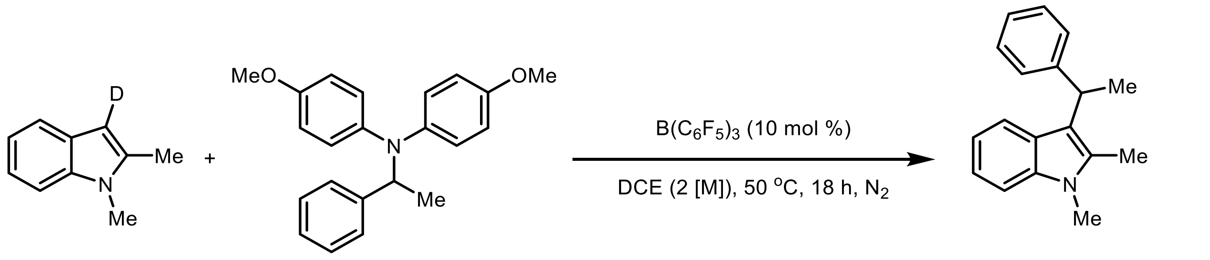

28, 75% D 7, 1.2 equiv. 2, 54%, <2% D

The experiment was conducted according to general procedure 6 using 1,2-dimethyl-1*H*-indole-3-*d* (29) (14.6 mg, 0.1 mmol, 75% D) and 4-methoxy-N-(4-methoxyphenyl)-N-(1-phenylethyl)aniline (7) (40 mg, 0.12 mmol). Yield determined by crude ^{1}H NMR using 1,3,5-trimethylbenzene as internal standard: 54%

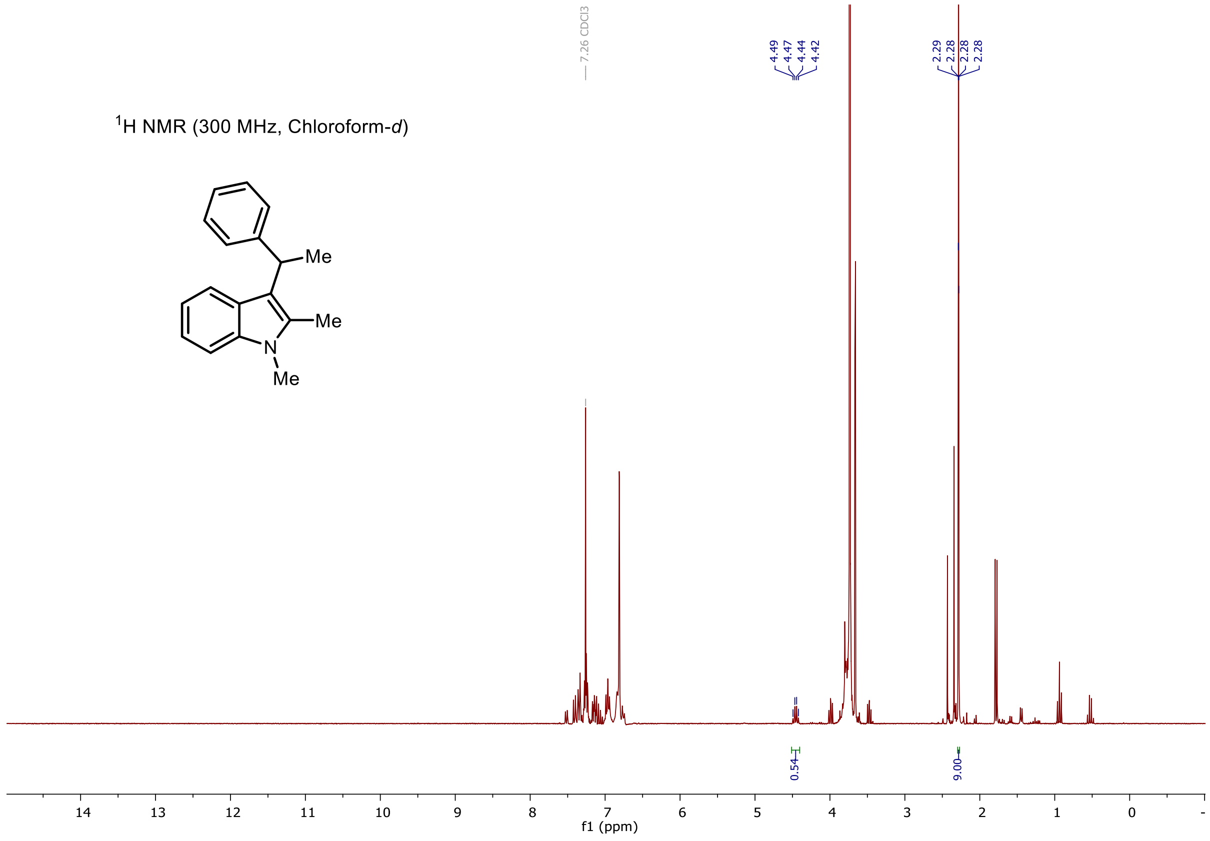

1,2-dimethyl-3-(1-phenylethyl-1-d)-1H-indole (30)

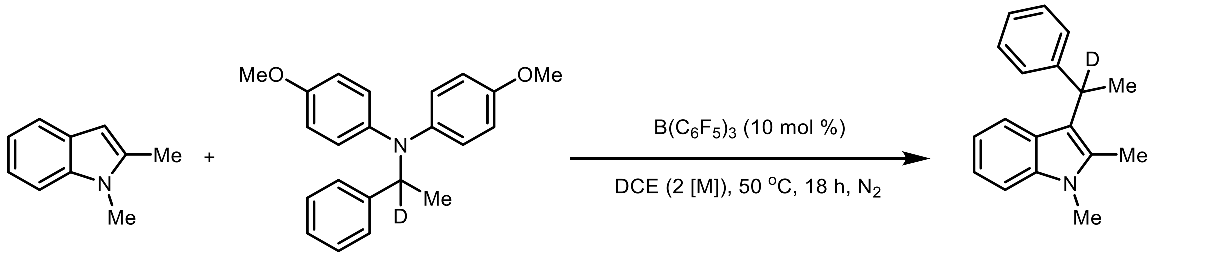

1 29, >99% D, 1.2 equiv. 30, 61%, >98% D

The experiment was conducted according to general procedure 6 using 1,2-dimethyl-1*H*-indole (14.5 mg, 0.1 mmol) and 4-methoxy-N-(4-methoxyphenyl)-N-(1-phenylethyl-1-d)aniline (30) (40 mg, 0.12 mmol, >99% D). Yield determined by crude ^{1}H NMR using 1,3,5-trimethylbenzene as internal standard: 61%

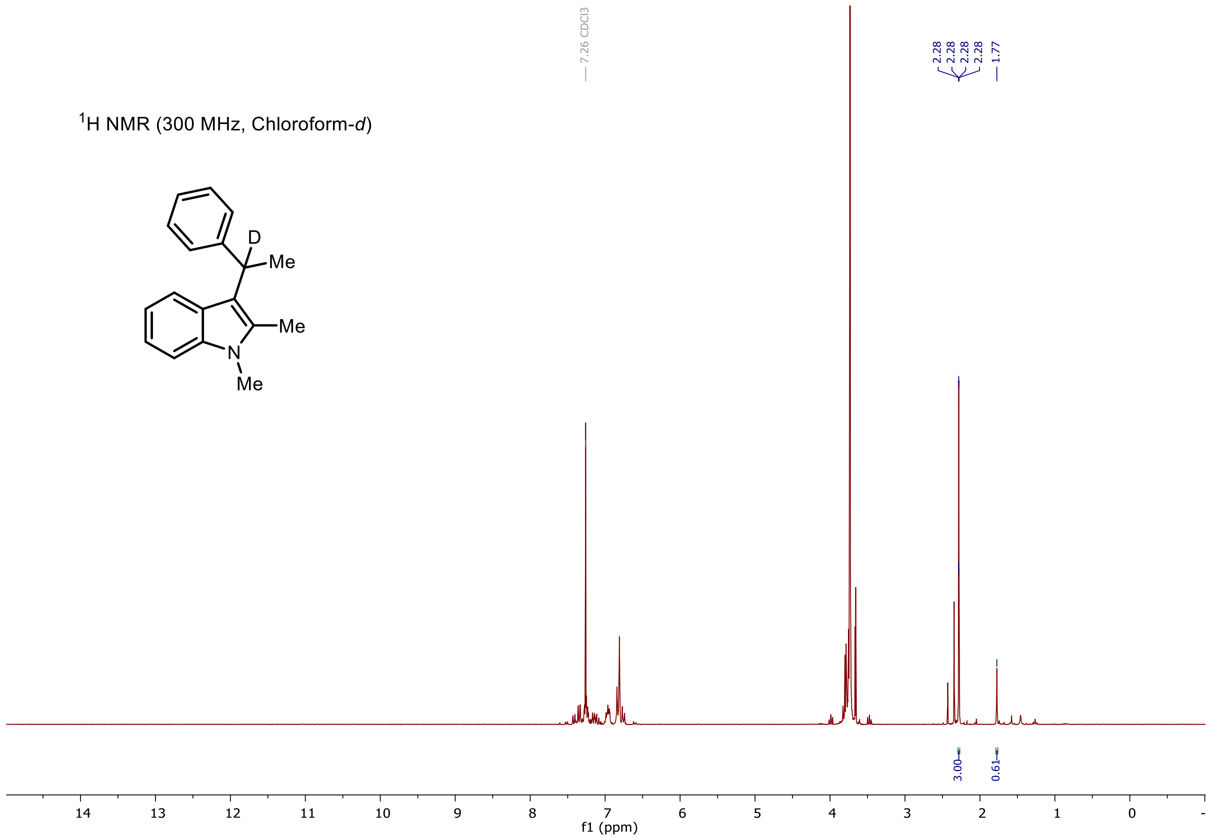

Deuterium incorporation was determined by ^{1}H NMR after purification by a preparative TLC (eluent = 2.5% EtOAc in petroleum ether); vmax / cm-1 (film) 2980, 1471, 1251, 1151, 952, 736, 698, 418; **^{1}H NMR (500 MHz, Chloroform-*d*)** δ 7.42 (dt, *J* = 7.9, 1.0 Hz, 1H), 7.36 – 7.32 (m, 2H), 7.26 – 7.22 (m, 3H), 7.17 – 7.13 (m, 1H), 7.13 – 7.09 (m, 1H), 6.97 (ddd, *J* = 8.0, 7.0, 1.0 Hz, 1H), 3.66 (s, 3H), 2.34 (s, 3H), 1.77 (s, 3H); **^{13}C{^{1}H} NMR (126 MHz, Chloroform-*d*)** δ 146.5, 136.9, 132.6, 129.0, 128.2, 127.5, 125.6, 120.4, 119.5, 118.7, 115.5, 108.7, 29.6, 20.8, 10.7; HRMS (CI-QUADRUPOLE) calculated $[C_{18}H_{18}{}^2HN]^+$ (M^+) m/z 250.1575, found 250.1576.

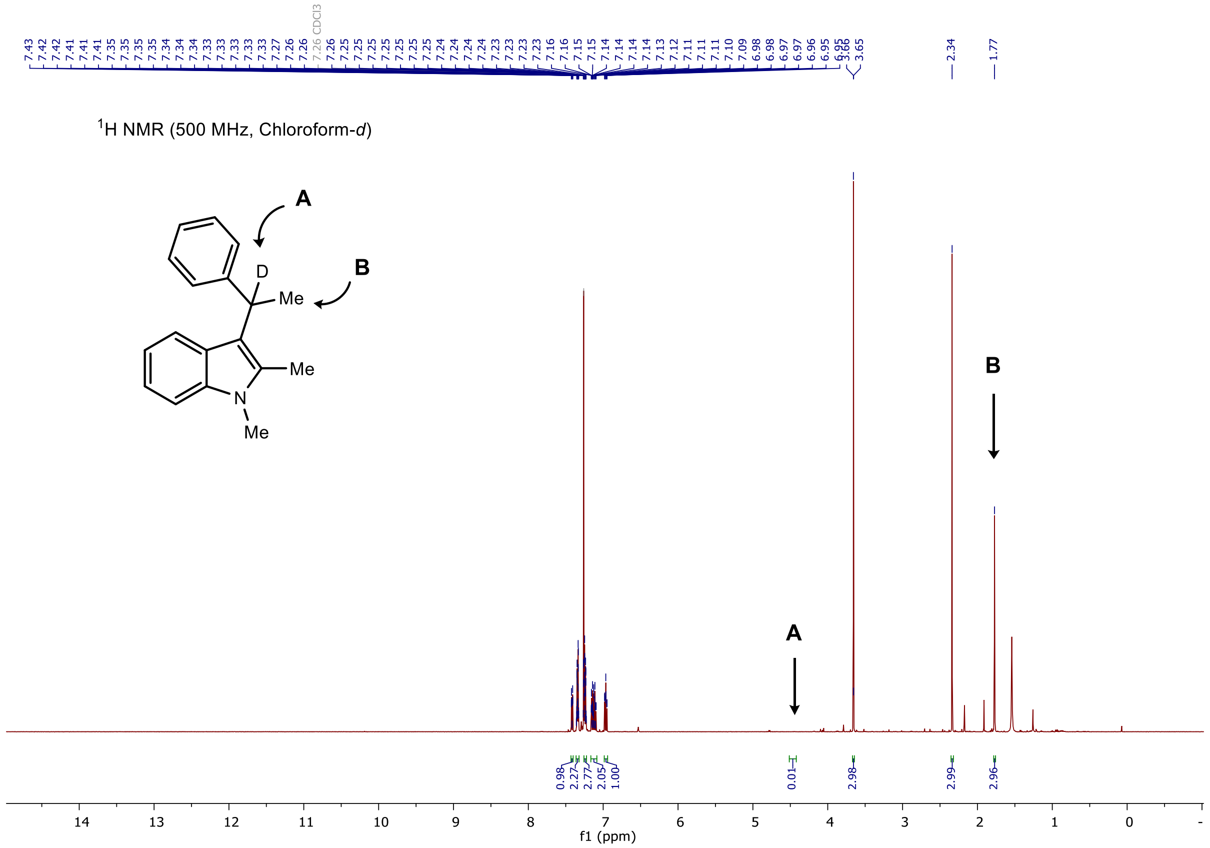

1H NMR (500 MHz, Chloroform-d)
A
B
D
Me
Me
N
Me
2.34
1.77
0.98
2.27
2.77
2.05
1.00
0.01
2.98
2.99
2.96
f1 (ppm)

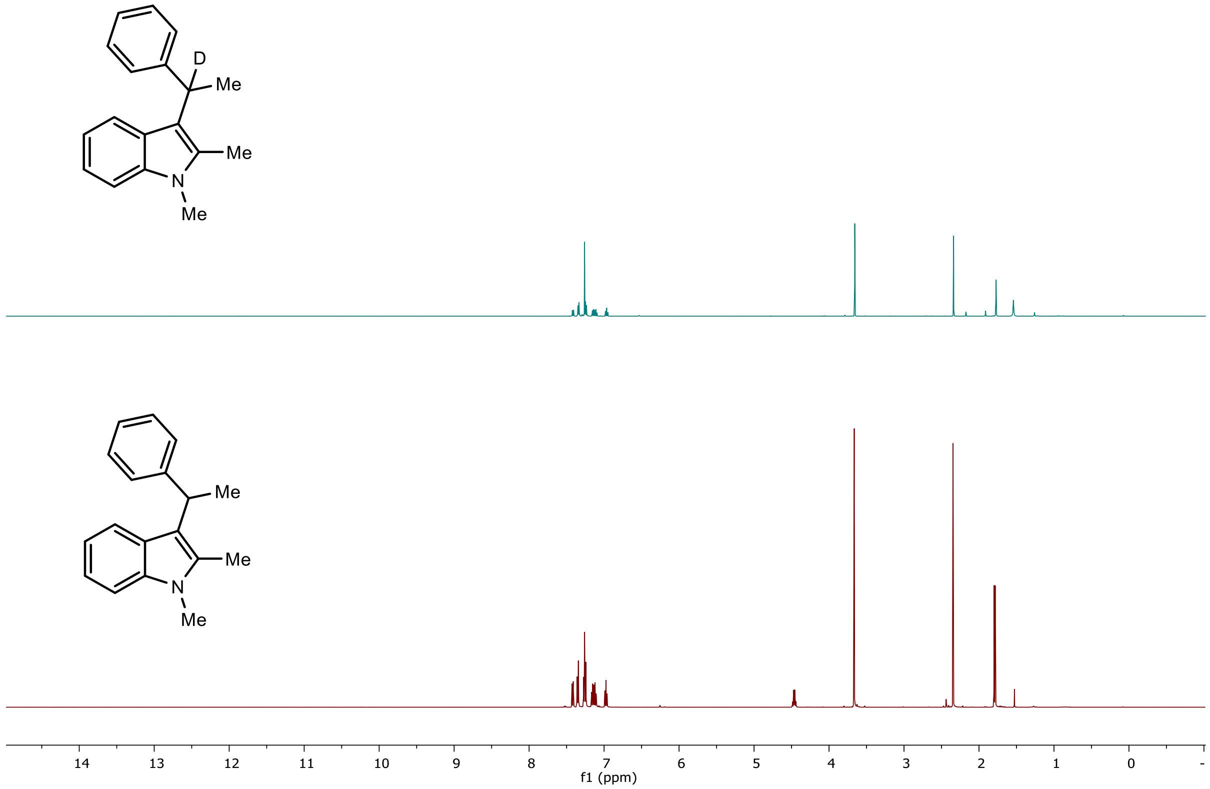

D
Me
Me
N
Me
Me
Me
N
Me
f1 (ppm)

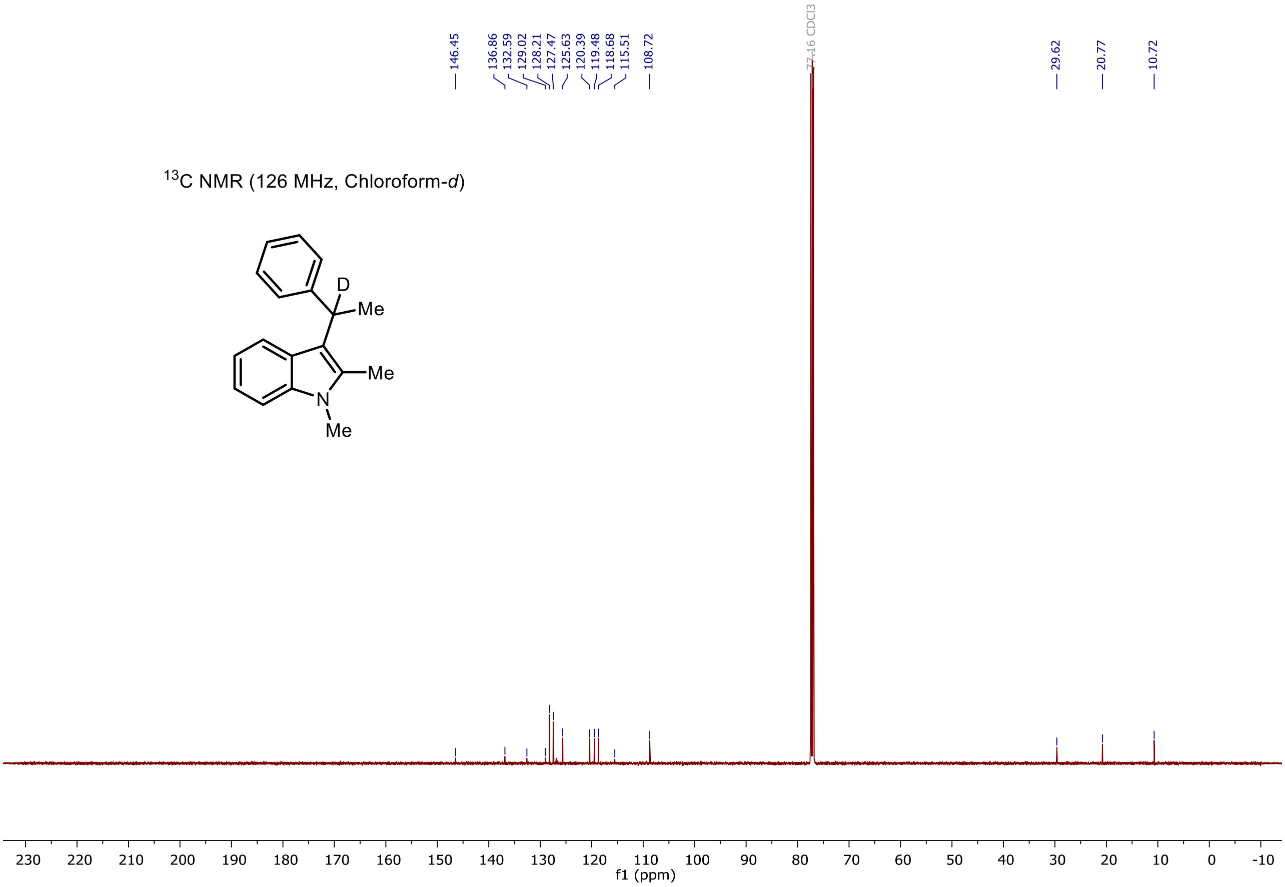

146.45
136.86
132.59
129.02
128.21
127.47
125.63
120.39
119.48
118.68
115.51
108.72
77.16 CDCl3
29.62
20.77
10.72
13C NMR (126 MHz, Chloroform-d)
D
Me
Me
N
Me
230
220
210
200
190
180
170
160
150
140
130
120
110
100
90
80
70
60
50
40
30
20
10
0
-10
f1 (ppm)

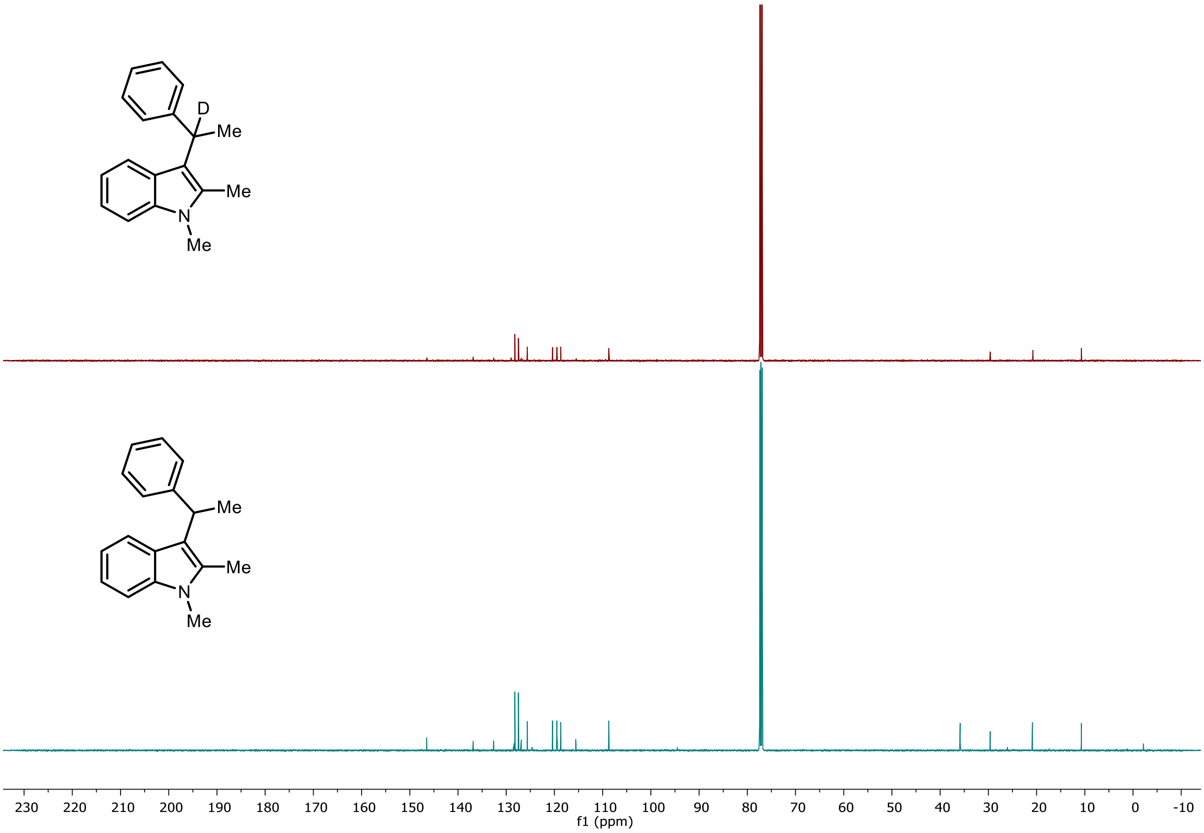

D
Me
Me
N
Me
Me
Me
N
Me
230
220
210
200
190
180
170
160
150
140
130
120
110
100
90
80
70
60
50
40
30
20
10
0
-10
f1 (ppm)

3. References

1. Estopiñá-Durán, S.; Mclean, E. B.; Donnelly, L. J.; Hockin, B. M.; Taylor, J. E. Arylboronic Acid-Catalyzed C-Alkylation and Allylation Reactions Using Benzylic Alcohols. *Org. Lett.* **2020**, *22*, 7547-7551.
2. Chakraborti, G.; Paladhi, S.; Mandal, T.; Dash, J. "On Water'' Promoted Ullmann-Type C−N Bond-Forming Reactions: Application to Carbazole Alkaloids by Selective N-Arylation of Aminophenols. *J. Org. Chem.* **2018**, *83*, 7347−7359.
3. Micksch, M.; Tenne, M.; Strassner, T. Synthesis of 1,2-Diaryl- and 1-Aryl-2-alkylimidazoles with Sterically Demanding Substituents. *Eur. J. Org. Chem.* **2013**, 6137-6145.
4. Nayal, O.; Bhatt, V.; Sharma, S.; Kumar, N. Chemoselective Reductive Amination of Carbonyl Compounds for the Synthesis of Tertiary Amines Using SnCl2·2H2O/PMHS/MeOH. *J. Org. Chem.* **2015**, *80*, 5912-5918
5. Nowrouzi, N.; Jonaghani, M. Highly selective mono-N-benzylation and amidation of amines with alcohols or carboxylic acids using the Ph2PCl/I2/imidazole reagent system. *Can. J. Chem.* **2012**, *90*, 498-509.
6. Muramatsu, W.; Nakano, K.; Li, C. Direct sp3 C-H Bond Arylation, Alkylation, and Amidation of Tetrahydroisoquinolines Mediated by Hypervalent Iodine(III) under Mild Conditions. *Org. Biomol. Chem.* **2014**, *12*, 2189-2192.
7. Xiong, Y.; Zhang, G. Visible Light-Induced Copper-Catalyzed Intermolecular Markovnikov Hydroamination of Alkenes. *Org. Lett.* **2019**, *21*, 7873-7877.
8. Basak, S.; Alvarez-Montoya, A.; Winfrey, L.; Melen, R. L.; Morrill, L. C.; Pulis, A. P. $B(C_6F_5)_3$-Catalyzed Direct C3 Alkylation of Indoles and Oxindoles. *ACS Catal.* **2020**, *10*, 4835-4840
9. Wen, Z.; Xu, J.; Wang, Z.; Qi, H.; Xu, Q.; Bai, Z.; Zhang, Q.; Bao, K.; Wu, Y.; Zhang, W. 3-(3,4,5-Trimethoxyphenylselenyl)-1H-Indoles and Their Selenoxides as Combretastatin A-4 Analogs: Microwave-Assisted Synthesis and Biological Evaluation. *Eur. J. Med. Chem.* **2015**, *90*, 184−194.
10. Kuyper, L. F.; Baccanari, D. P.; Jones, M. L.; Hunter, R. N.; Tansik, R. L.; Joyner, S. S.; Boytos, C. M.; Rudolph, S. K.; Knick, V.; Wilson, H. R.; Caddell, J. M.; Friedman, H. S.; ComLey, J. C. W.; Stables, J. N. High-Affinity Inhibitors of Dihydrofolate Reductase: Antimicrobial and Anticancer Activities of 7,8-Dialkyl-1,3-diaminopyrrolo[3,2-f]quinazolines with Small Molecular Size. *J. Med. Chem.* **1996**, *39*, 892−903.
11. Zhang, J.; Kohlbouni, S. T.; Borhan, B. Cu-Catalyzed Oxidation of C2 and C3 Alkyl Substituted Indole via Acyl Nitroso Reagents. *Org. Lett.* **2019**, *21*, 14-17.
12. Trabbic, C. J.; George, S. M.; Alexander, E. M.; Du, S.; Offenbacher, J. M.; Crissman, E. J.; Overmeyer, J. H.; Maltese, W. A.; Erhardt, P. W. Synthesis and Biological Evaluation of Isomeric Methoxy Substitutions on AntiCancer Indolyl-Pyridinyl-Propenones: Effects on Potency and Mode of Activity. *Eur. J. Med. Chem.* **2016**, *122*, 79-91.
13. Dasgupta, A.; Babaahmadi, R.; Slater, B.; Yates, B. F.; Ariafard, A.; Melen R. L. Borane-Catalyzed Stereoselective C–H Insertion, Cyclopropanation, and Ring-Opening Reactions. *Chem.* **2020**, *6*, 2364-2381.
14. Tsuchimoto, T.; Kanbara, M. Reductive Alkylation of Indoles with Alkynes and Hydrosilanes under Indium Catalysis. *Org. Lett.* **2011**, *13*, 912 −915.

15. Zhang, Z.; Wang, X.; Widenhoefer, R. Platinum(ii)-catalyzed intermolecular hydroarylation of unactivated alkenes with indoles. *Chem. Commun.* **2006**, 3717-3719
16. Esezobor, O. Z.; Zeng, W.; Niederegger, L.; Grubel, M.; Hess, C. R. Co–Mabiq Flies Solo: Light-Driven Markovnikov-Selective C- and N-Alkylation of Indoles and Indazoles without a Cocatalyst. *J. Am. Chem. Soc.* **2022**, *7*, 2994-3004.
17. Gao, F.; Ferlin, F.; Bai, R.; Li, M.; Vaccaro, L.; Gu, Y. Replacing halogenated solvent by a butyl acetate solution of bisphenol S in the transformations of indoles. *Green Chem.* **2012**, *23*, 3588-3594.
